# Highly Efficient Organocatalytic House‐Meinwald Rearrangement for the Facile Synthesis of Aldehydes: Swift Access to Ibuprofen

**DOI:** 10.1002/chem.202502982

**Published:** 2025-12-12

**Authors:** Friedemann Dressler, Ihssane El Fdali, Luisa L. Ciezarek, Peter R. Schreiner

**Affiliations:** ^1^ Institute of Organic Chemistry Justus Liebig University Giessen Giessen Germany; ^2^ EaStCHEM, School of Chemistry University of St. Andrews St. Andrews United Kingdom

**Keywords:** aldehyde synthesis, House‐Meinwald rearrangement, ibuprofen, organocatalysis

## Abstract

We present a new organocatalytic method for synthesizing aldehydes under mild conditions using readily accessible terminal epoxides as starting materials and bis(trifluoromethane)sulfonimide (Tf2NH) as the catalyst. We identified intermediate aldehyde dimerization products at lower temperatures and observed their cleavage at 55°C. We isolated the products in yields of 89–97% using catalyst loadings as low as 0.5 mol%. To underline the applicability of our new approach, we synthesized ibuprofen in a three‐step process and overall yield of 90%.

## Graphical abstract

We use readily available epoxides and 0.5 mol% bis(trifluoromethane)sulfonimide (Tf_2_N) as a catalyst to synthesize a broad range of aldehydes in yields ranging from 89% to 97%. Furthermore, we identified aldehyde dimerization intermediates at lower temperatures and observed their cleavage at 55 °C. To demonstrate the applicability of our new approach, we synthesized ibuprofen in a three‐step process with an overall yield of 90%.

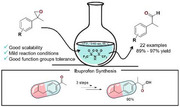

## 1 Introduction

**SCHEME 1:**
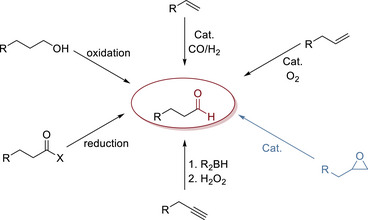
Common procedures for the synthesis of aldehydes.

The most established industrial processes for the synthesis of aldehydes include the formylation of arenes and the hydroformylation of olefins [1, 2, 3, 4]. An alternative prominent method consists of the Tsuji–Wacker oxidation, widely employed in the synthesis of acetaldehyde [5]. However, this reaction lacks regioselectivity when applied to unsymmetrically substituted olefins, leading to the formation of ketones as the predominant oxidation product [6]. Moreover, all mentioned processes hold some drawbacks, such as a limited substrate scope or the use of expensive metal‐based catalysts in the hydroformylation and the Tsuji–Wacker oxidation, such as Pd‐ and Rh‐complexes [7]. In the laboratory, the most common procedures for aldehyde synthesis include the oxidation of alcohols [8, 9], the reduction of carboxyl derivatives [10, 11], and the formylation of arenes [12]. An alternative pathway for the synthesis of aldehydes exploits the rearrangement of epoxides to carbonyls (Scheme 1) [13].

This redox neutral rearrangement has been known since the early 20th century, when Klages [14], Stoermer [15], Meerwein [16], as well as Danilow and Venus‐Danilowa [17] independently reported the rearrangement of epoxides to aldehydes and ketones. These authors primarily used Brønsted acids to promote the epoxide rearrangement or observed the formation of carbonyl compounds as side products during epoxidation reactions. It was not until the 1950s that House conducted a comprehensive investigation into the epoxide rearrangement to carbonyl compounds, using Lewis acids as catalysts for the rearrangement of *cis*‐ and *trans*‐stilbene oxides [18, 19]. A few years later, Meinwald reported his observations on the epoxide rearrangement of norbornadiene derivatives [20, 21]. Since then, the epoxide rearrangement, nowadays known as House–Meinwald rearrangement, has been well established and used in the total synthesis of various natural products, for example, fredericamycin A [22] and cuparenone derivatives [23]. Despite being known for almost 100 years, this redox‐neutral rearrangement has yet to become a standard method for the synthesis of simple aldehydes. The drawbacks of the House–Meinwald rearrangement are frequently observed low chemo‐ and regioselectivity [24, 25, 26], along with the frequent requirement of stoichiometric amounts of a promoter [22, 24], or high catalyst loading [27, 28, 29] to ensure full conversion and suppress side reactions. Attempts to use substoichiometric amounts of a promoter frequently resulted in the formation of side products. Thus, often the aldehydes are not isolated, but reduced *in situ* to the corresponding alcohols [30, 31] or transfer into the corresponding amines [32, 33] to circumvent side reactions, such as dimerization and further oxidation. Nevertheless, a few recent procedures have emerged using catalytic amounts of Lewis or Brønsted acid catalysts in the House–Meinwald rearrangement for the synthesis of aldehydes. One drawback is that these procedures often require anhydrous conditions [25, 34, 35, 36, 37]. Here, we report a metal‐free alternative for the synthesis of aldehydes under ambient conditions. The products were obtained in high yields starting from 2,2‐disubstituted epoxides using catalyst loadings as low as 0.5 mol%.

## 2 Results and Discussion

**SCHEME 2:**
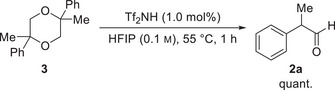
Cleavage of the dimerization side product.

We chose 2‐methyl‐2‐phenyloxirane **1a** as test substrate and CH_2_Cl_2_ as solvent for catalyst screening. CH_2_Cl_2_ has already been used in Lewis as well as Brønsted acid catalyzed House–Meinwald rearrangements [36, 38, 39]. We used 5 mol% of readily available sulfone amides and observed that these Brønsted acids lacked activity, as the conversion was always lower than 10%, even at prolonged reaction times and increased temperatures (Table 1, entries 1–3). The low reactivity could be attributed to the low acidity, as we observed similar results in a previous study [36]. Thus, the corresponding more acidic sulfonic acids resulted in complete epoxide conversion yielding 2‐phenylpropionaldehyde **2a** in yields between 28% and 51%. However, we also detected the formation of dimers as side products via GC‐MS analysis (entries 4–6). The use of the corresponding sulfone imides slightly increased the yields of the aldehyde compared to the sulfonic acids (entries 7–9), reaching 55% of **2a** using Tf_2_NH. This finding is consistent with our previous observations that sulfone imides have superior reactivity to the corresponding sulfonic acids. We screened various polar and nonpolar solvents to further optimize the reaction. We also reduced the catalyst loading to 2.5 mol% and shortened the reaction time to 1 h, since we observed that these conditions are suitable to get full epoxide conversion (entries 10–15). The use of Et_2_O and *t*BuOMe furnished **2a** in 70% and 80% yield, respectively (entries 10 and 11). The use of THF gave only 24% of the desired product, with formation of several side products such as dimers, detected via GC‐MS analysis (entry 13). Ethyl acetate and *n*‐hexane furnished comparable results to CH_2_Cl_2_ (entries 13 and 14). Considering that Moran and Lebœuf reported that hexafluoroisopropanol (HFIP) promotes the isomerization and *in situ* hydrosilylation of epoxides using a Brønsted acid catalyst, we also tested this solvent, receiving 93% of the desired aldehyde (entry 15). We decide to further optimize the reaction using HFIP as solvent and used 58 °C reaction temperature obtaining almost pure product quantitatively [31]. Moreover, we observed that we could decrease the reaction time to 15 min (entry 16). To further optimize the reaction, we decreased the catalyst loading to 1.0 mol% (entry 17) and then even to 0.5 mol% (entry 18), still obtaining quantitative yield. Using 0.1 mol% catalyst, we observed product formation of 92% (entry 19). To investigate the influence of the reaction temperature, we decided to use 0.5 mol% Tf_2_NH and run the reaction at room temperature (entry 20). Besides the formation of 50% of the desired aldehyde **2a**, we observed the formation of some side products via GC‐MS und NMR analyses and isolated as a major side product 1,4‐dioxane **3** as a set of diastereomers in a combined yield of 30%. Literature precedents describe the formation of these dimers roughly 100 years ago as side products in epoxide rearrangements [14, 15, 17, 40], and Danilow and Venus‐Danilowa described a method to cleave **3** into **2a** using aqueous hydrochloric acid at a reaction temperature up to 180 °C [17]. Since Tf_2_NH is a much stronger Brønsted acid than HCl and HFIP is an excellent hydrogen bond donor, we envisioned that the cleavage of the dioxanes could take place here at 55 °C [41, 42, 43]. Pleasingly, we subjected **3** to our reaction conditions and obtained **2a** in quantitative yield (Scheme 2). We observed traces of product in a blind reaction, which demonstrated the crucial role of the catalyst in the rearrangement (entry 21).

**TABLE 1:** Optimization of reaction conditions for the formation of 2‐phenyl‐propanal.

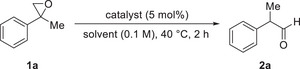			

Entry	Solvent	Catalyst [mol%]	Yield [%]^[^ ^a^ ^]^
1^[^ ^b^ ^]^	CH_2_Cl_2_	TsNH_2_ (5)	8
2^[^ ^b^ ^]^	CH_2_Cl_2_	MsNH_2_ (5)	7
3^[^ ^b^ ^]^	CH_2_Cl_2_	TfNH_2_ (5)	8
4	CH_2_Cl_2_	TsOH (5)	28
5	CH_2_Cl_2_	MsOH (5)	23
6	CH_2_Cl_2_	TfOH (5)	51
7	CH_2_Cl_2_	Ts_2_NH (5)	43
8	CH_2_Cl_2_	Ms_2_NH (5)	34
9	CH_2_Cl_2_	Tf_2_NH (5)	55
10^[^ ^c^ ^]^	Et_2_O	Tf_2_NH (2.5)	80
11^[^ ^c^ ^]^	*t*BuOMe	Tf_2_NH (2.5)	70
12^[^ ^c^ ^]^	THF	Tf_2_NH (2.5)	24
13^[^ ^c^ ^]^	EtOAc	Tf_2_NH (2.5)	54
14^[^ ^c^ ^]^	*n*‐hexane	Tf_2_NH (2.5)	53
15^[^ ^c^ ^]^	HFIP	Tf_2_NH (2.5)	93
16^[^ ^[d]^, ^[e]^ ^]^	HFIP	Tf_2_NH (2.5)	> 97
17^[^ ^[d]^, ^[e]^ ^]^	HFIP	Tf_2_NH (1.0)	> 97
18^[^ ^[d]^, ^[e]^ ^]^	HFIP	Tf_2_NH (0.5)	96
19^[^ ^e^ ^]^	HFIP	Tf_2_NH (0.1)	92
20^[^ ^[d]^, ^[f]^ ^]^	HFIP	Tf_2_NH (0.5)	50
21^[^ ^e^ ^]^	HFIP	none	Traces

Reaction conditions: 0.2 mmol scale, 2 mL solvent.

^[a]^ Yields determined by quantitative ^1^H NMR using 0.1 eq. *p*‐nitrobenzaldehyde as internal standard.

^[b]^ Reaction time was 24 h.

^[c]^ Reaction time was 1 h.

^[d]^ Reaction time was 15 min.

^[e]^ Reaction temperature was 58°C.

^[f]^ Reaction was run at room temperature.

**SCHEME 3:**
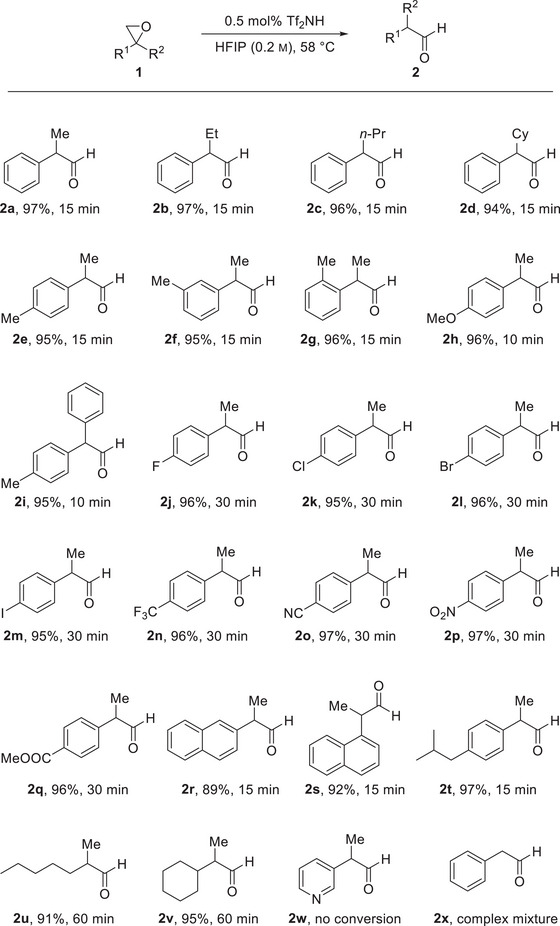
Scope and limitation of the triflimide‐catalyzed House–Meinwald rearrangement. Reaction conditions: 2.0 eq. 1, 0.01 mmol Tf_2_NH, 10 mL HFIP (0.1 m), 55°C, 10–60 min. Yields refer to pure, isolated products.

Having identified the optimal reaction conditions (cf. entry 17), we then explored the scope and limitations of the reaction using various 2,2‐disubstituted epoxides (**1**) (Scheme 3). All substrates were easily accessible via Corey–Chaykovsky reaction in very high yields (see Supporting Information for details) [44]. We observed that the variation of the alkyl substituent from methyl to ethyl, *n*‐propyl, and cyclohexyl did not change the reactivity and resulted in the formation of aldehydes **2a**–**2d** with yields between 94% and 97%. The use of methyl‐substituted derivatives **2e**–**2g** showed no influence of the substitution pattern on the phenyl moiety as we obtained the products consistently with exceptional yields of 95–96%. The *para*‐methoxy substituted epoxide **1h** and bis‐aryl epoxide **1i** showed the highest reactivity and furnished aldehydes **2h** and **2i** in 96% and 95%, respectively, after a reduced reaction time of 10 min. We reason that, due to the stabilization of the benzyl cation intermediate, the epoxide opening occurs with a higher reaction rate, which is consistent with our observation that epoxides substituted with an electron‐withdrawing group required a reaction time of 30 min. We isolated the corresponding aldehydes **2j**–**2q** in yields of 95–97%. Our procedure is also tolerant to the ester moiety in aldehyde **2q**, which was not cleaved under the reaction conditions. We obtained the naphthyl‐substituted derivatives **2r** and **2s** in yields of 89% and 92%, respectively. These aldehydes were the only products that required purification, whereas we obtained all other aldehydes in analytically pure form after simple extraction from the reaction mixture (see Supporting Information for details). After the investigation of aryl‐substituted substrates, we focused on the synthesis of bis‐alkyl substrates **2u** and **2v**. We observed that these substrates required a slightly prolonged reaction time of 60 min, but even in these cases, it gave the desired aldehydes **2u** and **2v** in very good yields of 91% and 95%, respectively. In an effort to further expand the substrate scope, we investigated pyridine‐derived epoxide **1w**, but observed no conversion. We attribute this to a fast acid‐base reaction and the formation of a pyridinium triflimide. We tested mono‐substituted epoxides, using epoxide **1x**. However, this reaction produced a complex mixture of dimers and higher oligomers, yielding only trace amounts of the desired product, detected via NMR analysis. This finding underlines that, although our approach shows applicability to various 2,2‐disubstituted epoxides, developing a general approach for organocatalytic epoxide rearrangement remains challenging [25, 38, 45, 46].

**SCHEME 4:**
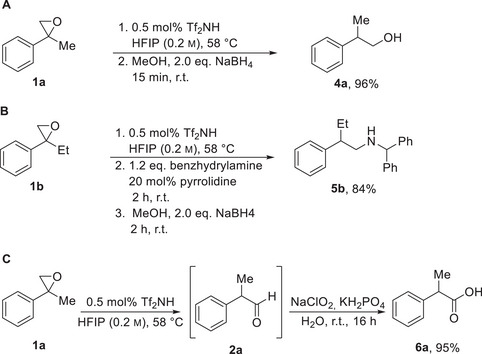
Subsequent transformation of the aldehyde function in a one‐pot sequence (A and B) and in a two‐step process (C). Yields refer to pure, isolated products.

To demonstrate the utility of our method, we further derivatized the aldehyde moiety into various functional groups. We synthesized 2‐phenyl‐1‐propanol **4a** in 96%, without isolation of the intermediate aldehyde by addition of methanol as cosolvent and using NaBH_4_ as reductant [38]. Accordingly, we successfully carried out a reductive amination, using the organocatalytic protocol described by Lledós, Riera, and co‐workers, and obtained amine **5b** in 84% yield [32]. This allows the isolation of stable and easy to handle amines, opening the possibility of synthesizing an extensive range of secondary amines with a one‐pot transformation, using pyrrolidine as an imine‐formation catalyst [47, 48, 49]. Additionally, we tried the synthesis of the corresponding carboxylic acid **6a** in a one‐pot reaction using Oxone, but observed a yield of only 60% of the desired carboxylic acid and some unidentified side products [50]. Therefore, we subjected **2a** to a Pinnick oxidation in a two‐step process and obtained 2‐phenylpropionic acid (6a) in a yield of 95% (Scheme 4) [51].

**SCHEME 5:**
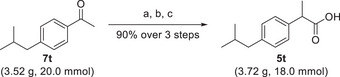
Gram scale synthesis of ibuprofen using the organocatalytic House–Meinwald rearrangement as key step. Reaction conditions: (a) 1.2 eq. of Me_3_SOI, 1.1 eq. KOtBu, DMSO, room temperature, 36 h, (b) 0.2 mol% of Tf_2_NH, HFIP, 58 °C, and 45 min, (c) 1.2 eq. of NaOCl, 1.2 eq. of KH_2_PO_4_, tBuOH/H_2_O 3:2, room temperature, and 18 h. Yield refers pure, isolated product.

This allows this reaction sequence to be employed in the synthesis of a wide range of 2‐arylpropionic acid derivatives with pharmaceutical applications as nonsteroidal anti‐inflammatory drugs (NSAID), such as ibuprofen, ketoprofen, and naproxen [52]. Therefore, to further underline the applicability of our method, we envisioned the synthesis of ibuprofen on multigram scale and reduced catalyst loading of 0.2 mol% in the epoxide rearrangement. Starting from commercial 4'‐isobutylacetophenone **7t** and our established reaction sequence based on epoxidation—rearrangement—oxidation, we isolated pure ibuprofen **8t** in 90% yield (Scheme 5). This reaction sequence opens a metal‐free alternative to the industrial synthesis of ibuprofen and other derivatives [53].

## 3 Conclusion

In conclusion, we developed a new strategy for the synthesis of various α‐substituted aldehydes starting from abundantly available ketones in two steps via Corey–Chaykovsky reactions and House–Meinwald rearrangements. Our method tolerates various functional groups and we obtained the rearrangement products in very good yields ranging from 89% to 97%, using simple and easily accessible Tf_2_NH as a catalyst at loadings as low as 0.2 mol%. This means a turnover number of up to 485 and a turnover frequency of up to 1,152 h^–^^1^ (at 0.5 mol%), and highlights the effectiveness of our method. We demonstrate the applicability of our method through the direct conversion of the aldehyde functionality in the rearrangement products to various groups such as alcohols and amines. Furthermore, we developed a new reaction sequence for the synthesis of ibuprofen on multigram scale and obtained it in 90% over three steps. This makes our method potentially suitable for industrial applications in the synthesis of active pharmaceutical ingredients. A natural extension of our work is currently underway in our laboratories, focusing on the use of chiral Brønsted acids as catalysts to enable enantioselective House–Meinwald rearrangements for the asymmetric synthesis of dexibuprofen, naproxen, and related NSAID derivatives [38, 39, 54].

## Author Contribution

Friedemann Dressler designed the study. Friedemann Dressler, Ihssane El Fdali, and Luisa Ciezarek carried out all experiments. The manuscript was written by FD. All authors have given approval to the final version of the manuscript. Peter R. Schreiner supervised the work.

## Conflicts of Interest

The authors declare no conflict of interest.

## Supporting information




**Supporting Information file** 1:The authors have cited additional references within the Supporting Information [1, 2, 3, 4, 5, 6, 7, 8, 9, 10, 11, 12, 13, 14, 15, 16, 17].
