# Supplementary material for: Highly Efficient Organocatalytic House‐Meinwald Rearrangement for the Facile Synthesis of Aldehydes: Swift Access to Ibuprofen
Source: Chemistry. 2025 Dec 12;32(2):e02982. doi: 10.1002/chem.202502982 (PMC12790323; doi:10.1002/chem.202502982)
Supplement: Supplementary file 1 — Supporting Information file 1:The authors have cited additional references within the Supporting Information [1, 2, 3, 4, 5, 6, 7, 8, 9, 10, 11, 12, 13, 14, 15, 16, 17]. [file CHEM-32-e02982-s001.pdf]

Supporting Information

Highly Efficient Organocatalytic House-Meinwald Rearrangement for the Facile Synthesis of Aldehydes: Swift Access to Ibuprofen

*Friedemann Dressler, Ihssane El Fdali, Luisa Ciezerek, and Peter R. Schreiner**
Institute of Organic Chemistry, Justus Liebig University Giessen, Heinrich-Buff-Ring 17, 35392 Giessen, Germany.

prs@uni-giessen.de

Content

1. General Remarks...3
2. Spectroscopy and Spectrometry...3
3. General Procedures...4
4. Overview of Synthesized Substances..5
5. Synthetic Procedures and Analytical Data..6
6. NMR Spectra...22
7. References..80

1. General Remarks

Unless otherwise specified, reagents were purchased from commercial suppliers (Sigma Aldrich, Carl Roth, Acros Organics, BLDpharm, or TCI) at the highest purity grade available and were used as received. All solvents were distilled prior to use. Dry and absolute solvents were ordered from Acros Organics (H_2O < 50 ppm). HFIP was distilled over 3 Å molecular sieve to remove water. All reactions involving air sensitive compounds were carried out under dry nitrogen or argon by means of an inert gas/reduced pressure double manifold line and standard Schlenk techniques. All reactions using organocatalysts were run by the use of metal-free stirring bars used only in metal-free reactions. Flash column chromatography was performed using silica gel 60 M (Macherey-Nagel; 0.040 – 0.063 mm, 230 – 400 mesh ASTM). Reaction control was performed *via* TLC-MS by an Advion expression® CMS or GC-MS analysis (Agilent 7820 GC with 5977B MSD). Analytical thin-layer chromatography was performed using precoated polyester sheets Polygram® SIL G/UV254 (Macherey-Nagel; 0.2 mm silica gel layer with fluorescent indicator). Visualization was accomplished by irradiation with UV light (λ = 254 nm) and/or ceric ammonium molybdate solution,[a] phosphomolybdic acid solution,[b] or potassium permanganate solution.[c]

[a] 5 g ammonium molybdate tetrahydrate, 2 g cerium ammonium sulfate dihydrate, 20 mL conc. H_2SO_4, 180 mL H_2O.

[b] 10 g phosphomolybdic acid, 100 mL EtOH.

[c] 1.5g $KMnO_4$, 10 g K_2CO_3, 200 mL H_2O.

2. Spectroscopy and Spectrometry

Nuclear Magnetic Resonance Spectroscopy: ^{1}H-NMR (400 MHz), ^{13}C-NMR (101 MHz), and ^{19}F-NMR (377 MHz) measurements were carried out using a Bruker AV 400 or Bruker AV 400 HD spectrometer at 298 K. Chemical shifts (δ) are given in parts per million (ppm) relative to the respective residual protium or carbon resonances of the deuterated solvent ($CDCl_3$: δ = 7.26 and 77.16 ppm; DMSO-d_6: δ = 2.50 and 39.52 ppm; CD_2Cl_2: δ = 5.32 and 53.84 ppm; C_6D_6 δ = 7.16 and 128.06 ppm). Data are reported as follows: chemical shift, multiplicity (s = singlet, d = doublet, t = triplet, q = quartet, sept = septet, m = multiplet, br = broad, or combinations thereof), coupling constants (Hz), integration. 19F-NMR spectra are given without reference. All spectral data are reported based on appearance.

Mass spectrometry: HRMS was performed on a Bruker Mikro-TOF or Bruker Impact II (ESI or APCI) by using methanol as the carrier.

3. General Procedures

General procedure 1 (GP1): Corey–Chaykovsky reactions

Following a literature procedure,[1] 3.80 g (17.25 mmol, 1.15 eq) trimethylsulfoxonium iodide was dissolved in 100 mL dry DMSO. Afterwards, 1.23 g (11.0 mmol, 1.1 eq.) KO*t*Bu was added and the solution was stirred for 45 min at room temperature. Then, 15.0 mmol (1.0 eq.) of the corresponding ketone was added portionwise and the reaction mixture was stirred for 36 h at room temperature. The reaction mixture was diluted with 150 mL water and extracted with ethyl acetate (6 x 20 mL). The combined organic phases were washed with brine (3 x 30 mL), dried over Na_2SO_4 and concentrated under reduced pressure. Purification by column chromatography using silica gel as stationary phase afforded the product, details are given for each substrate.

General procedure 2 (GP2): Tf_2NH catalyzed House-Meinwald Rearrangement

2.8 mg (0.01 mmol) Tf_2NH was dissolved in 10 mL HFIP and the reaction mixture was heated to 55 °C. Afterwards, 2.0 mmol epoxide was added slowly and the reaction mixture was stirred for the indicated time at 55 °C. Then, 10 mL saturated $NaHCO_3$ solution were added and the aqueous phase was extracted with *n*-hexane (5 x 5 mL). The combined organic phases were washed with brine (2 x 5 mL), dried over $MgSO_4$, and the solvent was removed under reduced pressure. Usually, the product was pure after simple extraction. Purification by column chromatography are mention for indicated products.

4. Overview of Synthesized Substances

Catalysts:

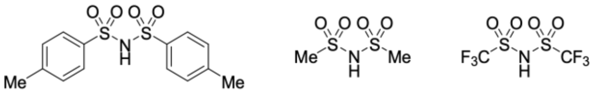

Ts₂NH **Ms₂NH** **Tf₂NH**

Epoxides:

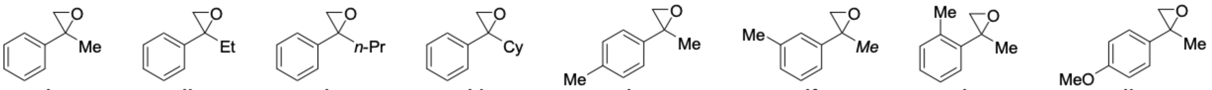

1a **1b** **1c** **1d** **1e** **1f** **1g** **1h**

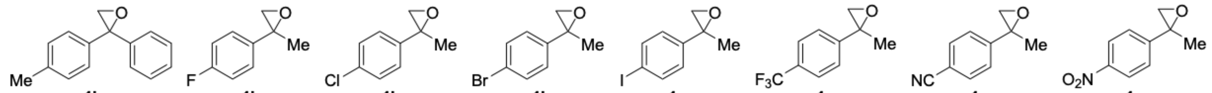

1i **1j** **1k** **1l** **1m** **1n** **1o** **1p**

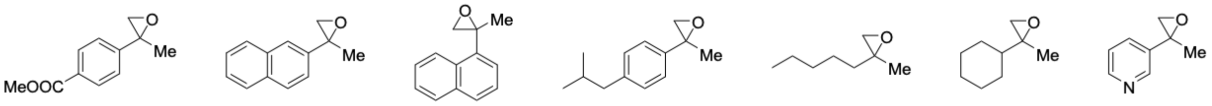

1q **1r** **1s** **1t** **1u** **1v** **1w**

Aldehydes:

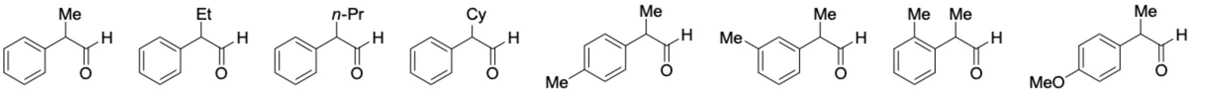

2a **2b** **2c** **2d** **2e** **2f** **2g** **2h**

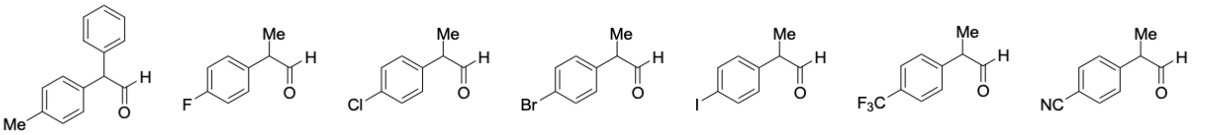

2i **2j** **2k** **2l** **2m** **2n** **2o**

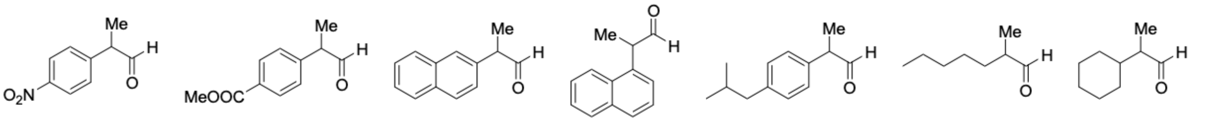

2p **2q** **2r** **2s** **2t** **2u** **2v**

Further products:

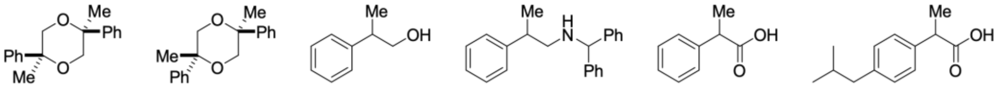

3a **3b** **4a** **5b** **6a** **6t**

5. Synthetic Procedures and Analytical Data

Bis(*para*-tosyl)imide

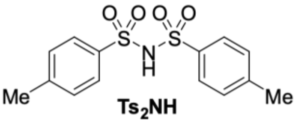

Ts_2NH

Ts_2NH was synthesized according to literature known procedures.[2] To a solution of 1.91 g (10.0 mmol, 1.0 eq.) *p*-toluenesulfonyl chloride and 1.88 g (11.0 mmol, 1.1 eq.) *p*-toluenesulfonamide in 20 mL toluene was added 0.24 g (2.0 mmol, 0.2 eq.) DMAP and 2.1 mL (1.53 g, 15.1 mmol, 1.5 eq.) triethylamine. The reaction mixture was heated at 70 °C overnight and then cooled to room temperature. The reaction was quenched with 2 M HCl (~ 25 mL) to reach pH = 1, and extracted with ethyl acetate (5 x 20 mL). The combined organic phases were dried over $MgSO_4$ and the solvent was removed under reduced pressure. The crude product mixture was purified *via* recrystallization using hot chloroform/*n*-hexane to afford 2.66 g (8.17 mmol, 81%) of the desired product as colorless needles.

^{1}H NMR (400 MHz, DMSO-d_6) δ/ppm = 7.56 (dt, *J* = 8.2, 2.1 Hz, 4H), 7.22 (dt, *J* = 8.2, 2.1 Hz, 4H), 4.97 (br s, 1H), 2.34 (s, 6H).

^{13}C NMR (101 MHz, DMSO-d_6) δ/ppm = 141.5, 141.1, 128.8, 126.5, 20.9.

IR (ATR, cm^{-1}): 3155 (w), 1594 (w), 1356 (m), 1164 (m), 1082 (m), 852 (m), 806 (m), 658 (s), 538 (s).

HRMS (ESI): m/z = 348.0332 $[M+Na]^+$ (calc. for $C_{14}H_{15}NO_4S_2Na^+$ m/z = 348.0335).

Note: Due to high water amount in the DMSO-d_6, the N−H peak is shifted and the integral appears larger, but the ^{13}C NMR fits perfectly with the literature.[2]

Bis(methanesulfonyl)imide

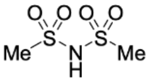

Ms_2NH

Ms_2NH was synthesized according to literature known procedures.[2] A solution of 1.90 g (20.0 mmol, 1.0 eq.) methanesulfonamide and 2.25 g (40.1 mmol, 2.0 eq.) KOH in 100 mL water was cooled to 0 °C. Then, 2.54 g (22.2 mmol, 1.1 eq.) methanesulfonyl chloride was added dropwise and the mixture was heated to 60 °C for 1o h. Afterwards, the reaction mixture was cooled to 0 °C and acidified with 30 mL conc. HCl. The reaction mixture was extracted with ethyl acetate (3 x 50 mL), dried over Na_2SO_4, and the solvent removed under reduced pressure. The crude product mixture was purified by recrystallization using hot chloroform/*n*-hexane to afford 1.93 g (11.1 mmol, 55%) of the desired product as colorless needles.

^{1}H NMR (400 MHz, DMSO-d_6) δ/ppm = 10.09 (br s, 1H), 3.10 (s, 6H).

^{13}C NMR (101 MHz, DMSO-d_6) δ/ppm = 42.8.

IR (ATR, cm^{-1}): 3248 (w), 3028 (w), 2942 (w), 1344 (m), 1139 (s), 969 (m), 877 (m), 764 (m).

HRMS (ESI): m/z = 171.9745 $[M-H]^-$ (calc. for $C_2H_6NO_4S_2^-$ m/z = 171.9744).

Bis(trifluoromethanesulfonyl)imide

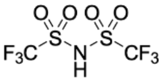

Tf_2NH

Tf_2NH was synthesized according to literature known procedures.[2] 2.15 g (7.49 mmol) $LiNTf_2$ was dissolved in 13 mL sulfuric acid and stirred at room temperature under a nitrogen stream for 30 min. Then, the flask was equipped with a Uglass tube and connected to a Schlenk tube. After bulb-to-bulb sublimation (100 °C oil bath temperature, 0.5 mbar), 1.65 g (5.87 mmol, 78%) of a colorless solid was obtained. Note: The product is hygroscopic and we recommend storage under nitrogen or argon.

^{1}H NMR (400 MHz, $CDCl_3$) δ/ppm = 6.41 (br s, 1H).

^{19}F NMR (377 MHz, $CDCl_3$) δ/ppm = –75.1.

2-Methyl-2-phenyloxirane

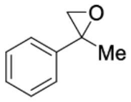

1a

Obtained as colorless liquid according to GP1. Analytical data are in accordance with those reported in the literature.[1]

Yield: 1.15 g (8.59 mmol, 86%).

***R*f** (*n*-hexane/ethyl acetate 40:1) = 0.24

^{1}H NMR (400 MHz, $CDCl_3$) δ/ppm = 7.31 – 7.23 (m, 4H), 7.22 – 7.18 (m, 1H), 2.90 (d, *J* = 5.4 Hz, 1H), 2.72 (dq, *J* = 5.3, 0.8 Hz, 1H), 1.65 (d, *J* = 0.8 Hz, 3H).

^{13}C NMR (101 MHz, $CDCl_3$) δ/ppm = 141.3, 128.5, 127.6, 125.2, 57.2, 56.9, 22.0.

HRMS (ESI): m/z = 157.0625 $[M+Na]^+$ (calc. for $C_9H_{10}ONa^+$ m/z = 157.0624).

2-Ethyl-2-phenyloxirane

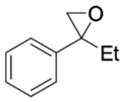

1b

Obtained as colorless liquid according to GP1. Analytical data are in accordance with those reported in the literature.[1]

Yield: 1.25 g (8.40 mmol, 84%).

***R*f** (*n*-hexane/ethyl acetate 5:1) = 0.46

^{1}H NMR (400 MHz, CD_2Cl_2) δ/ppm = 7.39 – 7.31 (m, 4H), 7.30 – 7.24 (m, 1H), 2.95 (d, *J* = 5.4 Hz, 1H), 2.70 (d, *J* = 5.3 Hz, 1H), 2.17 (dq, *J* = 14.8, 7.5 Hz, 1H), 1.80 (dq, *J* = 14.8, 7.5 Hz, 1H), 0.91 (dd, *J* = 7.5, 7.5 Hz, 3H).

^{13}C NMR (101 MHz, CD_2Cl_2) δ/ppm = 140.9, 128.8, 127.8, 126.6, 61.2, 55.7, 28.8, 9.3.

HRMS (ESI): m/z = 171.0778 $[M+Na]^+$ (calc. for $C_{10}H_{12}ONa^+$ m/z = 171.0780).

2-Phenyl-2-*n*-propyloxirane

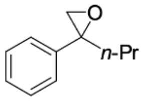

1c

Obtained as colorless liquid according to GP1. Analytical data are in accordance with those reported in the literature.[3]

Yield: 1.33 g (8.22 mmol, 82%).

***R*f** (*n*-hexane/ethyl acetate 5:1) = 0.44

^{1}H NMR (400 MHz, CD_2Cl_2) δ/ppm = 7.39 – 7.30 (m, 4H), 7.30 – 7.23 (m, 1H), 2.92 (d, *J* = 5.4 Hz, 1H), 2.69 (d, *J* = 5.5 Hz, 1H), 2.18 – 2.08 (m, 1H), 1.76 – 1.66 (m, 1H), 1.42 – 1.30 (m, 2H), 0.91 (t, *J* = 7.4 Hz, 3H).

^{13}C NMR (101 MHz, CD_2Cl_2) δ/ppm = 140.9, 128.6, 127.6, 126.4, 60.5, 55.6, 37.9, 18.7, 14.3.

HRMS (ESI): m/z = 185.0936 $[M+Na]^+$ (calc. for $C_{11}H_{14}ONa^+$ m/z = 185.0937).

2-Cyclohexyl-2-phenyloxirane

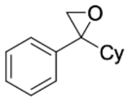

1d

Obtained as colorless solid according to GP1. Analytical data are in accordance with those reported in the literature.[1]

Yield: 1.70 g (8.39 mmol, 84%).

***R*f** (*n*-hexane/ethyl acetate 40:1) = 0.21

^{1}H NMR (400 MHz, CD_2Cl_2) δ/ppm = 7.36 – 7.24 (m, 5H), 2.98 (d, *J* = 5.2 Hz, 1H), 2.63 (d, *J* = 5.2 Hz, 1H), 1.81 – 1.68 (m, 5H), 1.66 – 1.58 (m, 1H), 1.29 – 1.16 (m, 2H), 1.12 – 0.92 (m, 3H).

^{13}C NMR (101 MHz, CD_2Cl_2) δ/ppm = 140.4, 128.2, 127.7, 127.6, 64.3, 53.0, 43.3, 29.2, 28.6, 26.7, 26.6, 26.5.

HRMS (APCI): m/z = 203.1431 $[M+H]^+$ (calc. for $C_{14}H_{19}O$ m/z = 203.1431).

2-(4-Methylphenyl)-2-methyloxirane

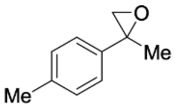

1e

Obtained as colorless oil according to GP1. Analytical data are in accordance with those reported in the literature.[4]

Yield: 1.23 g (8.31 mmol, 83%).

***R*f** (*n*-hexane/ethyl acetate 40:1) = 0.21

^{1}H NMR (400 MHz, CD_2Cl_2) δ/ppm = 7.26 – 7.21 (m, 2H), 7.17 – 7.12 (m, 2H), 2.92 (d, *J* = 5.5 Hz, 1H), 2.75 (dq, *J* = 5.4, 0.8 Hz, 1H), 2.33 (s, 3H), 1.67 (d, *J* = 0.8 Hz, 3H).

^{13}C NMR (101 MHz, CD_2Cl_2) δ/ppm = 138.8, 137.6, 129.3, 125.6, 57.2, 56.8, 22.0, 21.1.

HRMS (APCI): m/z = 149.0957 $[M+H]^+$ (calc. for $C_{10}H_{13}O^+$ m/z = 149.0961).

2-(3-Methylphenyl)-2-methyloxirane

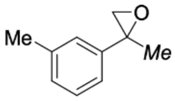

1f

Obtained as colorless oil according to GP1. Analytical data are in accordance with those reported in the literature.[5]

Yield: 1.21 g (8.19 mmol, 82%).

***R*f** (*n*-hexane/ethyl acetate 40:1) = 0.19

^{1}H NMR (400 MHz, CD_2Cl_2) δ/ppm = 7.25 – 7.19 (m, 1H), 7.19 – 7.12 (m, 2H), 7.11 – 7.06 (m, 1H), 2.92 (d, J = 5.5 Hz, 1H), 2.75 (dq, J = 5.5, 0.8 Hz, 1H), 2.34 (s, 3H), 1.67 (d, J = 0.8 Hz, 3H).

^{13}C NMR (101 MHz, CD_2Cl_2) δ/ppm = 141.8, 138.5, 128.5, 128.5, 126.3, 122.8, 57.2, 56.9, 22.1, 21.6.

HRMS (APCI): m/z = 149.0963 $[M+H]^+$ (calc. for $C_{10}H_{13}O^+$ m/z = 149.0961).

2-(2-Methylphenyl)-2-methyloxirane

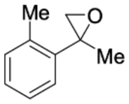

1g

Obtained as colorless oil according to GP1. Analytical data are in accordance with those reported in the literature.[6]

Yield: 1.18 g (7.98 mmol, 80%).

***R*f** (*n*-hexane/ethyl acetate 40:1) = 0.22

^{1}H NMR (400 MHz, CD_2Cl_2) δ/ppm = 7.36 – 7.29 (m, 1H), 7.22 – 7.10 (m, 3H), 2.93 (d, *J* = 5.3 Hz, 1H), 2.77 (dq, *J* = 5.4, 0.8 Hz, 1H), 2.40 (s, 3H), 1.56 (d, *J* = 0.7 Hz, 3H).

^{13}C NMR (101 MHz, CD_2Cl_2) δ/ppm = 140.5, 135.7, 130.4, 127.8, 127.2, 126.1, 58.4, 54.9, 23.7, 19.2.

HRMS (APCI): m/z = 149.0958 $[M+H]^+$ (calc. for $C_{10}H_{13}O^+$ m/z = 149.0961).

2-(4-Methoxyphenyl)-2-methyloxirane

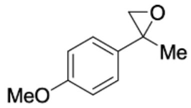

1h

Obtained as colorless oil according to GP1 and distillation at 65 °C at 8 mbar. Analytical data are in accordance with those reported in the literature.[6]

Yield: 1.45 g (8.15 mmol, 81%).

***R*f** (*n*-hexane/ethyl acetate 5:1) = 0.26

^{1}H NMR (400 MHz, C_6D_6) δ/ppm = 7.23 (dd, *J* = 8.8, 3.0 Hz, 2H), 6.76 (ddd, *J* = 184.3, 8.8, 3.0 Hz, 2H), 3.29 (s, 3H), 2.53 (d, *J* = 5.6 Hz, 1H), 2.47 (d, *J* = 5.6 Hz, 1H), 1.44 (s, 3H).

^{13}C NMR (101 MHz, C_6D_6) δ/ppm = 159.6, 134.1, 126.9, 114.1, 56.7, 56.2, 54.8, 22.0.

HRMS (ESI): m/z = 187.0733 $[M+Na]^+$ (calc. for $C_{10}H_{12}O_2Na^+$ m/z = 187.0730).

2-(4-Methylphenyl)-2-phenyloxirane

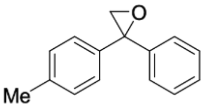

1i

Obtained as colorless oil according to GP1. Analytical data are in accordance with those reported in the literature.[7]

Yield: 1.77 g (8.42 mmol, 84%).

***R*f** (*n*-hexane/ethyl acetate 20:1) = 0.16

^{1}H NMR (400 MHz, $CDCl_3$) δ/ppm = 7.35 – 7.30 (m, 5H), 7.24 – 7.20 (m, 2H), 7.18 – 7.14 (m, 2H), 3.25 (d, *J* = 5.5 Hz, 1H), 3.22 (d, *J* = 5.5 Hz, 1H), 2.35 (s, 3H).

^{13}C NMR (101 MHz, $CDCl_3$) δ/ppm = 140.5, 138.2, 137.2, 129.3, 128.6, 128.2, 127.8, 127.8, 61.9, 57.0, 21.3.

HRMS (APCI): m/z = 211.1119 $[M+H]^+$ (calc. for $C_{15}H_{15}O^+$ m/z = 211.1118).

2-(4-Fluorophenyl)-2-methyloxirane

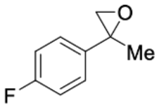

1j

Obtained as colorless oil according to GP1. Analytical data are in accordance with those reported in the literature.[8]

Yield: 1.40 g (9.17 mmol, 92%).

***R*f** (*n*-hexane/ethyl acetate 40:1) = 0.17

^{1}H NMR (400 MHz, $CDCl_3$) δ/ppm = 7.37 – 7.30 (m, 2H), 7.07 – 6.99 (m, 2H), 2.94 (d, *J* = 5.3 Hz, 1H), 2.74 (dq, *J* = 5.3, 0.7 Hz, 1H), 1.67 (d, *J* = 0.8 Hz, 3H).

^{13}C NMR (101 MHz, $CDCl_3$) δ/ppm = 162.5 (d, *J* = 245.2 Hz), 137.8 (d, *J* = 3.2 Hz), 127.5 (d, *J* = 8.2 Hz), 115.4 (d, *J* = 21.3 Hz), 57.2, 56.5, 22.0.

^{19}F NMR (377 MHz, $CDCl_3$) δ/ppm = –116.1

HRMS (APCI): m/z = 153.0707 $[M+H]^+$ (calc. for $C_9H_{10}FO$ m/z = 153.0710).

2-(4-Chlorophenyl)-2-methyloxirane

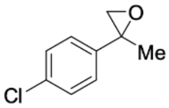

1k

Obtained as colorless oil according to GP1. Analytical data are in accordance with those reported in the literature.[8]

Yield: 1.55 g (9.19 mmol, 92%).

$\boldsymbol{R_f}$ (*n*-hexane/ethyl acetate 20:1) = 0.17

^{1}H NMR (400 MHz, $CDCl_3$) δ/ppm = 7.33 – 7.28 (m, 4H), 2.95 (d, *J* = 5.3 Hz, 1H), 2.73 (dq, *J* = 5.3, 0.7 Hz, 1H), 1.67 (d, *J* = 0.7 Hz, 3H).

^{13}C NMR (101 MHz, $CDCl_3$) δ/ppm = 140.2, 133.0, 128.4, 126.9, 56.9, 56.0, 21.4.

HRMS (ESI): m/z = 191.0237 $[M+Na]^+$ (calc. for $C_9H_9ONaCl^+$ m/z = 191.0234).

2-(4-Bromophenyl)-2-methyloxirane

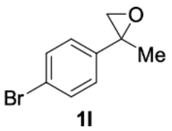

1l

Obtained as colorless oil according to GP1. Analytical data are in accordance with those reported in the literature.[8]

Yield: 1.96 g (9.21 mmol, 92%).

$\boldsymbol{R_f}$ (*n*-hexane/ethyl acetate 20:1) = 0.18

^{1}H NMR (400 MHz, CD_2Cl_2) δ/ppm = 7.51 – 7.42 (m, 2H), 7.28 – 7.20 (m, 2H), 2.95 (d, *J* = 5.3 Hz, 1H), 2.73 (dq, *J* = 5.4, 0.7 Hz, 1H), 1.67 (d, *J* = 0.8 Hz, 3H).

^{13}C NMR (101 MHz, CD_2Cl_2) δ/ppm = 141.1, 131.7, 127.6, 121.6, 57.3, 56.5, 21.7.

HRMS (APCI): m/z = 212.9912 $[M+H]^+$ (calc. for $C_9H_{10}OBr^+$ m/z = 212.9910).

2-(4-Iodophenyl)-2-methyloxirane

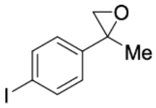

1m

Obtained as colorless oil according to GP1. Analytical data are in accordance with those reported in the literature.[8]

Yield: 1.66 g (6.36 mmol, 64%).

$\boldsymbol{R_f}$ (*n*-hexane/ethyl acetate 40:1) = 0.14

^{1}H NMR (400 MHz, CD_2Cl_2) δ/ppm = 7.70 – 7.64 (m, 2H), 7.15 – 7.08 (m, 2H), 2.94 (d, *J* = 5.4 Hz, 1H), 2.72 (dq, *J* = 5.4, 0.8 Hz, 1H), 1.66 (d, *J* = 0.7 Hz, 3H).

^{13}C NMR (101 MHz, CD_2Cl_2) δ/ppm = 141.8, 137.7, 127.8, 93.1, 57.3, 56.5, 21.6.

HRMS (APCI): m/z = 260.9773 $[M+H]^+$ (calc. for $C_9H_{10}OI^+$ m/z = 260.9771).

2-(4-Trifluoromethylphenyl)-2-methyloxirane

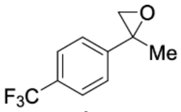

1n

Obtained as colorless oil according to GP1. Analytical data are in accordance with those reported in the literature.[9]

Yield: 1.72 g (8.51 mmol, 85%).

$\boldsymbol{R_f}$ (*n*-hexane/ethyl acetate 20:1) = 0.14

^{1}H NMR (400 MHz, CD_2Cl_2) δ/ppm = 7.63 – 7.58 (m, 2H), 7.52 – 7.47 (m, 2H), 2.99 (d, *J* = 5.3 Hz, 1H), 2.75 (dq, *J* = 5.4, 0.8 Hz, 1H), 1.72 (d, *J* = 0.7 Hz, 3H).

^{13}C NMR (101 MHz, CD_2Cl_2) δ/ppm = 146.1 (q, *J* = 1.3 Hz), 129.8 (q, *J* = 32.2 Hz), 126.2, 125.6 (q, *J* = 3.9 Hz), 124.7 (q, *J* = 271.7 Hz), 57.4, 56.6, 21.6.

^{19}F NMR (377 MHz, CD_2Cl_2) δ/ppm = -62.84.

HRMS (ESI): 203.0681 $[M+H]^+$ (calc. for $C_{10}H_{10}F_3O^+$ m/z = 203.0678).

2-(4-Cyanophenyl)-2-methyloxirane

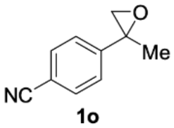

1o

Obtained as colorless oil according to GP1. Analytical data are in accordance with those reported in the literature.[8]

Yield: 1.52 g (9.54 mmol, 95%).

***R*f** (*n*-hexane/ethyl acetate 5:1) = 0.25

^{1}H NMR (400 MHz, CD_2Cl_2) δ/ppm = 7.69 – 7.60 (m, 2H), 7.52 – 7.45 (m, 2H), 3.00 (d, *J* = 5.3 Hz, 1H), 2.74 (dq, *J* = 5.3, 0.8 Hz, 1H), 1.71 (d, *J* = 0.8 Hz, 3H).

^{13}C NMR (101 MHz, CD_2Cl_2) δ/ppm = 147.3, 132.6, 126.5, 119.1, 111.7, 57.5, 56.5, 21.4.

HRMS (APCI): m/z = 160.0754 $[M+H]^+$ (calc. for $C_{10}H_{10}NO^+$ m/z = 160.0757).

2-(4-Nitrophenyl)-2-methyloxirane

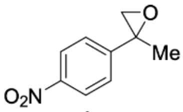

1p

Obtained as colorless oil according to GP1. Analytical data are in accordance with those reported in the literature.[4]

Yield: 1.41 g (8.88 mmol, 89%).

***R*f** (*n*-hexane/ethyl acetate 4:1) = 0.25

^{1}H NMR (400 MHz, $CDCl_3$) δ/ppm = 8.19 – 8.17 (m, 2H), 7.55 – 7.52 (m, 2H), 3.03 (d, *J* = 5.3 Hz, 1H), 2.77 (dq, *J* = 5.3, 0.7 Hz, 1H), 1.74 (d, *J* = 0.7 Hz, 3H).

^{13}C NMR (101 MHz, $CDCl_3$) δ/ppm = 148.9, 147.3, 126.3, 123.5, 57.1, 56.1, 21.0.

HRMS (ESI): m/z = 202.0479 $[M+Na]^+$ (calc. for $C_9H_9NO_3Na^+$ m/z = 202.0475).

2-(4-Methylbenzoate)-2-methyloxirane

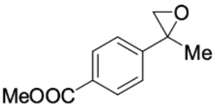

1q

Obtained as colorless solid according to GP1. Analytical data are in accordance with those reported in the literature.[8]

Yield: 1.61 g (9.01 mmol, 90%).

***R*f** (*n*-hexane/ethyl acetate 5:1) = 0.19

^{1}H NMR (400 MHz, CD_2Cl_2) δ/ppm = 8.02 – 7.94 (m, 2H), 7.47 – 7.39 (m, 2H), 3.88 (s, 3H), 2.98 (d, *J* = 5.4 Hz, 1H), 2.76 (dq, *J* = 5.4, 0.8 Hz, 1H), 1.71 (d, *J* = 0.7 Hz, 3H).

^{13}C NMR (101 MHz, CD_2Cl_2) δ/ppm = 167.0, 147.0, 129.9, 125.8, 57.4, 56.7, 52.3, 21.6.

HRMS (ESI): m/z = 215.0681 $[M+Na]^+$ (calc. for $C_{11}H_{12}O_3Na^+$ m/z = 215.0678).

2-Methyl-2-(naphthalene-2-yl)oxirane

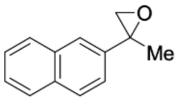

1r

Obtained as colorless oil according to GP1. Analytical data are in accordance with those reported in the literature.[8]

Yield: 1.53 g (8.32 mmol, 83%).

***R*f** (*n*-hexane/ethyl acetate 10:1) = 0.17

^{1}H NMR (400 MHz, CD_2Cl_2) δ/ppm = 7.89 – 7.80 (m, 4H), 7.53 – 7.41 (m, 3H), 3.03 (d, *J* = 5.3 Hz, 1H), 2.88 (dd, *J* = 5.4, 0.9 Hz, 1H), 1.80 (d, *J* = 0.8 Hz, 3H).

^{13}C NMR (101 MHz, CD_2Cl_2) δ/ppm = 139.3, 133.6, 133.1, 128.4, 128.3, 127.9, 126.6, 126.3, 124.8, 123.6, 57.3, 57.0, 22.0.

HRMS (APCI): m/z = 185.0956 $[M+Na]^+$ (calc. for $C_{13}H_{13}ON^+$ m/z = 185.0961).

2-Methyl-2-(naphthalene-1-yl)oxirane

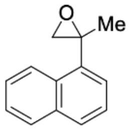

1s

Obtained as colorless oil according to GP1. Analytical data are in accordance with those reported in the literature.[10]

Yield: 1.50 g (8.13 mmol, 81%).

***R*$_f$** (*n*-hexane/ethyl acetate 20:1) = 0.16

^{1}H NMR (400 MHz, CD_2Cl_2) δ/ppm = 7.92 – 7.87 (m, 1H), 7.81 (dt, *J* = 8.3, 1.2 Hz, 1H), 7.61 – 7.42 (m, 4H), 3.12 (d, *J* = 5.3 Hz, 1H), 2.93 (dq, *J* = 5.4, 0.8 Hz, 1H), 1.77 (d, *J* = 0.7 Hz, 3H).

^{13}C NMR (101 MHz, CD_2Cl_2) δ/ppm = 138.5, 133.9, 131.0, 129.0, 128.3, 126.5, 126.1, 125.7, 124.7, 124.6, 58.3, 55.0, 24.6.

HRMS (ESI): m/z = 207.0781 $[M+Na]^+$ (calc. for $C_{13}H_{12}ONa^+$ m/z = 207.0780).

2-(4-*iso*-Butylphenyl)-2-methyloxirane

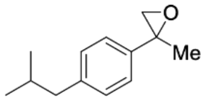

1t

Obtained as colorless oil according to GP1. Analytical data are in accordance with those reported in the literature.[11]

Yield: 1.851g (9.52 mmol, 95%).

***R*$_f$** (*n*-hexane/ethyl acetate 40:1) = 0.15

^{1}H NMR (400 MHz, CD_2Cl_2) δ/ppm = 7.26 – 7.24 (m, 2H), 7.13 – 7.11 (m, 2H), 2.93 (d, *J* = 5.4 Hz, 1H), 2.78 (dd, *J* = 5.4, 0.7 Hz, 1H), 2.46 (d, *J* = 7.2 Hz, 2H), 1.84 (pseudo sept, *J* = 6.7 Hz, 1H), 1.68 (d, *J* = 0.7 Hz, 3H), 0.89 (d, *J* = 6.5 Hz, 6H).

^{13}C NMR (101 MHz, CD_2Cl_2) δ/ppm = 141.0, 138.6, 128.9, 125.1, 56.9, 56.4, 44.9, 30.2, 22.0, 21.6.

HRMS (ESI): m/z = 213.1252 $[M+Na]^+$ (calc. for $C_{13}H_{18}ONa^+$ m/z = 213.1250).

2-Methyl-2-pentyloxirane

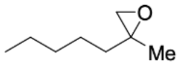

1u

Obtained as colorless oil according to GP1. Analytical data are in accordance with those reported in the literature.[12]

Yield: 0.93 g (7.2 mmol, 72%).

***R*$_f$** (*n*-hexane/ethyl acetate 40:1) = 0.21

^{1}H NMR (400 MHz, $CDCl_3$) δ/ppm = 2.62 – 2.55 (m, 2H), 1.64 – 1.24 (m, 11H), 0.89 (t, *J* = 6.9 Hz, 3H).

^{13}C NMR (101 MHz, $CDCl_3$) δ/ppm = 57.2, 54.1, 36.9, 32.0, 25.1, 22.7, 21.1, 14.1.

HRMS (APCI): m/z = 129.1271 $[M+H]^+$ (calc. for $C_8H_{17}O^+$ m/z = 129.1274).

2-Cyclohexyl-2-methyloxirane

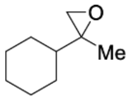

1v

Obtained as colorless oil according to GP1. Analytical data are in accordance with those reported in the literature.[8]

Yield: 1.04 g (7.43 mmol, 74%).

***R*f** (*n*-hexane/ethyl acetate 40:1) = 0.19

^{1}H NMR (400 MHz, $CDCl_3$) δ/ppm = 2.55 (dd, *J* = 5.0, 0.8 Hz, 1H), 2.47 (d, *J* = 5.0 Hz, 1H), 1.79 – 1.63 (m, 5H), 1.20 (d, *J* = 0.8 Hz, 4H), 1.27 – 1.02 (m, 6H).

^{13}C NMR (101 MHz, $CDCl_3$) δ/ppm = 59.9, 44.9, 29.3, 28.9, 27.2, 26.8, 26.7, 26.6, 18.2.

HRMS (APCI): m/z = 139.1118 $[M−H]^+$ (calc. for $C_9H_{15}O^+$ m/z = 139.1128).

3-(2-Methyl-2-oxiranyl)pyridine

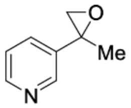

1w

Obtained as yellowish oil according to GP1. Analytical data are in accordance with those reported in the literature.[13]

Yield: 0.63 g (4.7 mmol, 47%).

***R*f** (*n*-hexane/ethyl acetate 3:1) = 0.14

^{1}H NMR (400 MHz, CD_2Cl_2) δ/ppm = 8.53 (ddd, *J* = 4.9, 1.8, 1.0 Hz, 1H), 7.66 (td, *J* = 7.8, 1.8 Hz, 1H), 7.30 (dt, *J* = 8.0, 1.1 Hz, 1H), 7.19 (ddd, *J* = 7.5, 4.9, 1.2 Hz, 1H), 2.98 (d, *J* = 5.5 Hz, 1H), 2.88 (dq, *J* = 5.5, 0.7 Hz, 1H), 1.76 (d, *J* = 0.7 Hz, 3H).

^{13}C NMR (101 MHz, CD_2Cl_2) δ/ppm = 160.7, 149.2, 137.0, 122.8, 119.1, 57.8, 57.0, 20.0.

HRMS (APCI): m/z = 158.0580 $[M+Na]^+$ (calc. for $C_8H_9NONa^+$ m/z = 158.0576).

2-Phenylpropionaldehyde

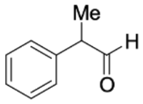

2a

Obtained as colorless oil according to GP2. Analytical data are in accordance with those reported in the literature.[8]

Yield: 261 g (1.95 mmol, 97%).

^{1}H NMR (400 MHz, $CDCl_3$) δ/ppm = 9.69 (d, *J* = 1.5 Hz, 1H), 7.41 – 7.36 (m, 2H), 7.34 – 7.28 (m, 1H), 7.24 – 7.20 (m, 2H), 3.64 (qd, *J* = 7.0, 1.4 Hz, 1H), 1.45 (d, *J* = 7.1 Hz, 3H).

^{13}C NMR (101 MHz, $CDCl_3$) δ/ppm = 201.3, 137.9, 129.2, 128.5, 127.7, 53.2, 14.8.

HRMS (APCI): m/z = 135.00807 $[M+H]^+$ (calc. for $C_9H_{11}O^+$ m/z = 135.0804).

2-Phenylbutyraldehyde

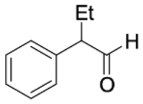

2b

Obtained as colorless oil according to GP2. Analytical data are in accordance with those reported in the literature.[8]

Yield: 288 mg (1.94 mmol, 97%).

^{1}H NMR (400 MHz, $CDCl_3$) δ/ppm = 9.68 (d, *J* = 2.1 Hz, 1H), 7.40 – 7.35 (m, 2H), 7.33 – 7.27 (m, 1H), 7.23 – 7.17 (m, 2H), 3.44 – 3.37 (m, 1H), 2.18 – 2.05 (m, 1H), 1.84 – 1.69 (m, 1H), 0.91 (t, *J* = 7.4 Hz, 3H).

^{13}C NMR (101 MHz, $CDCl_3$) δ/ppm = 201.2, 136.5, 129.2, 129.0, 127.7, 61.0, 23.1, 11.9.

HRMS (APCI): m/z = 149.0960 $[M+H]^+$ (calc. for $C_{10}H_{13}O^+$ m/z = 149.0961).

2-Phenylvaleraldehyde

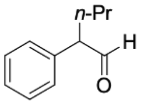

2c

Obtained as colorless oil according to GP2. Analytical data are in accordance with those reported in the literature.[3]

Yield: 311 mg (1.92 mmol, 96%).

^{1}H NMR (400 MHz, $CDCl_3$) δ/ppm = 9.67 (d, *J* = 2.1 Hz, 1H), 7.41 – 7.34 (m, 2H), 7.33 – 7.27 (m, 1H), 7.22 – 7.17 (m, 2H), 3.54 – 3.47 (m, 1H), 2.11 – 1.98 (m, 1H), 1.79 – 1.66 (m, 1H), 1.36 – 1.24 (m, 1H), 0.92 (t, *J* = 7.3 Hz, 3H).

^{13}C NMR (101 MHz, $CDCl_3$) δ/ppm = 201.2, 136.7, 129.2, 128.9, 127.6, 59.1, 31.9, 20.4, 14.1.

HRMS (APCI): m/z = 163.1119 $[M+H]^+$ (calc. for $C_{12}H_{15}O^+$ m/z = 163.1118).

2-Cyclohexyl-2-phenylacetaldehyde

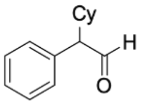

2d

Obtained as colorless solid according to GP2. Analytical data are in accordance with those reported in the literature.[8]

Yield: 381 mg (1.88 mmol, 94%).

^{1}H NMR (400 MHz, $CDCl_3$) δ/ppm = 9.70 (d, *J* = 3.5 Hz, 1H), 7.38 – 7.33 (m, 2H), 7.31 – 7.26 (m, 1H), 7.20 – 7.16 (m, 2H), 3.25 (dd, *J* = 9.6, 3.5 Hz, 1H), 2.11 (tdt, *J* = 11.3, 9.6, 3.4 Hz, 1H), 1.88 – 1.80 (m, 1H), 1.79 – 1.71 (m, 1H), 1.69 – 1.59 (m, 2H), 1.45 – 1.01 (m, 5H), 0.86 – 0.74 (m, 1H).

^{13}C NMR (101 MHz, $CDCl_3$) δ/ppm = δ 201.4, 135.4, 129.5, 129.0, 127.6, 66.0, 38.3, 32.0, 30.4, 26.4, 26.2, 26.2.

HRMS (APCI): m/z = 203.1431 $[M+H]^+$ (calc. for $C_{14}H_{19}O$ m/z = 203.1431).

2-(4-Methylphenyl)-propionaldehyde

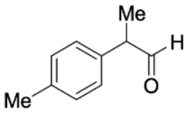

2e

Obtained as colorless liquid according to GP2. Analytical data are in accordance with those reported in the literature.[4]

Yield: 283 mg (1.91 mmol, 95%).

^{1}H NMR (400 MHz, $CDCl_3$) δ/ppm = 9.67 (d, *J* = 1.4 Hz, 1H), 7.22 – 7.17 (m, 2H), 7.12 – 7.08 (m, 2H), 3.60 (qd, *J* = 7.1, 1.4 Hz, 1H), 2.35 (s, 3H), 1.43 (d, *J* = 7.0 Hz, 3H).

^{19}C NMR (101 MHz, $CDCl_3$) δ/ppm = 201.4, 137.4, 134.8, 129.9, 128.3, 52.8, 21.2, 14.8.

HRMS (APCI): m/z = 149.0963 $[M+H]^+$ (calc. for $C_{10}H_{13}O^+$ m/z = 149.0961).

2-(3-Methylphenyl)-propionaldehyde

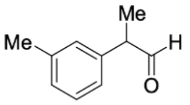

2f

Obtained as colorless oil according to GP2. Analytical data are in accordance with those reported in the literature.[14]

Yield: 283 mg (1.91 mmol, 95%).

^{1}H NMR (400 MHz, $CDCl_3$) δ/ppm = 9.68 (d, *J* = 1.4 Hz, 1H), 7.31 – 7.24 (m, 1H), 7.14 – 7.10 (m, 1H), 7.04 – 6.99 (m, 2H), 3.60 (qd, *J* = 7.1, 1.4 Hz, 1H), 2.36 (s, 3H), 1.43 (d, *J* = 7.1 Hz, 3H).

^{13}C NMR (101 MHz, $CDCl_3$) δ/ppm = 201.35, 138.97, 137.79, 129.22, 129.12, 128.43, 125.50, 53.13, 21.55, 14.75.

HRMS (APCI): m/z = 149.0963 $[M+H]^+$ (calc. for $C_{10}H_{13}O^+$ m/z = 149.0961).

2-(2-Methylphenyl)-propionaldehyde

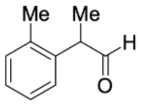

2g

Obtained as colorless oil according to GP2. Analytical data are in accordance with those reported in the literature.[8]

Yield: 285 mg (1.92 mmol, 96%)

^{1}H NMR (400 MHz, $CDCl_3$) δ/ppm = 9.67 (d, *J* = 1.2 Hz, 1H), 7.25 – 7.17 (m, 3H), 7.09 – 7.02 (m, 1H), 3.85 (qd, *J* = 7.0, 1.3 Hz, 1H), 2.37 (s, 3H), 1.41 (d, *J* = 7.0 Hz, 3H).

^{13}C NMR (101 MHz, $CDCl_3$) δ/ppm = 201.3, 136.8, 136.5, 131.1, 127.7, 127.6, 126.8, 49.4, 19.8, 14.5.

HRMS (APCI): m/z = 149.0957 $[M+H]^+$ (calc. for $C_{10}H_{13}O^+$ m/z = 149.0961).

2-(4-Methoxyphenyl)-propionaldehyde

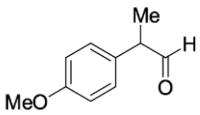

2h

Obtained as colorless oil according to GP2. Analytical data are in accordance with those reported in the literature.[8]

Yield: 343 mg (1.92 mmol, 96%)

^{1}H NMR (400 MHz, $CDCl_3$) δ/ppm = 9.65 (d, *J* = 1.5 Hz, 1H), 7.16 – 7.10 (m, 2H), 6.95 – 6.88 (m, 2H), 3.81 (s, 3H), 3.58 (qd, *J* = 7.1, 1.5 Hz, 1H), 1.42 (d, *J* = 7.1 Hz, 3H).

^{13}C NMR (101 MHz, $CDCl_3$) δ/ppm = 201.3, 159.2, 129.5, 114.7, 55.5, 52.3, 14.8.

HRMS (APCI): m/z = 165.0911 $[M+H]^+$ (calc. for $C_{10}H_{13}O_2^+$ m/z = 165.0910).

2-(4-Methylphenyl)-2-(phenyl)acetaldehyde

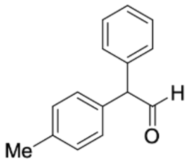

2i

Obtained as colorless solid according to GP2. Analytical data are in accordance with those reported in the literature.[15]

Yield: 400 mg (1.90 mmol, 95%).

^{1}H NMR (400 MHz, $CDCl_3$) δ/ppm = 9.94 (d, *J* = 2.5 Hz, 1H), 7.40 – 7.35 (m, 2H), 7.33 – 7.27 (m, 1H), 7.24 – 7.17 (m, 4H), 7.13 – 7.10 (m, 2H), 4.86 (d, *J* = 2.5 Hz, 1H), 2.35 (s, 3H).

^{13}C NMR (101 MHz, CD_2Cl_2) δ/ppm = 198.9, 137.5, 136.7, 133.3, 132.3, 130.5, 130.1, 129.9, 129.2, 129.2, 129.1, 128.4, 127.7, 63.9, 21.2.

HRMS (APCI): m/z = 211.1117 $[M+H]^+$ (calc. for $C_{15}H_{15}O^+$ m/z = 211.1118).

2-(4-Fluorophenyl)-propionaldehyde

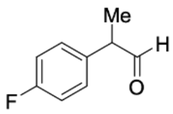

2j

Obtained as colorless liquid according to GP2. Analytical data are in accordance with those reported in the literature.[8]

Yield: 294 mg (1.93 mmol, 96%).

^{1}H NMR (400 MHz, $CDCl_3$) δ/ppm = 9.66 (d, *J* = 1.4 Hz, 1H), 7.21 – 7.14 (m, 2H), 7.11 – 7.03 (m, 2H), 3.63 (qd, *J* = 7.1, 1.4 Hz, 1H), 1.44 (d, *J* = 7.1 Hz, 3H).

^{13}**C NMR** (101 MHz, $CDCl_3$) δ/ppm = 200.9, 162.4 (d, *J* = 246.2 Hz), 133.5 (d, *J* = 3.1 Hz), 130.0 (d, *J* = 8.1 Hz), 116.1 (d, *J* = 21.2 Hz), 52.3, 14.9.

^{19}**F NMR** (377 MHz, $CDCl_3$) δ/ppm = -115.0

HRMS (APCI): m/z = 153.0711 $[M+H]^+$ (calc. for $C_9H_{10}FO$ m/z = 153.0710).

2-(4-Chlorophenyl)-propionaldehyde

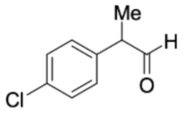

2k

Obtained as colorless oil according to GP2. Analytical data are in accordance with those reported in the literature.[8]

Yield: 320 mg (1.90 mmol, 95%).

^{1}**H NMR** (400 MHz, $CDCl_3$) δ/ppm = 9.66 (d, *J* = 1.4 Hz, 1H), 7.39 – 7.31 (m, 2H), 7.17 – 7.12 (m, 2H), 3.62 (qd, *J* = 7.1, 1.4 Hz, 1H), 1.43 (d, *J* = 7.1 Hz, 3H).

^{13}**C NMR** (101 MHz, $CDCl_3$) δ/ppm = 200.6, 136.3, 133.6, 129.8, 129.4, 52.4, 14.7.

HRMS (APCI): m/z = 169.0416 $[M+H]^+$ (calc. for $C_9H_{10}OCl^+$ m/z = 169.0415).

2-(4-Bromophenyl)-propionaldehyde

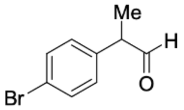

2l

Obtained as colorless oil according to GP2. Analytical data are in accordance with those reported in the literature.[8]

Yield: 409 mg (1.92 mmol, 96%).

^{1}**H NMR** (400 MHz, $CDCl_3$) δ/ppm = 9.66 (d, *J* = 1.4 Hz, 1H), 7.55 – 7.47 (m, 2H), 7.13 – 7.05 (m, 2H), 3.61 (qd, *J* = 7.1, 1.4 Hz, 1H), 1.44 (d, *J* = 7.1 Hz, 3H).

^{13}**C NMR** (101 MHz, $CDCl_3$) δ/ppm = 200.5, 136.8, 132.4, 130.1, 121.7, 52.5, 14.7.

HRMS (APCI): m/z = 212.9909 $[M+H]^+$ (calc. for $C_9H_{10}OBr^+$ m/z = 212.9910).

2-(4-Iodophenyl)-propionaldehyde

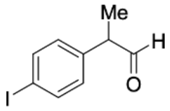

2m

Obtained as yellowish oil according to GP2. Analytical data are in accordance with those reported in the literature.[8]

Yield: 494 mg (1.90 mmol, 95%).

^{1}**H NMR** (400 MHz, $CDCl_3$) δ/ppm = 9.65 (d, *J* = 1.4 Hz, 1H), 7.75 – 7.67 (m, 2H), 7.01 – 6.90 (m, 2H), 3.59 (qd, *J* = 7.1, 1.4 Hz, 1H), 1.43 (d, *J* = 7.1 Hz, 3H).

^{13}**C NMR** (101 MHz, $CDCl_3$) δ/ppm = 200.5, 138.3, 137.5, 130.4, 93.2, 52.6, 14.7.

HRMS (APCI): m/z = 260.9773 $[M+H]^+$ (calc. for $C_9H_{10}OI^+$ m/z = 260.9771).

2-(4-Trifluoromethylphenyl)-propionaldehyde

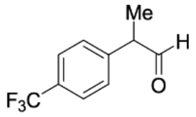

2n

Obtained as colorless liquid according to GP2. Analytical data are in accordance with those reported in the literature.[9]

Yield: 389 mg (1.92 mmol, 96%).

^{1}**H NMR** (400 MHz, $CDCl_3$) δ/ppm = 9.70 (d, *J* = 1.3 Hz, 1H), 7.64 (d, *J* = 8.1 Hz, 2H), 7.34 (d, *J* = 8.0 Hz, 2H), 3.76 – 3.68 (m, 1H), 1.49 (d, *J* = 7.1 Hz, 3H).

^{13}C NMR (101 MHz, $CDCl_3$) δ/ppm = 200.2, 141.9 (q *J* = 1.4 Hz), 130.1 (q, *J* = 32.8 Hz), 128.8, 126.1 (q, *J* = 3.7 Hz), 124.2 (q, *J* = 272.4 Hz) 52.9, 14.8.

^{19}F NMR (377 MHz, $CDCl_3$) δ/ppm = -62.6

HRMS (ESI): 203.0678 $[M+H]^+$ (calc. for $C_{10}H_{10}F_3O^+$ m/z = 203.0678).

2-(4-Cyanophenyl)-propionaldehyde

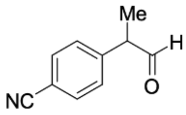

2o

Obtained as colorless oil according to GP2. Analytical data are in accordance with those reported in the literature.[8]

Yield: 310 mg (1.95 mmol, 97%).

^{1}H NMR (400 MHz, $CDCl_3$) δ/ppm = 9.69 (d, *J* = 1.2 Hz, 1H), 7.71 – 7.65 (m, 2H), 7.36 – 7.31 (m, 2H), 3.73 (qd, *J* = 7.2, 1.0 Hz, 1H), 1.49 (d, *J* = 7.1 Hz, 3H).

^{13}C NMR (101 MHz, $CDCl_3$) δ/ppm = 199.7, 143.3, 132.9, 129.3, 118.6, 111.8, 53.0, 14.7.

HRMS (APCI): m/z = 160.0753 $[M+H]^+$ (calc. for $C_{10}H_{10}NO^+$ m/z = 160.0757).

2-(4-Nitrophenyl)-propionaldehyde

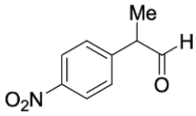

2p

Obtained as yellow oil according to GP2. Analytical data are in accordance with those reported in the literature.[4]

Yield: 348 mg (1.94 mmol, 97%).

^{1}H NMR (400 MHz, $CDCl_3$) δ/ppm = 9.72 (d, *J* = 1.2 Hz, 1H), 8.28 – 8.22 (m, 2H), 7.44 – 7.36 (m, 2H), 3.79 (qd, *J* = 7.2, 1.2 Hz, 1H), 1.52 (d, *J* = 7.1 Hz, 3H).

^{13}C NMR (101 MHz, $CDCl_3$) δ/ppm = 199.5, 147.6, 145.3, 129.4, 124.3, 52.8, 14.8.

HRMS (APCI): m/z = 180.0659 $[M+H]^+$ (calc. for $C_9H_{10}NO_3^+$ m/z = 180.0655).

2-(4-Methoxycarbonylphenyl)-propionaldehyde

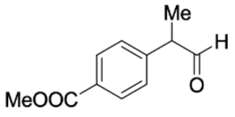

2q

Obtained as colorless oil according to GP2. Analytical data are in accordance with those reported in the literature.[8]

Yield: 369 mg (1.92 mmol, 96%).

^{1}H NMR (400 MHz, $CDCl_3$) δ/ppm = 9.70 (d, *J* = 1.3 Hz, 1H), 8.08 – 8.02 (m, 2H), 7.32 – 7.27 (m, 2H), 3.92 (s, 3H), 3.71 (qd, *J* = 7.1, 1.4 Hz, 1H), 1.47 (d, *J* = 7.1 Hz, 3H).

^{13}C NMR (101 MHz, $CDCl_3$) δ/ppm = 200.4, 166.9, 143.0, 130.5, 129.6, 128.5, 53.1, 52.3, 14.7.

HRMS (APCI): m/z = 193.0863 $[M+H]^+$ (calc. for $C_{11}H_{13}O_3^+$ m/z = 193.0859).

2-(Naphth-2-yl)propionaldehyde

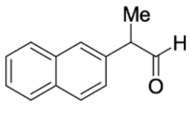

2r

Obtained as colorless solid according to GP2. Analytical data are in accordance with those reported in the literature.[8]

Yield: 329 mg (1.79 mmol, 89%).

^{1}H NMR (400 MHz, $CDCl_3$) δ/ppm = 9.77 (d, *J* = 1.4 Hz, 1H), 7.90 – 7.80 (m, 3H), 7.70 – 7.67 (m, 1H), 7.54 – 7.46 (m, 2H), 7.33 (dd, *J* = 8.5, 1.8 Hz, 1H), 3.81 (qd, *J* = 7.0, 1.4 Hz, 1H), 1.55 (d, *J* = 7.0 Hz, 3H).

^{13}C NMR (101 MHz, $CDCl_3$) δ/ppm = 201.1, 135.3, 133.8, 132.8, 129.0, 127.9, 127.3, 126.6, 126.3, 126.3, 53.3, 14.8.

HRMS (APCI): m/z = 185.0957 $[M+Na]^+$ (calc. for $C_{13}H_{13}ON^+$ m/z = 185.0961).

2-(Naphth-1-yl)propionaldehyde

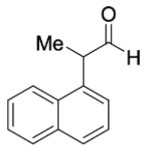

2s

Obtained as colorless oil according to GP2. Analytical data are in accordance with those reported in the literature.[14]

Yield: 339 mg (1.84 mmol, 92%).

^{1}H NMR (400 MHz, $CDCl_3$) δ/ppm = 9.77 (d, *J* = 1.4 Hz, 1H), 8.05 – 8.00 (m, 1H), 7.91 (dd, *J* = 8.0, 1.5 Hz, 1H), 7.83 (dt, *J* = 8.3, 1.1 Hz, 1H), 7.60 – 7.47 (m, 3H), 7.30 (dd, *J* = 7.1, 1.2 Hz, 1H), 4.39 (qd, *J* = 6.9, 1.3 Hz, 1H), 1.59 (d, *J* = 7.0 Hz, 3H).

^{13}C NMR (101 MHz, $CDCl_3$) δ/ppm = 201.4, 134.3, 134.3, 131.9, 129.3, 128.5, 126.8, 126.1, 125.8, 125.8, 123.1, 49.0, 14.8.

HRMS (APCI): m/z = 185.0961 $[M+Na]^+$ (calc. for $C_{13}H_{13}ON^+$ m/z = 185.0961).

2-(4-*iso*-Butylphenyl)-propionaldehyde

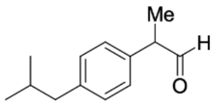

2t

Obtained as colorless oil according to GP2. Analytical data are in accordance with those reported in the literature.[11]

Yield: 370 mg (1,94 mmol, 97%).

^{1}H NMR (400 MHz, $CDCl_3$) δ/ppm = 9.67 (d, *J* = 1.5 Hz, 1H), 7.19 – 7.09 (m, 4H), 3.61 (qd, *J* = 7.0, 1.5 Hz, 1H), 2.47 (d, *J* = 7.2 Hz, 3H), 1.86 (pseudo sept, *J* = 6.8 Hz, 1H), 1.43 (d, *J* = 7.1 Hz, 3H).

^{13}C NMR (101 MHz, $CDCl_3$) δ/ppm = 201.4, 141.2, 135.0, 129.9, 128.2, 52.8, 45.2, 30.3, 22.5, 14.7.

HRMS (APCI): m/z = 191.1431 $[M+H]^+$ (calc. for $C_{13}H_{19}O^+$ m/z = 191.1431).

2-Methylheptanal

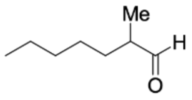

2u

Obtained as colorless oil according to GP2. Analytical data are in accordance with those reported in the literature.[13]

Yield: 233 mg (1.82 mmol, 91%).

^{1}H NMR (400 MHz, $CDCl_3$) δ/ppm = δ 9.61 (d, *J* = 2.0 Hz, 1H), 2.38 – 2.28 (m, 1H), 1.74 – 1.65 (m, 1H), 1.38 – 1.25 (m, 7H), 1.08 (d, *J* = 7.0 Hz, 3H), 0.93 – 0.84 (m, 3H).

^{13}C NMR (101 MHz, $CDCl_3$) δ/ppm = 205.6, 46.5, 32.0, 30.6, 26.8, 22.6, 14.1, 13.5.

HRMS (APCI): m/z = 129.1277 $[M+H]^+$ (calc. for $C_8H_{17}O^+$ m/z = 129.1274).

2-Cyclohexylpropionaldehyde

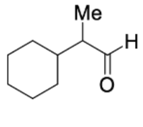

2v

Obtained as colorless oil according to GP2. Analytical data are in accordance with those reported in the literature.[8]

Yield: 267 mg (1.90 mmol, 95%).

^{1}**H NMR** (400 MHz, $CDCl_3$) δ/ppm = δ 9.65 (d, *J* = 2.3 Hz, 1H), 2.25 – 2.16 (m, 1H), 1.79 – 1.55 (m, 6H), 1.32 – 1.07 (m, 5H), 1.03 (d, *J* = 7.0 Hz, 3H).

^{13}**C NMR** (101 MHz, $CDCl_3$) δ/ppm = 206.1, 52.0, 38.7, 31.3, 29.3, 26.6, 26.5, 26.4, 10.2.

HRMS (APCI): m/z = 139.1121 $[M{-}H]^+$ (calc. for $C_9H_{15}O^+$ m/z = 139.1128).

2,5-Dimethyl-2,5-diphenyl-1,4-dioxane

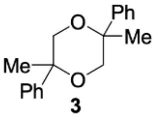

3

0.3 mg (1 µmol) Tf_2NH was dissolved in 1 mL HFIP and the reaction mixture was stirred at room temperature for 5 min. Afterwards, 27 mg (0.21 mmol) 2-methyl-2-phenyloxirane was added and the reaction mixture was stirred for 15 min. Then, 3 mL saturated $NaHCO_3$ solution were added and the aqueous phase was extracted with *n*-hexane (3 x 5 mL). The combined organic phases were washed with brine (2 x 5 mL), dried over $MgSO_4$, and the solvent was removed under reduced pressure. Purification by column chromatography (n-hexane/ethyl acetate 50:1 → 20:1) afforded 5.2 mg (0.019 mmol, 19%) of the syn-dimer and 3.1 mg (0.011 mmol, 11%) of the anti-dimer as colorless oils.

Note: We observe reproducibility issues for this reaction, probably due to sensitivity against humidity and small changes in the reaction conditions, such as reaction temperature and time.

Syn-2,5-Dimethyl-2,5-diphenyl-1,4-dioxane

Yield: 5.2 mg (0.019 mmol, 19%).

$\boldsymbol{R_f}$ (*n*-hexane/ethyl acetate 25:1): 0.17.

^{1}**H NMR** (400 MHz, $CDCl_3$) δ/ppm = 7.50 – 7.45 (m, 4H), 7.37 – 7.32 (m, 4H), 7.27 – 7.22 (m, 2H), 4.09 (d, *J* = 12.4 Hz, 2H), 3.51 (d, *J* = 12.4 Hz, 2H), 1.18 (s, 6H).

^{13}**C NMR** (101 MHz, $CDCl_3$) δ/ppm = 143.3, 128.6, 127.2, 126.4, 74.3, 67.2, 26.9.

HRMS (ESI): m/z = 291.1359 $[M+Na]^+$ (calc. for $C_{18}H_{20}O_2Na^+$ m/z = 291.1356).

Anti-2,5-Dimethyl-2,5-diphenyl-1,4-dioxane

Yield: 3.1 mg (0.011 mmol, 11%).

$\boldsymbol{R_f}$ (*n*-hexane/ethyl acetate 25:1): 0.15.

^{1}**H NMR** (400 MHz, $CDCl_3$) δ/ppm = 7.44 – 7.40 (m, 4H), 7.33 – 7.28 (m, 4H), 7.25 – 7.19 (m, 2H), 3.91 (d, *J* = 12.2 Hz, 2H), 3.79 (d, *J* = 12.2 Hz, 2H), 1.59 (s, 6H).

^{13}**C NMR** (101 MHz, $CDCl_3$) δ/ppm = 143.5, 128.3, 127.1, 125.8, 73.9, 67.8, 24.4.

HRMS (ESI): m/z = 291.1355 $[M+Na]^+$ (calc. for $C_{18}H_{20}O_2Na^+$ m/z = 291.1356).

2-Phenyl-1-propanol

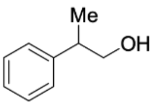

4a

2.8 mg (0.01 mmol) Tf_2NH was dissolved in 10 mL HFIP and the reaction mixture was heated to 55 °C. Afterwards, 269 mg (2.01 mmol, 1.0 eq) 2-methyl-2-phenyloxirane was added slowly and the reaction mixture was stirred for 15 min at 55 °C. The solution was cooled to 0 °C and 10 mL methanol and 150 mg (3.97 mmol, 2.0 eq) $NaBH_4$ were added. Afterwards, the reaction mixture was stirred for 2 h at room temperature. Then, the reaction mixture was quenched with 10 mL brine and extracted with CH_2Cl_2 (5 x 10 mL). The combined organic phases were washed with brine (2 x 5 mL), dried over $MgSO_4$, and the solvent was removed under reduced pressure. Purification by column chromatography (n-hexane/ethyl acetate 5:1 → 2:1) afforded 257 mg

(1.92 mmol, 96%) product as colorless oil. Analytical data are in accordance with those reported in the literature.[16]

Yield: 257 mg (1.92 mmol, 96%).

***R*f** (*n*-hexane/ethyl acetate 5:1) = 0.16

^{1}H NMR (400 MHz, CD_2Cl_2) δ/ppm = 7.35 – 7.30 (m, 1H), 7.26 – 7.20 (m, 2H), 3.72 – 3.62 (m, 1H), 2.92 (tt, *J* = 7.0, 7.0 Hz, 1H), 1.44 (br s, 1H), 1.26 (d, *J* = 7.1 Hz, 2H).

^{13}C NMR (101 MHz, CD_2Cl_2) δ/ppm = 144.6, 128.9, 127.9, 126.9, 68.9, 42.9, 17.9.

HRMS (ESI): m/z = 149.0610 [M−H]$^-$ (calc. for $C_9H_9O_2^-$ m/z = 149.0608).

N-Benzhydryl-2-phenylbutan-1-amine

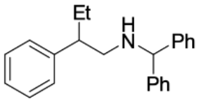

5b

2.8 mg (0.01 mmol) Tf_2NH was dissolved in 10 mL HFIP and the reaction mixture was heated to 55 °C. Afterwards, 297 mg (2.0 mmol, 10. eq) 2-ethyl-2-phenyloxirane was added slowly and the reaction mixture was stirred for 15 min at 55 °C. The solution was cooled to room temperature and 33 µl (28 mg, 0.4 mmol, 0.2 eq). pyrrolidine and 0.31 mL (0.33 mg, 2.4 mmol, 1.2 eq) benzhydrylamine were added to the reaction mixture. After 1 h stirring vigorously, 150 mg (3.97 mmol, 2.0 eq) $NaBH_4$ and 10 mL methanol were added. After further 2 h, 20 mL water were added to the mixture and then extracted with CH_2Cl_2 (5 x 10 mL). The combined organic phases were washed with brine (2 x 5 mL), dried over $MgSO_4$, and the solvent was removed under reduced pressure. Purification by column chromatography (n-hexane/ethyl acetate 5:1 → 2:1) afforded 528 mg (1.67 mmol, 84%) product as colorless oil. Analytical data are in accordance with those reported in the literature.[1]

Yield: 528 mg (1.67 mmol, 84%).

***R*f** (*n*-hexane/ethyl acetate 2:1): 0.30.

^{1}H NMR (400 MHz, $CDCl_3$) δ/ppm = 7.26 – 7.07 (m, 15H), 4.67 (s, 1H), 2.78 – 2.59 (m, 3H), 1.70 – 1.58 (m, 1H), 1.53 – 1.39 (m, 2H), 0.68 (t, *J* = 7.4 Hz, 3H).

^{13}C NMR (101 MHz, $CDCl_3$) δ/ppm = 144.3, 144.3, 143.9, 128.6, 128.5, 128.5, 128.1, 127.4, 127.4, 127.0, 127.0, 126.5, 67.5, 53.8, 48.3, 27.4, 12.2.

HRMS (ESI): m/z = 338.1878 [M+Na]$^+$ (calc. for $C_{22}H_{23}NNa^+$ m/z = 338.1879).

2-Phenylpropionic acid

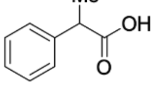

6a

Following GP2, crude 2-phenyl-propionaldehyde (2.0 mmol, 1.0 eq.) was dissolved in mixture of 3.3 mL *tert*-butanol and 2.2 mL distilled water. Then, 238 mg (2.63 mmol, 1.3 eq) $NaClO_2$ and 349 mg (2.56 mmol, 1.3 eq) KH_2PO_4 were added and the mixture was stirred over night at room temperature. Afterwards, 5 mL distilled water were added and the reaction mixture got extracted with CH_2Cl_2 (5 x 10 mL). The combined organic phases were washed with brine (2 x 5 mL), dried over $MgSO_4$, and the solvent was removed under reduced pressure. Purification by column chromatography (n-hexane/ethyl acetate 3:1 → 2:1) afforded 284 mg (1.89 mmol 95%) product as colorless oil. Analytical data are in accordance with those reported in the literature.[17]

Yield: 284 mg (1.89 mmol, 95%).

***R*f** (*n*-hexane/ethyl acetate 3:1): 0.13

^{1}**H NMR** (400 MHz, $CDCl_3$) δ/ppm = 9.32 (br s, 1H), 7.29 – 7.19 (m, 5H), 3.70 (q, *J* = 7.1 Hz, 1H), 1.46 (d, *J* = 7.2 Hz, 3H).

^{13}**C NMR** (101 MHz, $CDCl_3$) δ/ppm = 180.8, 139.8, 128.7, 127.6, 127.4, 45.4, 18.1.

HRMS (ESI): m/z = 149.0610 $[M-H]^-$ (calc. for $C_9H_9O_2^-$ m/z = 149.0608).

Ibuprofen

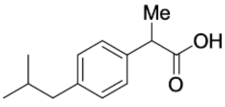

6t

5.05 g (22.9 mmol, 1.15 eq) trimethylsulfoxonium iodide was dissolved in 135 mL dry DMSO. Afterwards, 2.68 g (21.9 mmol, 1.1 eq.) KO*t*Bu was added and the solution was stirred for 45 min at room temperature. Then, 3.52 g (20.0 mmol (1.0 eq.) 4'-*iso*-butylacetophenone was added slowly *via* syringe and the reaction was stirred for 36 h at room temperature. The reaction mixture was diluted with 200 mL water and extracted with ethyl acetate (6 x 30 mL). The combined organic phases were washed with brine (3 x 30 mL), dried over Na_2SO_4 and concentrated under reduced pressure. Purification *via* filtration over silica gel with *n*-hexane/ethyl acetate 25:1 as eluent afforded crude 2-(4-*iso*-butylphenyl)-2-methyloxirane **1t**, which was used directly in the next step. 11.2 mg (0.040 mmol, 0.2 mol%) Tf_2NH was dissolved in 40 mL HFIP and heated to 55 °C. Afterwards, the crude 2-(4-*iso*-butylphenyl)-2-methyloxirane **1t** was dissolved in 10 mL HFIP added slowly *via* a dropping funnel and the reaction mixture was stirred for 45 min at 55 °C. Then, 20 mL saturated $NaHCO_3$ solution were added and the aqueous phase was extracted with *n*-hexane (5 x 15 mL). The combined organic phases were washed with brine (3 x 5 mL), dried over $MgSO_4$, and the solvent was removed under reduced pressure. Crude 2-(4-*iso*-Butylphenyl)-propionaldehyde was dissolved in mixture of 28 mL *tert*-butanol and 18 mL distilled water. Then, 2.17 g (24.0 mmol, 1.2 eq) $NaClO_2$ and 3.25 g (23.9 mmol, 1.2 eq) KH_2PO_4 were added and the mixture was stirred over night at room temperature. Afterwards, 50 mL distilled water were added and the reaction mixture was extracted with CH_2Cl_2 (5 x 30 mL). The combined organic phases were washed with brine (2 x 10 mL), dried over $MgSO_4$, and the solvent was removed under reduced pressure. Purification by column chromatography (n-hexane/ethyl acetate 3:1 → 2:1) afforded 3.72 g (18.0 mmol, 90% over three steps) product as colorless oil, which solidified after two days. Analytical data are in accordance with those reported in the literature.[11]

$\boldsymbol{R_f}$ (*n*-hexane/ethyl acetate 3:1): 0.14.

^{1}**H NMR** (400 MHz, $CDCl_3$) δ/ppm = 8.10 (br s, 1H), 7.22 (dt, *J* = 7.9, 1.8 Hz, 2H), 7.10 (dt, *J* = 7.9, 1.8 Hz 2H), 3.71 (q, *J* = 7.2 Hz, 1H), 2.45 (d, *J* = 7.2 Hz, 2H), 1.86 (sept, *J* = 6.7 Hz, 1H), 1.50 (d, *J* = 7.2 Hz, 3H), 0.90 (d, *J* = 6.7 Hz, 6H).

^{13}**C NMR** (101 MHz, $CDCl_3$) δ/ppm = 180.5, 140.9, 137.0, 129.4, 127.3, 45.1, 44.9, 30.2, 22.4, 18.1.

HRMS (ESI): m/z = 229.1200 $[M+Na]^+$ (calc. for $C_{13}H_{18}O_2Na^+$ m/z = 229.1199).

6. NMR Spectra

Bis(methanesulfonyl)imide

^{1}H-NMR (400 MHz, DMSO-d_6)

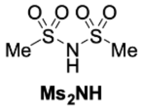

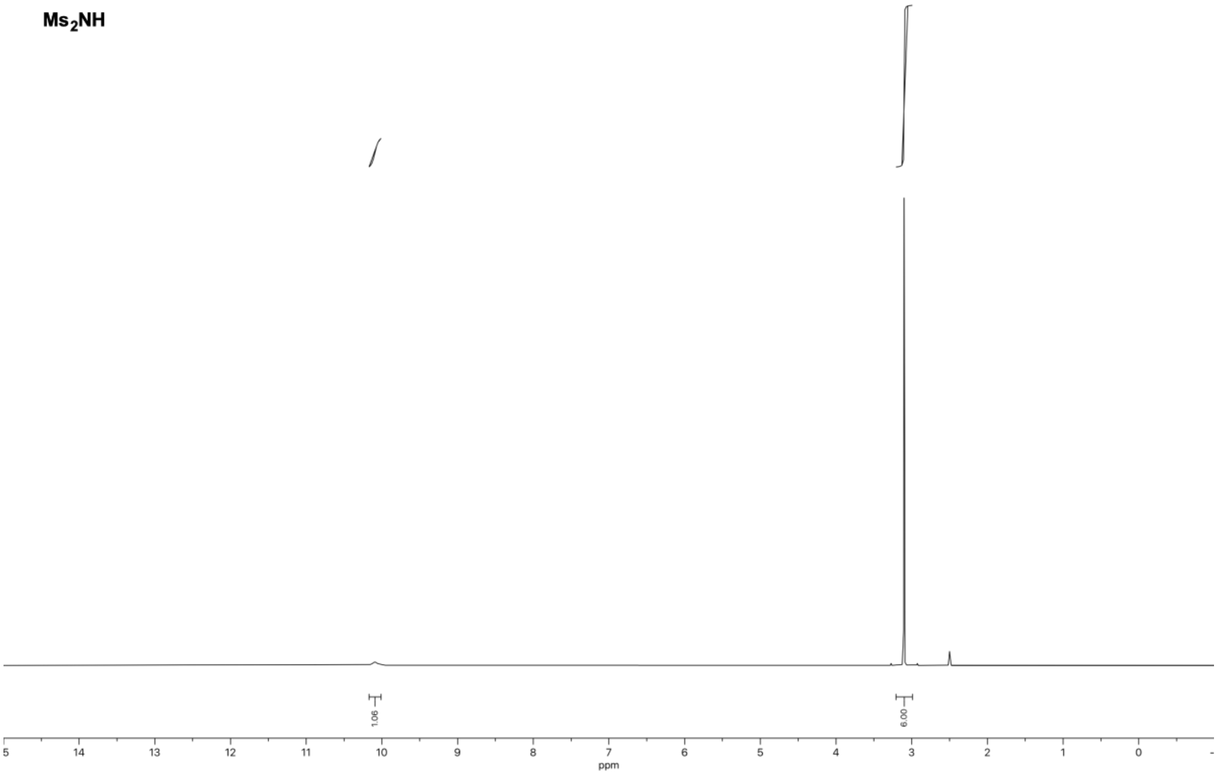

^{13}C-NMR (101 MHz, DMSO-d_6)

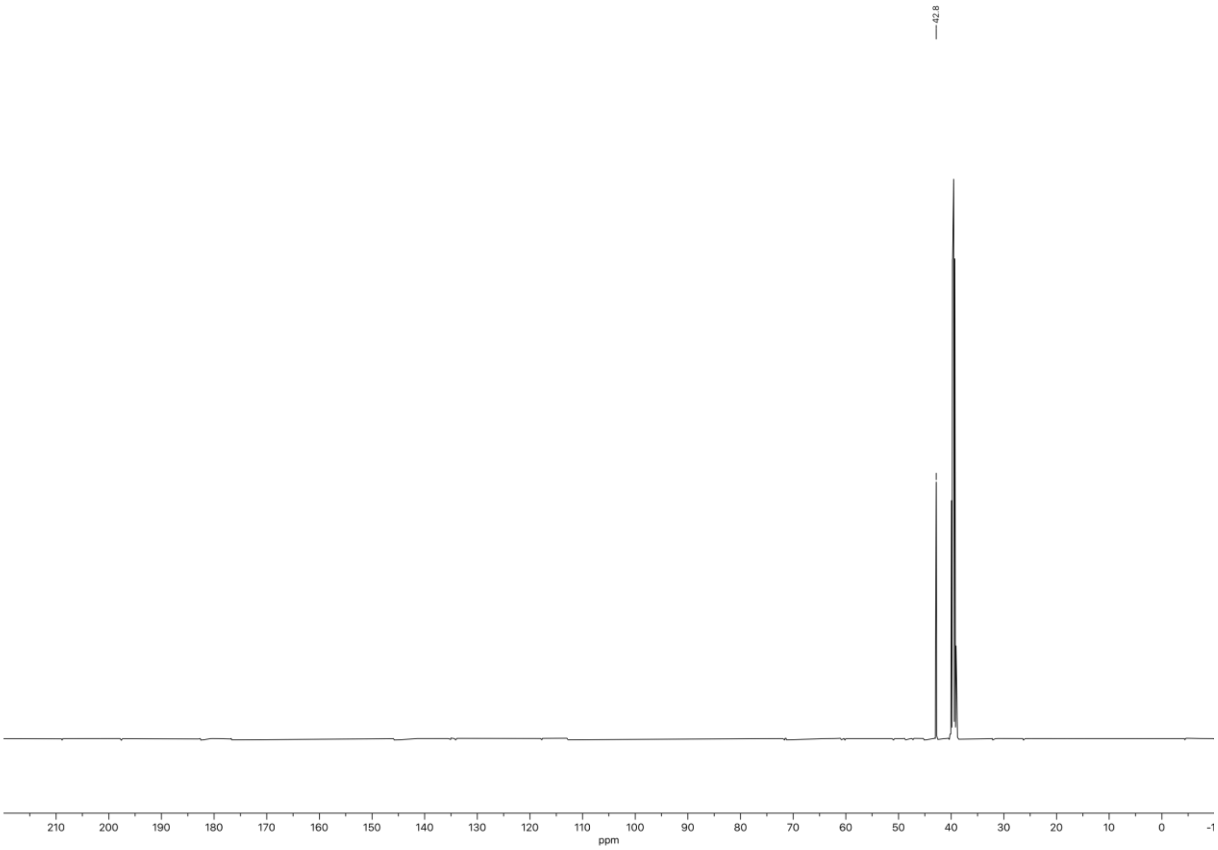

Bis(trifluoromethanesulfonyl)imide

^{1}H-NMR (400 MHz, $CDCl_3$)

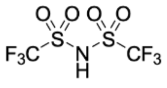

Tf$_2$NH

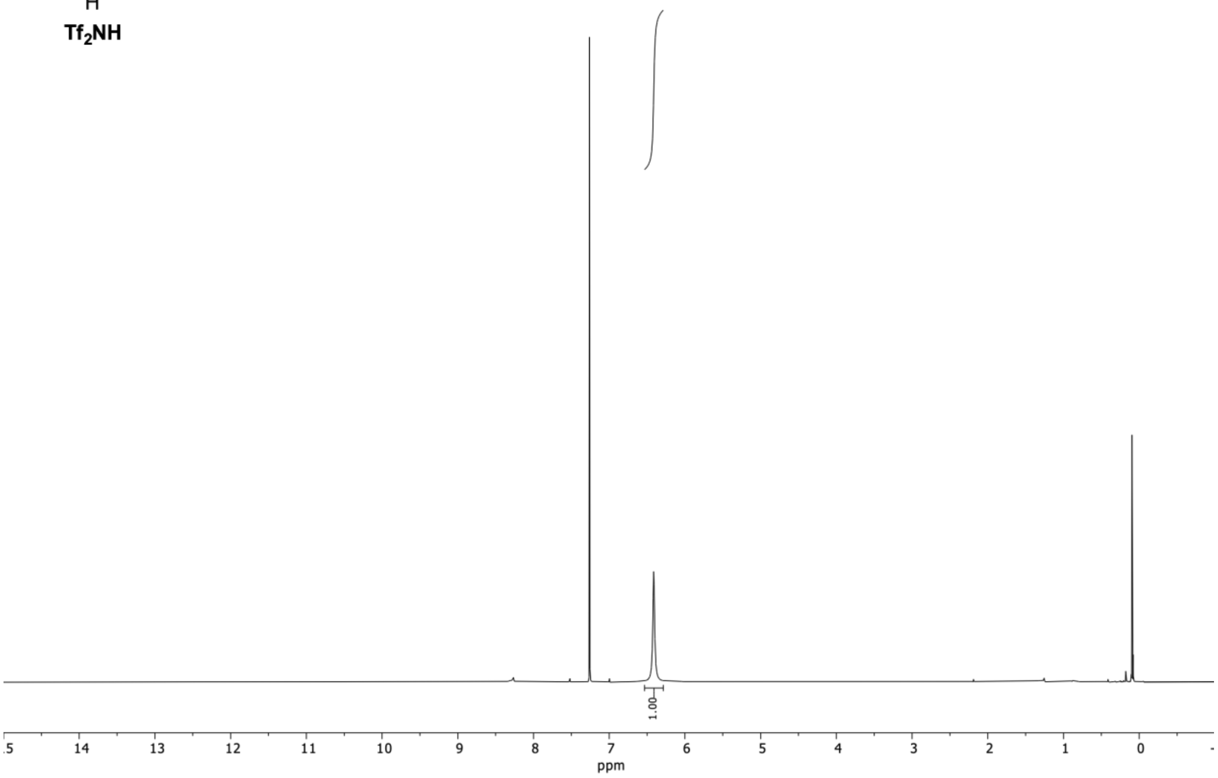

^{19}F-NMR (377 MHz, $CDCl_3$)

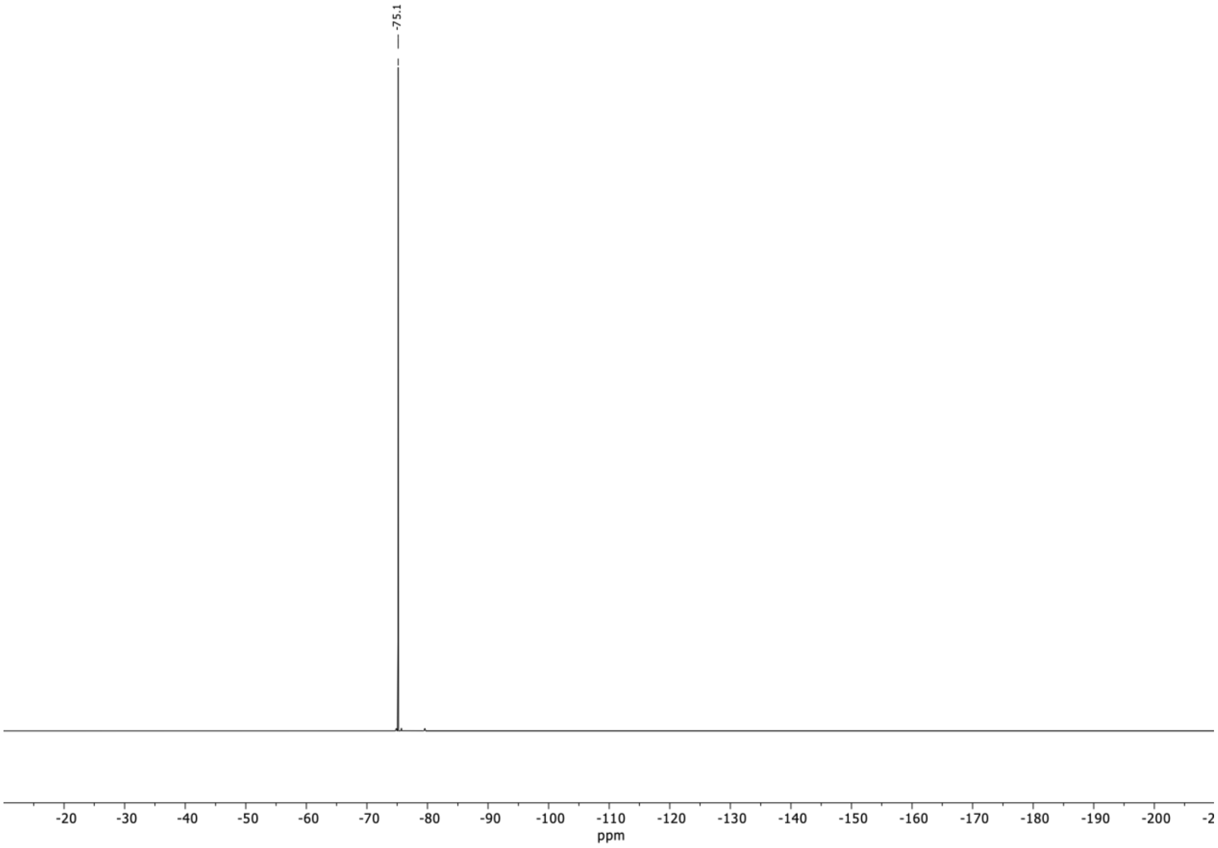

Bis(*para*-tosyl)imide

^{1}H-NMR (400 MHz, DMSO-d_6)

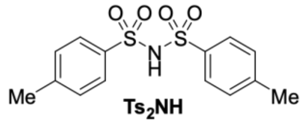

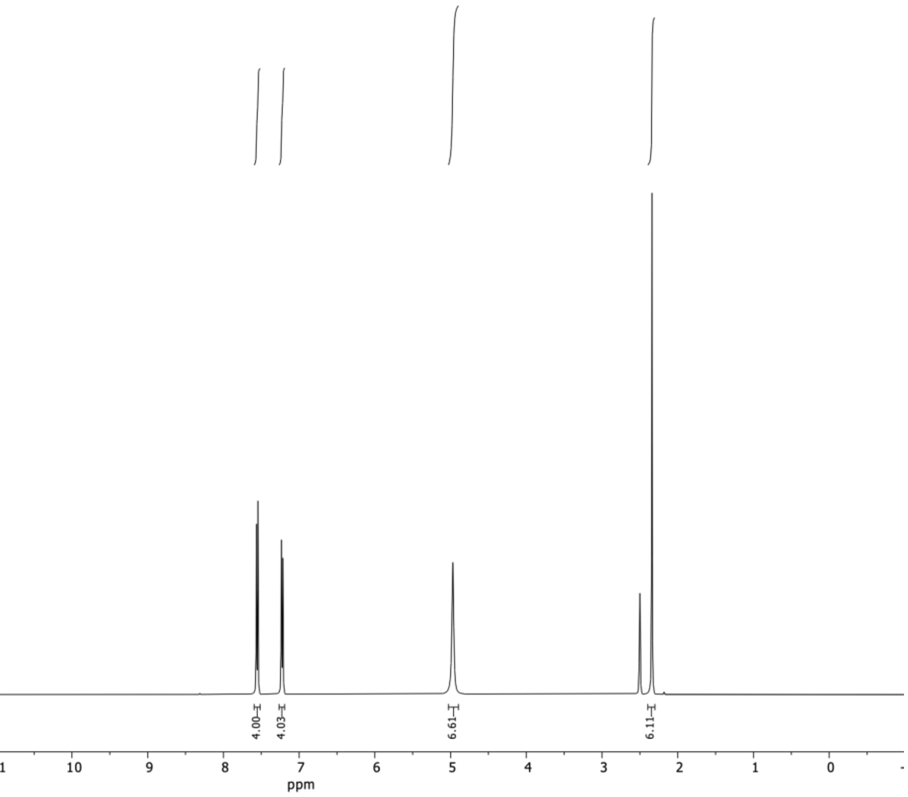

^{13}C-NMR (101 MHz, DMSO-d_6)

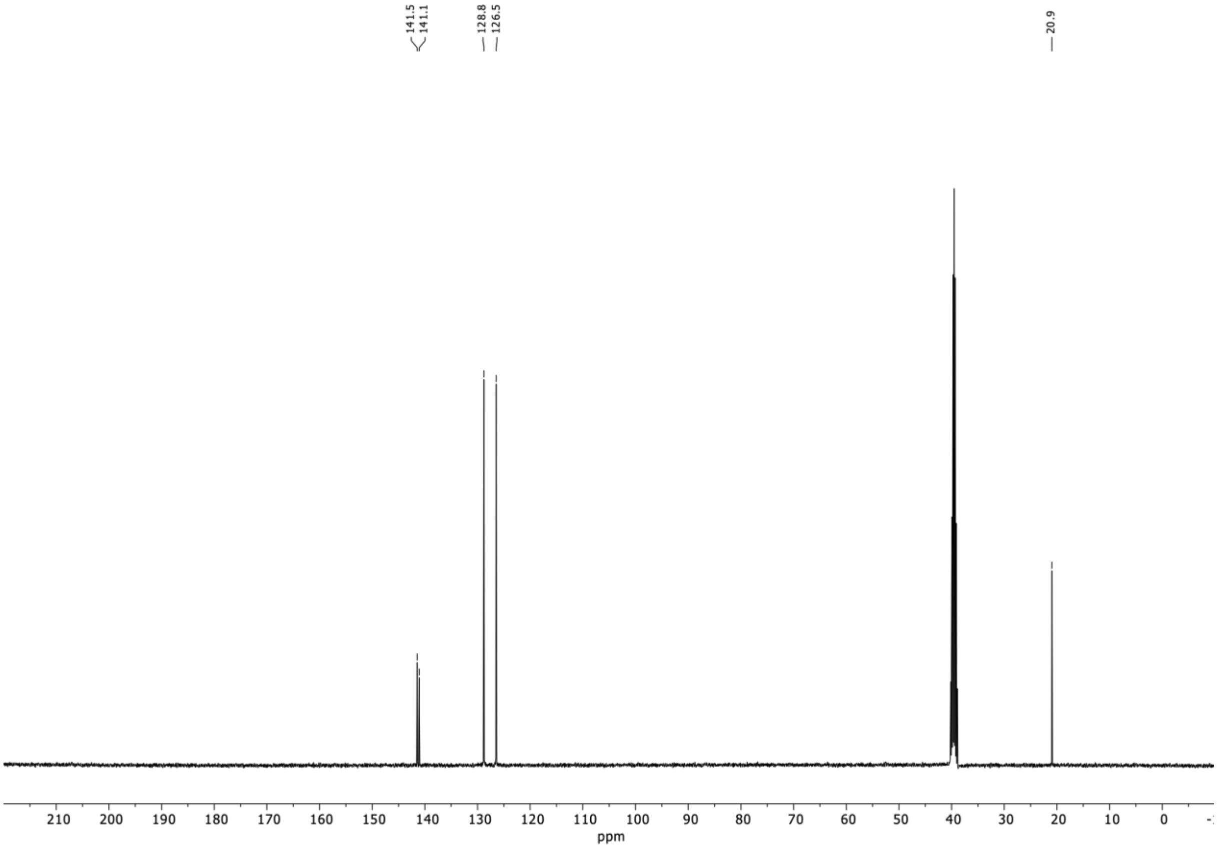

2-Methyl-2-phenyloxirane

^{1}H-NMR (400 MHz, CD_2Cl_2)

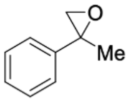

1a

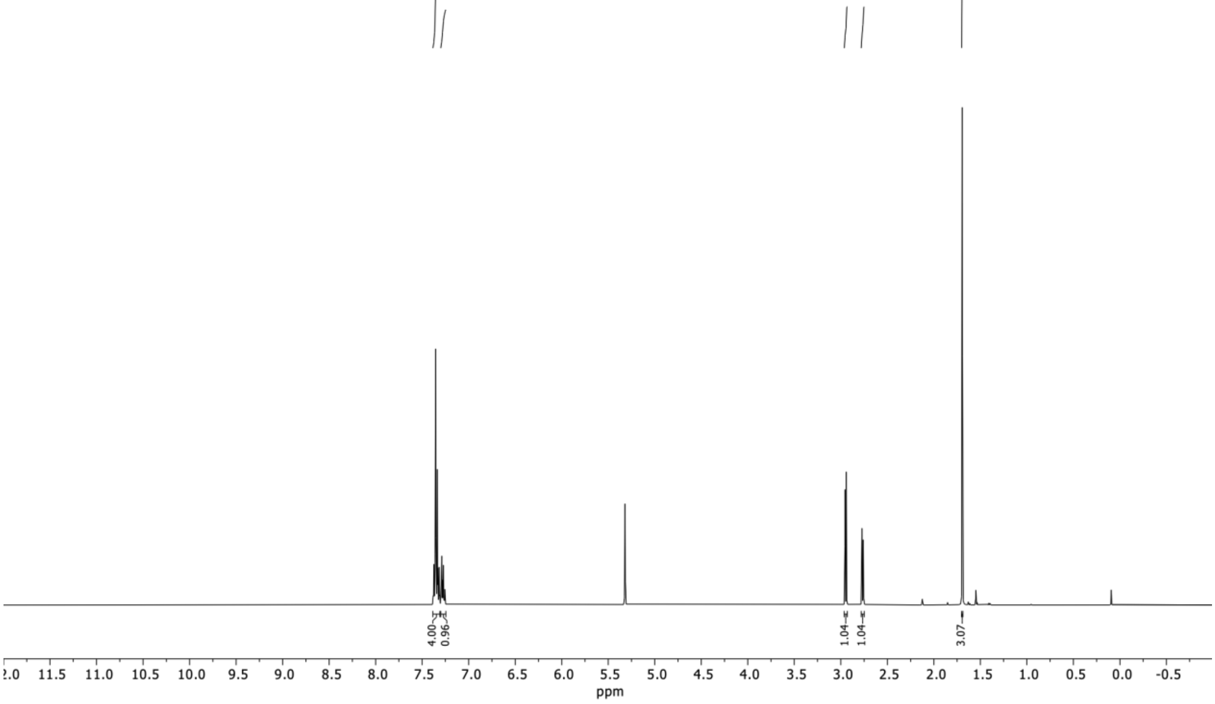

^{13}C-NMR (101 MHz, CD_2Cl_2)

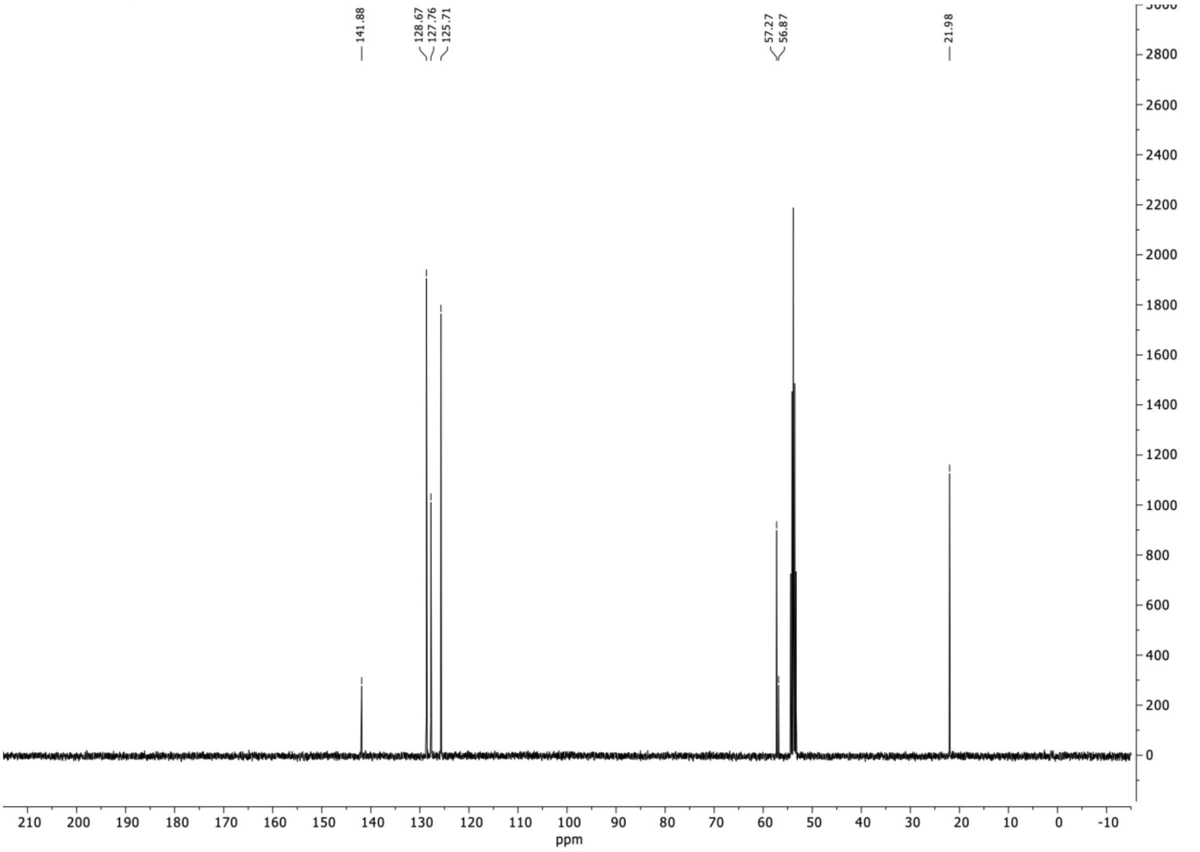

2-Ethyl-2-phenyloxirane

^{1}H-NMR (400 MHz, CD_2Cl_2)

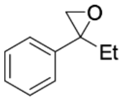

1b

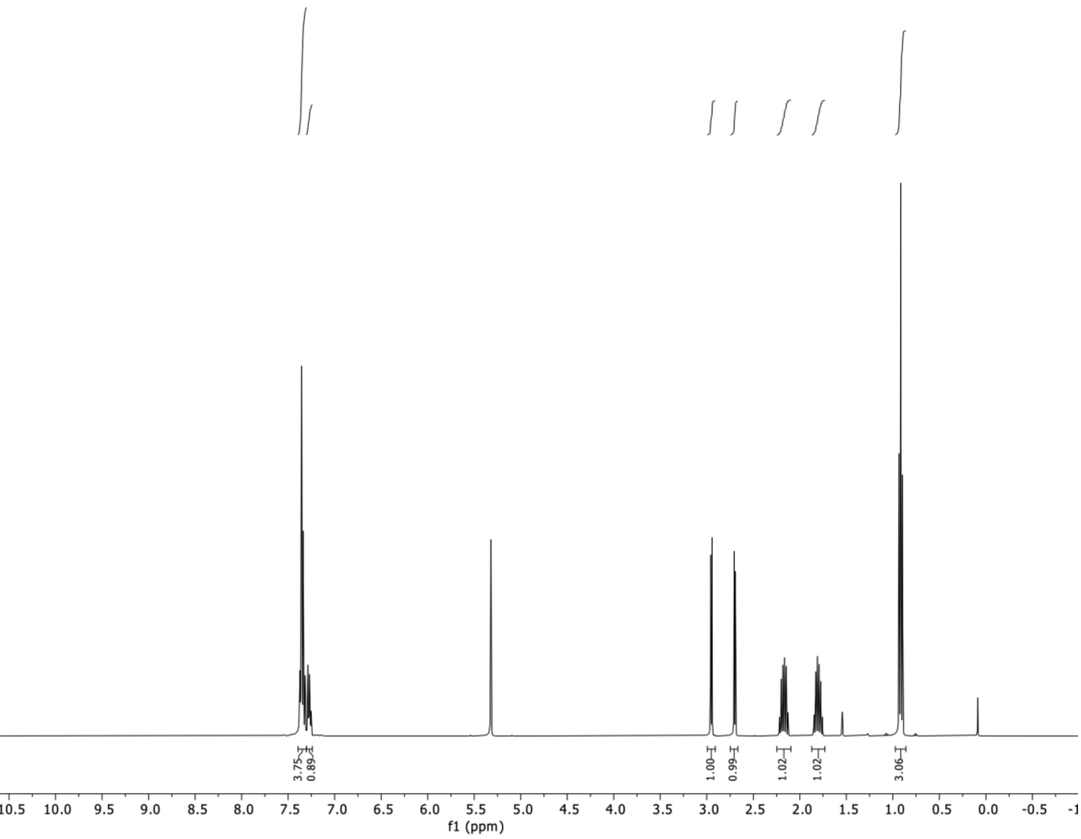

^{13}C-NMR (101 MHz, CD_2Cl_2)

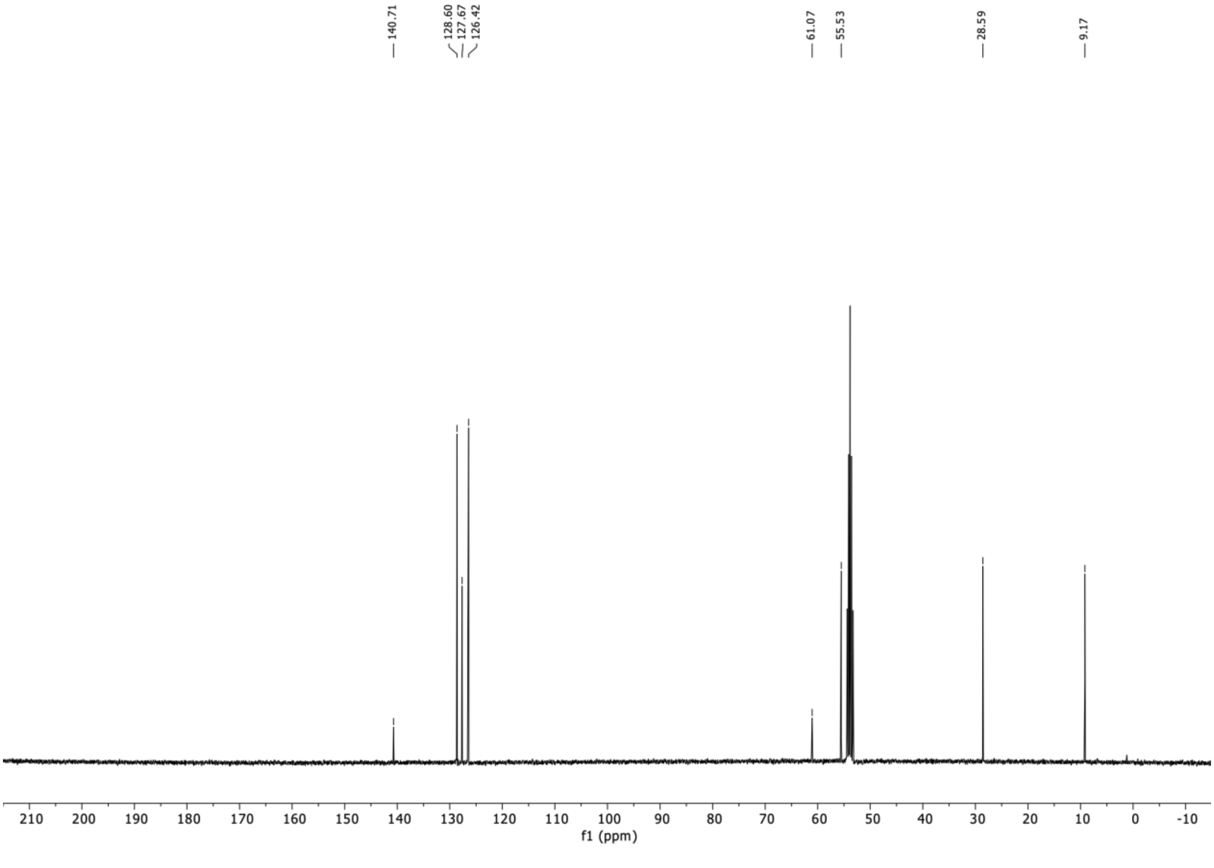

2-Phenyl-2-*n*-propyloxirane

^{1}H-NMR (400 MHz, CD_2Cl_2)

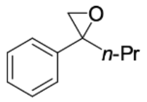

1c

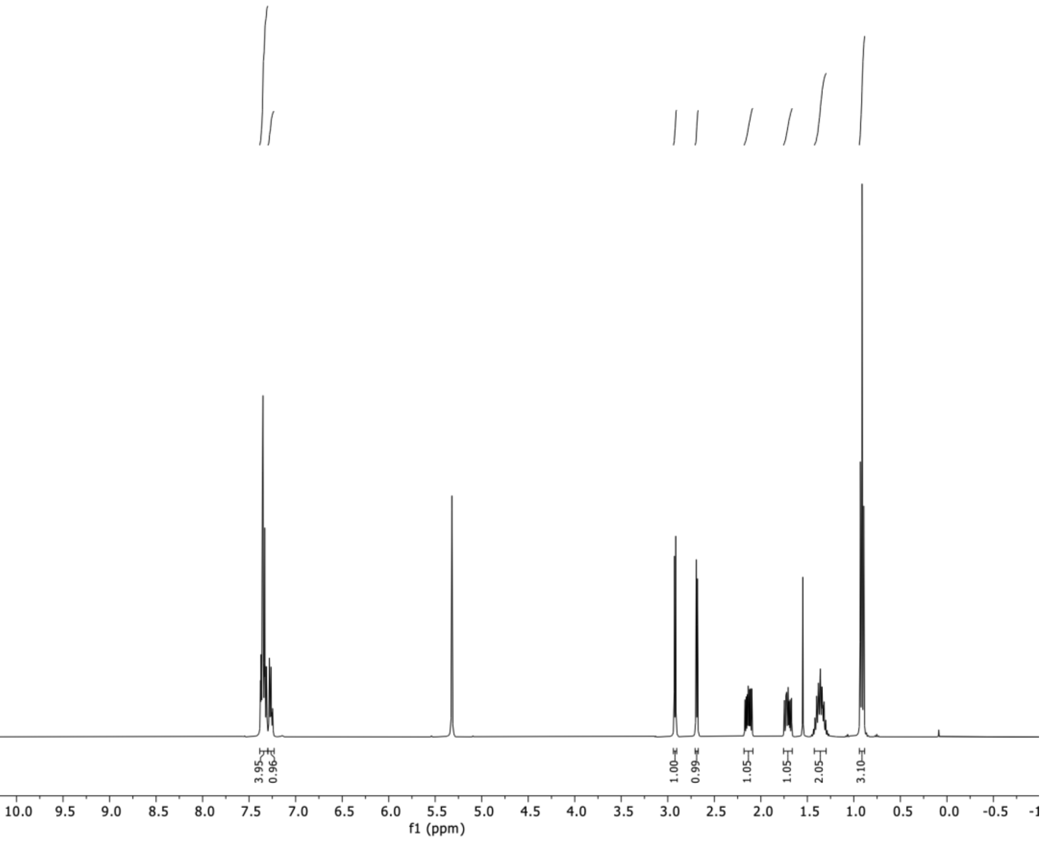

^{13}C-NMR (101 MHz, CD_2Cl_2)

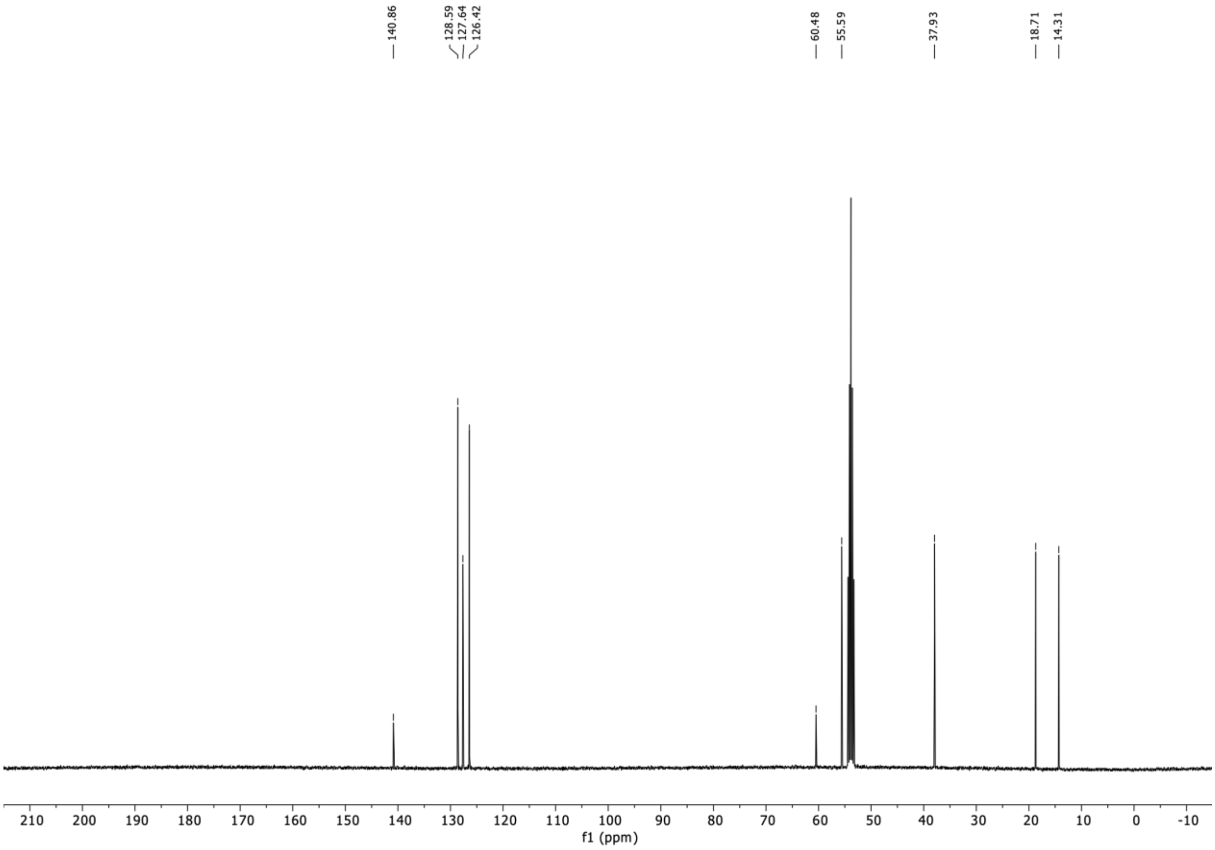

2-Cyclohexyl-2-phenyloxirane

^{1}H-NMR (400 MHz, CD_2Cl_2)

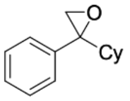

1d

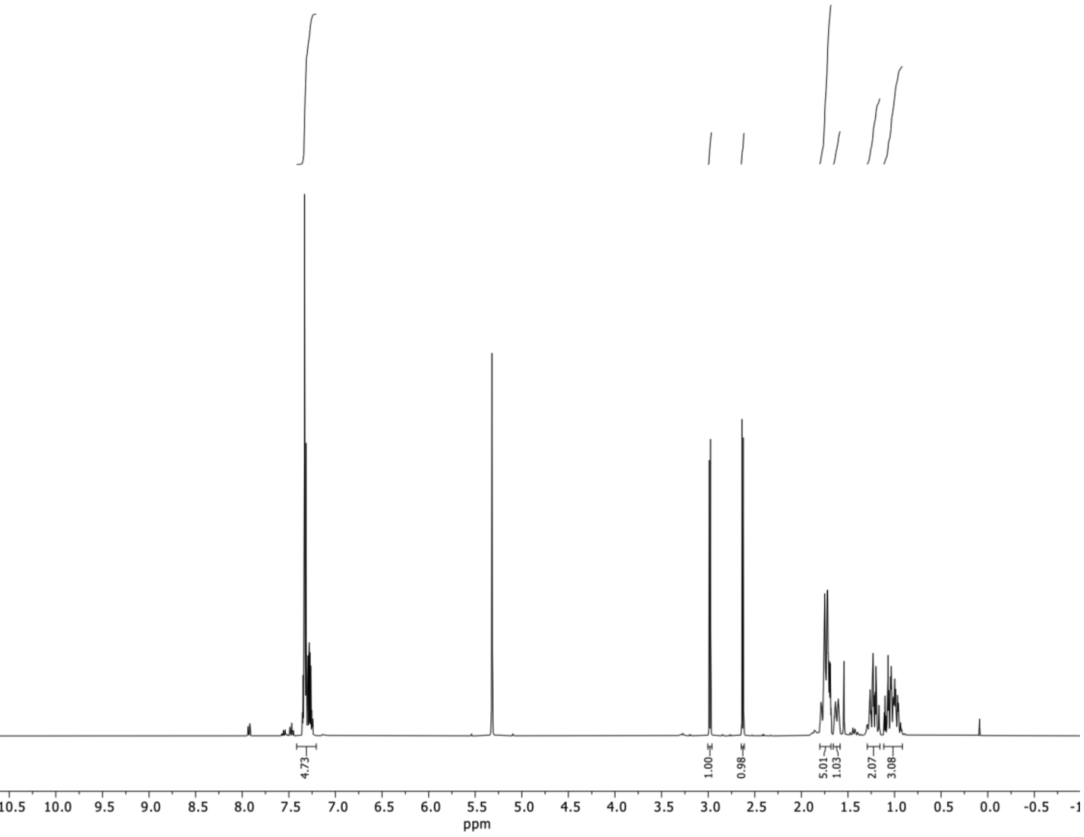

^{13}C-NMR (101 MHz, CD_2Cl_2)

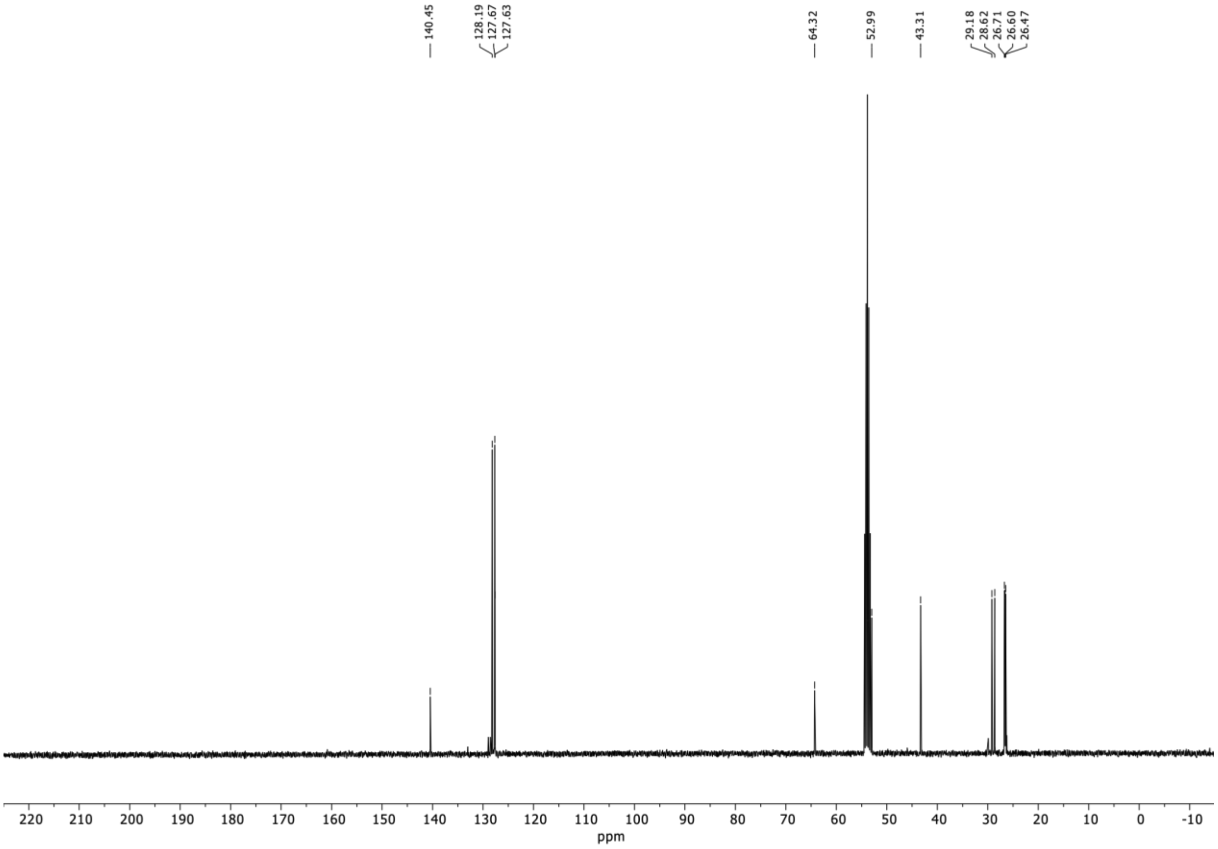

2-(4-Methylphenyl)-2-methyloxirane

^{1}H-NMR (400 MHz, CD_2Cl_2)

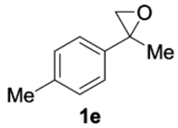

1e

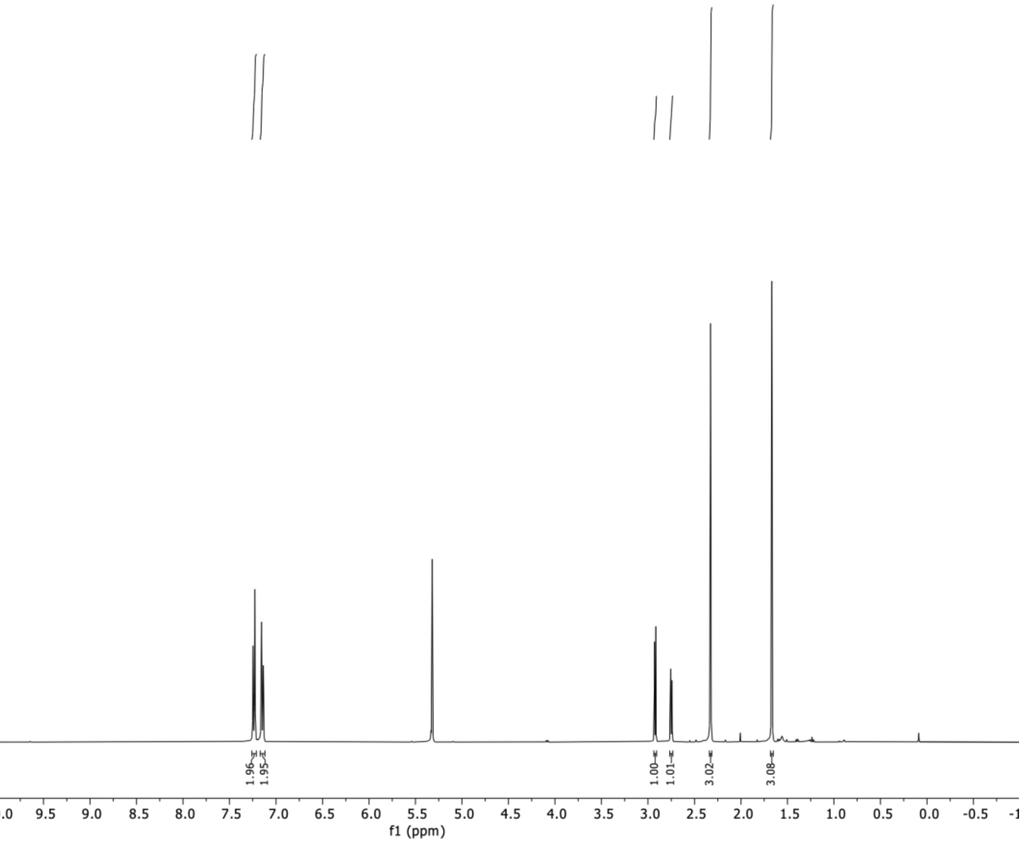

^{13}C-NMR (101 MHz, CD_2Cl_2)

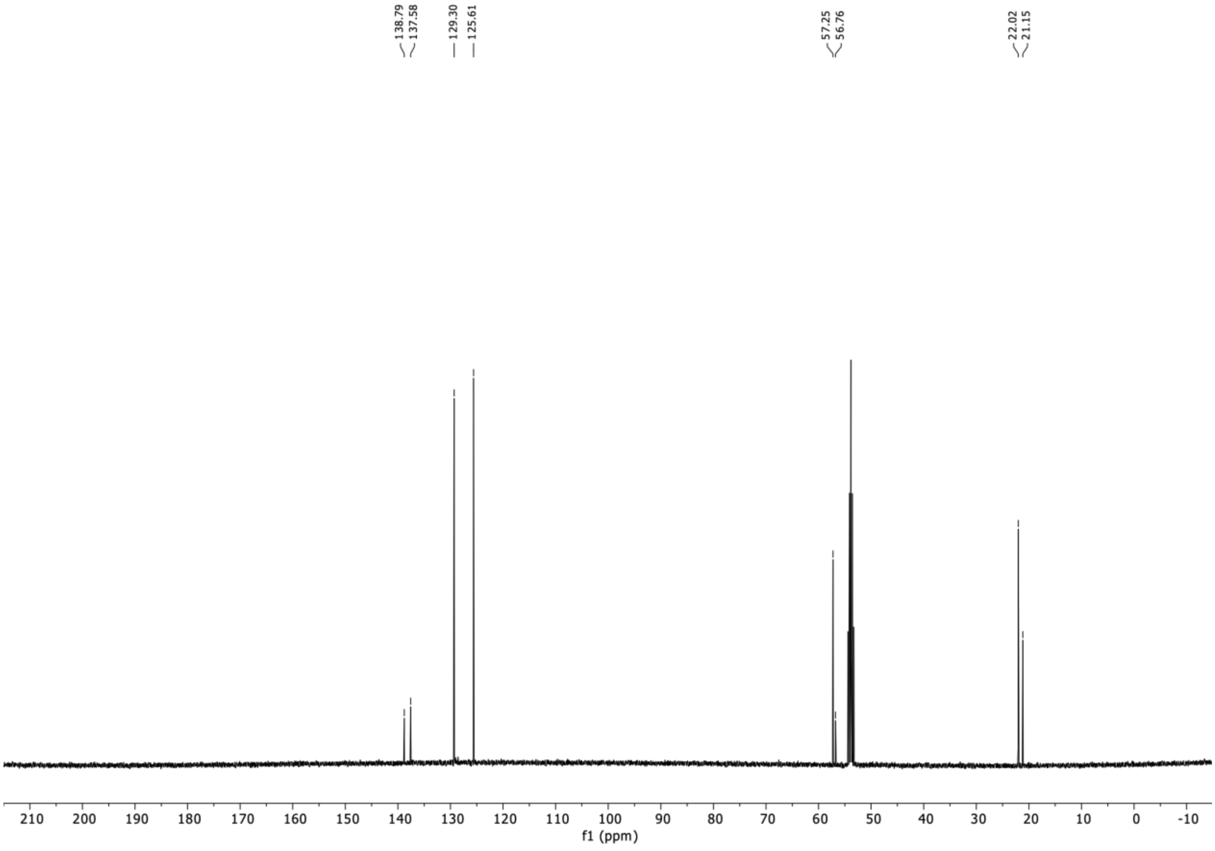

2-(3-Methylphenyl)-2-methyloxirane

^{1}H-NMR (400 MHz, CD_2Cl_2)

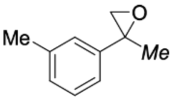

1f

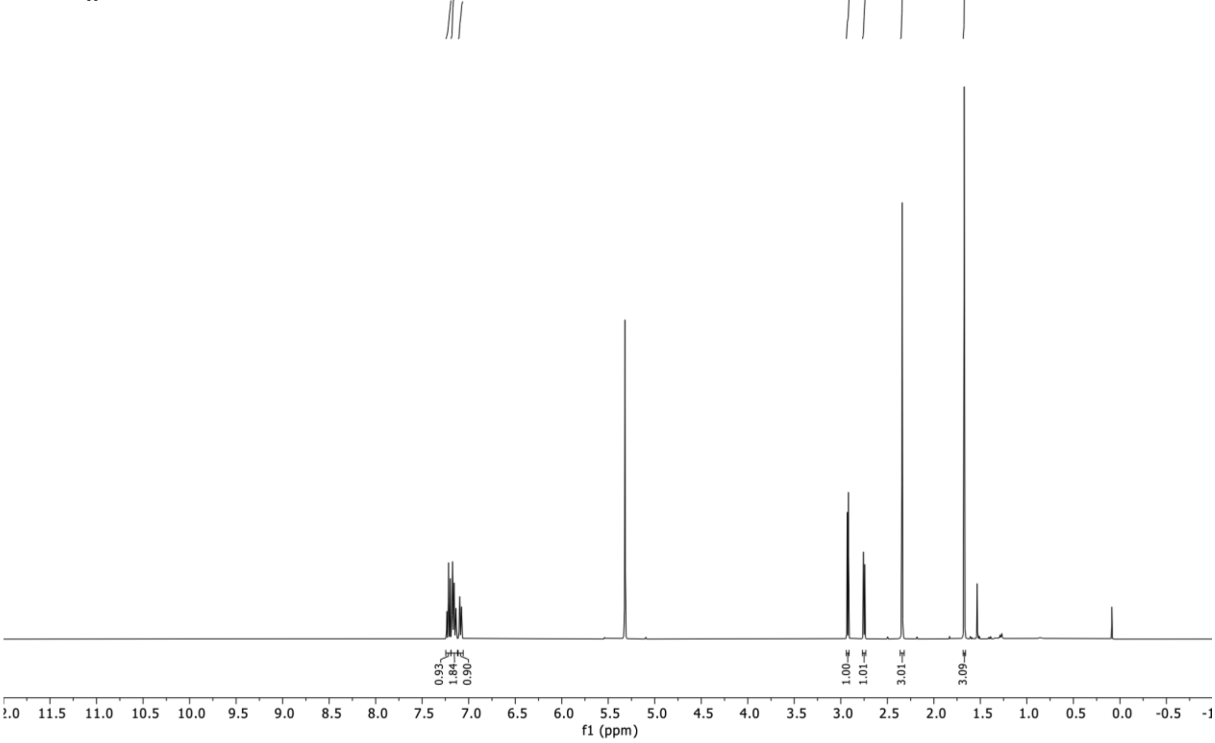

^{13}C-NMR (101 MHz, CD_2Cl_2)

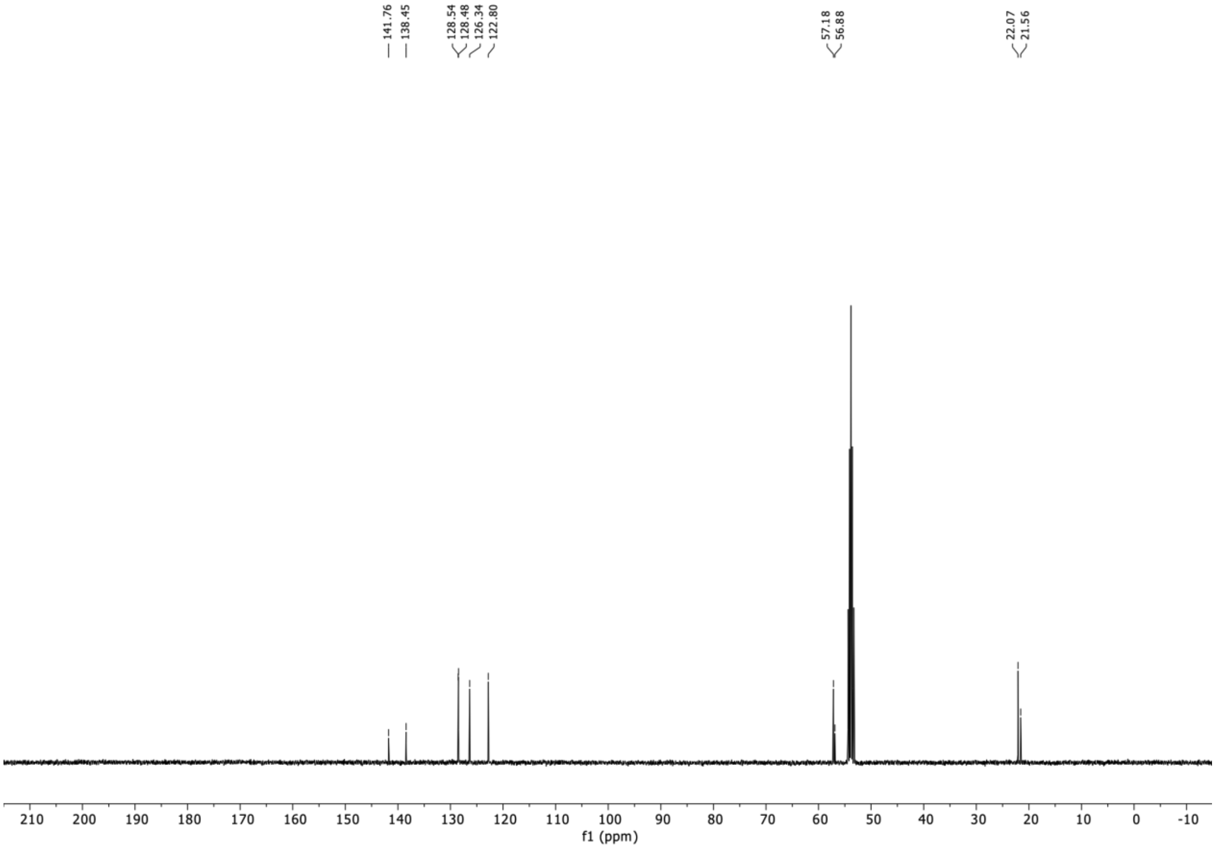

2-(2-Methylphenyl)-2-methyloxirane

^{1}H-NMR (400 MHz, CD_2Cl_2)

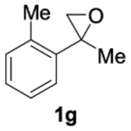

1g

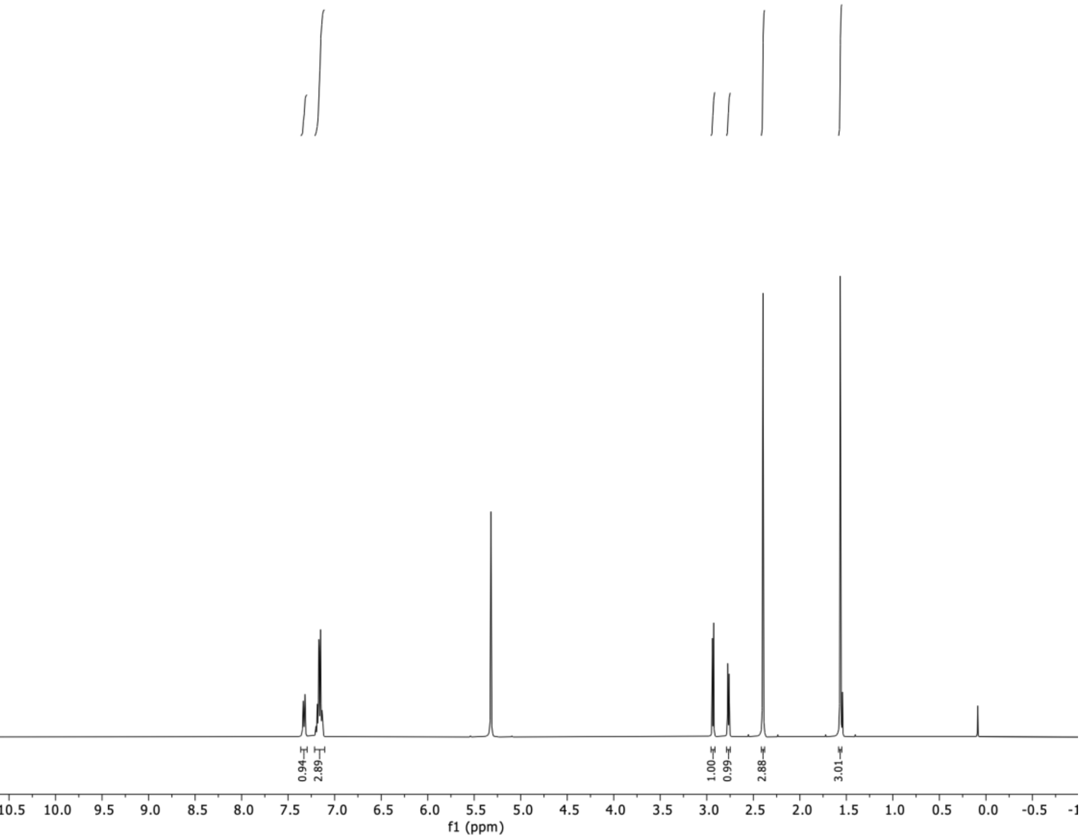

^{13}C-NMR (101 MHz, CD_2Cl_2)

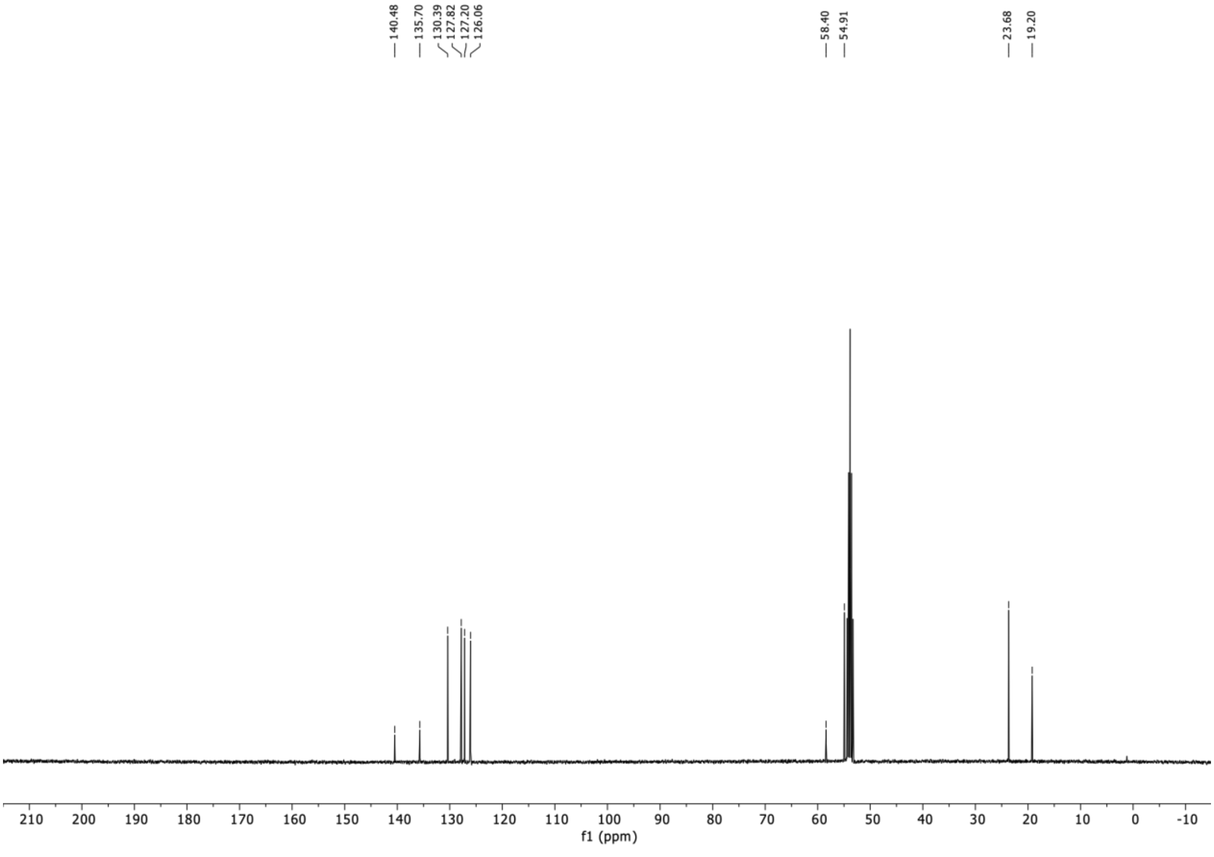

2-(4-Methoxyphenyl)-2-methyloxirane

^{1}H-NMR (400 MHz, C_6D_6)

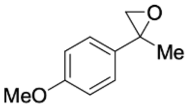

1h

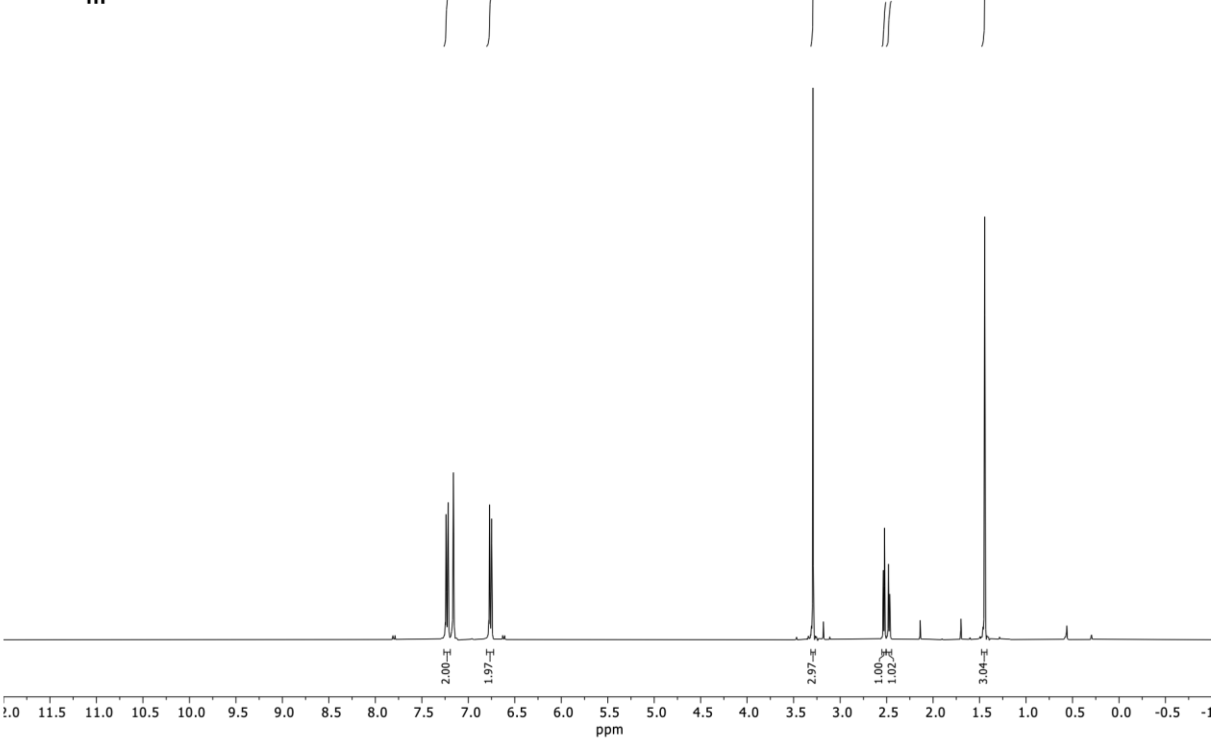

^{13}C-NMR (101 MHz, C_6D_6)

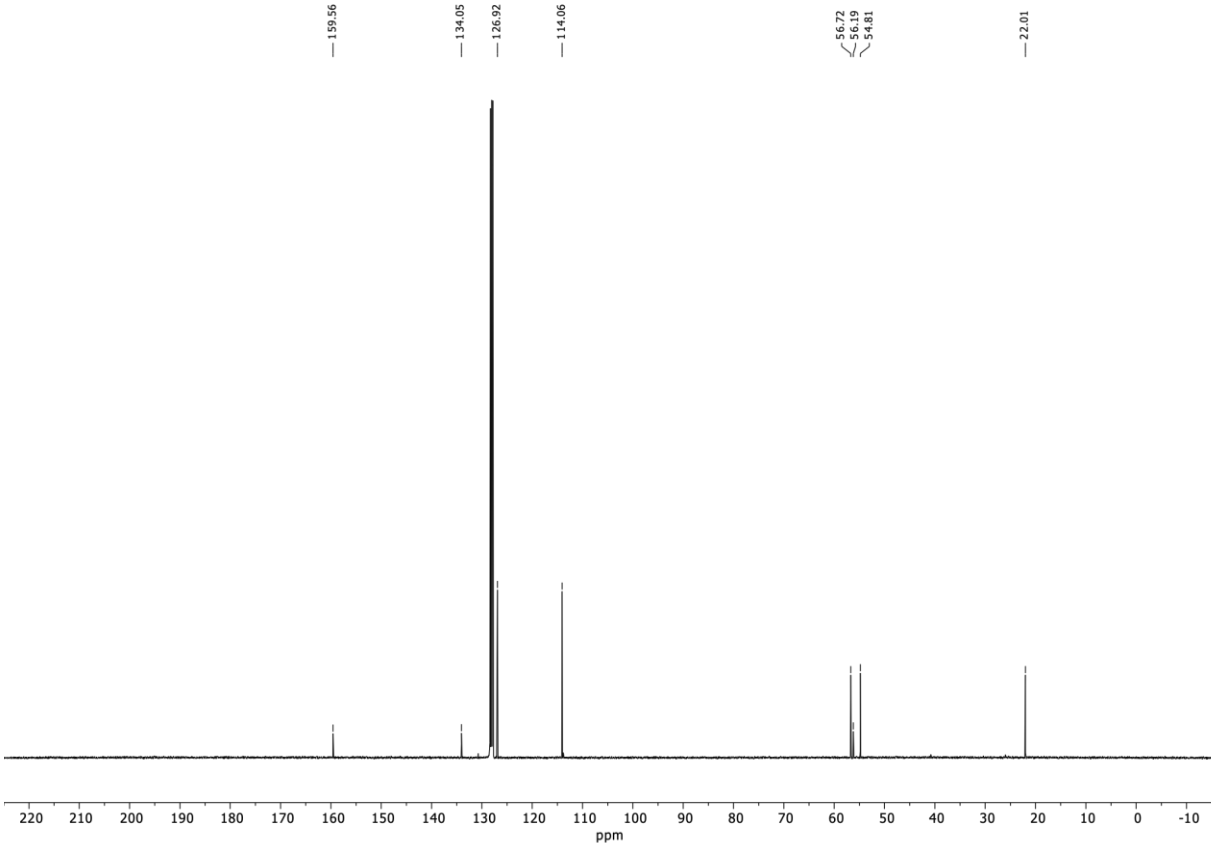

2-(4-Methylphenyl)-2-phenyloxirane

^{1}H-NMR (400 MHz, CD_2Cl_2)

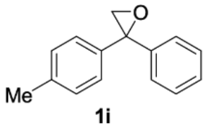

1i

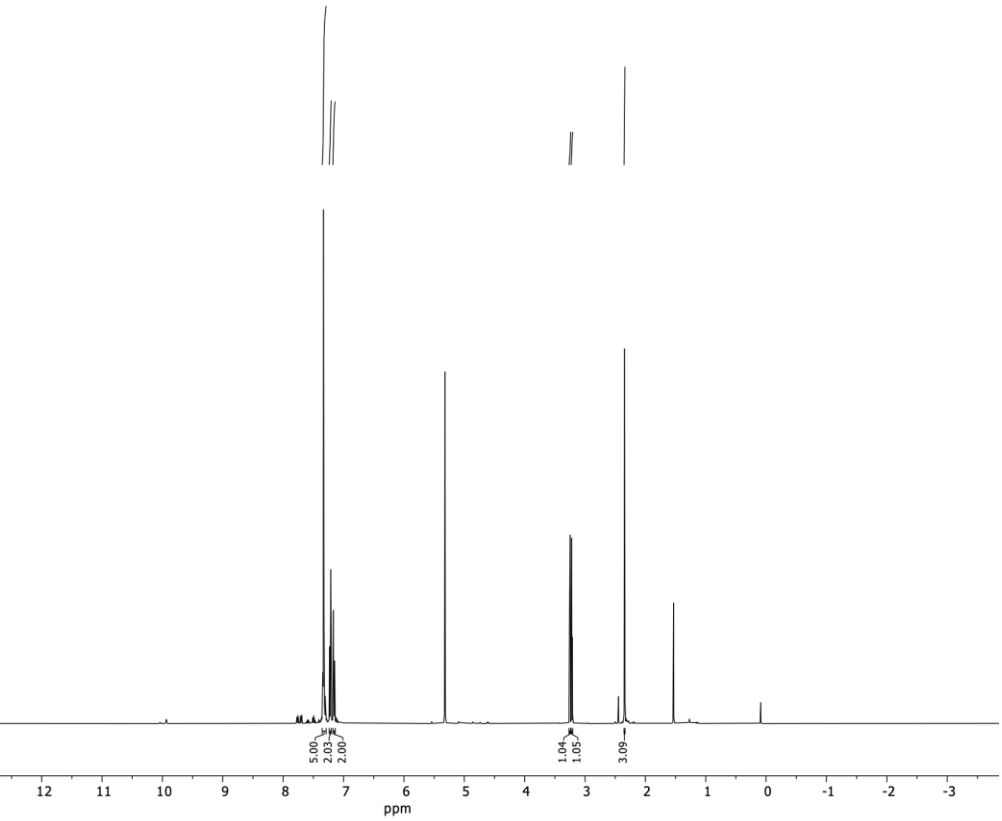

^{13}C-NMR (101 MHz, CD_2Cl_2)

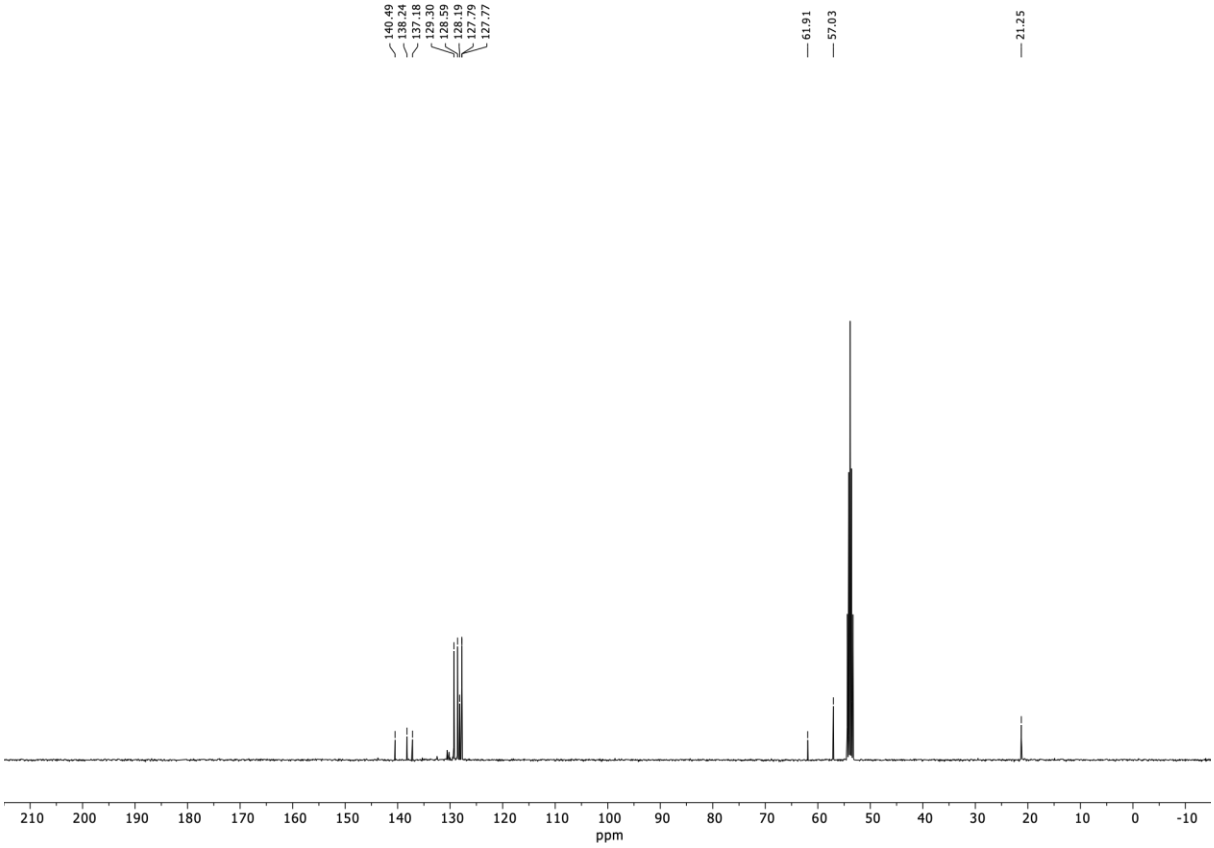

2-(4-Fluorophenyl)-2-methyloxirane

^{1}H-NMR (400 MHz, CD_2Cl_2)

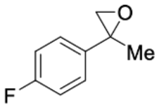

1j

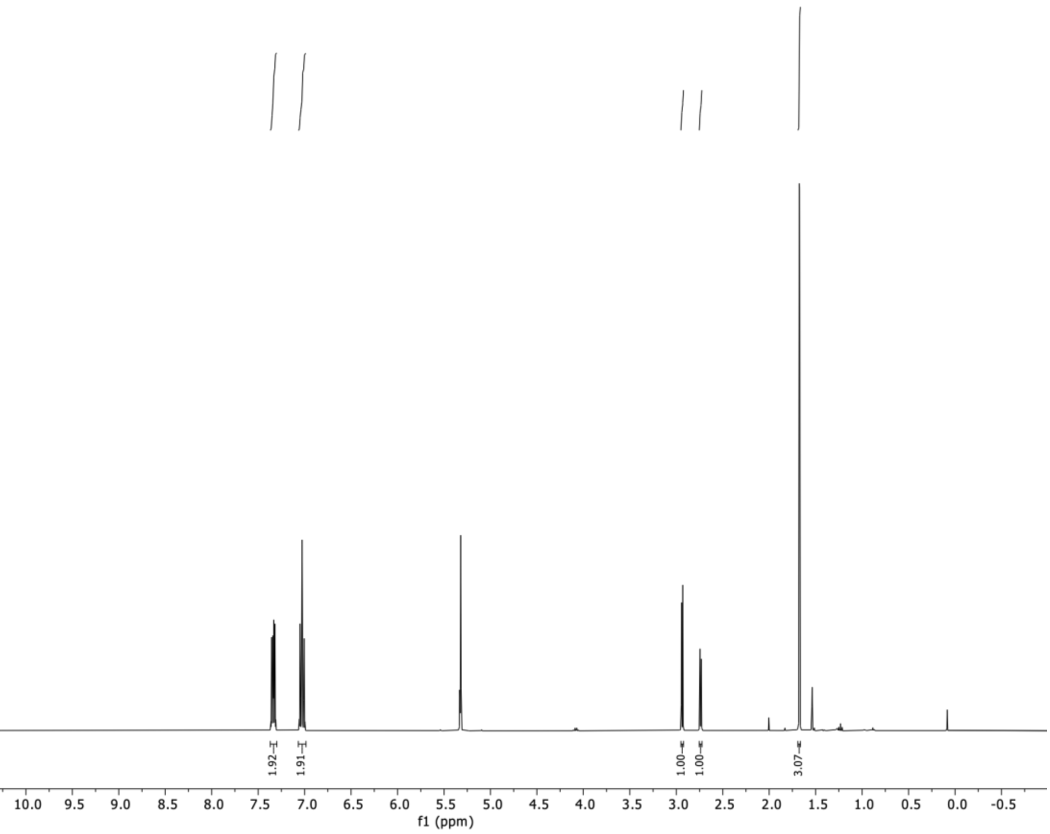

^{13}C-NMR (101 MHz, CD_2Cl_2)

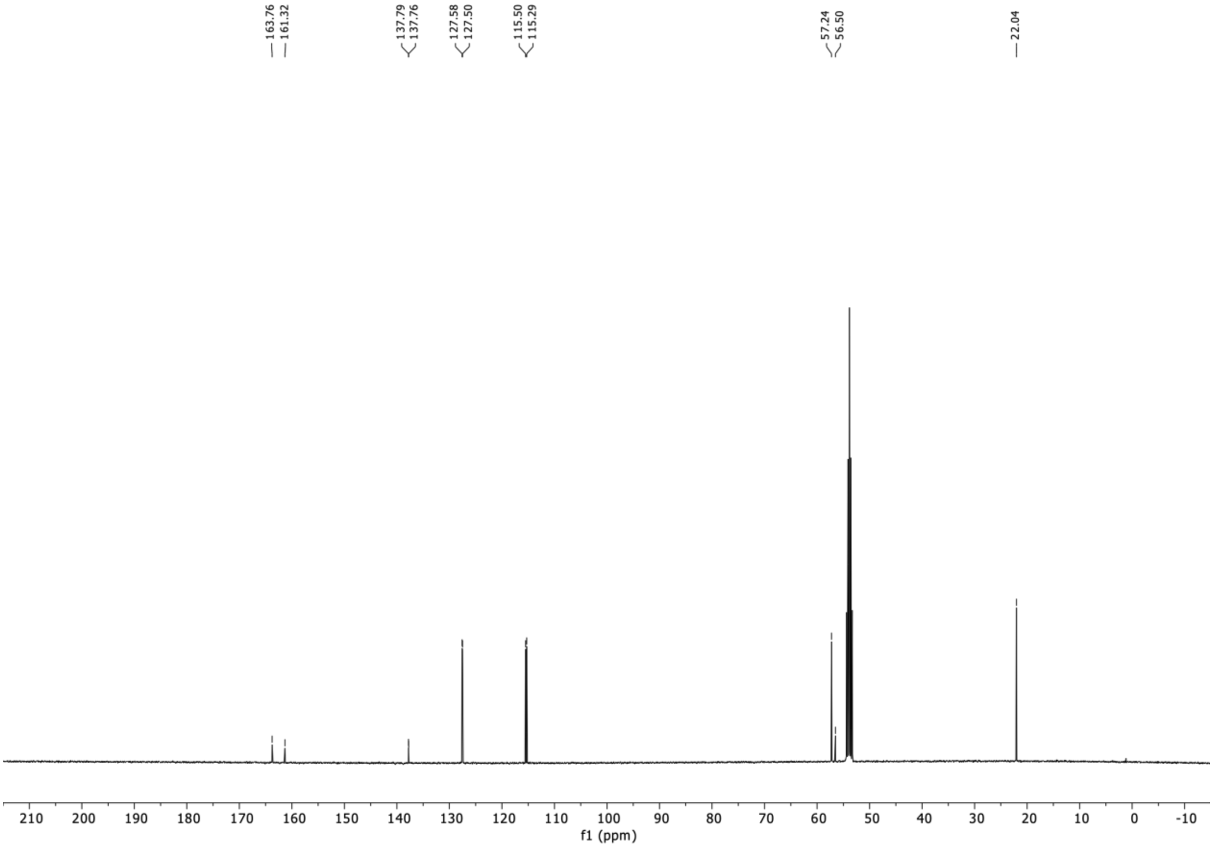

^{19}F-NMR (377 MHz, CD_2Cl_2)

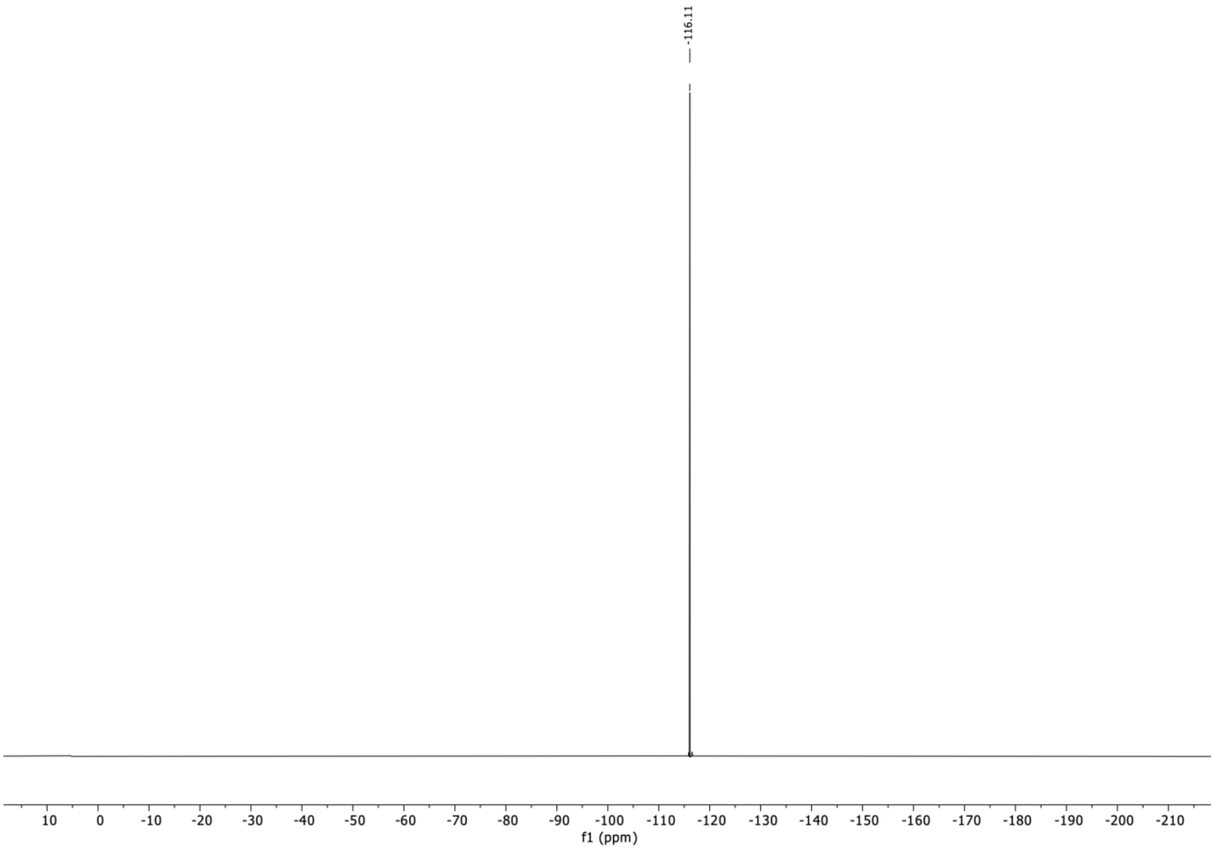

2-(4-Chlorophenyl)-2-methyloxirane

^{1}H-NMR (400 MHz, CD_2Cl_2)

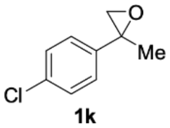

1k

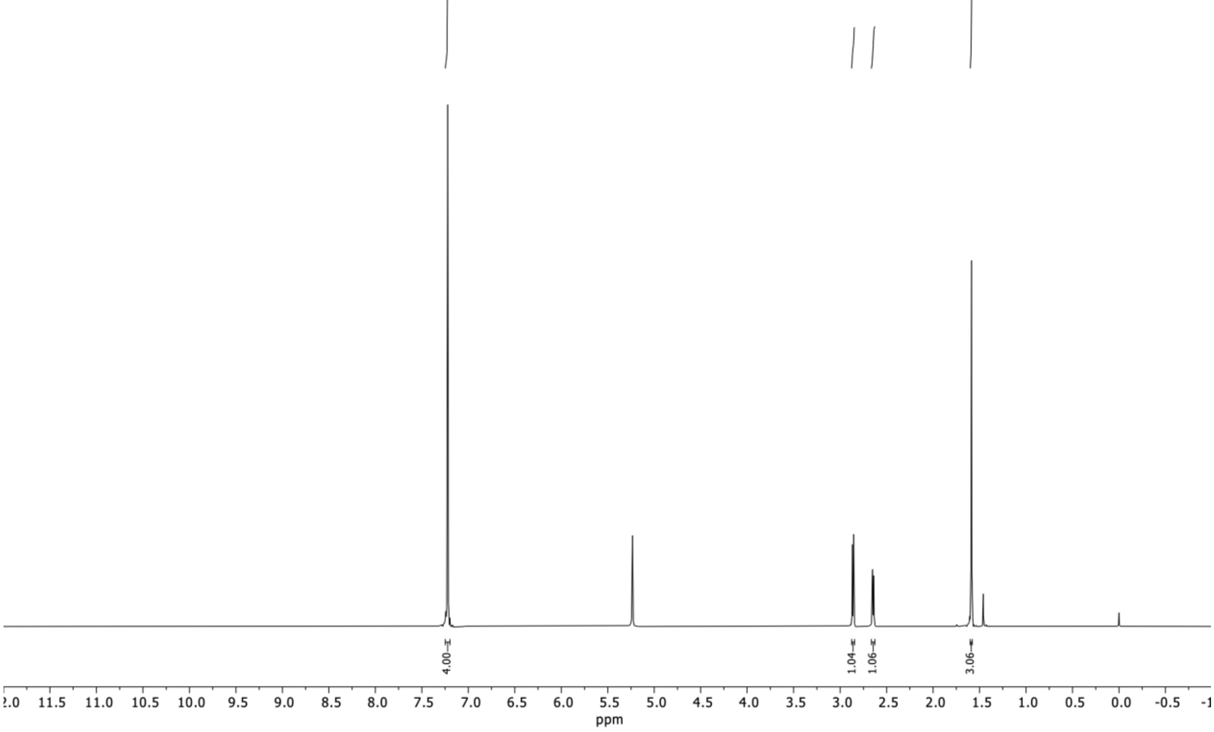

^{13}C-NMR (101 MHz, CD_2Cl_2)

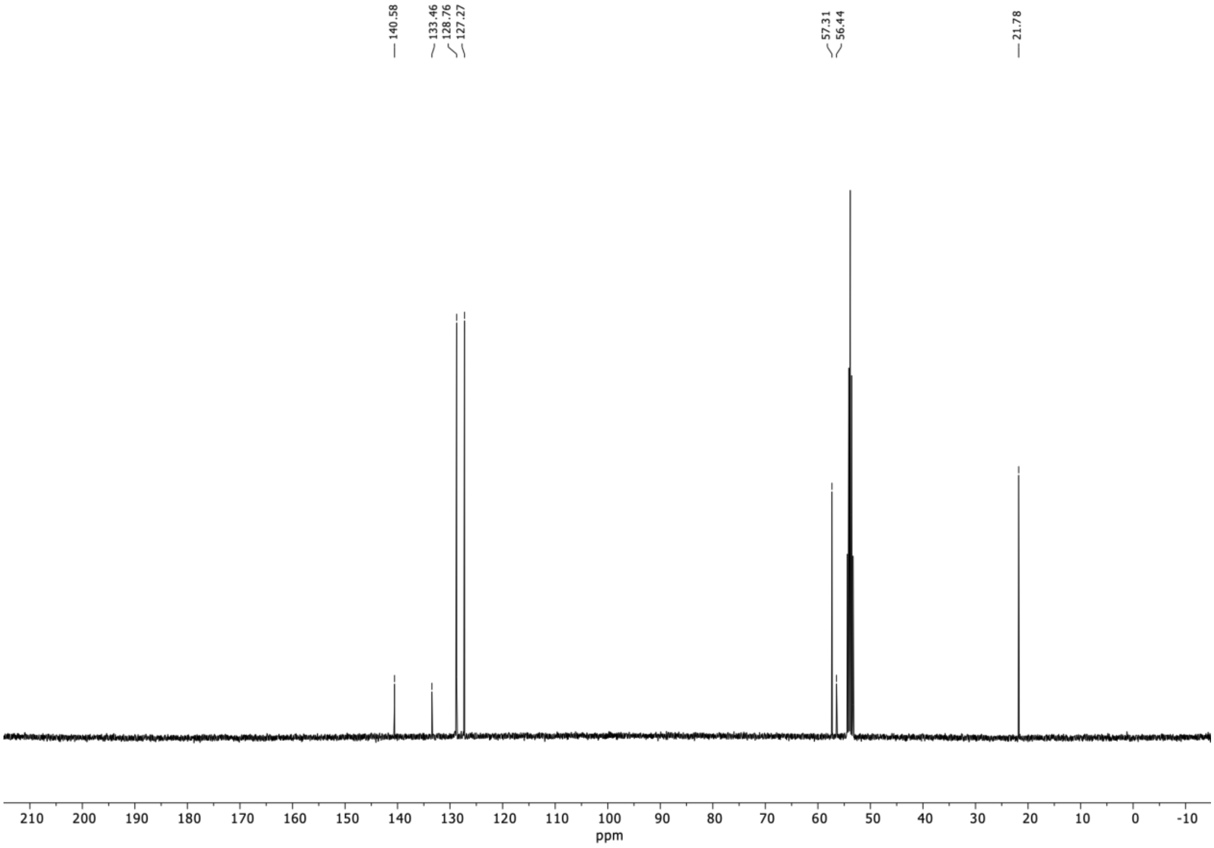

2-(4-Bromophenyl)-2-methyloxirane

^{1}H-NMR (400 MHz, CD_2Cl_2)

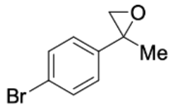

1l

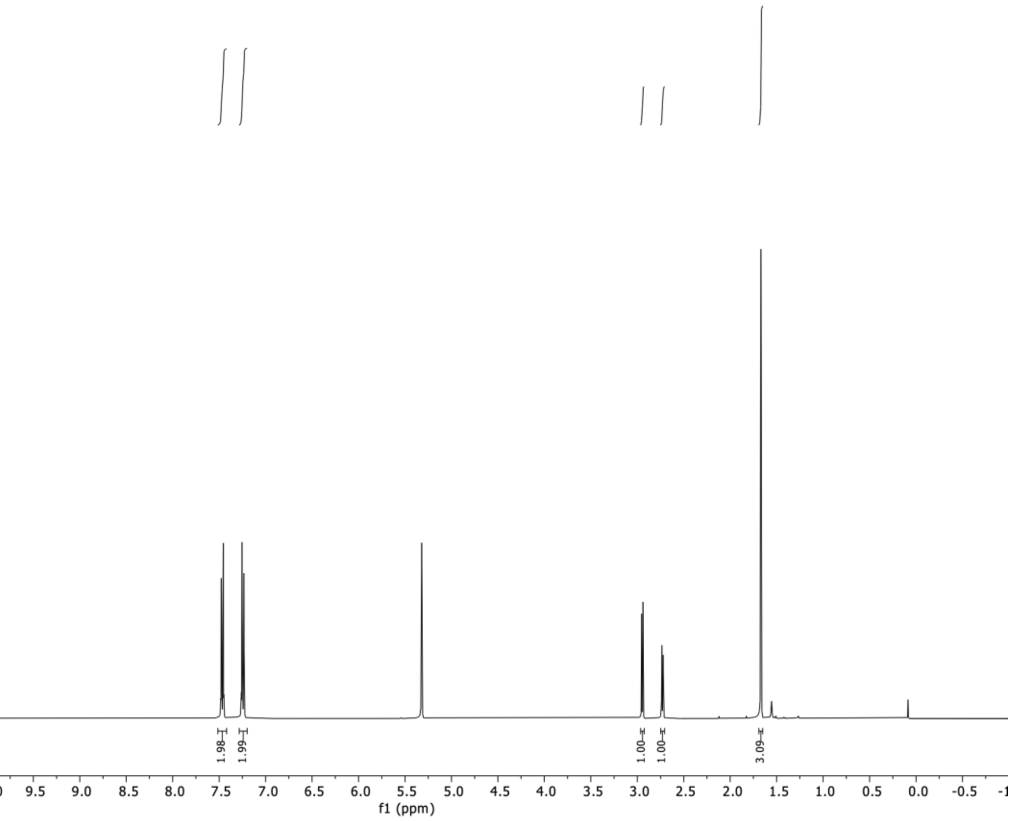

^{13}C-NMR (101 MHz, CD_2Cl_2)

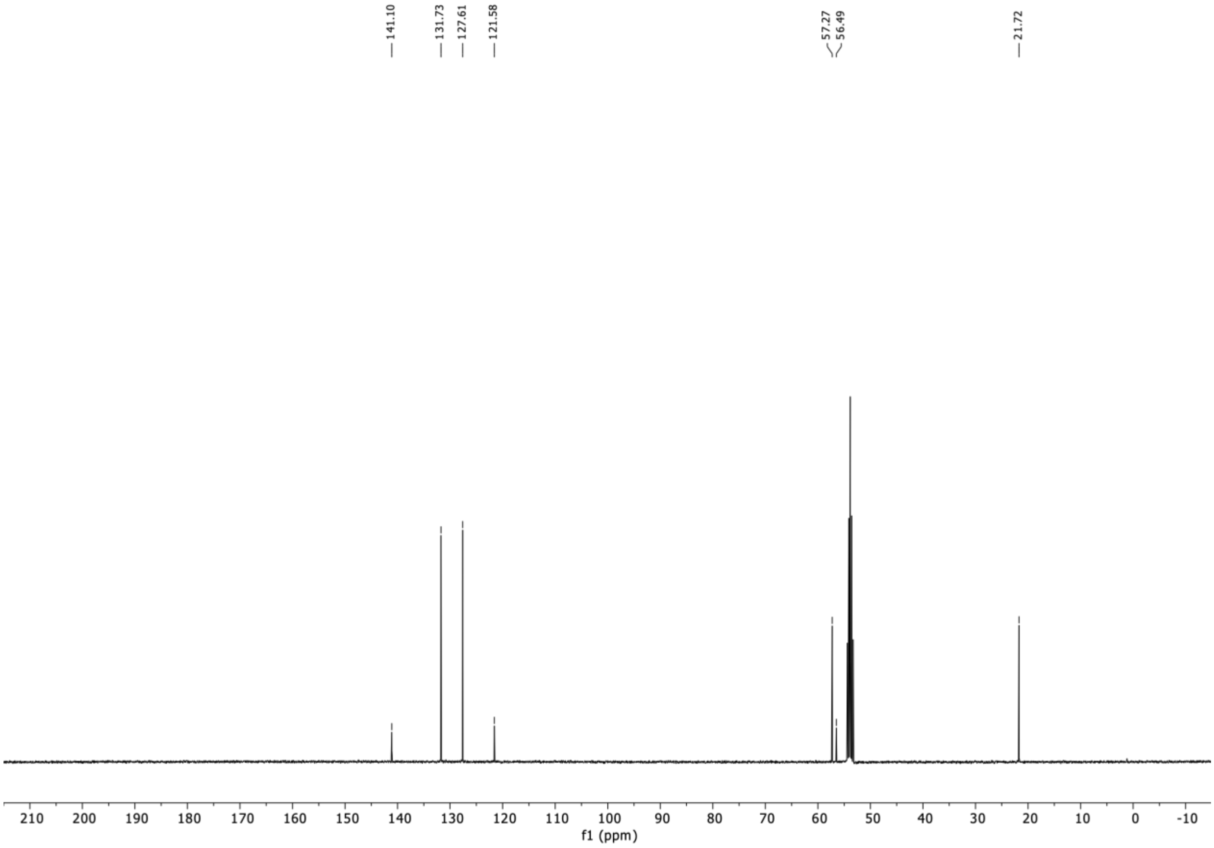

2-(4-Iodophenyl)-2-methyloxirane

^{1}H-NMR (400 MHz, CD_2Cl_2)

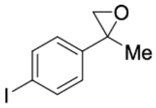

1m

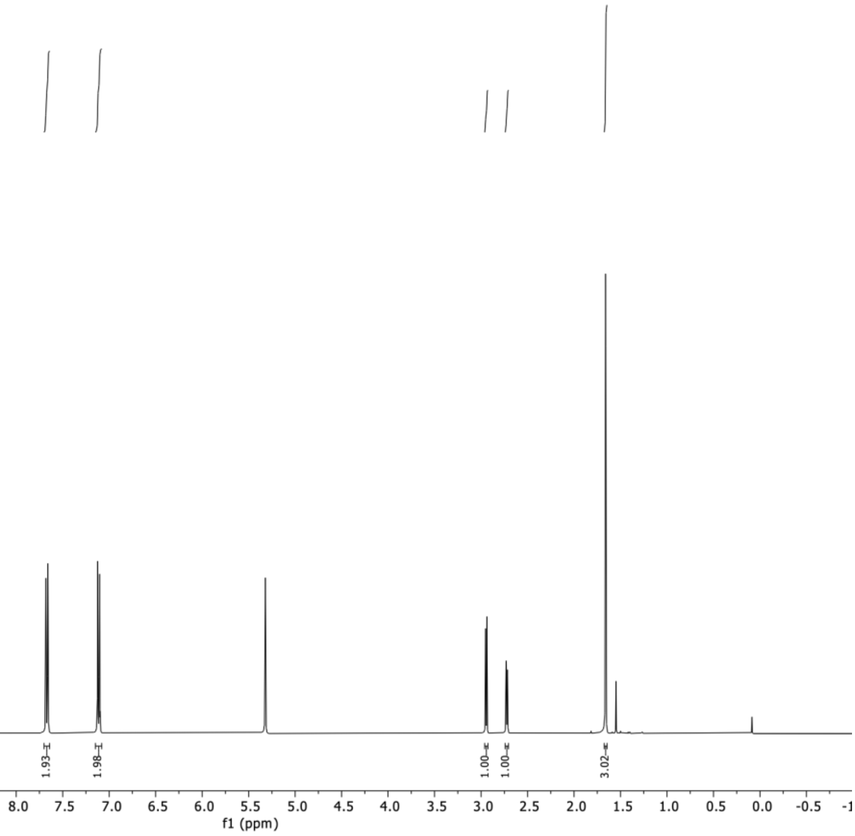

^{13}C-NMR (101 MHz, CD_2Cl_2)

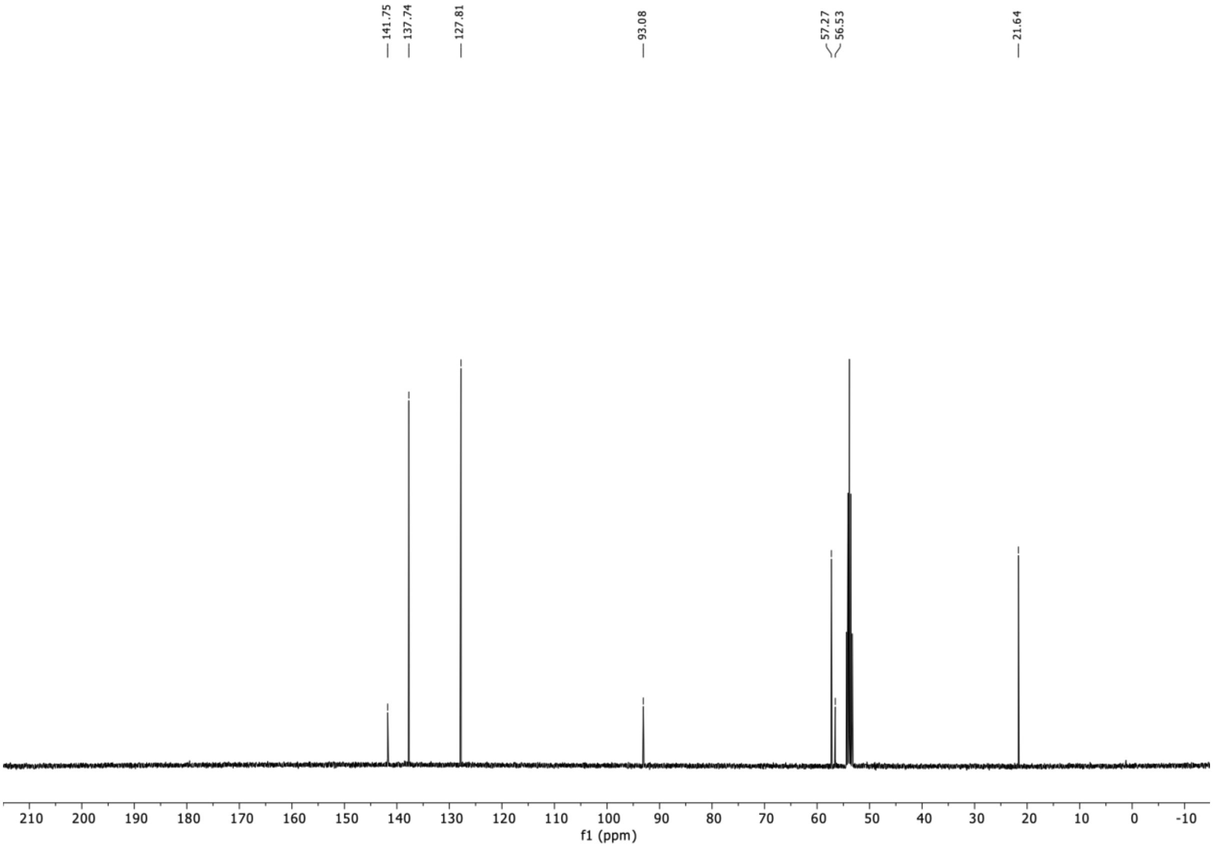

2-(4-Trifluoromethylphenyl)-2-methyloxirane

^{1}H-NMR (400 MHz, CD_2Cl_2)

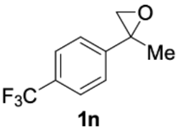

1n

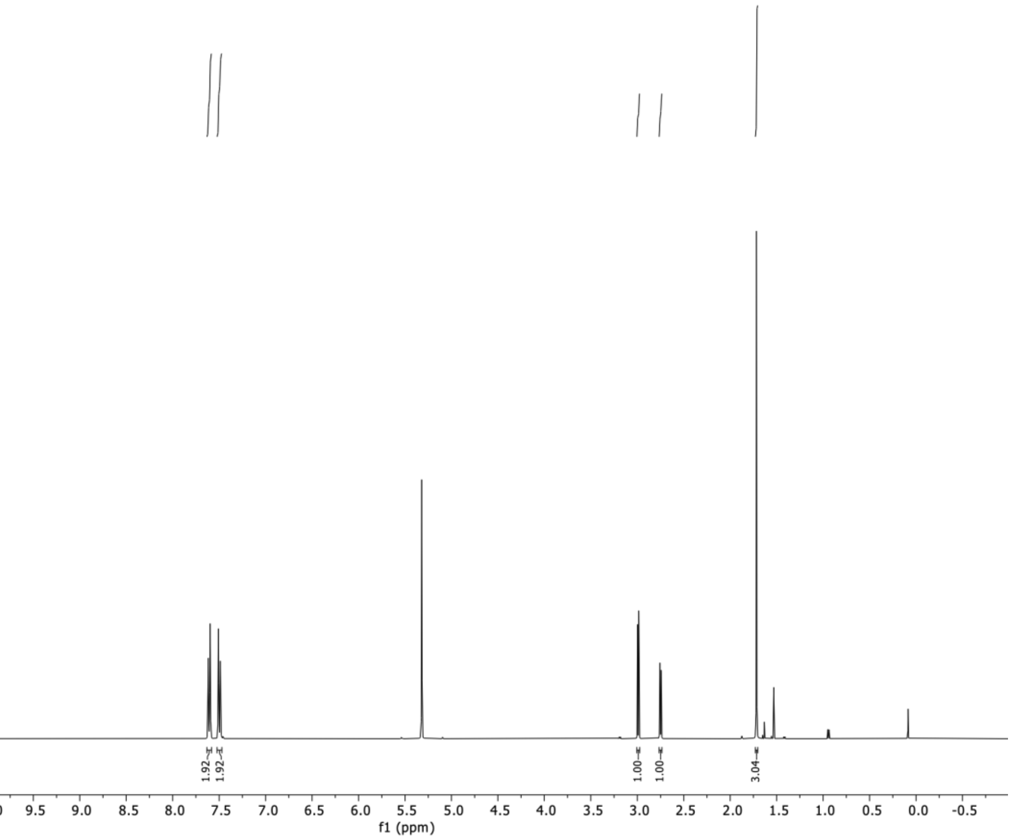

^{13}C-NMR (101 MHz, CD_2Cl_2)

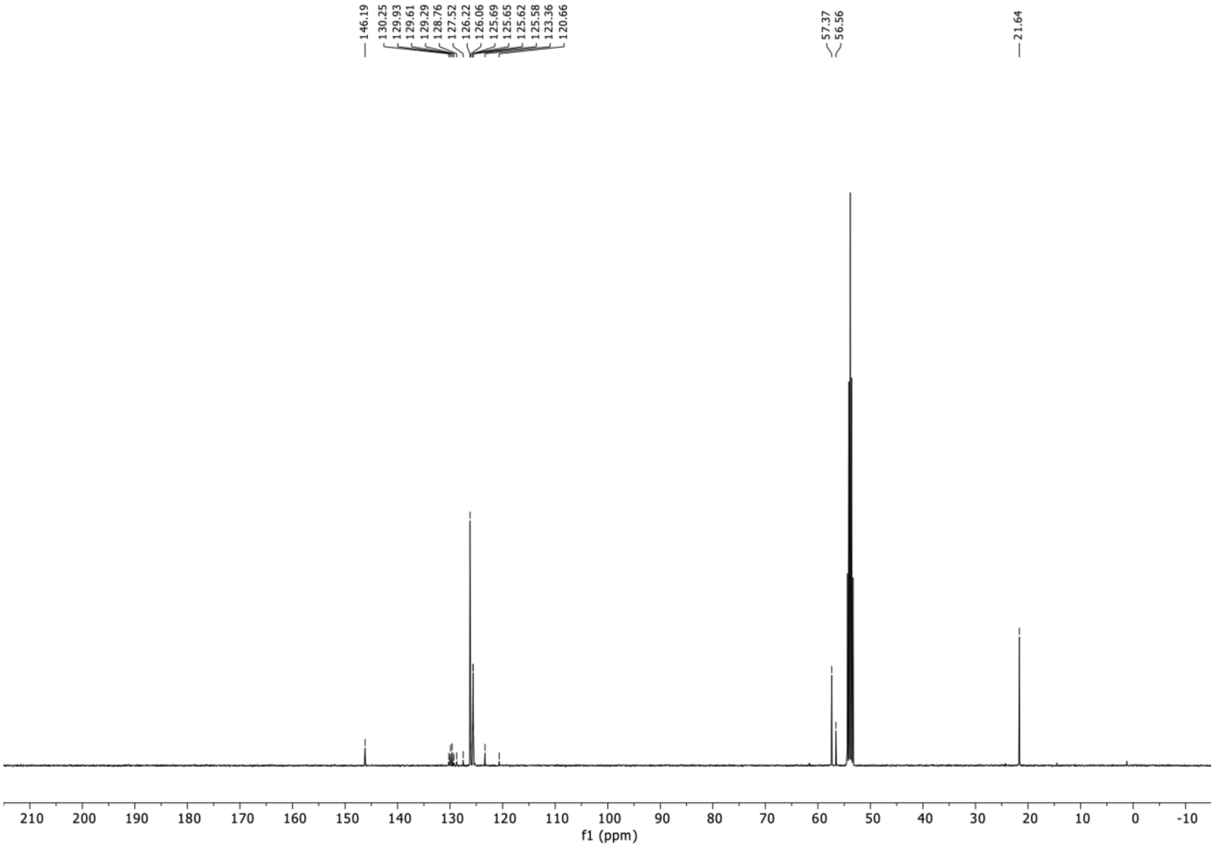

^{19}F-NMR (377 MHz, CD_2Cl_2)

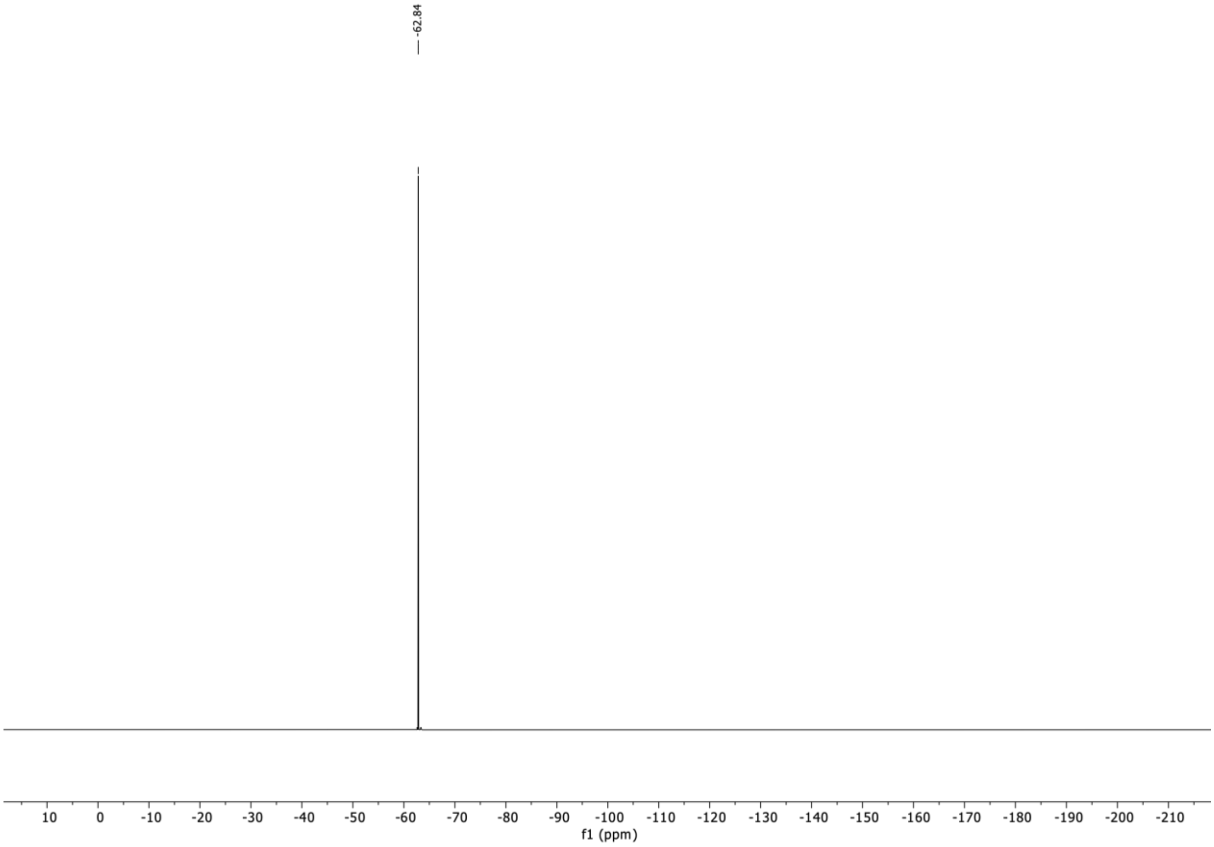

2-(4-Cyanophenyl)-2-methyloxirane

^{1}H-NMR (400 MHz, CD_2Cl_2)

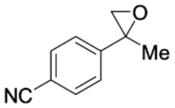

1o

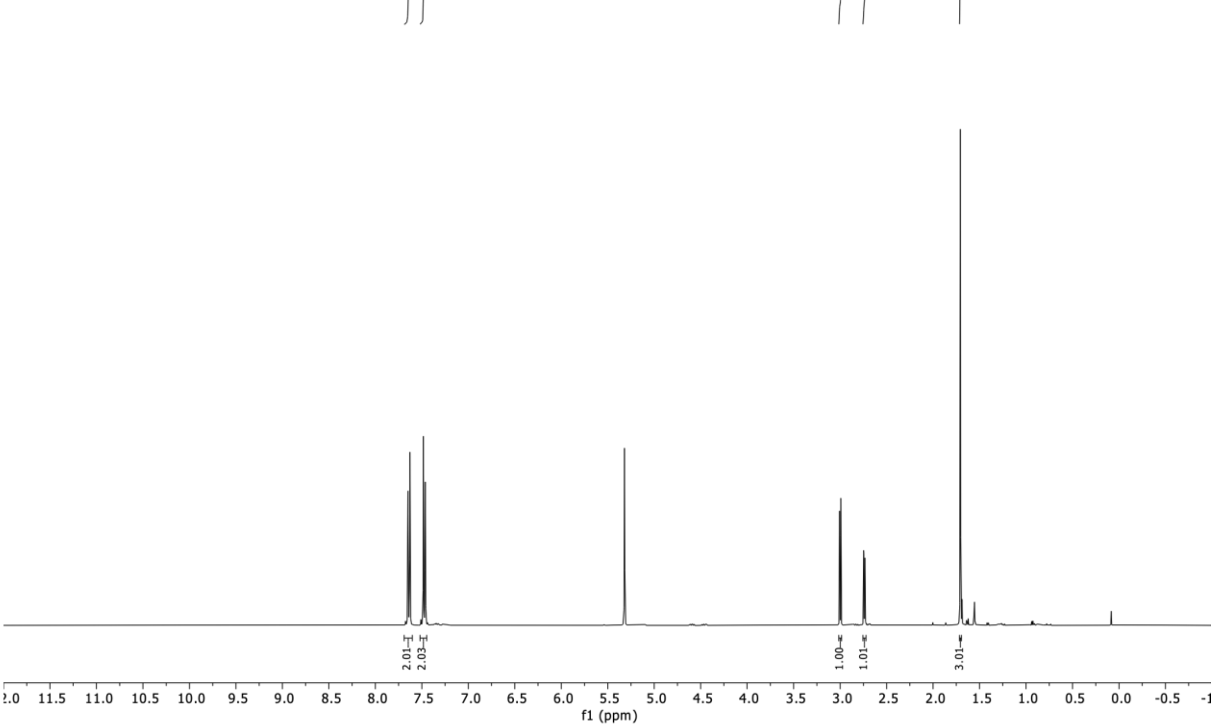

^{13}C-NMR (101 MHz, CD_2Cl_2)

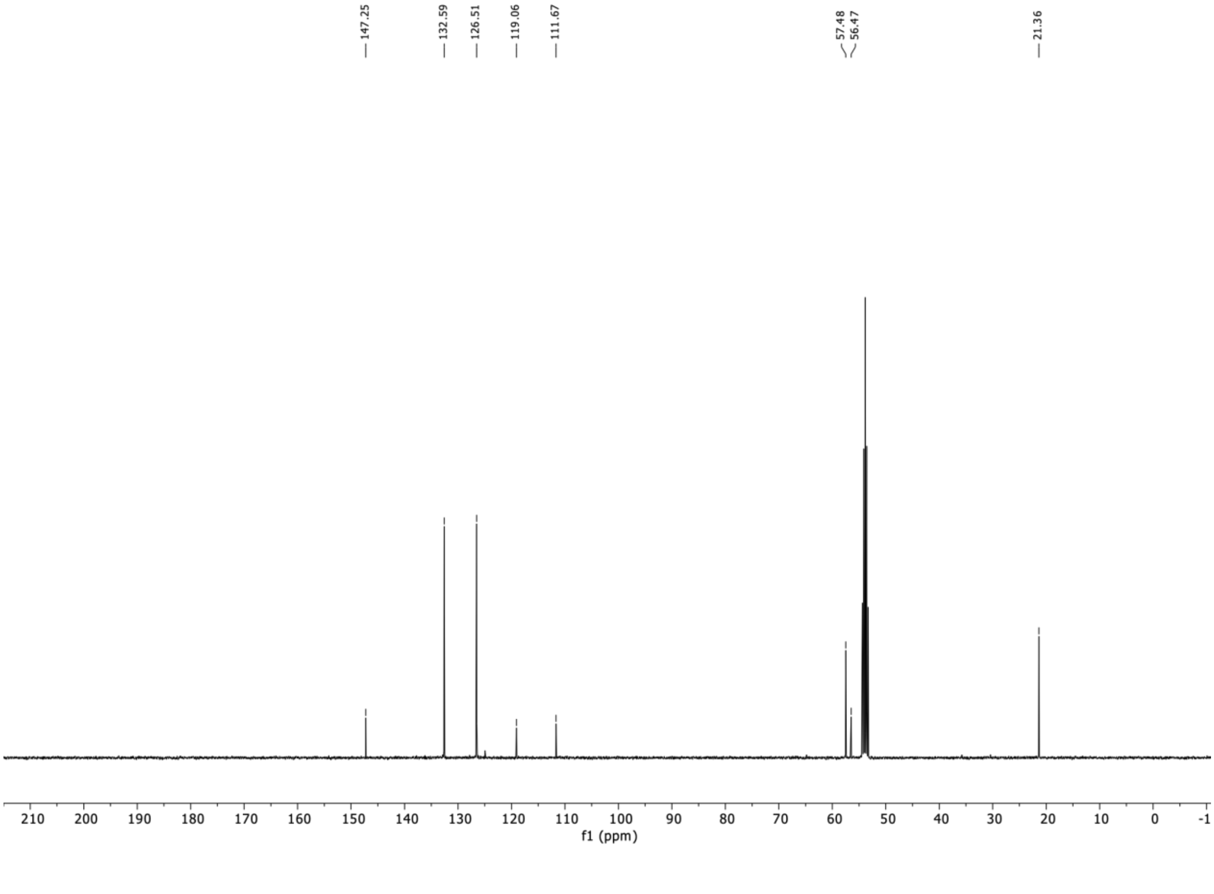

2-(4-Nitrophenyl)-2-methyloxirane

^{1}H-NMR (400 MHz, CD_2Cl_2)

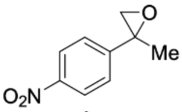

1p

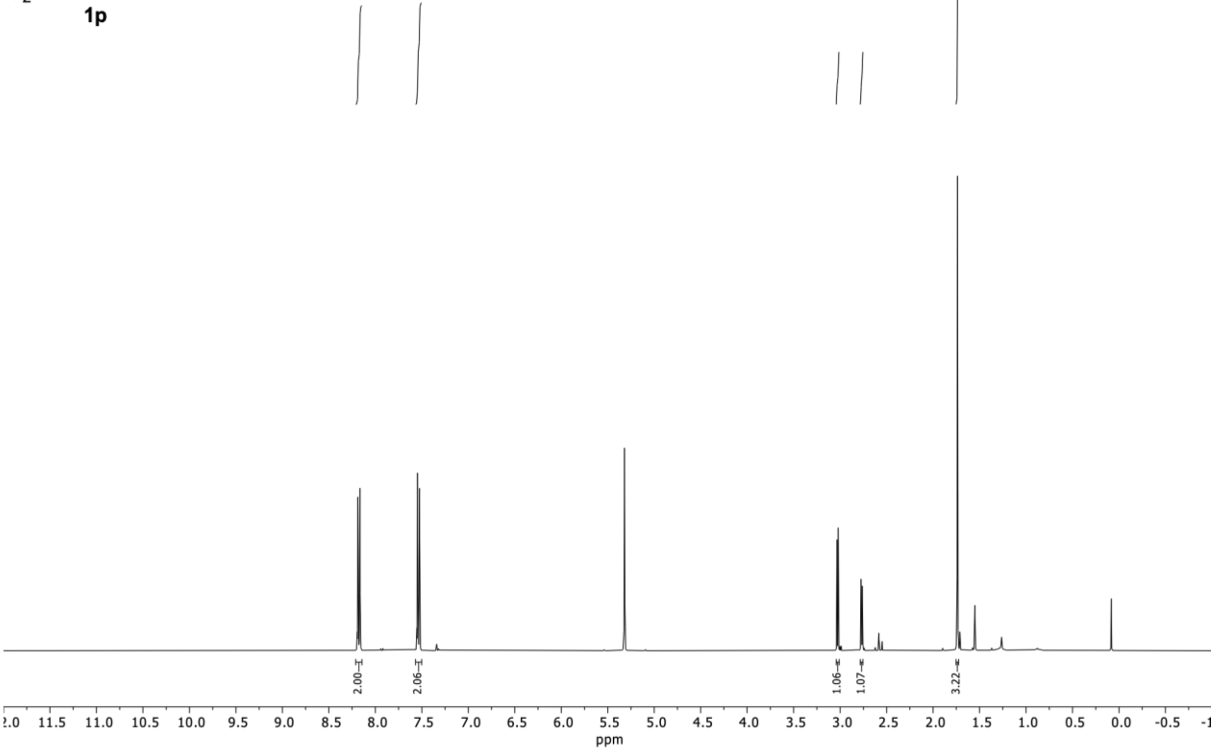

^{13}C-NMR (101 MHz, CD_2Cl_2)

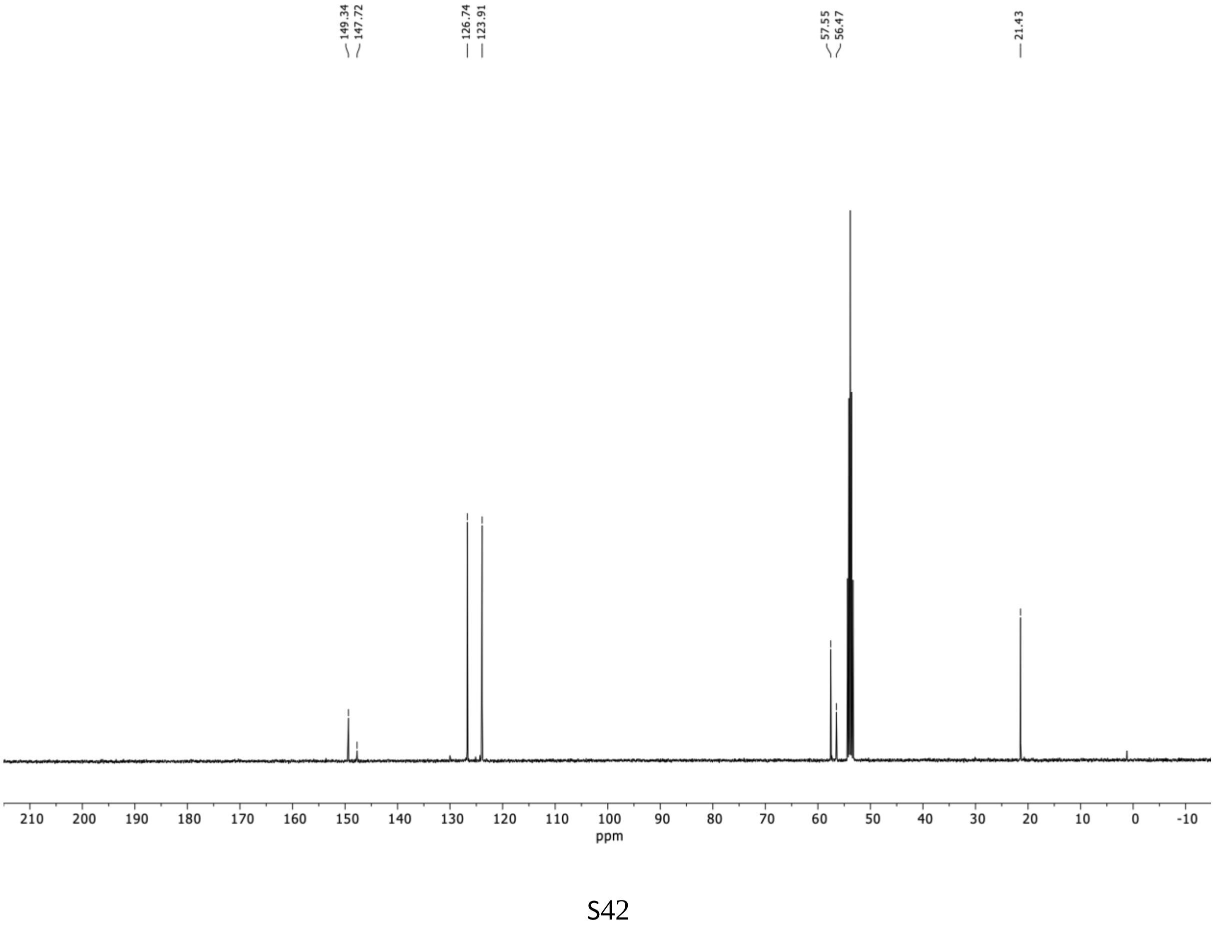

2-(4-Methylbenzoate)-2-methyloxirane

^{1}H-NMR (400 MHz, CD_2Cl_2)

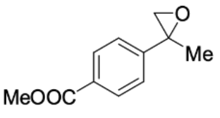

1q

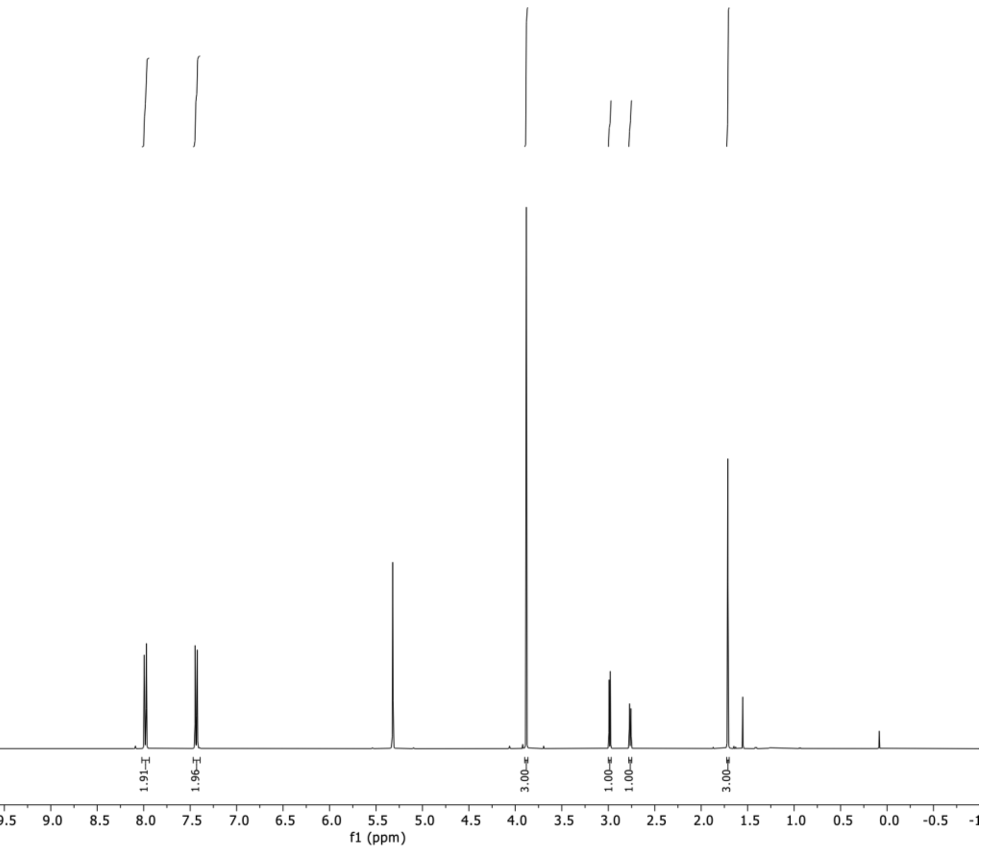

^{13}C-NMR (101 MHz, CD_2Cl_2)

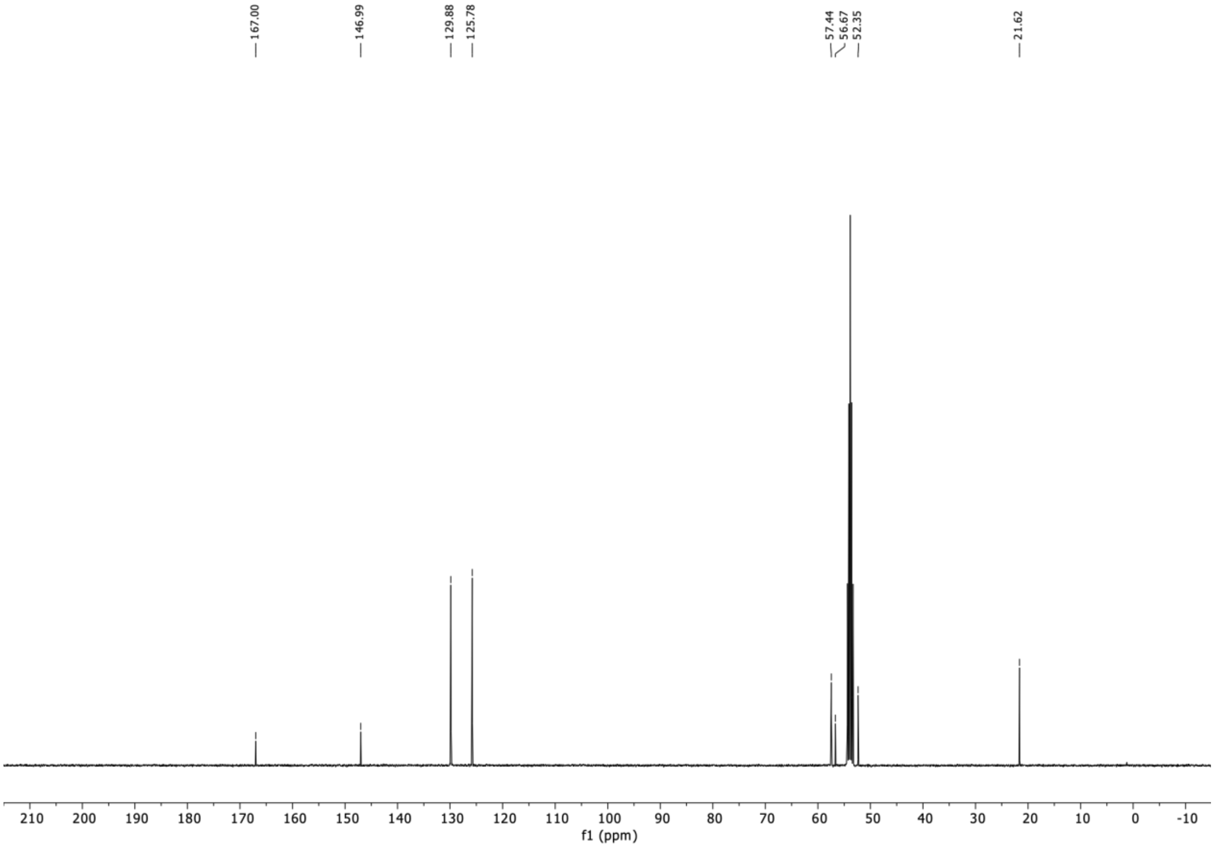

2-Methyl-2-(naphthalene-2-yl)oxirane

^{1}H-NMR (400 MHz, CD_2Cl_2)

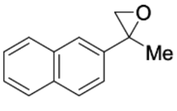

1r

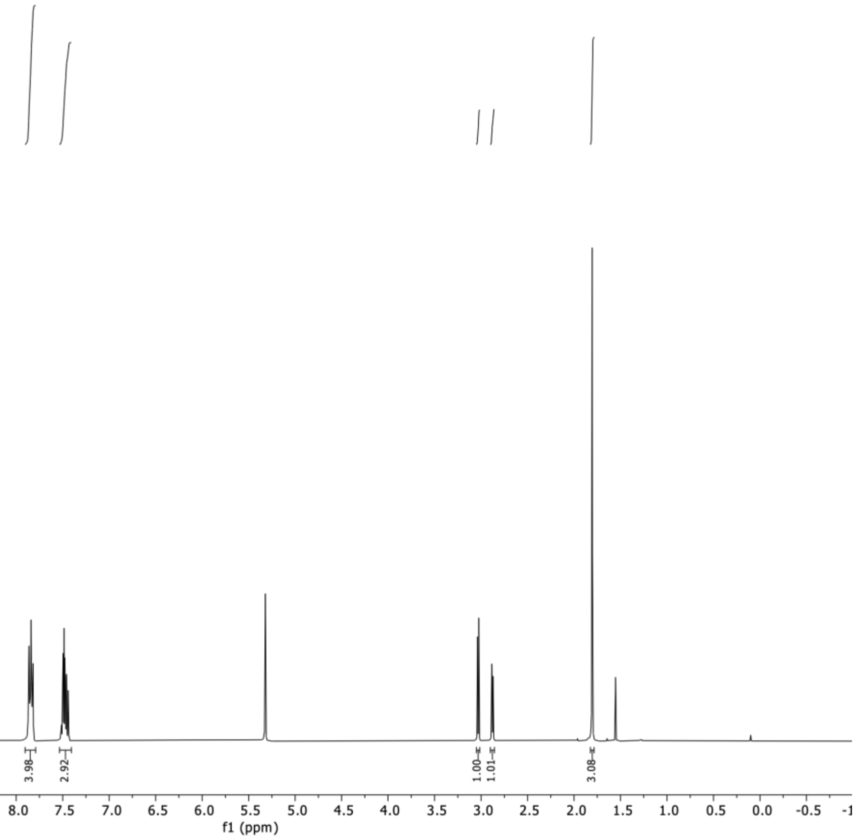

^{13}C-NMR (101 MHz, CD_2Cl_2)

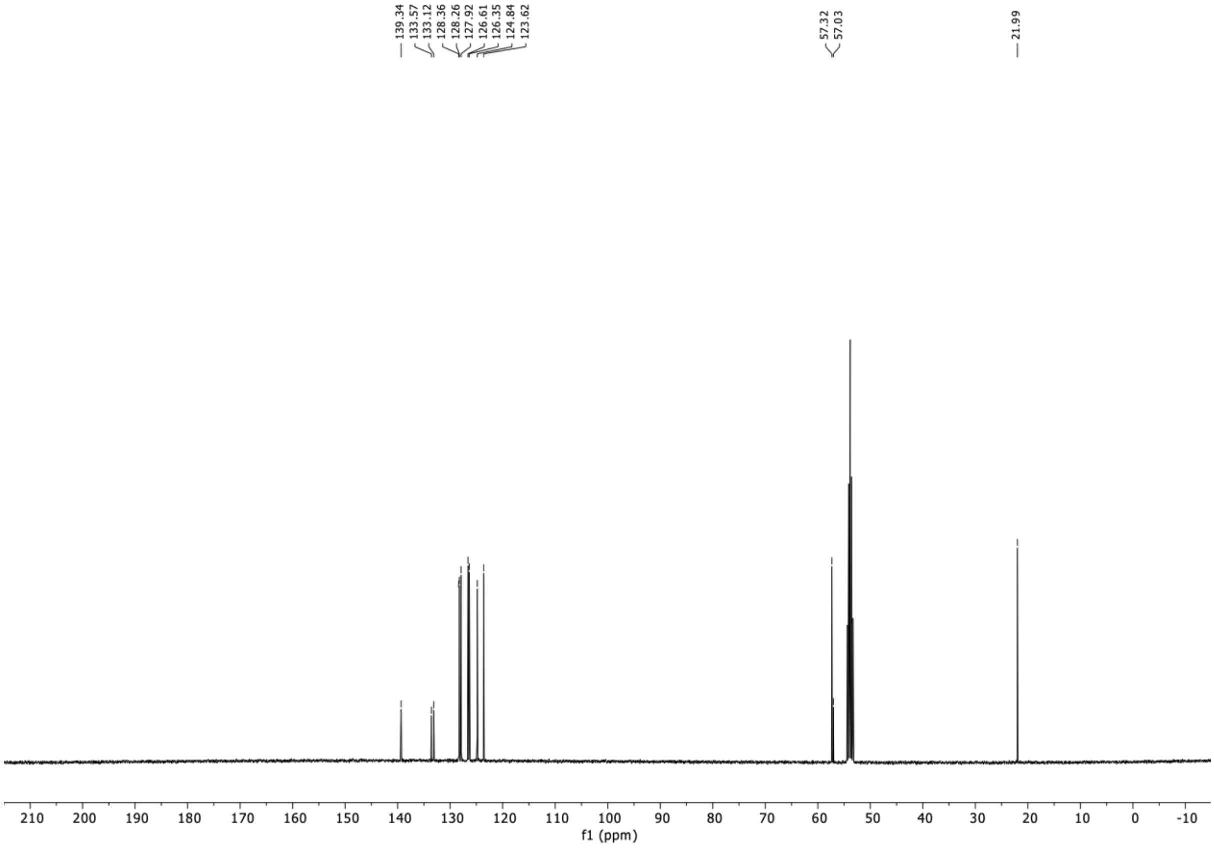

2-Methyl-2-(naphthalene-1-yl)oxirane

^{1}H-NMR (400 MHz, CD_2Cl_2)

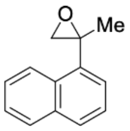

1s

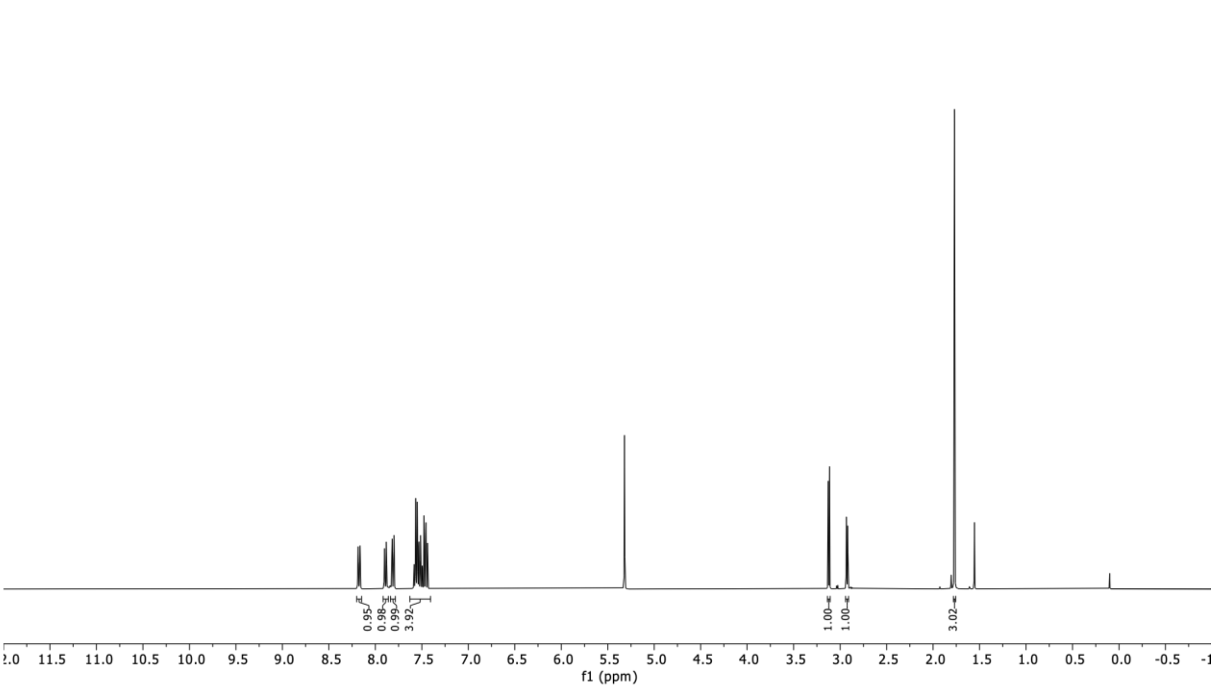

^{13}C-NMR (101 MHz, CD_2Cl_2)

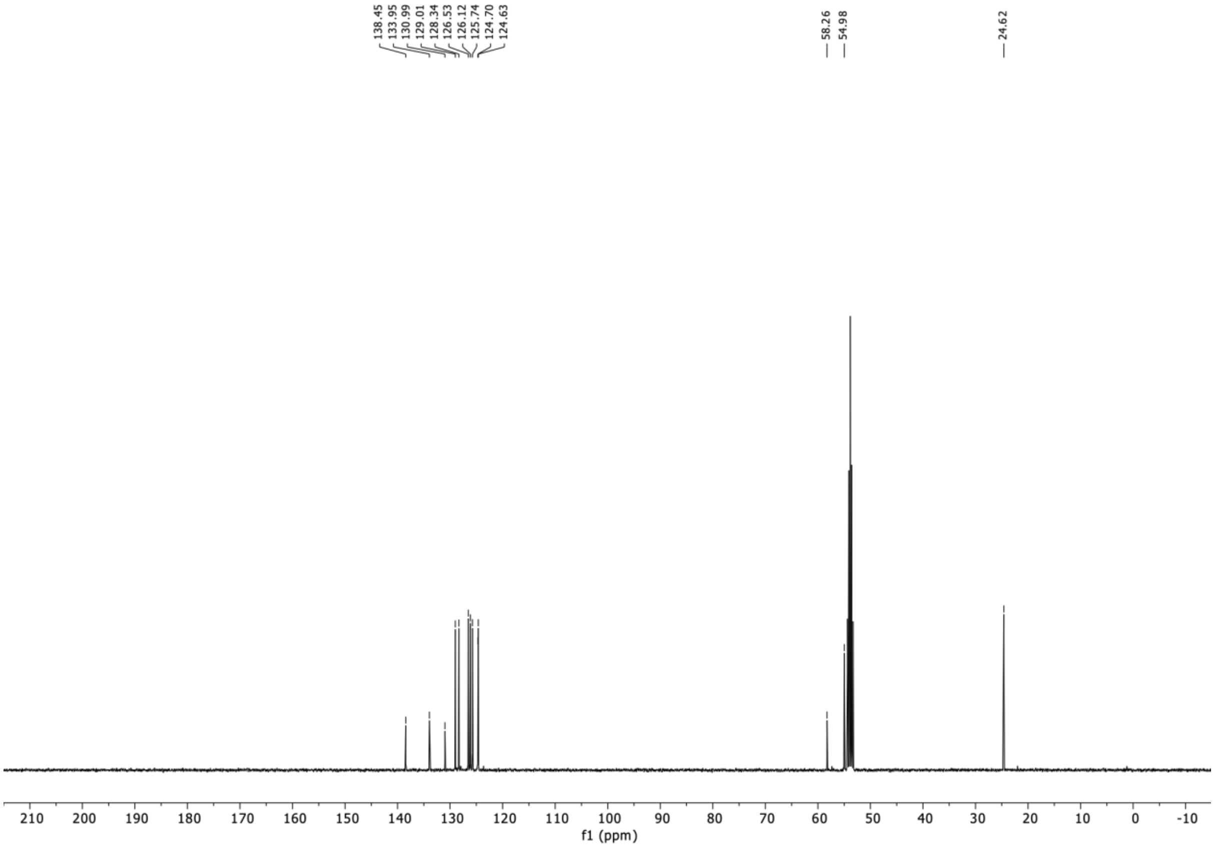

2-(4-*iso*-Butylphenyl)-2-methyloxirane

^{1}H-NMR (400 MHz, CD_2Cl_2)

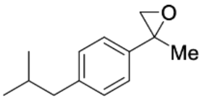

1t

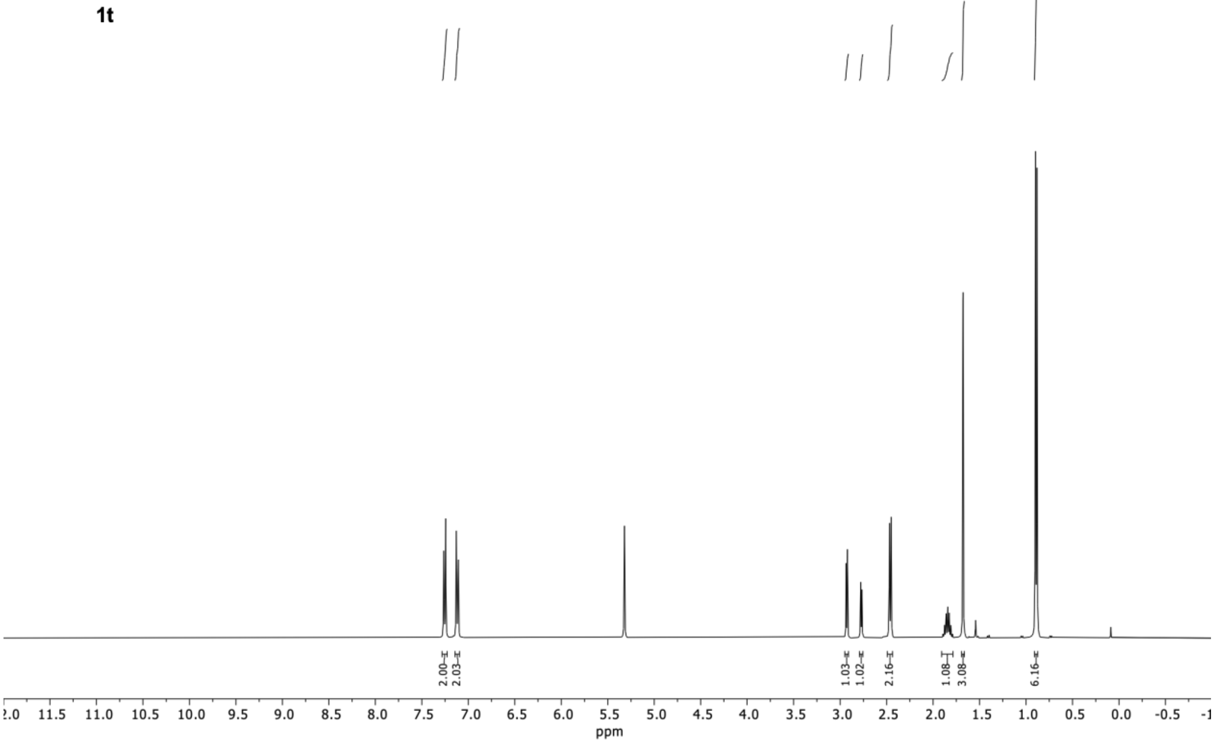

^{13}C-NMR (101 MHz, CD_2Cl_2)

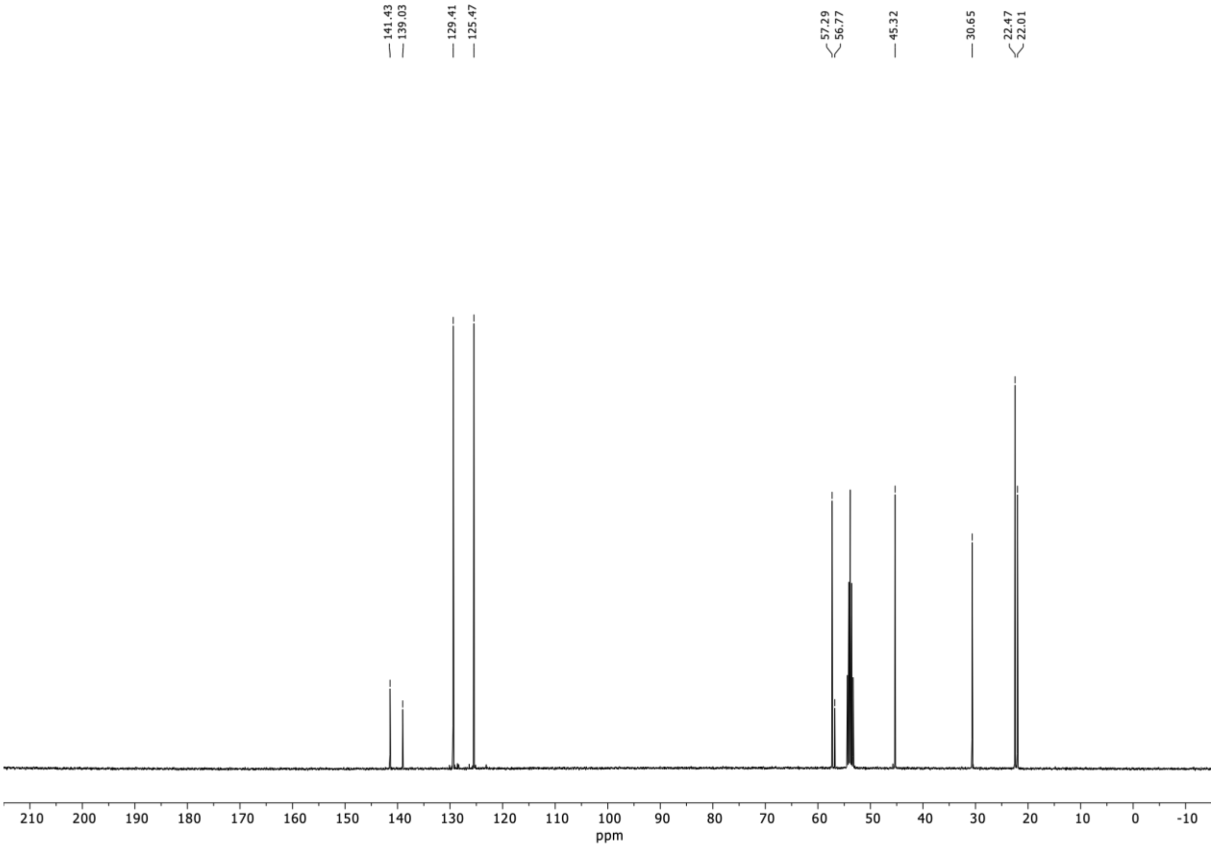

2-Methyl-2-pentyloxirane

^{1}H-NMR (400 MHz, $CDCl_3$)

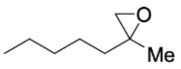

1u

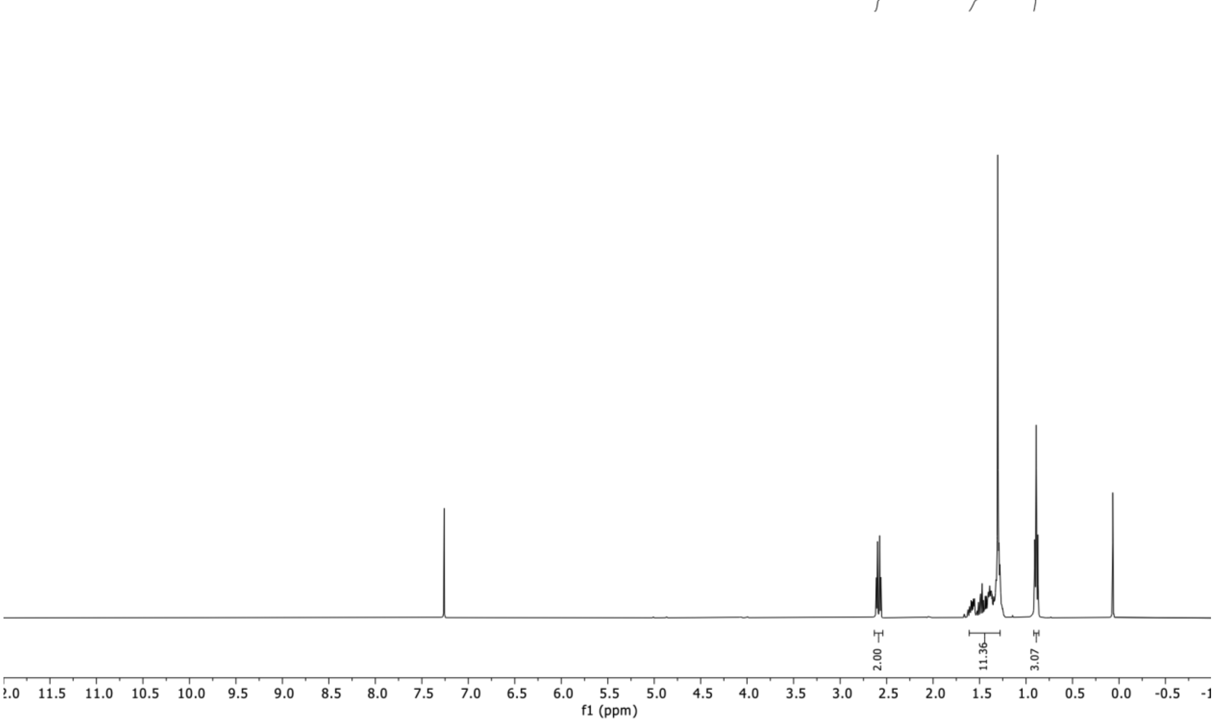

^{13}C-NMR (101 MHz, $CDCl_3$)

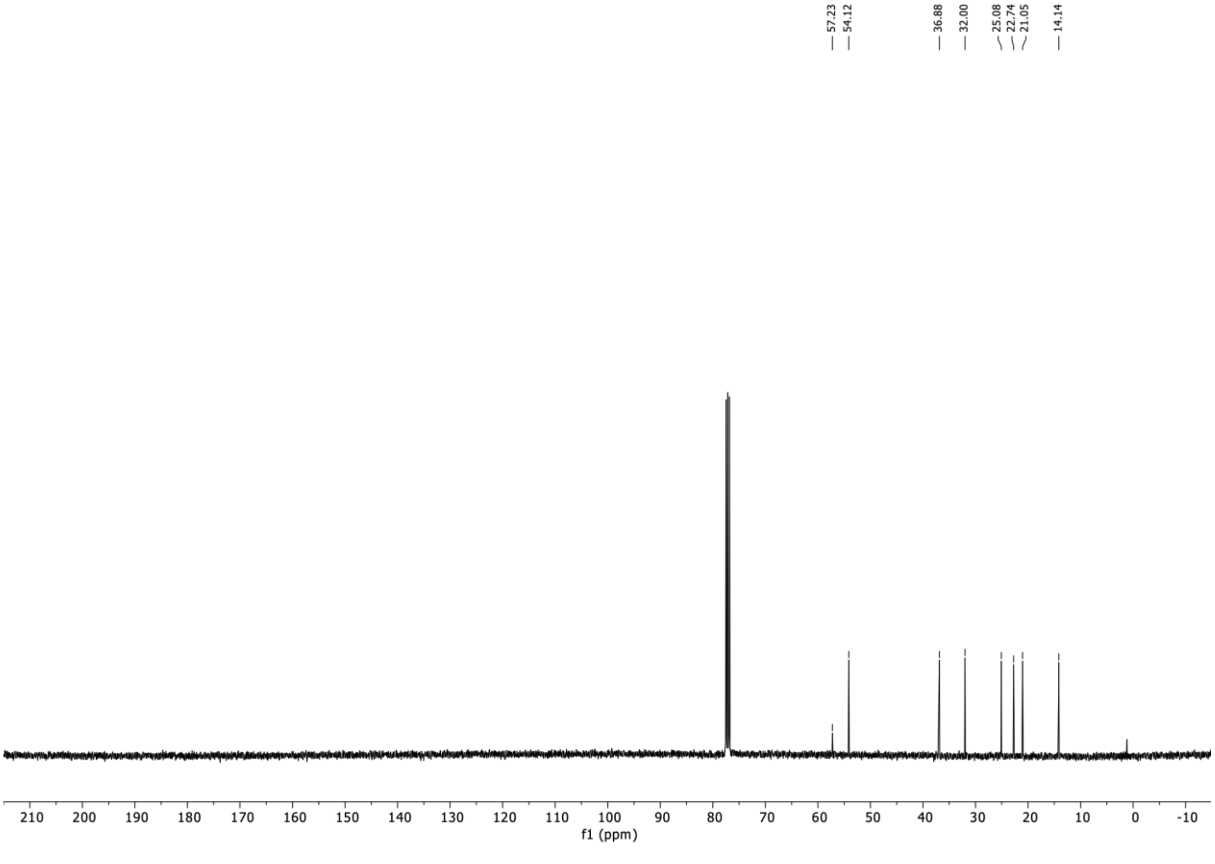

2-Cyclohexyl-2-methyloxirane

^{1}H-NMR (400 MHz, CD_2Cl_2)

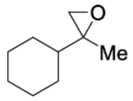

1v

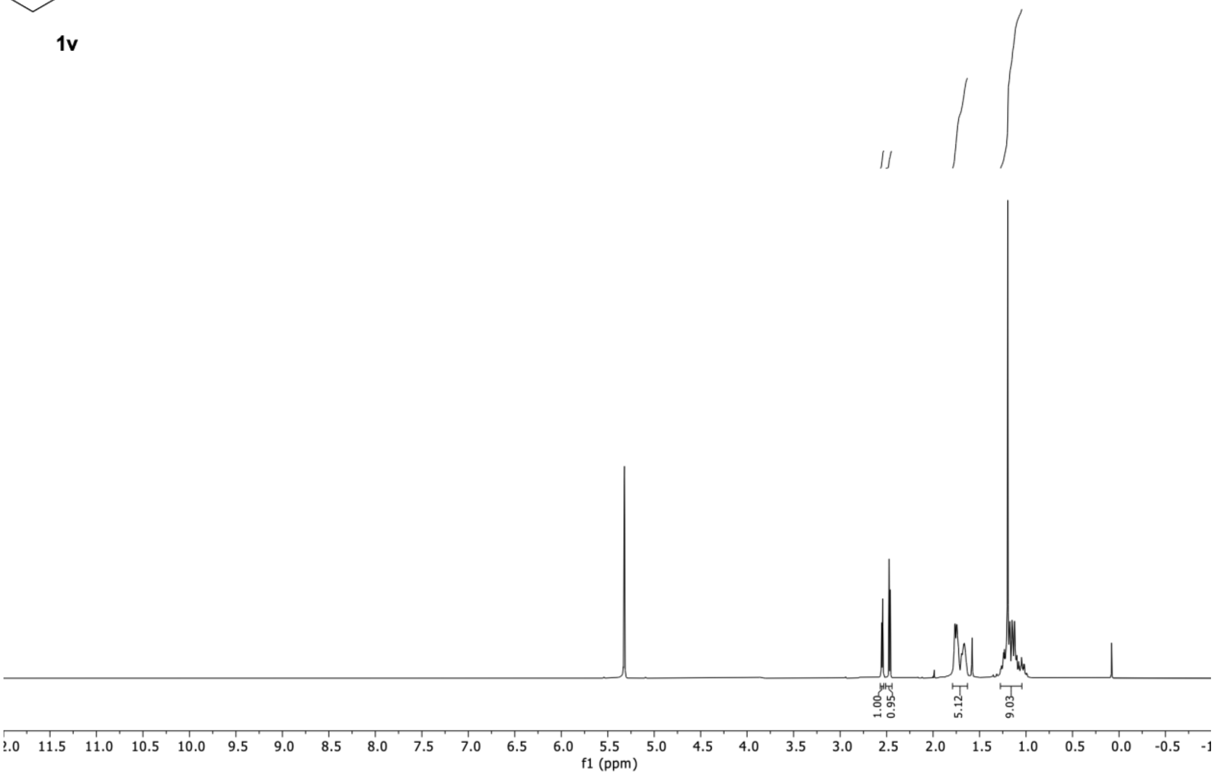

^{13}C-NMR (101 MHz, CD_2Cl_2)

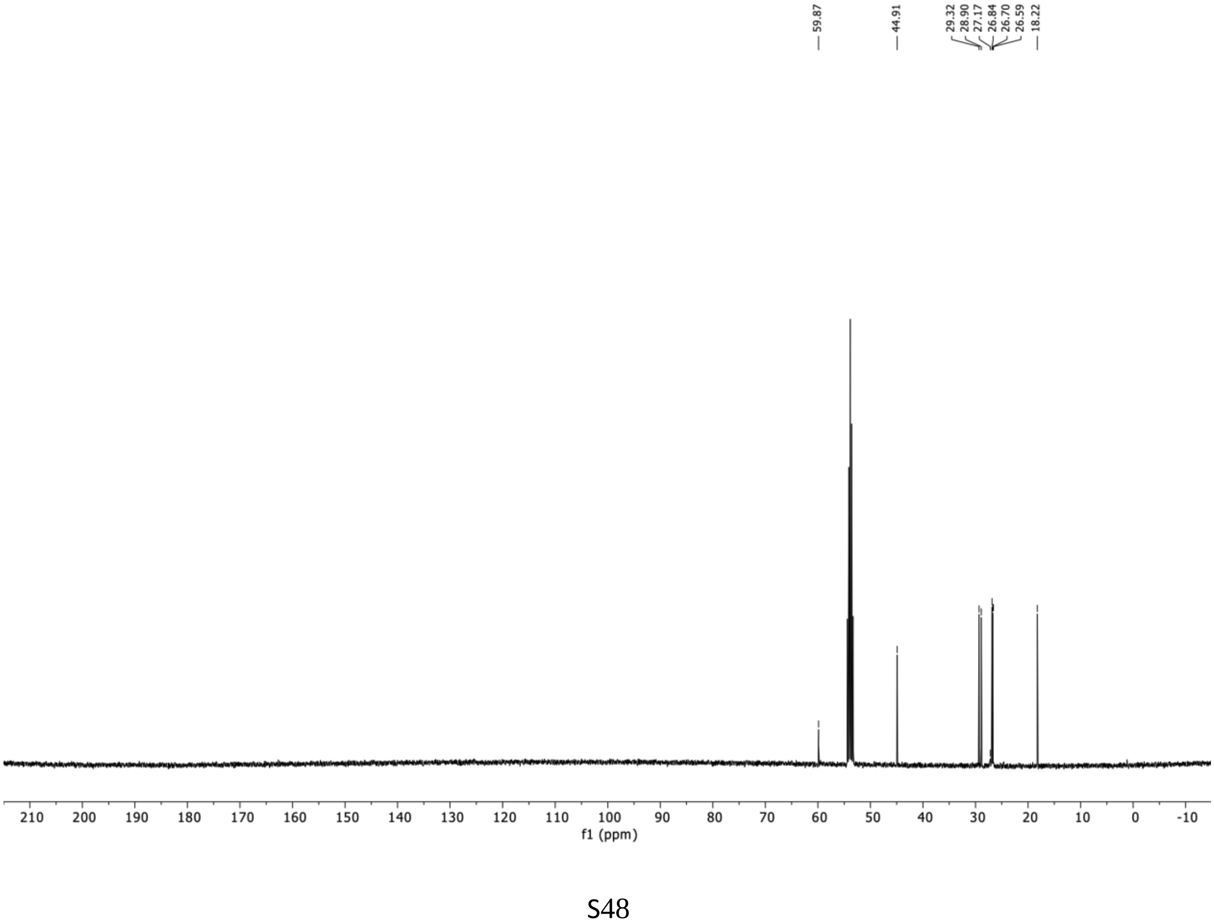

3-(2-Methyl-2-oxiranyl)pyridine

^{1}H-NMR (400 MHz, CD_2Cl_2)

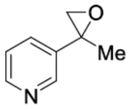

1w

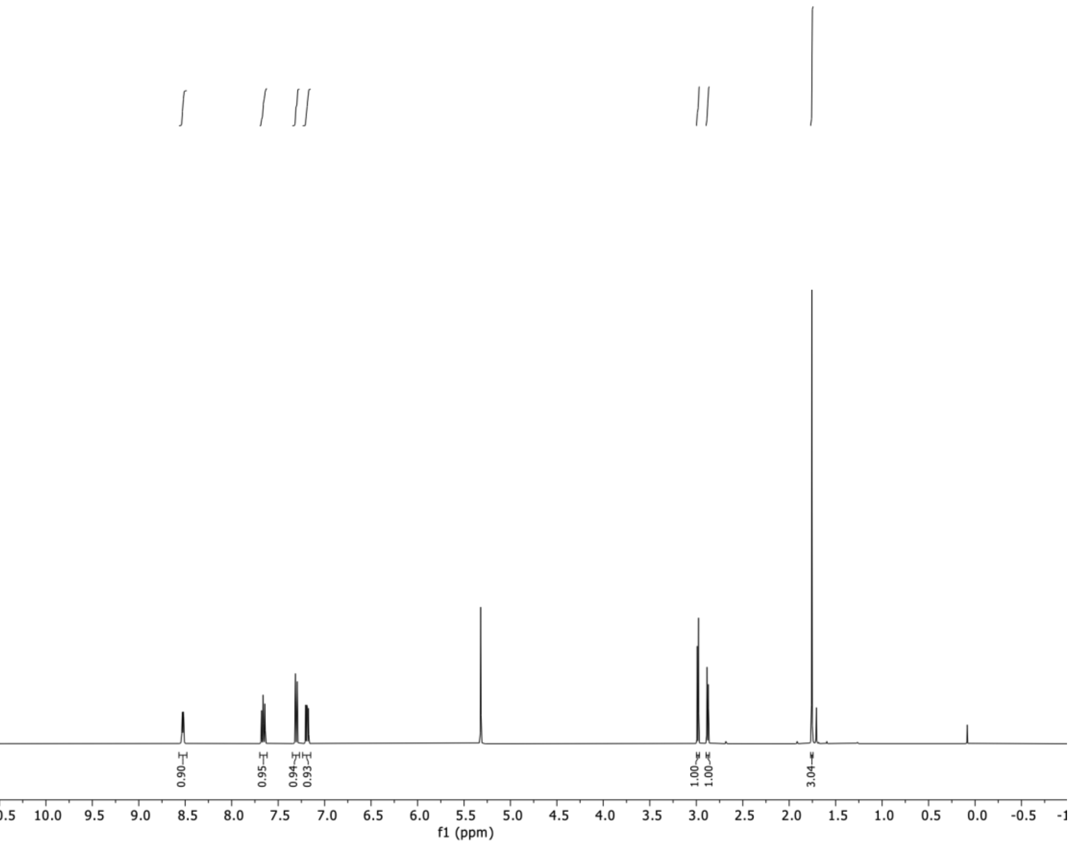

^{13}C-NMR (101 MHz, CD_2Cl_2)

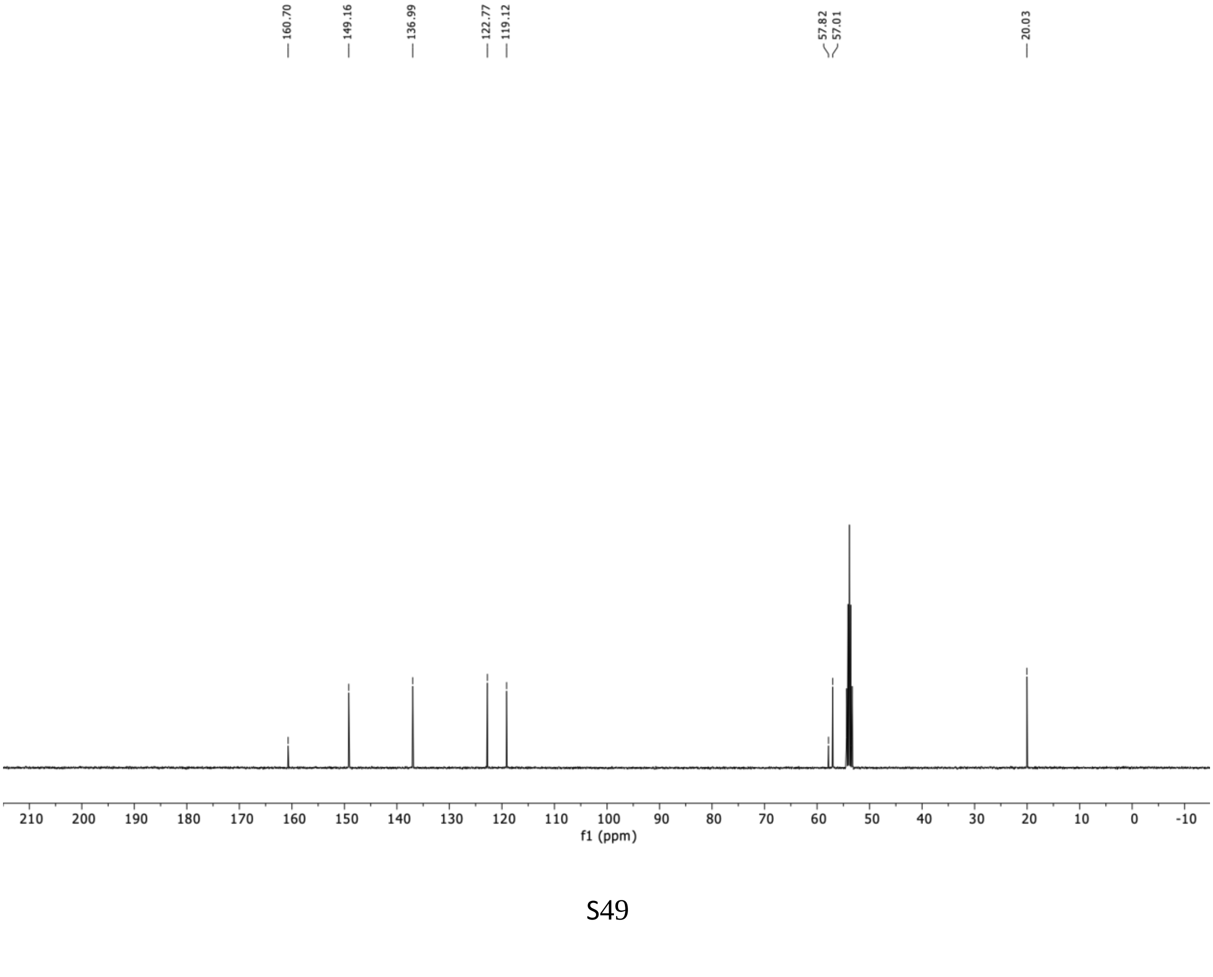

2-Phenylpropionaldehyde

^{1}H-NMR (400 MHz, $CDCl_3$)

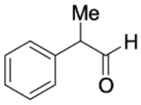

2a

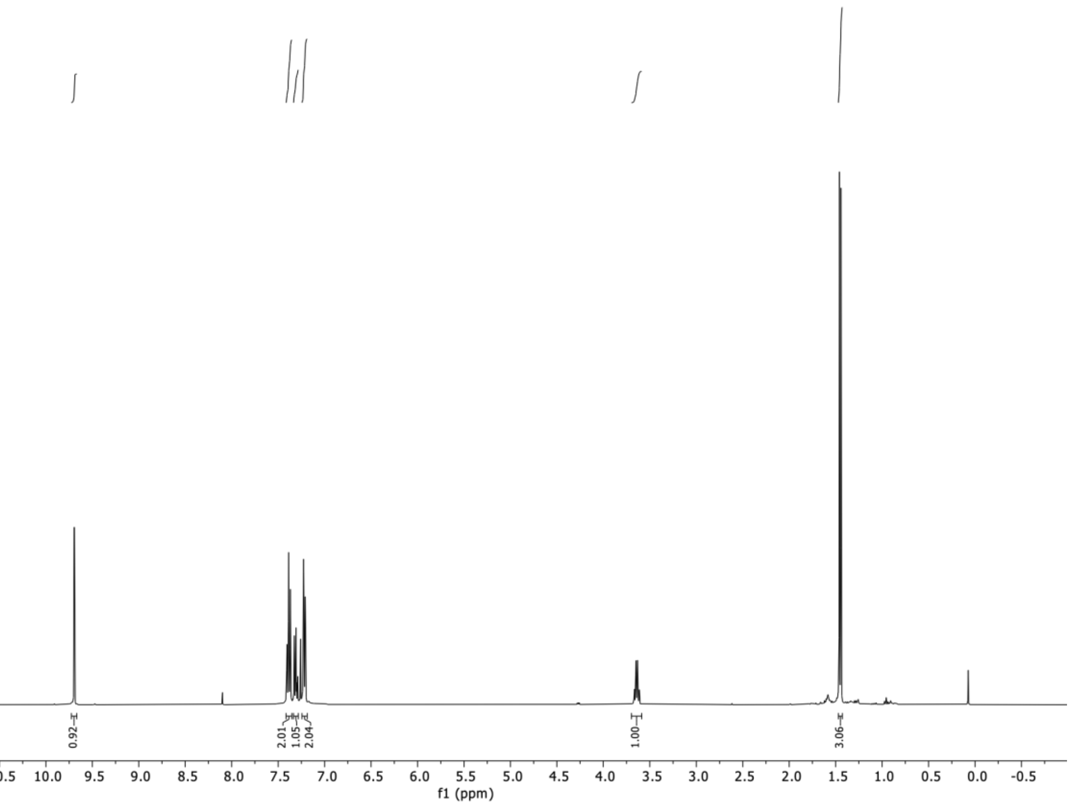

^{13}C-NMR (101 MHz, $CDCl_3$)

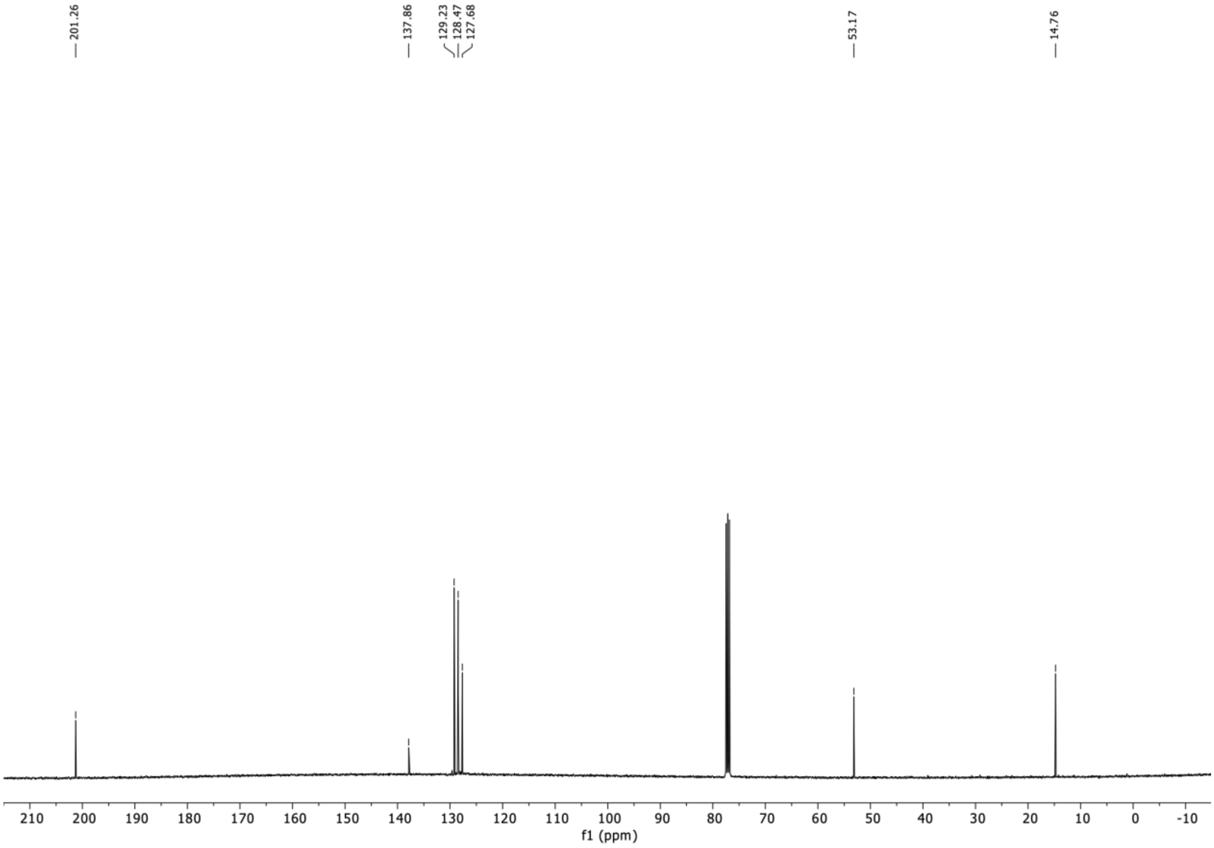

2-Phenylbutyraldehyde

^{1}H-NMR (400 MHz, $CDCl_3$)

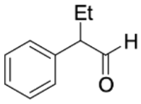

2b

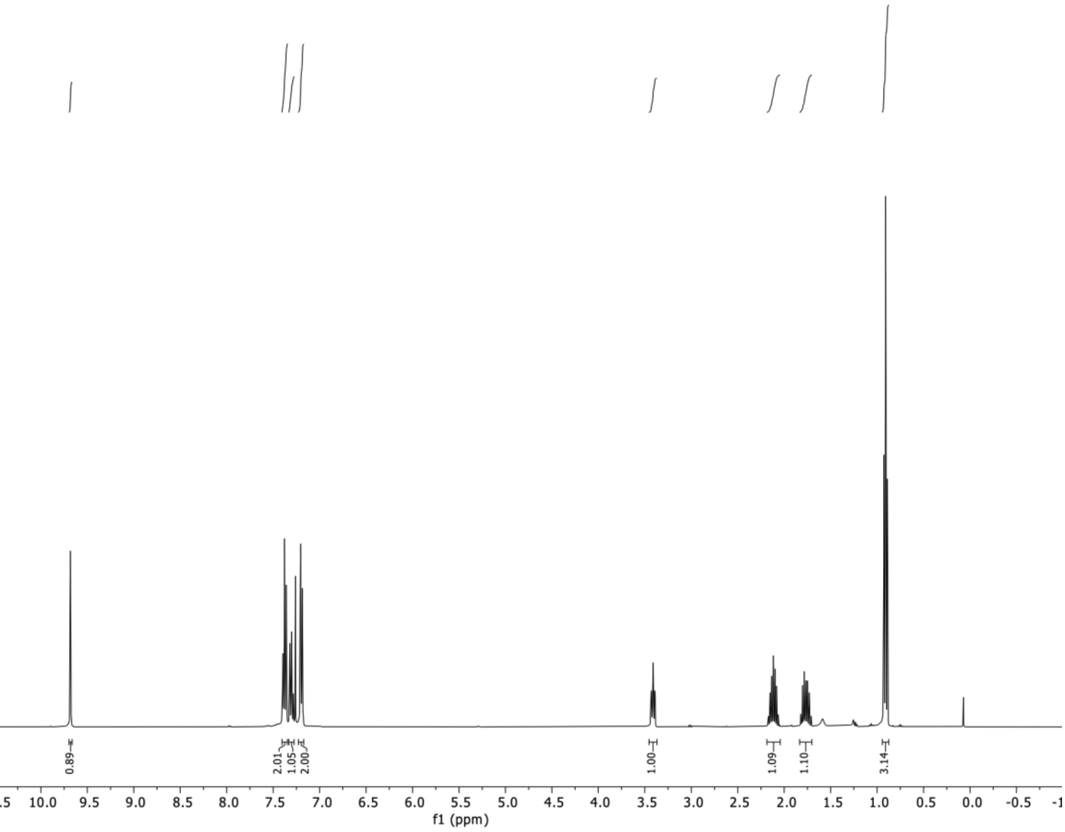

^{13}C-NMR (101 MHz, $CDCl_3$)

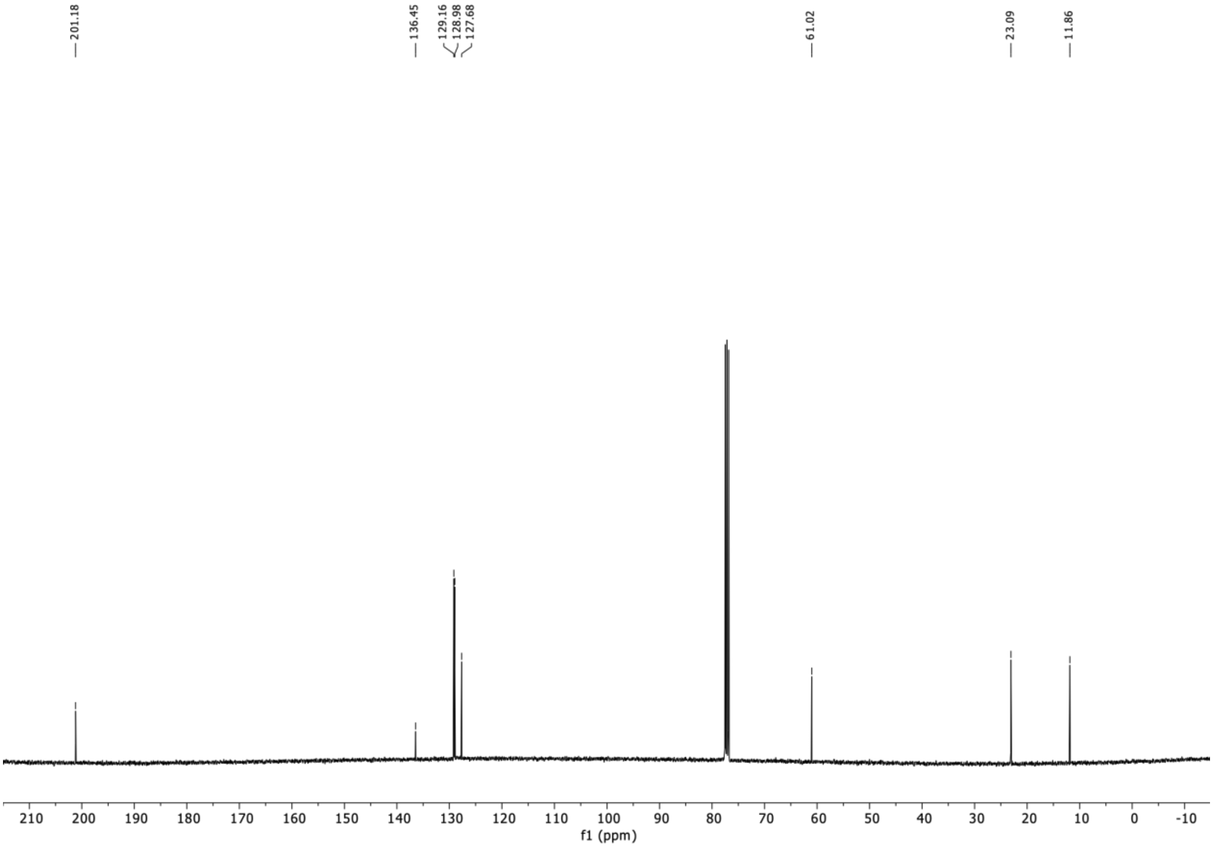

2-Phenylvaleraldehyde

^{1}H-NMR (400 MHz, $CDCl_3$)

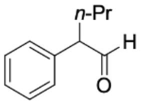

2c

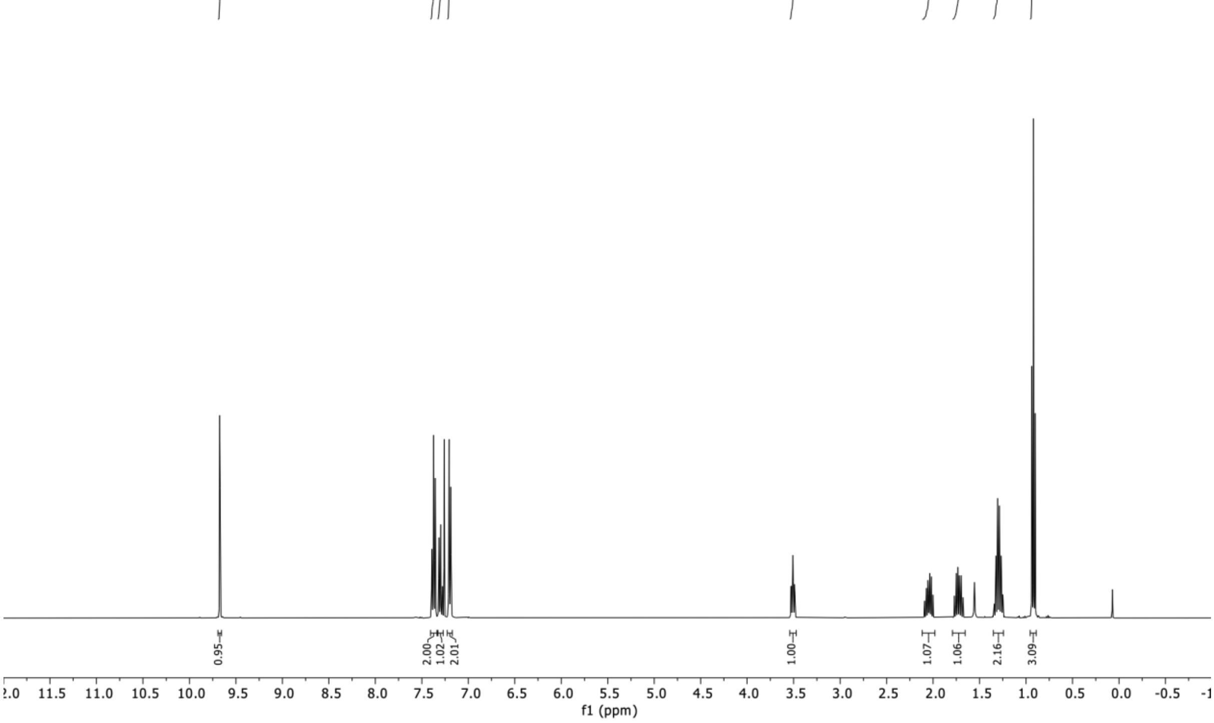

^{13}C-NMR (101 MHz, $CDCl_3$)

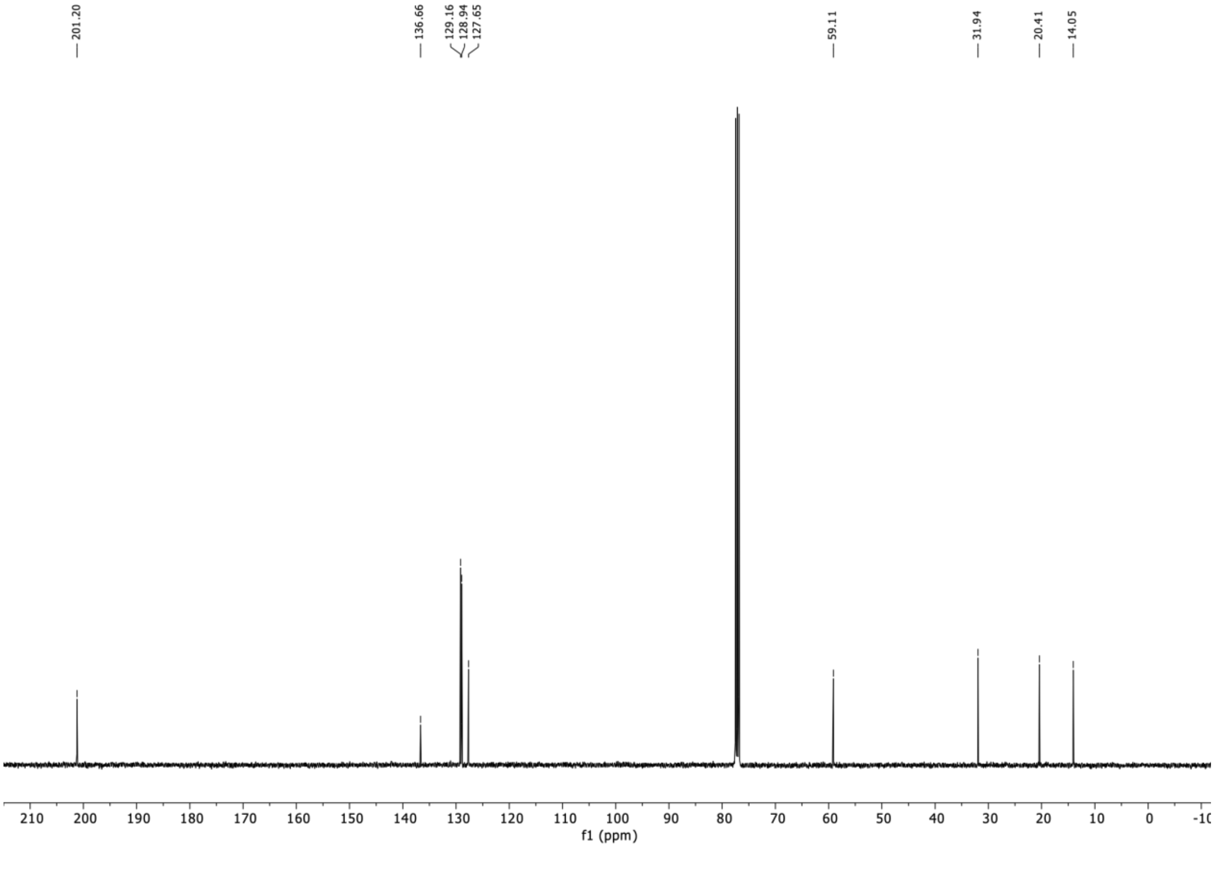

2-Cyclohexyl-2-phenylacetaldehyde

^{1}H-NMR (400 MHz, $CDCl_3$)

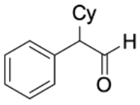

2d

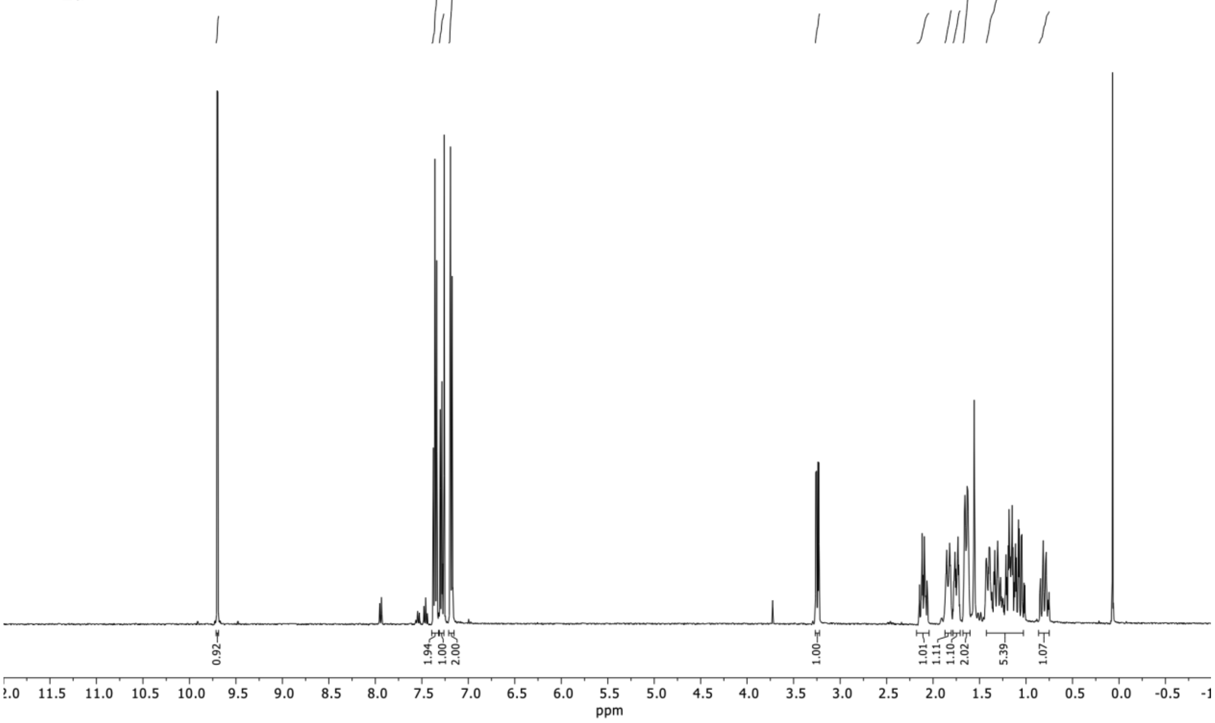

^{13}C-NMR (101 MHz, $CDCl_3$)

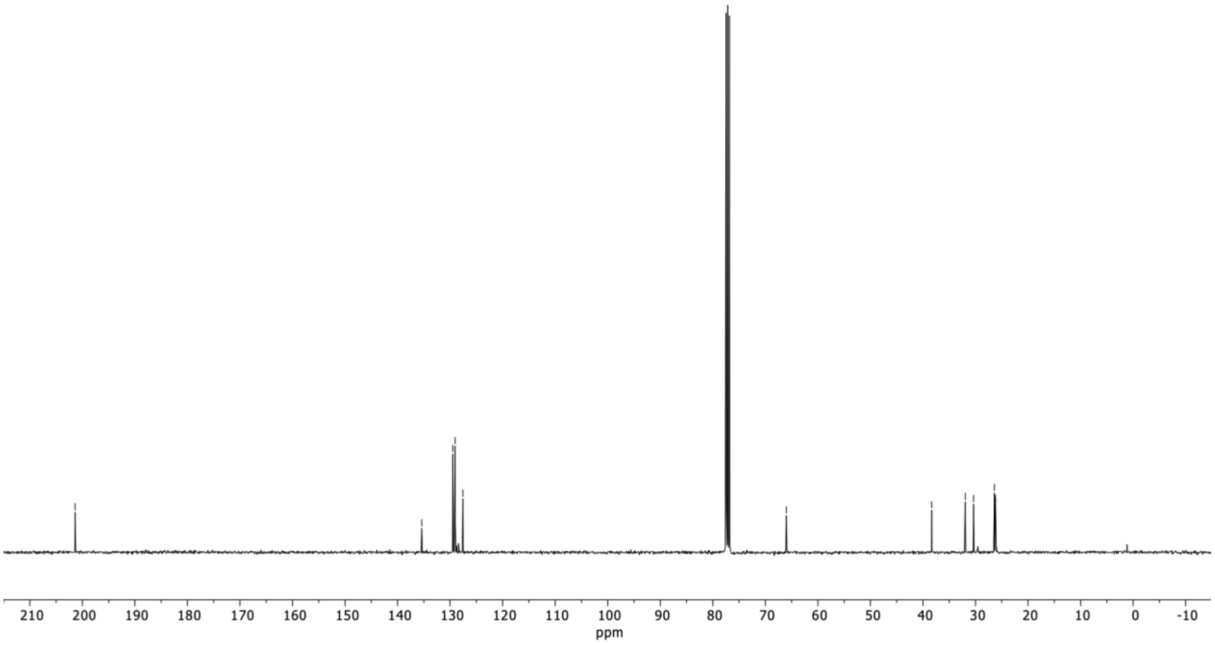

2-(4-Methylphenyl)-propionaldehyde

^{1}H-NMR (400 MHz, $CDCl_3$)

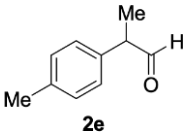

2e

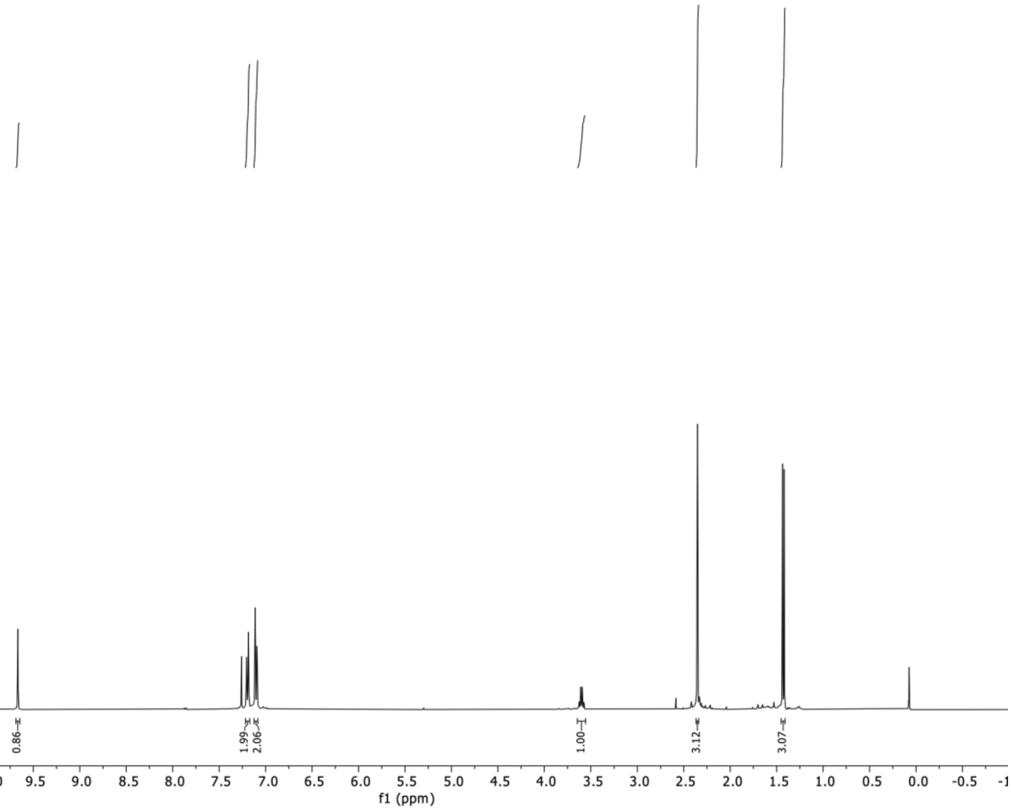

^{13}C-NMR (101 MHz, $CDCl_3$)

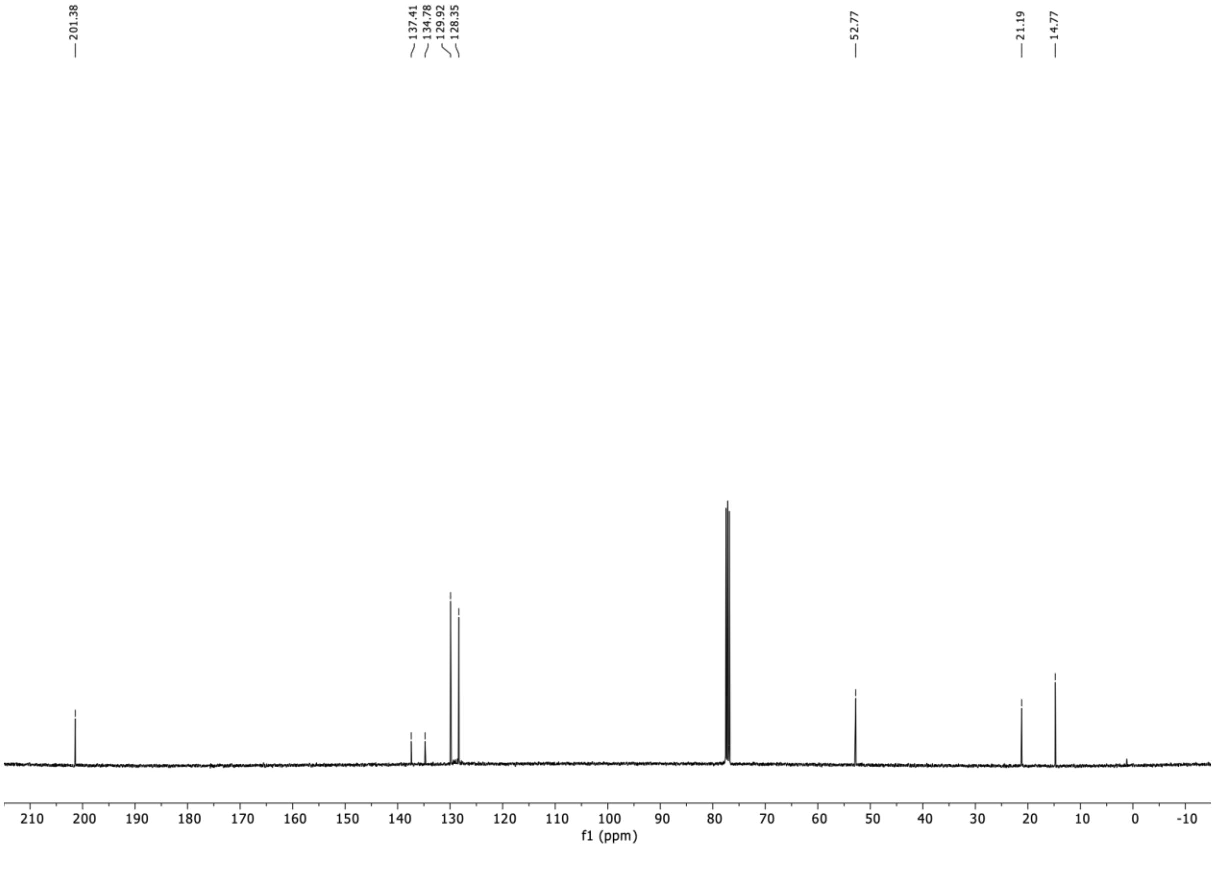

2-(3-Methylphenyl)-propionaldehyde

^{1}H-NMR (400 MHz, $CDCl_3$)

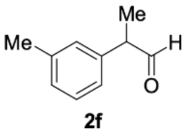

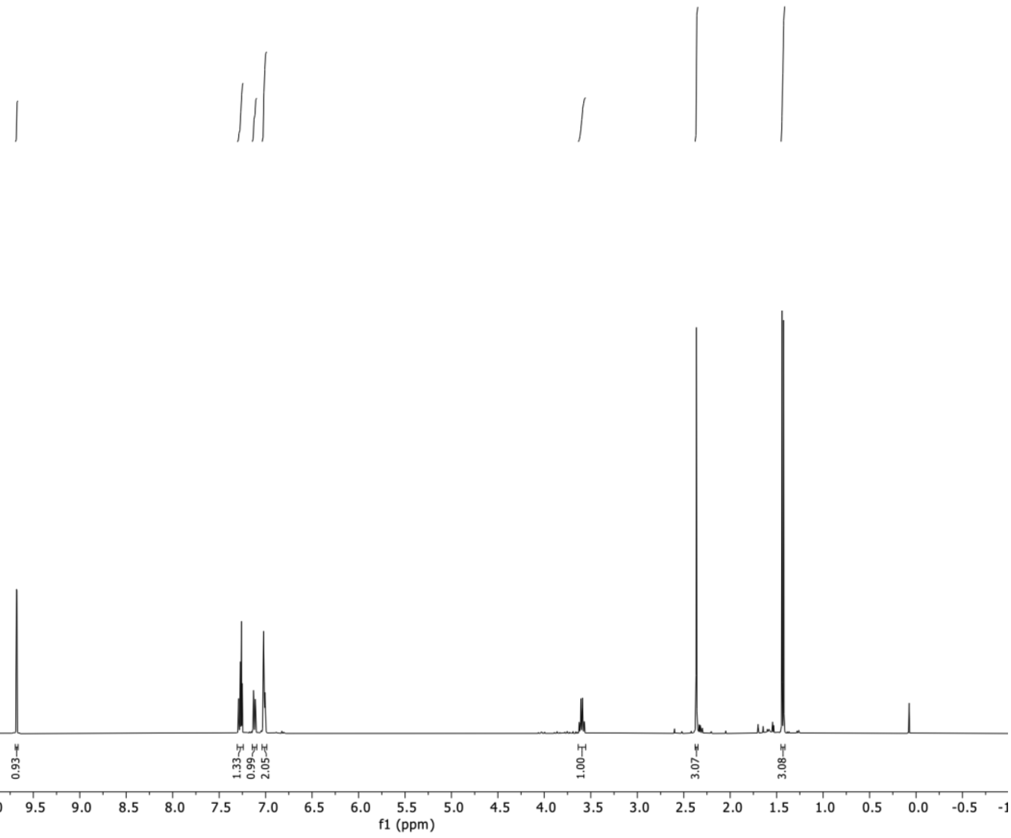

^{13}C-NMR (101 MHz, $CDCl_3$)

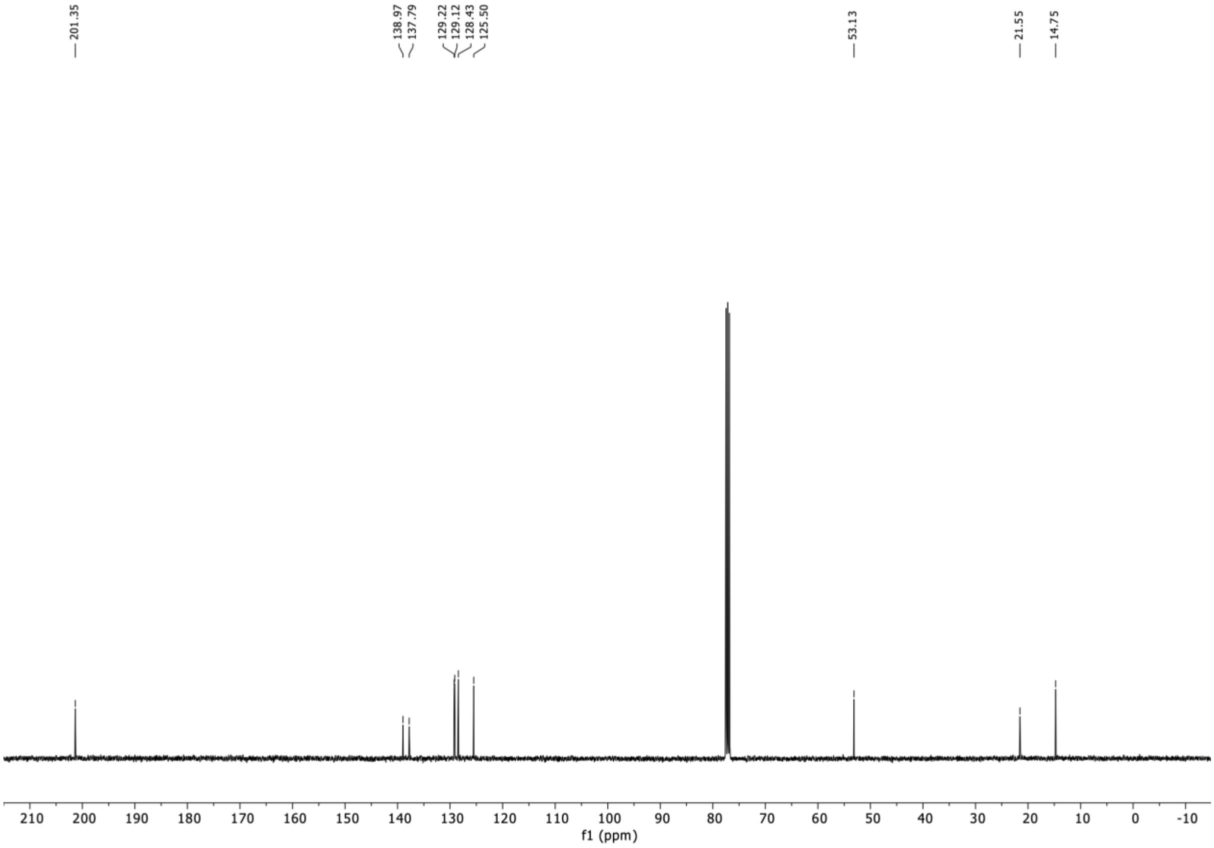

2-(2-Methylphenyl)-propionaldehyde

^{1}H-NMR (400 MHz, $CDCl_3$)

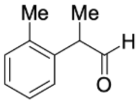

2g

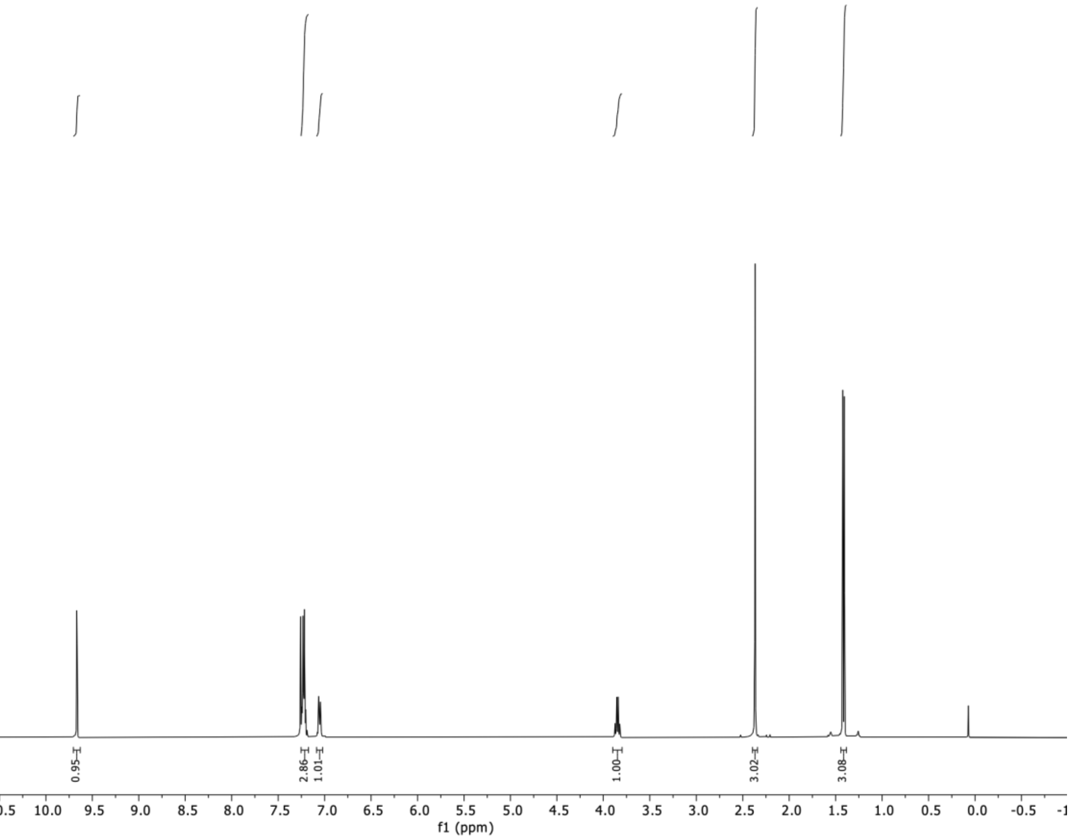

^{13}C-NMR (101 MHz, $CDCl_3$)

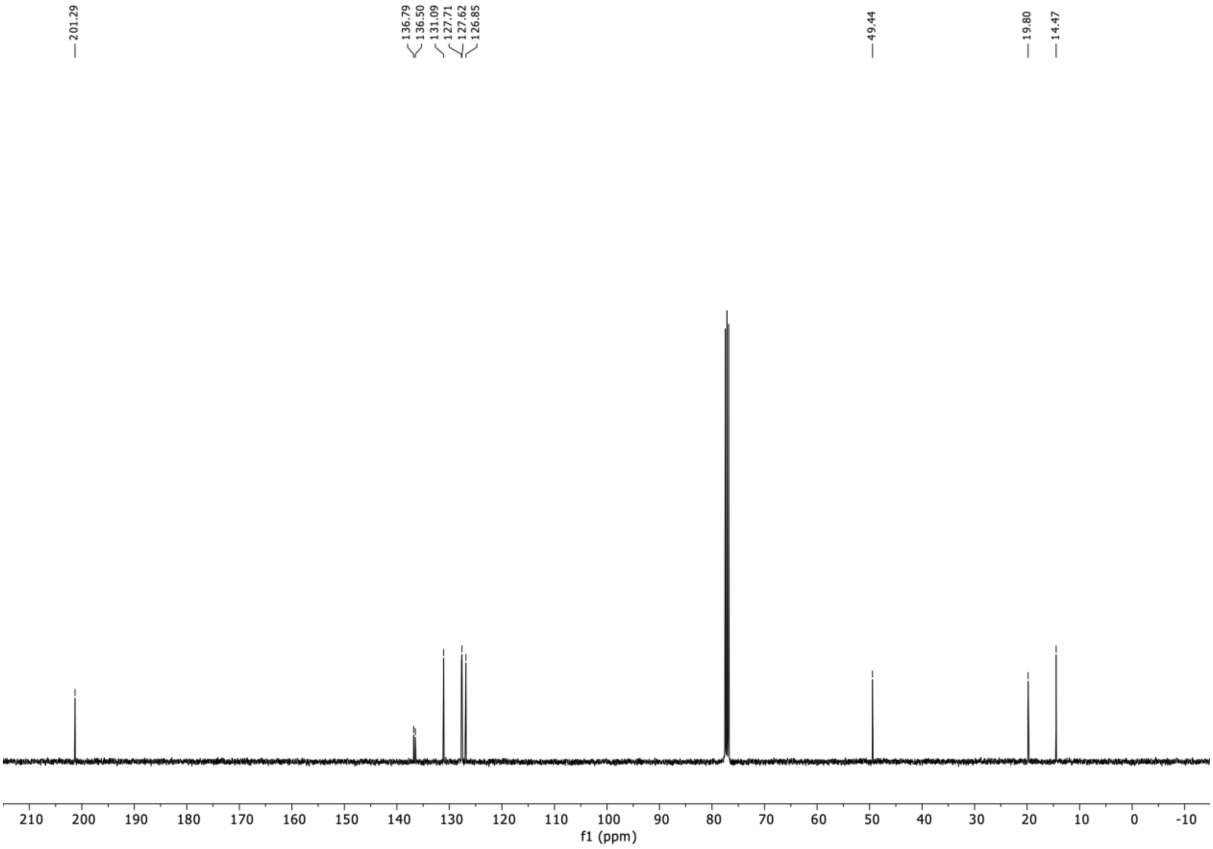

2-(4-Methoxyphenyl)-propionaldehyde

^{1}H-NMR (400 MHz, $CDCl_3$)

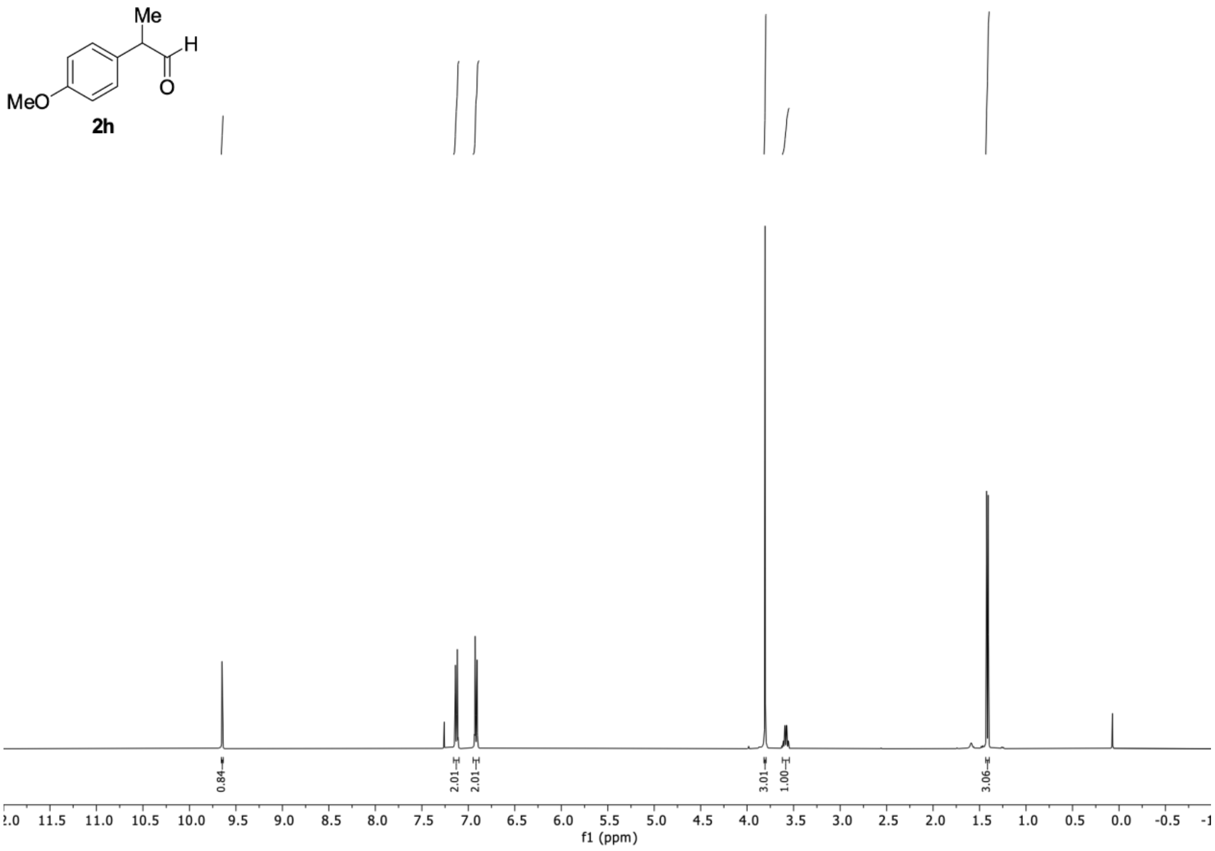

^{13}C-NMR (101 MHz, $CDCl_3$)

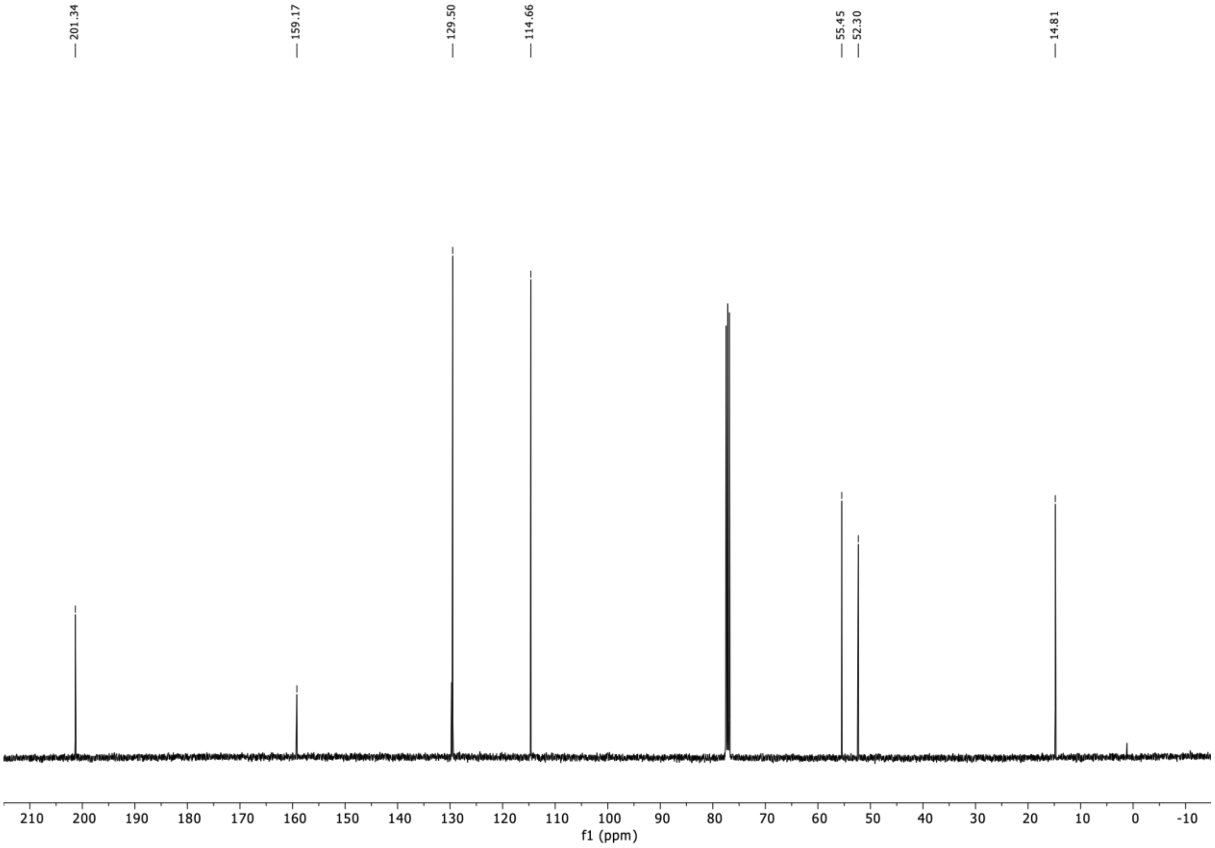

2-(4-Methylphenyl)-2-(phenyl)acetaldehyde

^{1}H-NMR (400 MHz, $CDCl_3$)

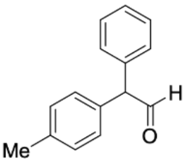

2i

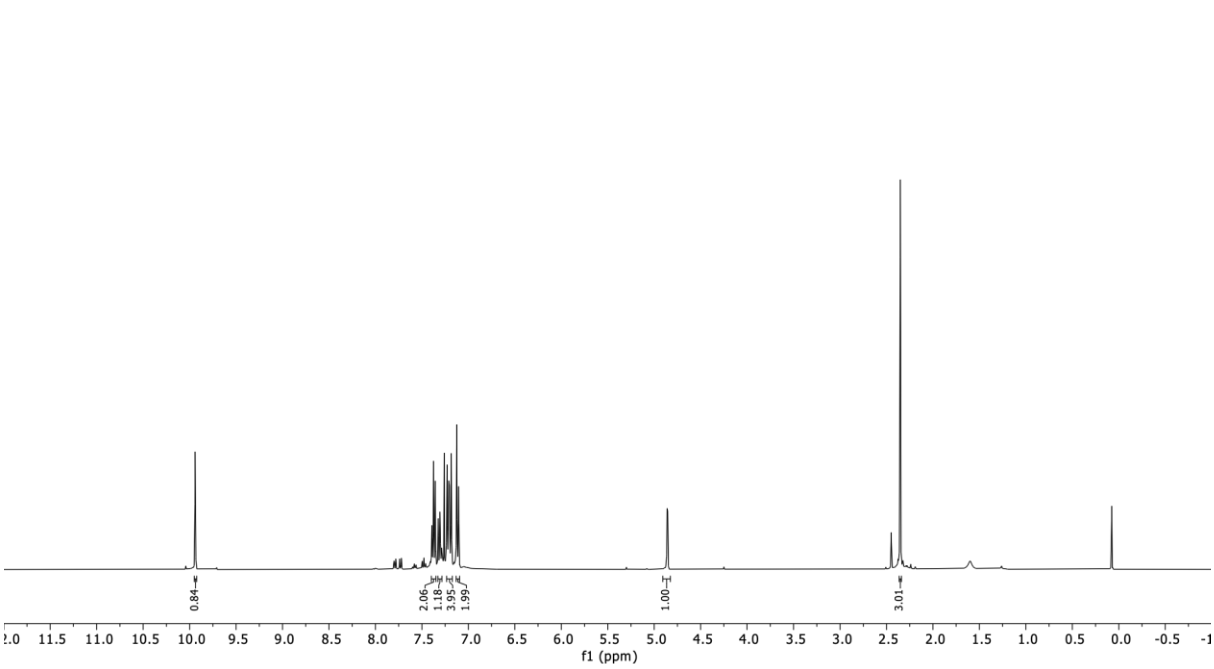

^{13}C-NMR (101 MHz, $CDCl_3$)

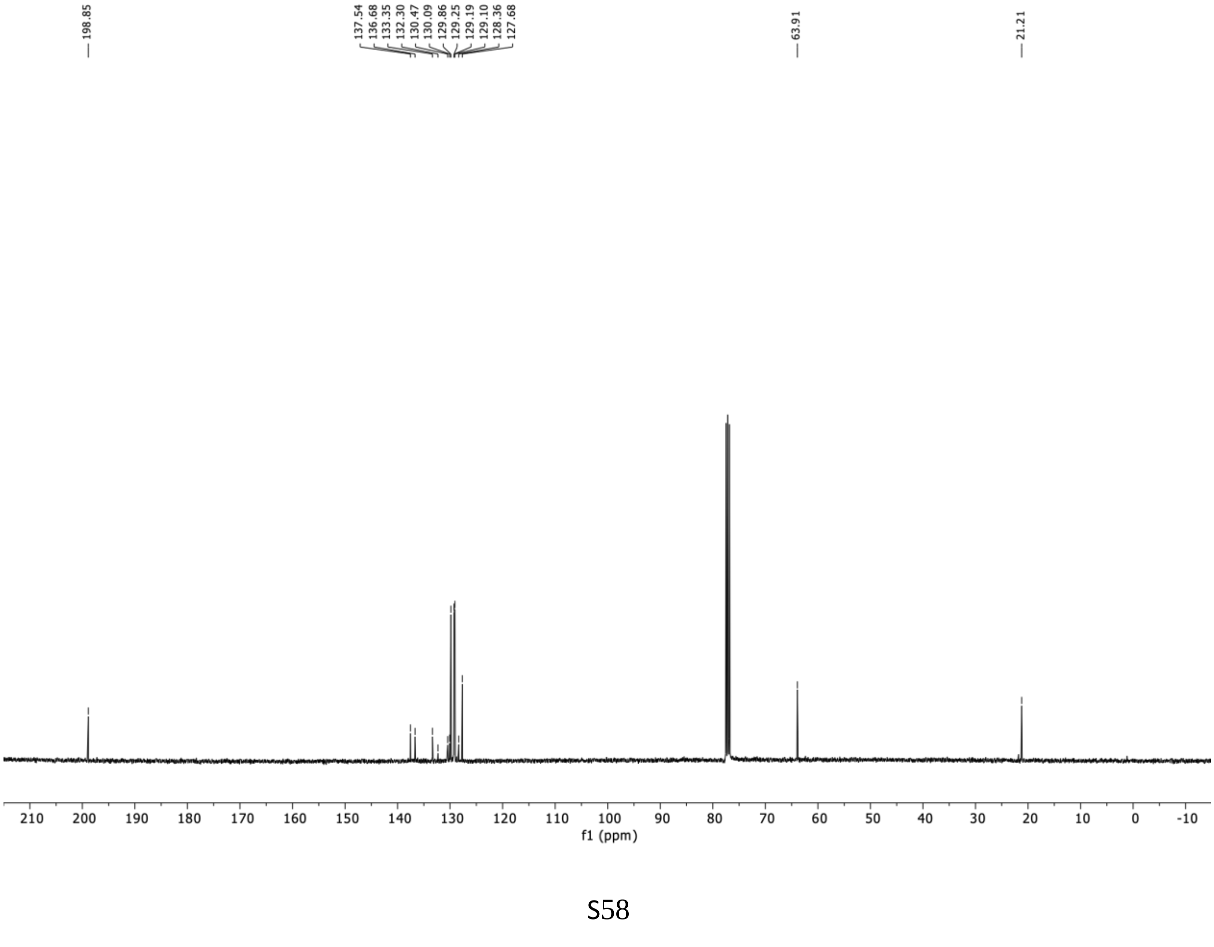

2-(4-Fluorophenyl)-propionaldehyde

^{1}H-NMR (400 MHz, $CDCl_3$)

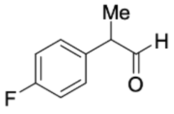

2j

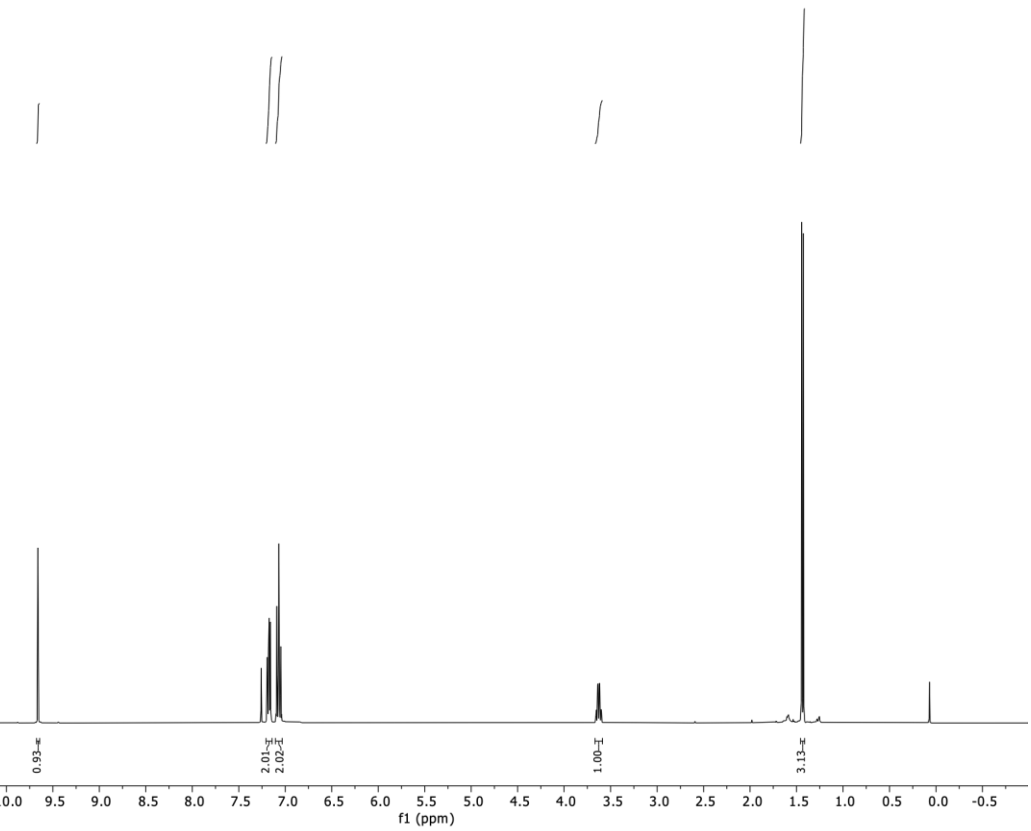

^{13}C-NMR (101 MHz, $CDCl_3$)

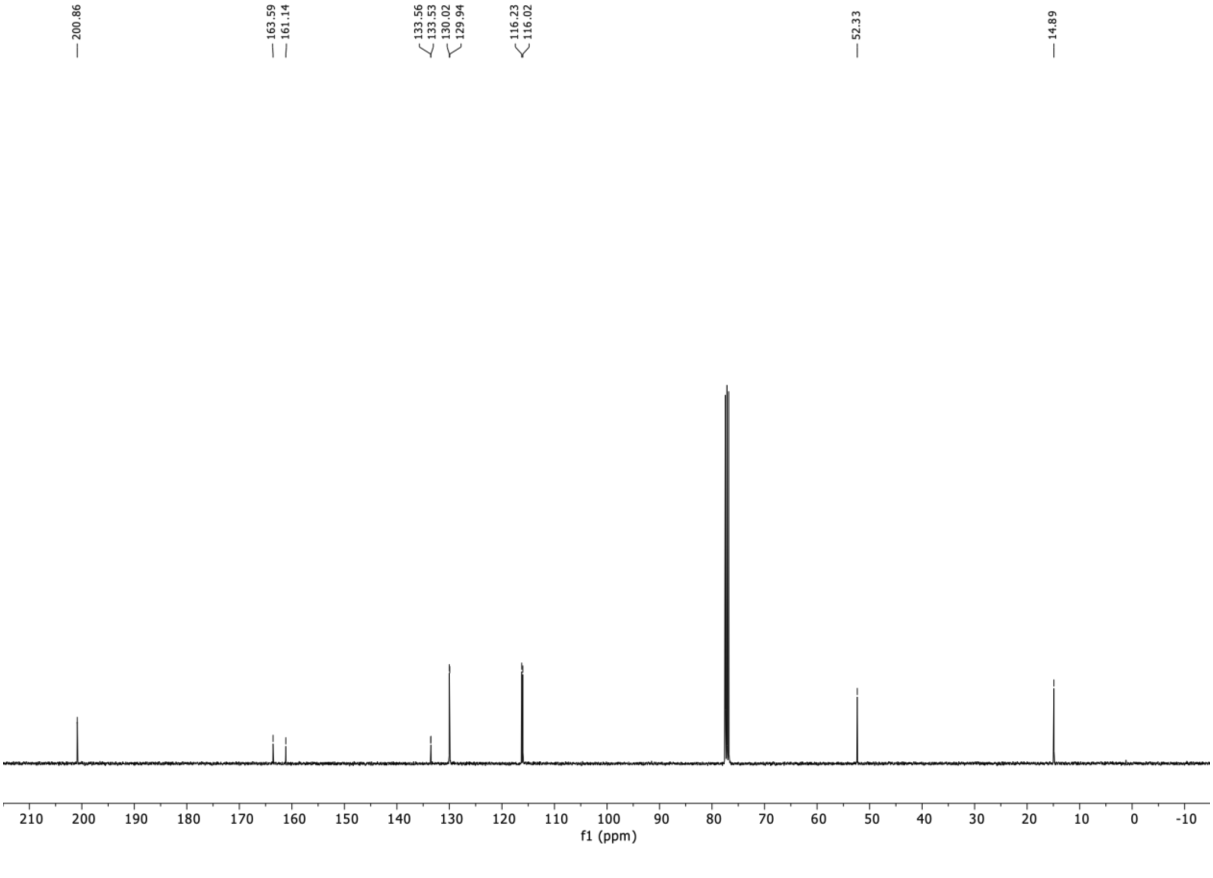

^{19}F-NMR (377 MHz, $CDCl_3$)

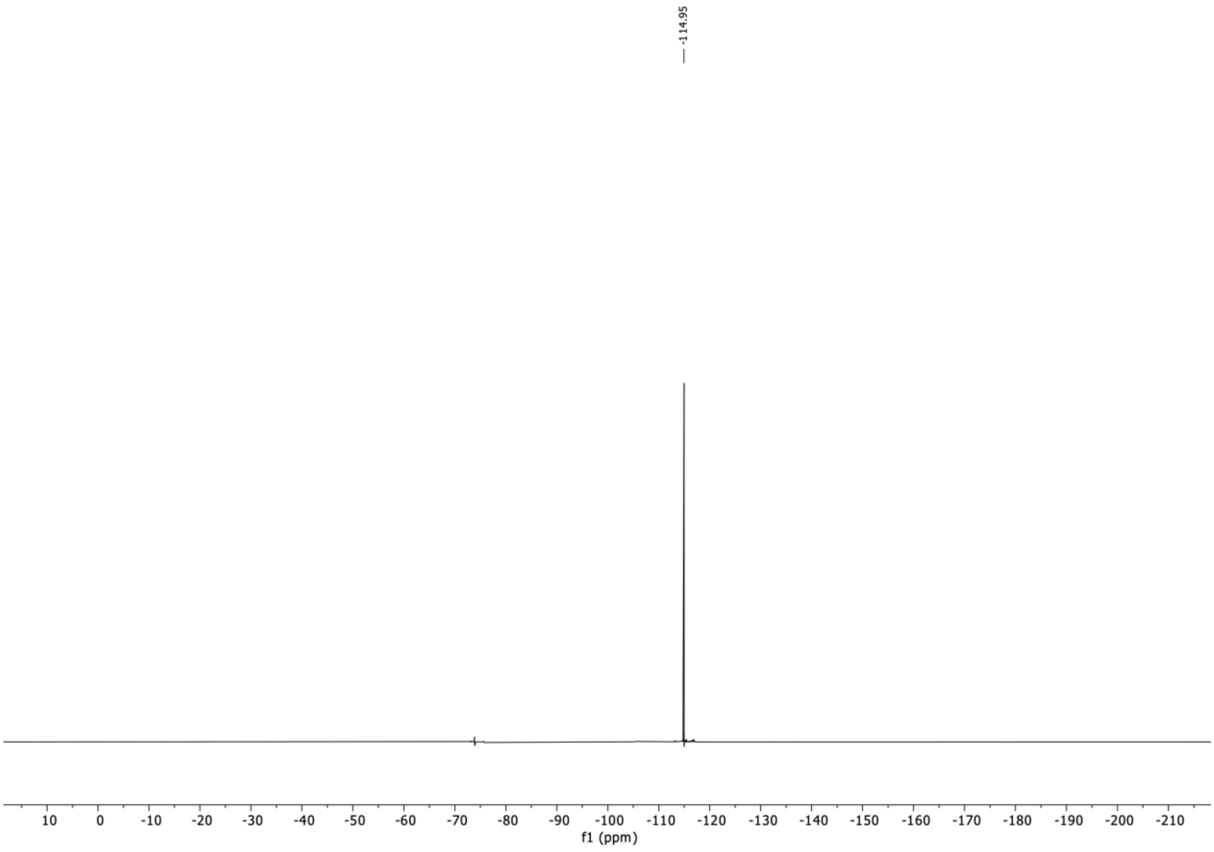

2-(4-Chlorophenyl)-propionaldehyde

^{1}H-NMR (400 MHz, $CDCl_3$)

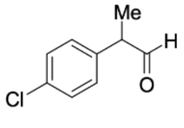

2k

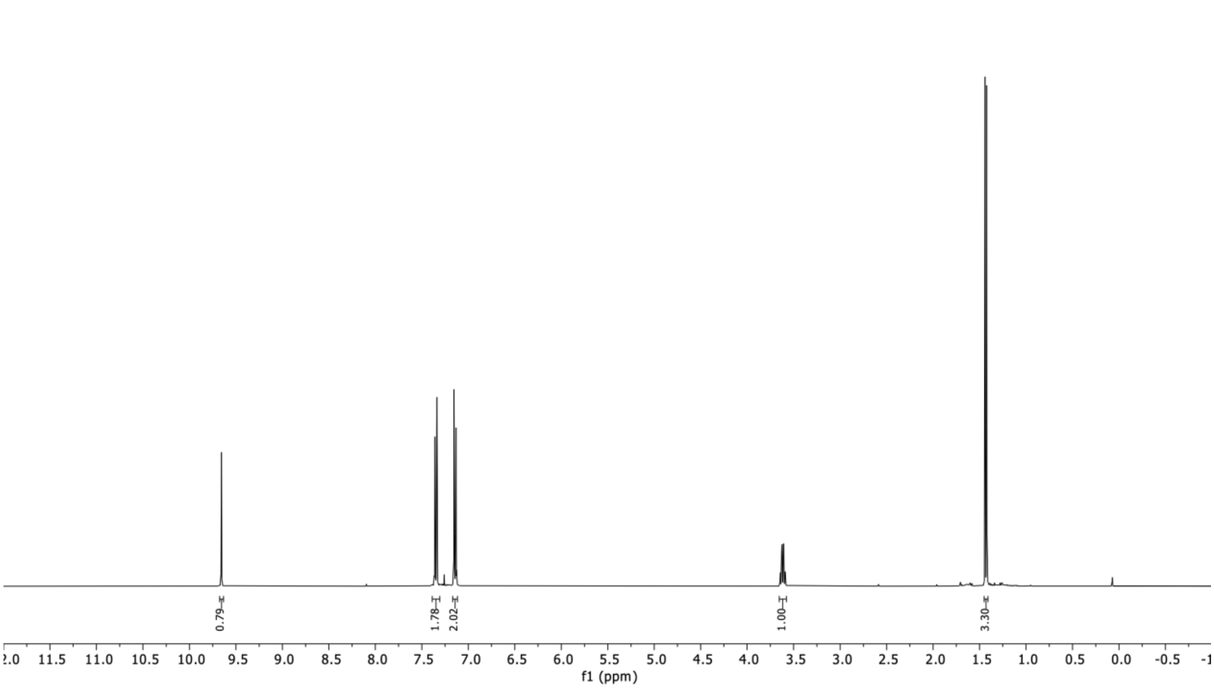

^{13}C-NMR (101 MHz, $CDCl_3$)

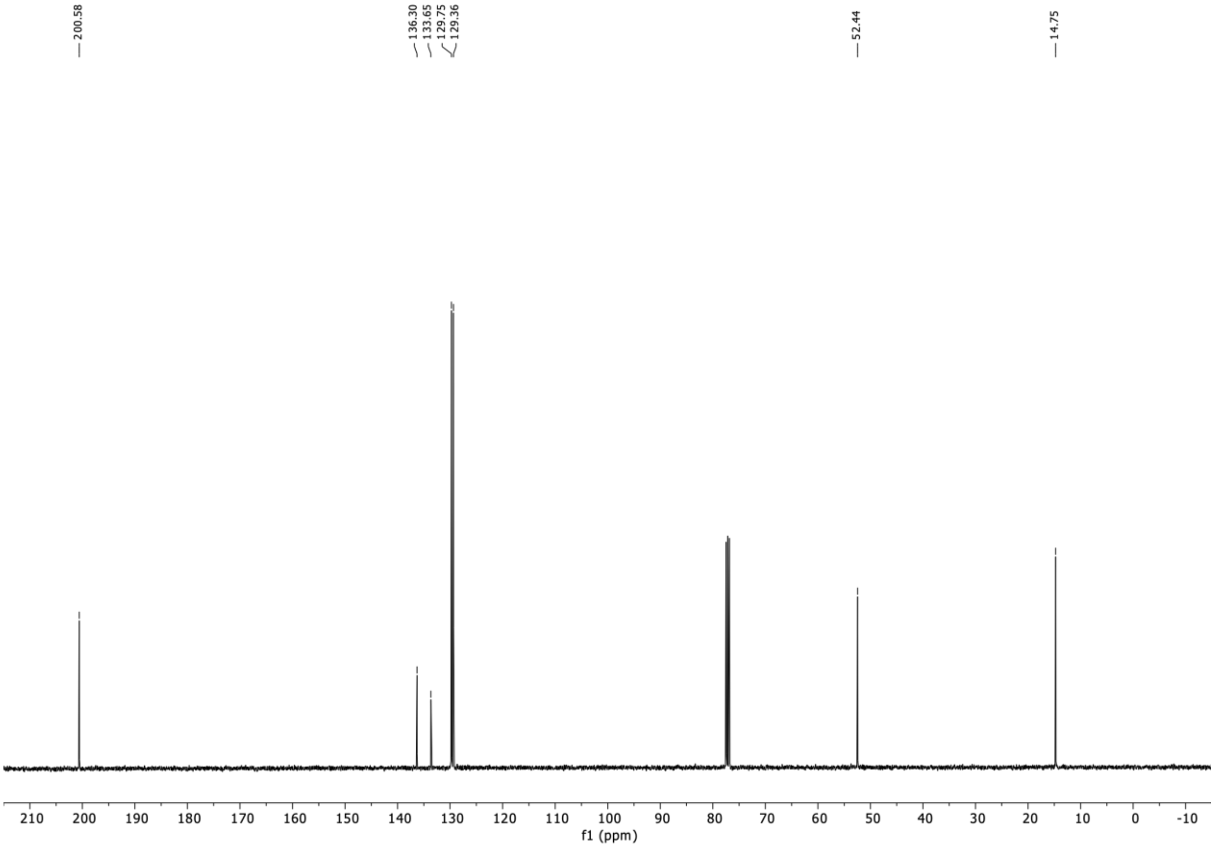

2-(4-Bromophenyl)-propionaldehyde

^{1}H-NMR (400 MHz, $CDCl_3$)

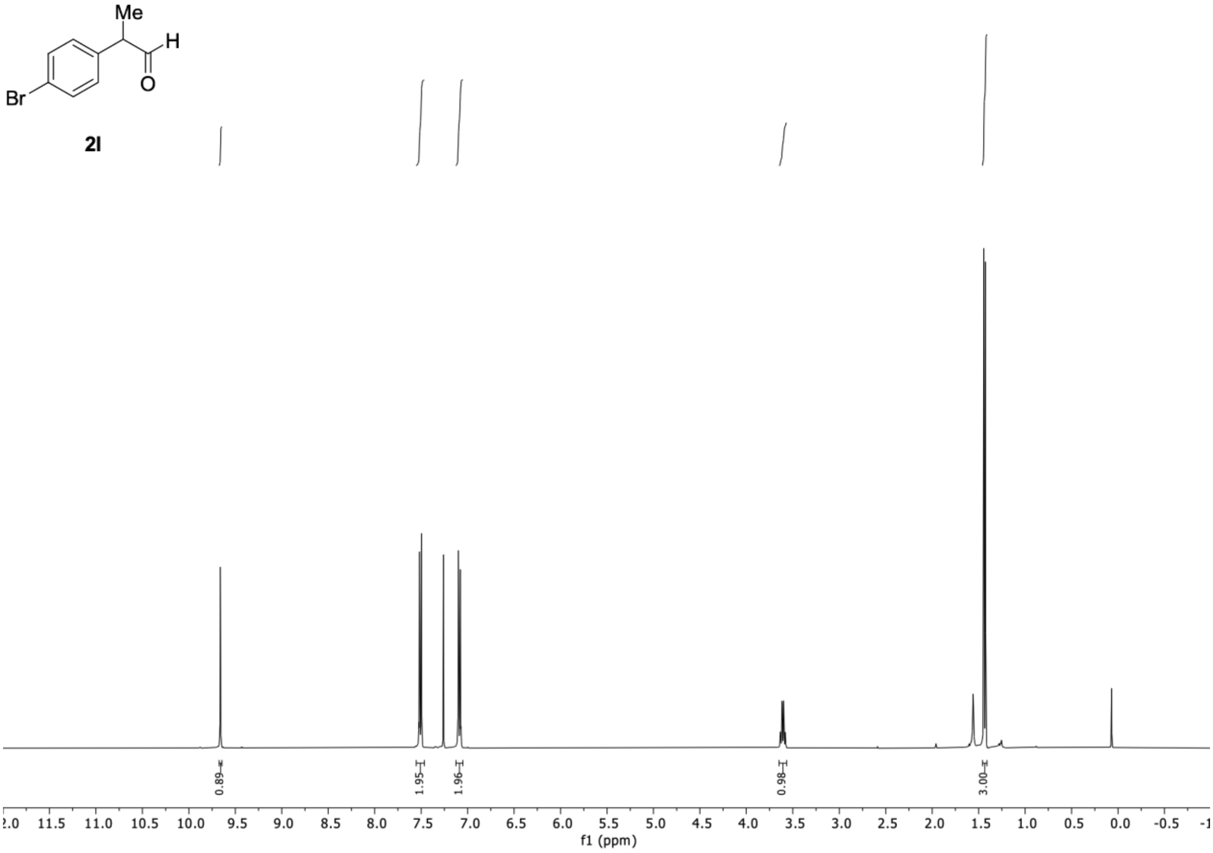

^{13}C-NMR (101 MHz, $CDCl_3$)

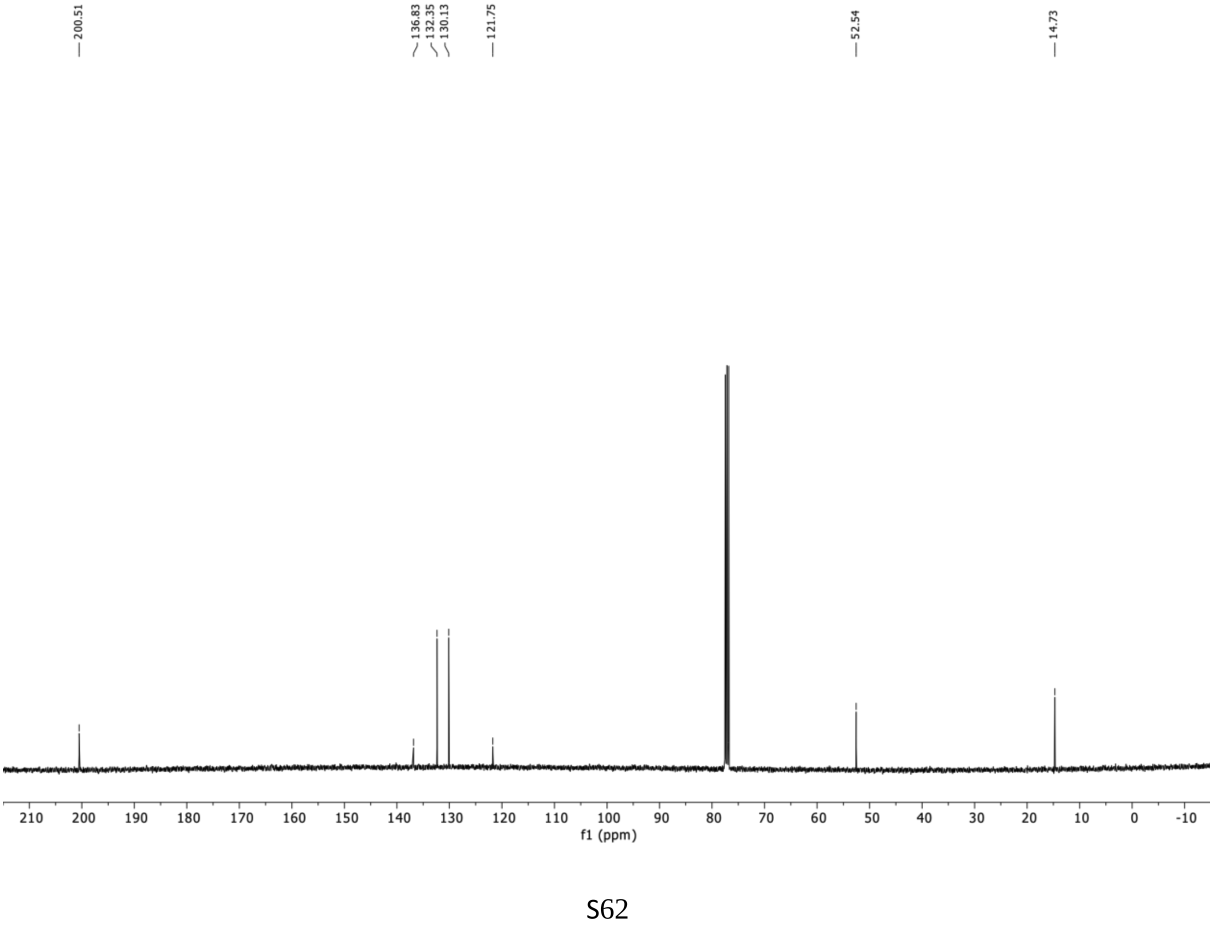

2-(4-Iodophenyl)-propionaldehyde

^{1}H-NMR (400 MHz, $CDCl_3$)

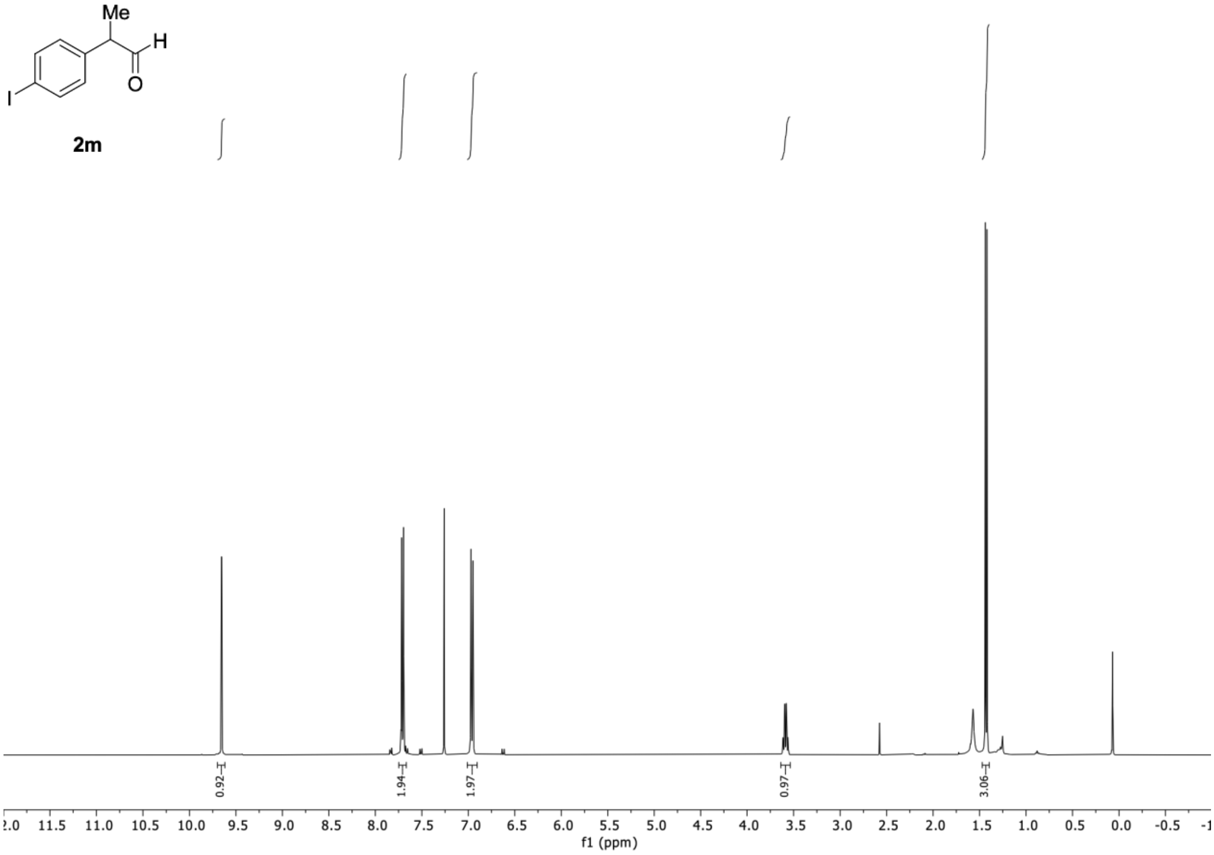

^{13}C-NMR (101 MHz, $CDCl_3$)

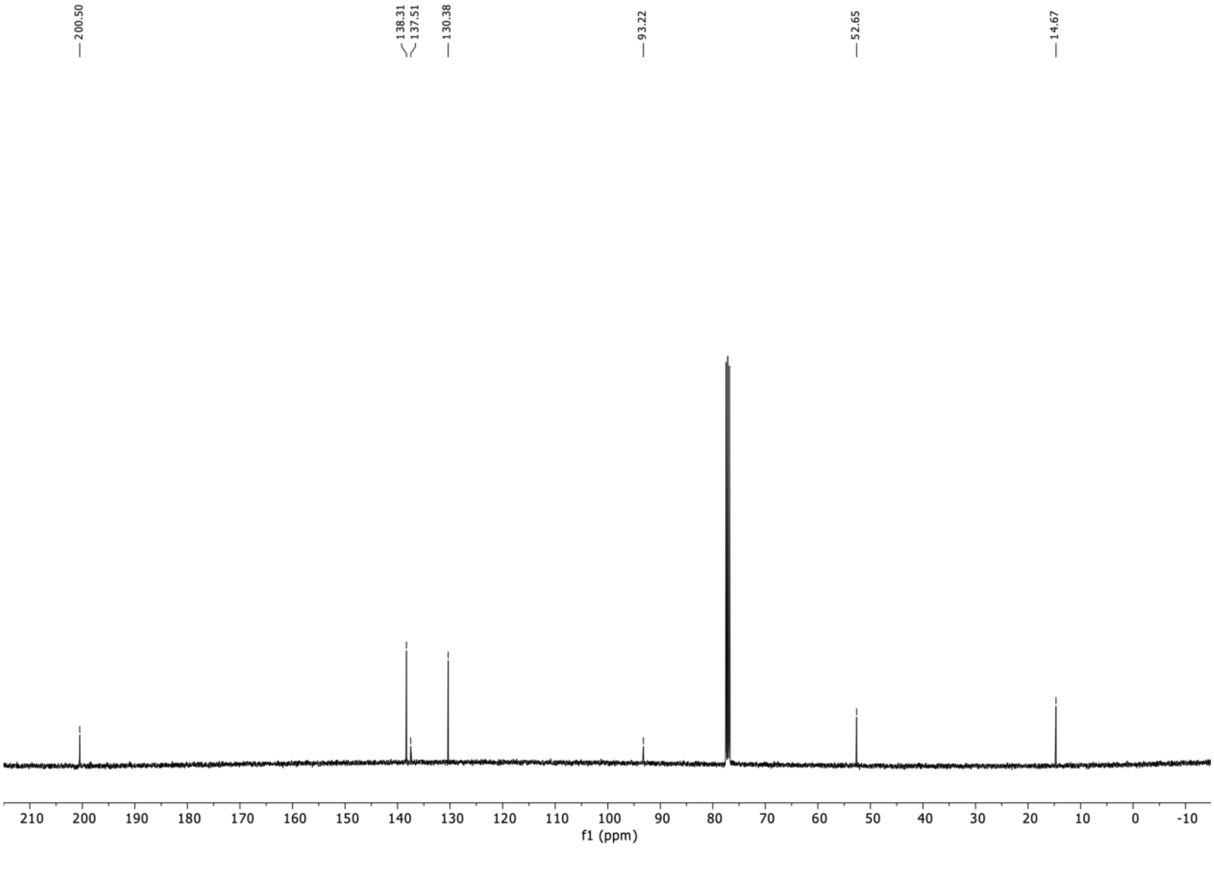

2-(4-Trifluoromethylphenyl)-propionaldehyde

^{1}H-NMR (400 MHz, $CDCl_3$)

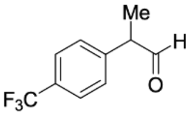

2n

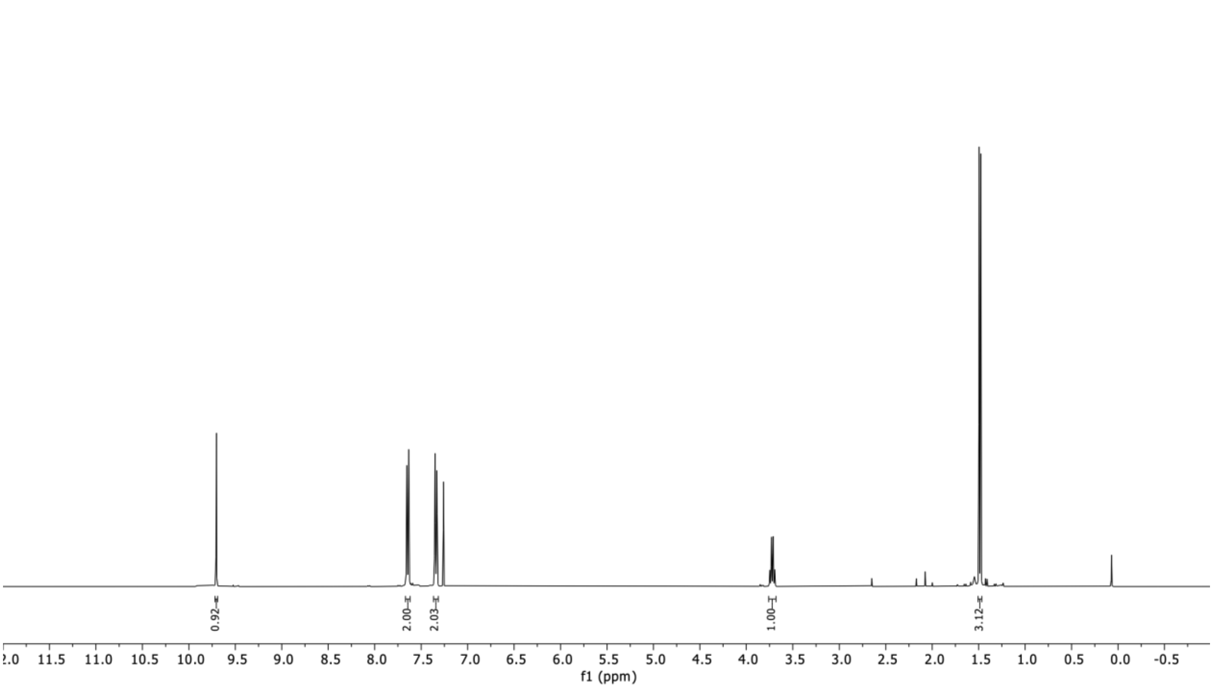

^{13}C-NMR (101 MHz, $CDCl_3$)

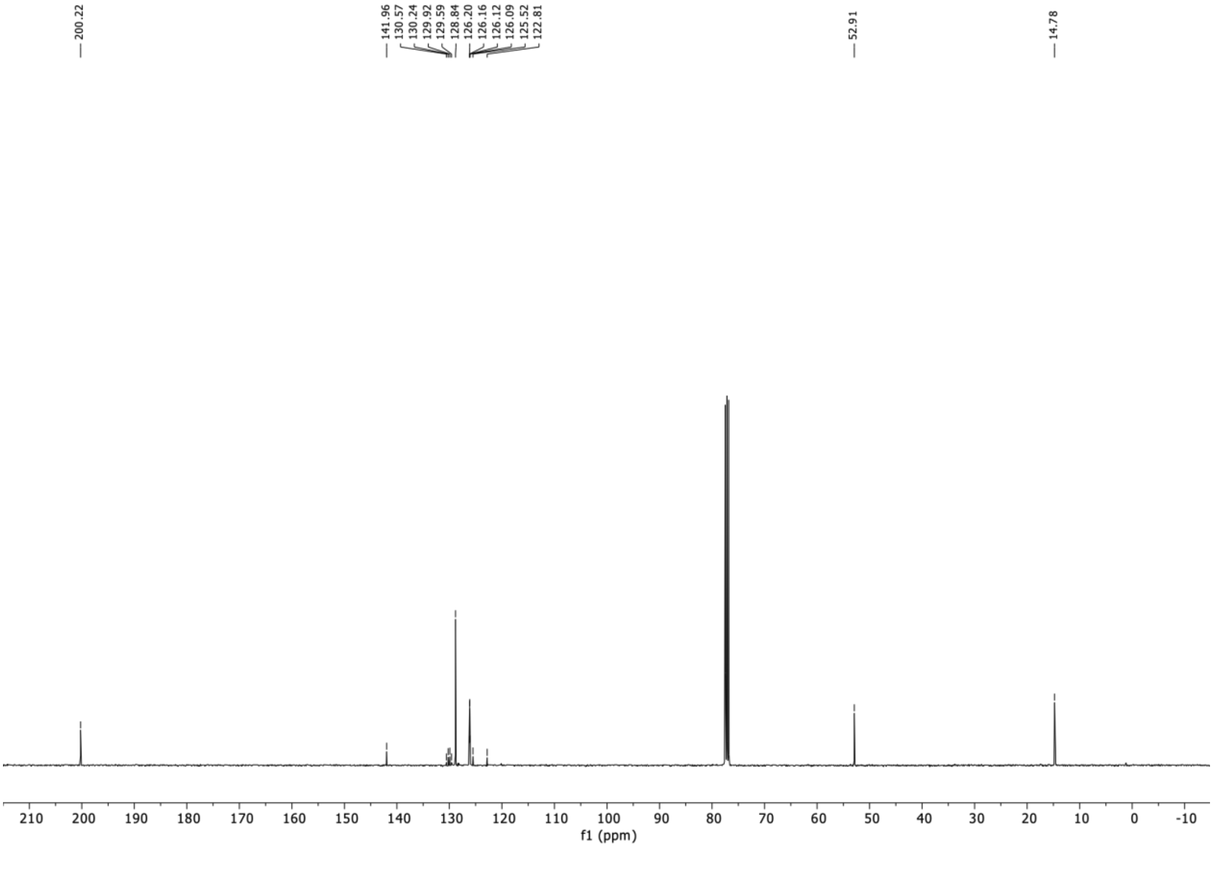

^{19}F-NMR (377 MHz, $CDCl_3$)

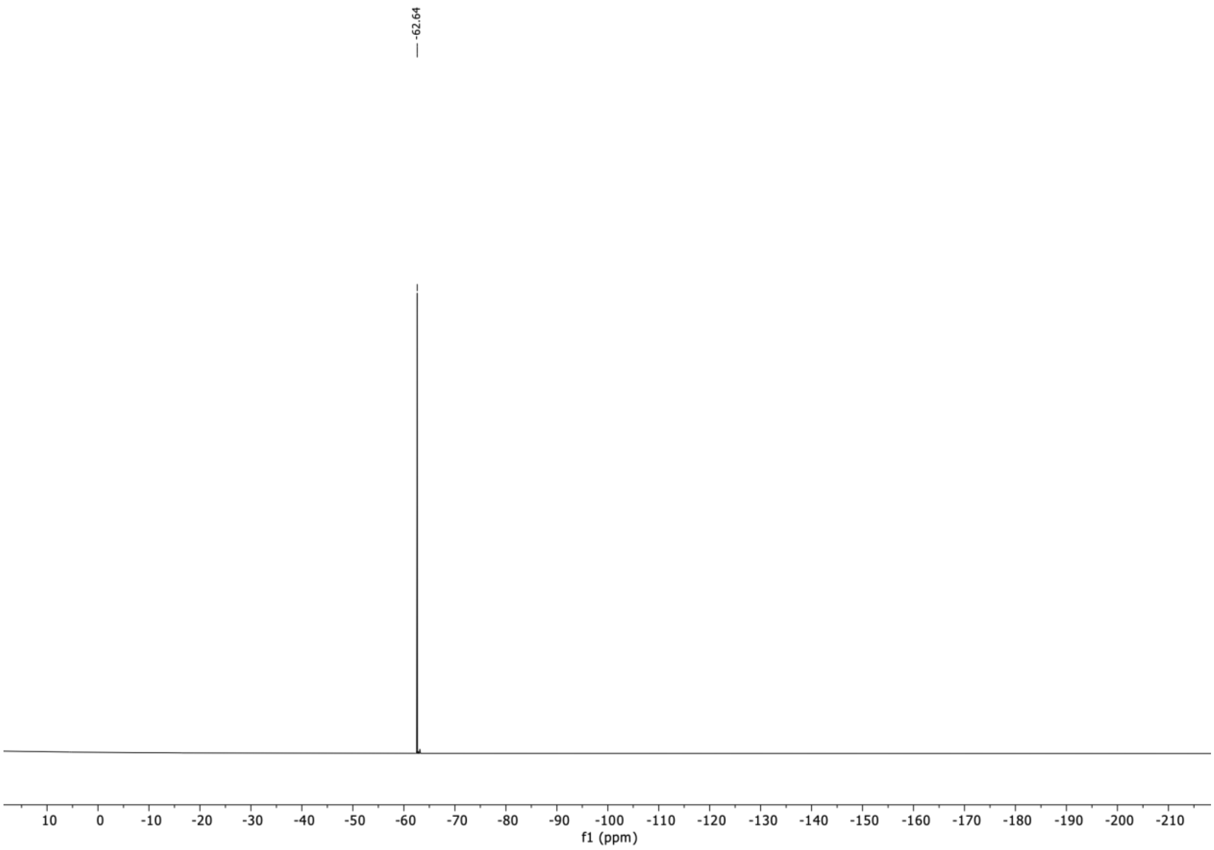

2-(4-Cyanophenyl)-propionaldehyde

^{1}H-NMR (400 MHz, $CDCl_3$)

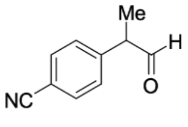

2o

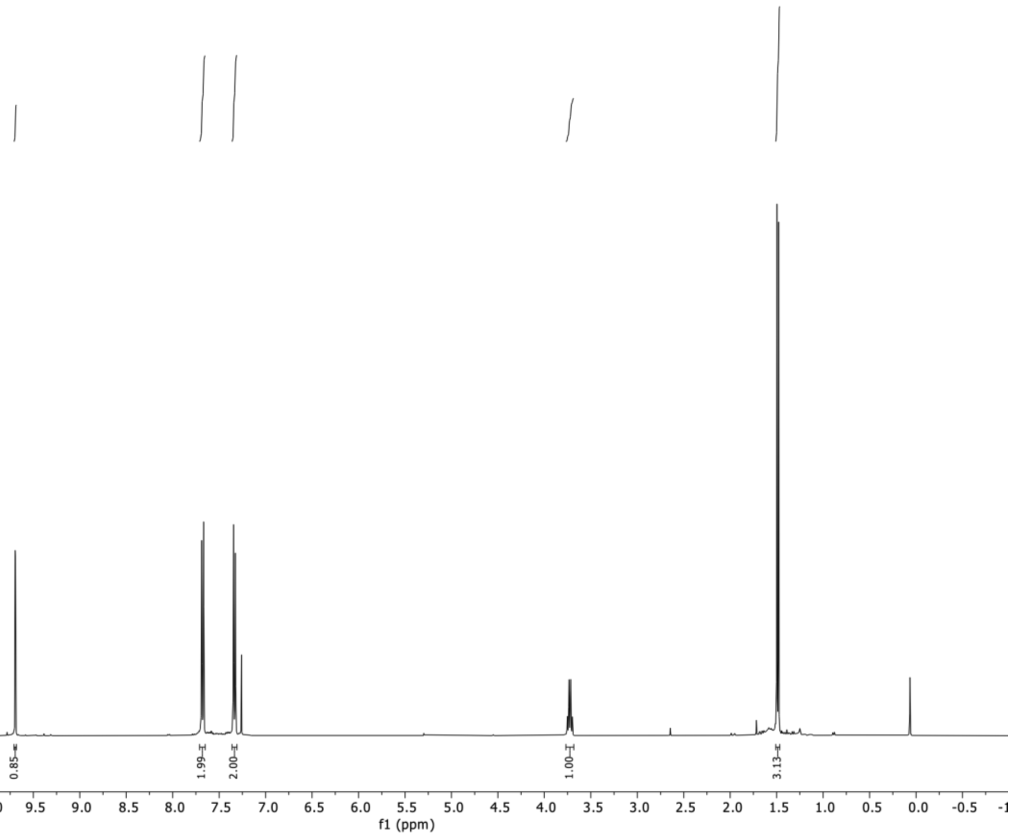

^{13}C-NMR (101 MHz, $CDCl_3$)

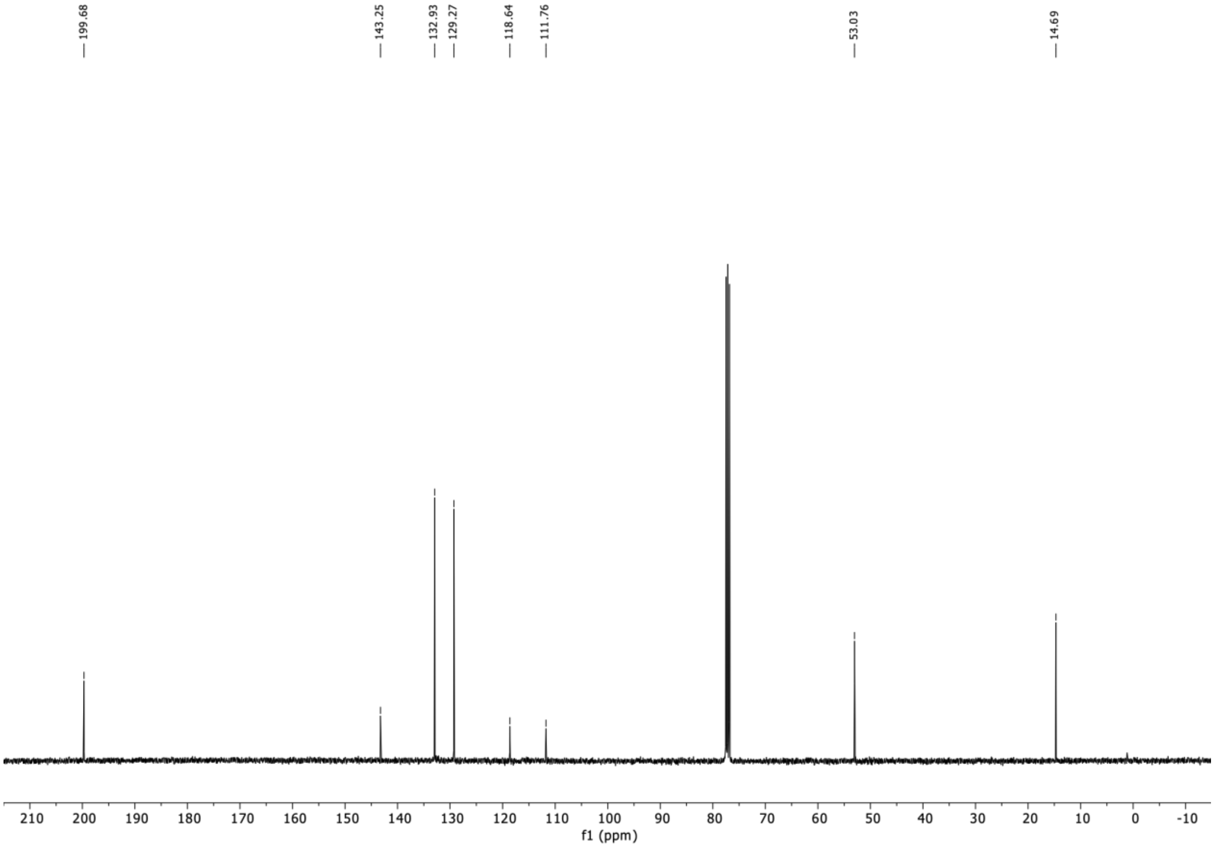

2-(4-Nitrophenyl)-propionaldehyde

^{1}H-NMR (400 MHz, $CDCl_3$)

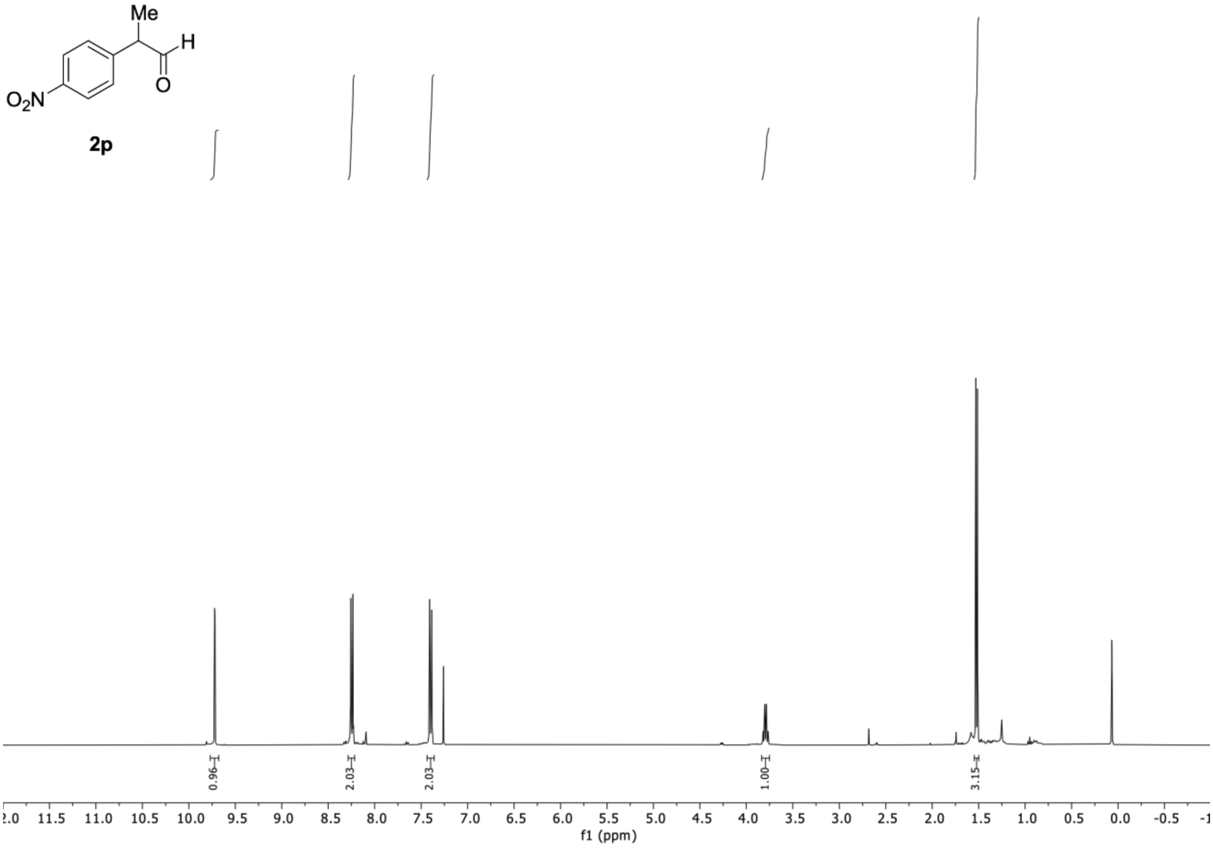

^{13}C-NMR (101 MHz, $CDCl_3$)

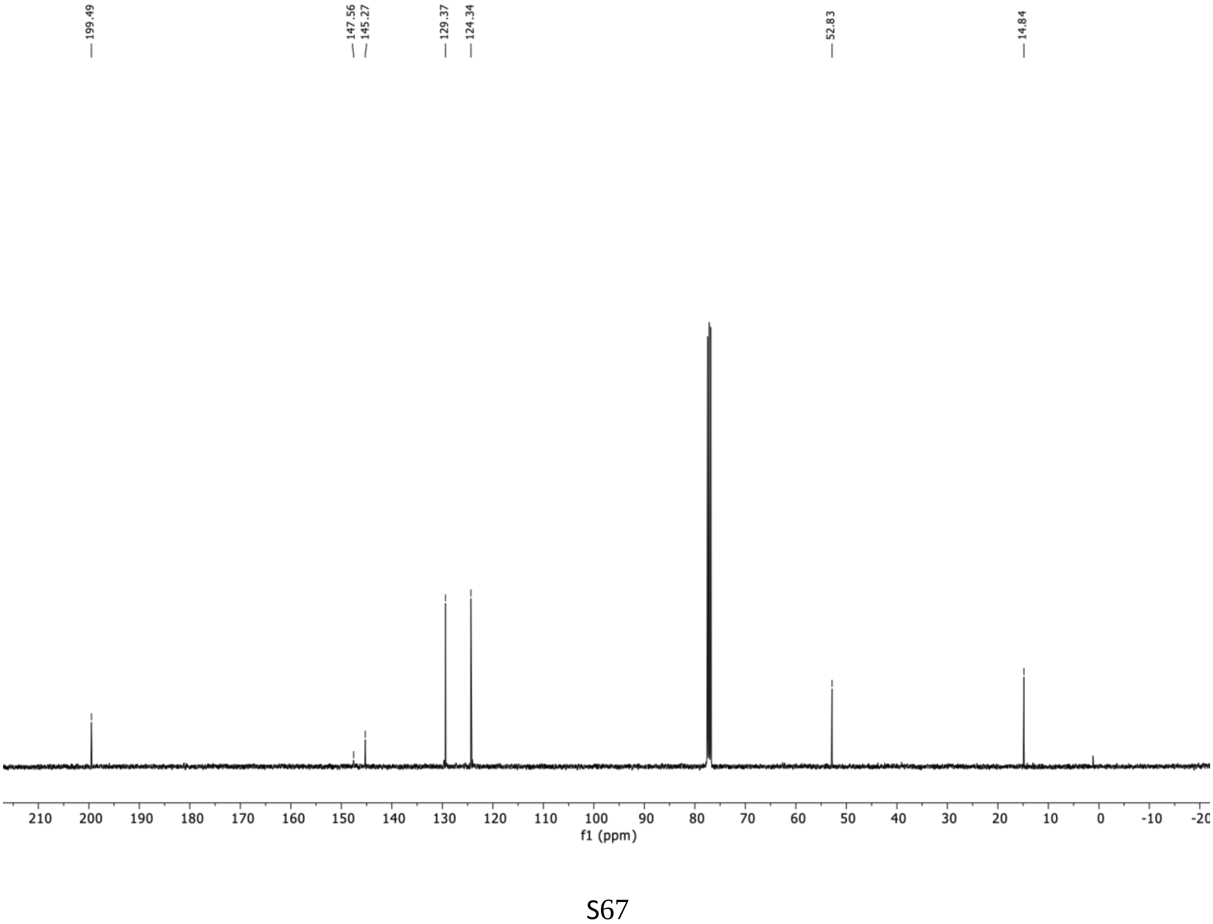

2-(4-Methoxycarbonylphenyl)-propionaldehyde

^{1}H-NMR (400 MHz, $CDCl_3$)

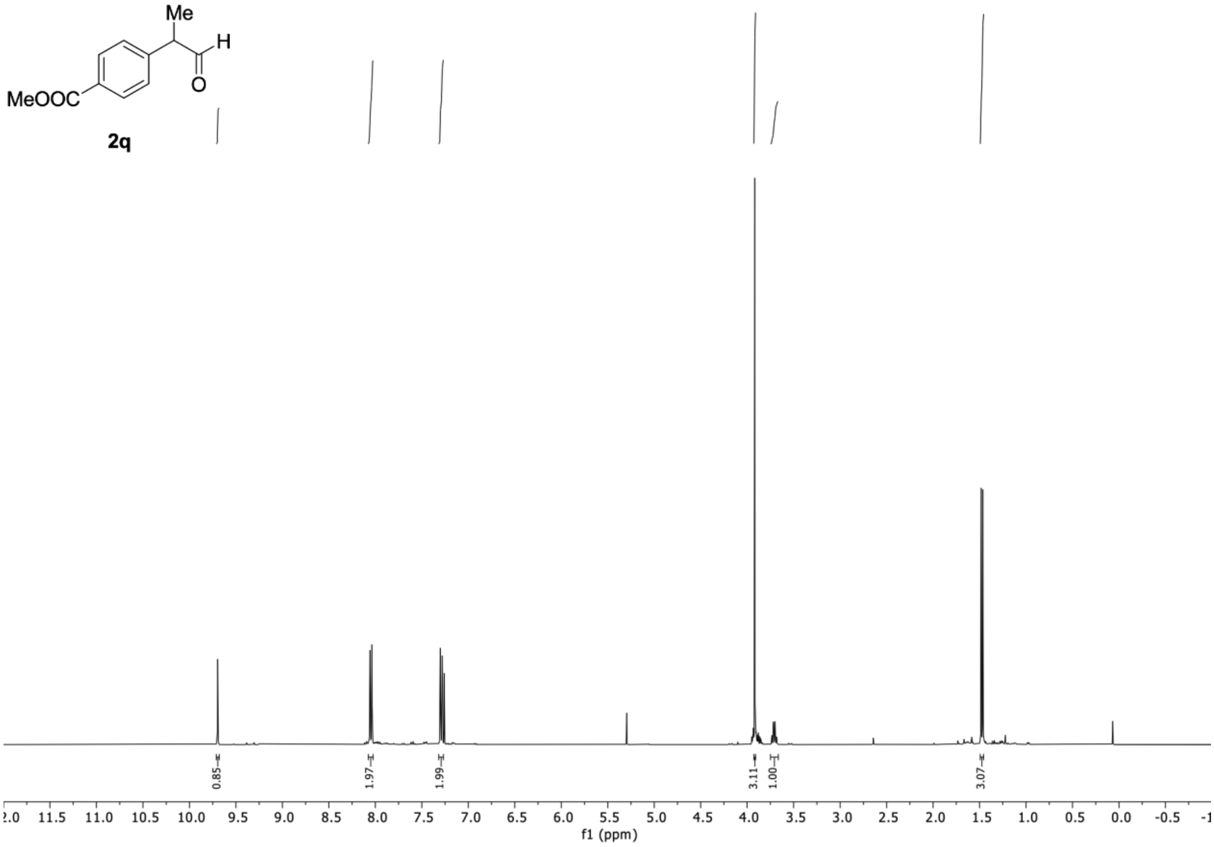

^{13}C-NMR (101 MHz, $CDCl_3$)

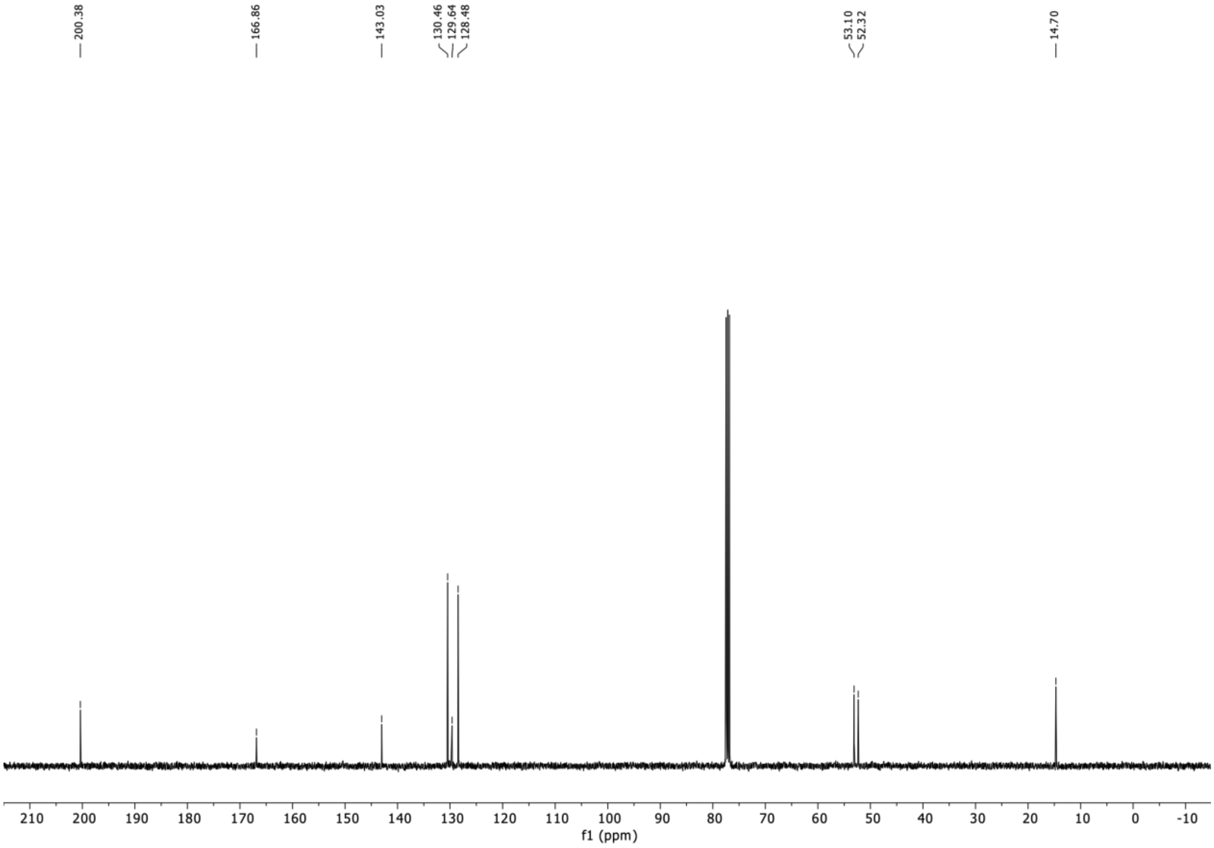

2-(Naphth-2-yl)propionaldehyde

^{1}H-NMR (400 MHz, $CDCl_3$)

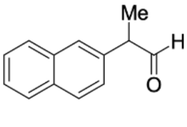

2r

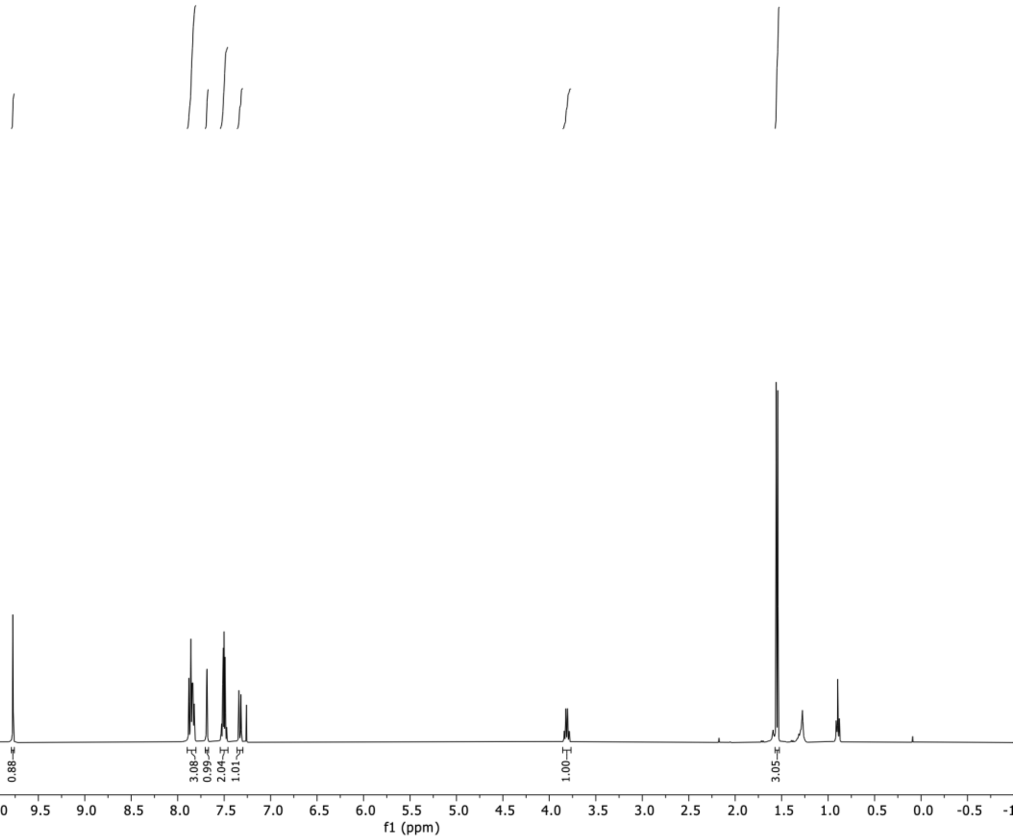

^{13}C-NMR (101 MHz, $CDCl_3$)

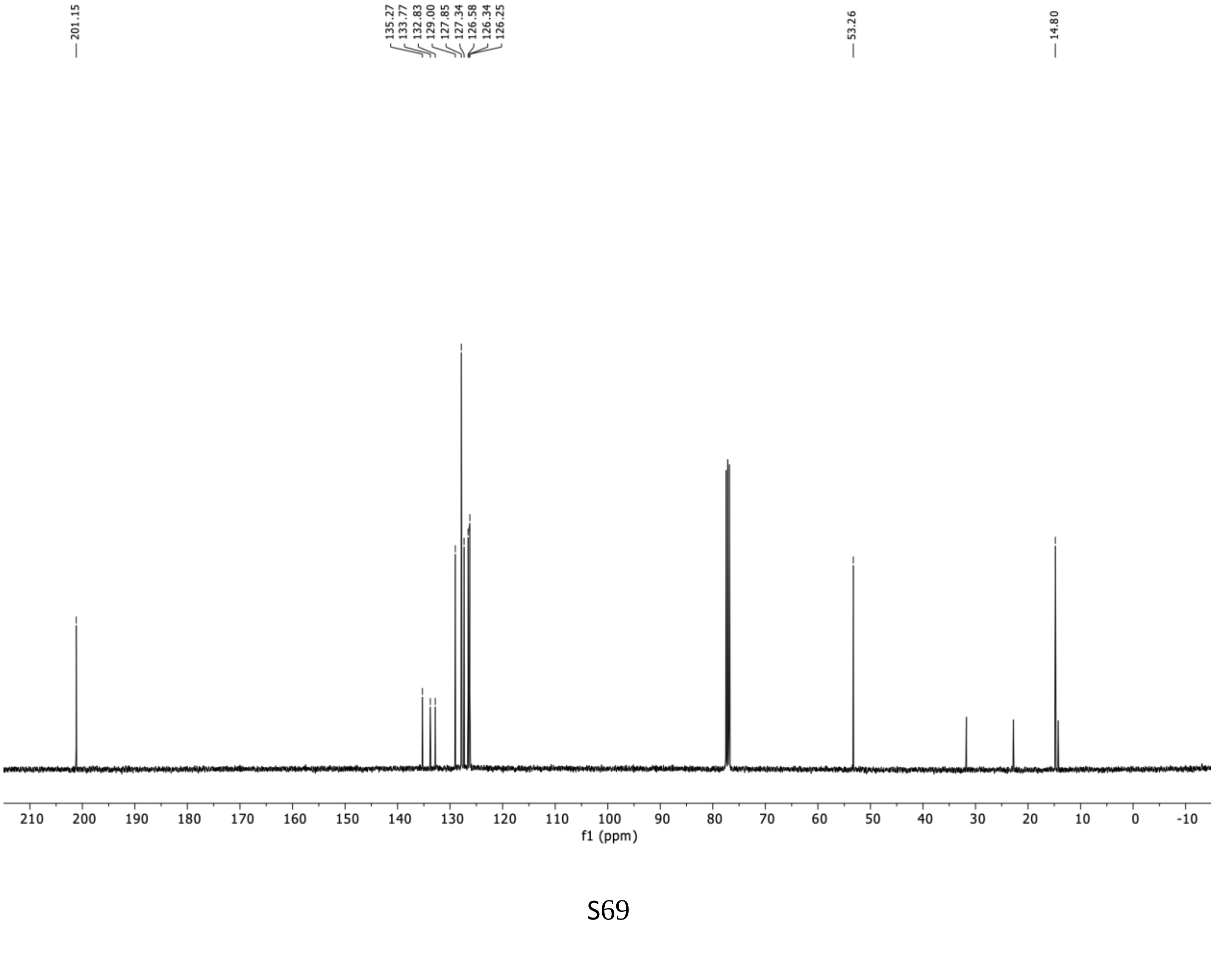

2-(Naphth-1-yl)propionaldehyde

^{1}H-NMR (400 MHz, CDCl$_3$)

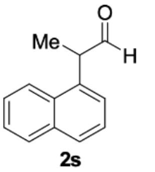
2s

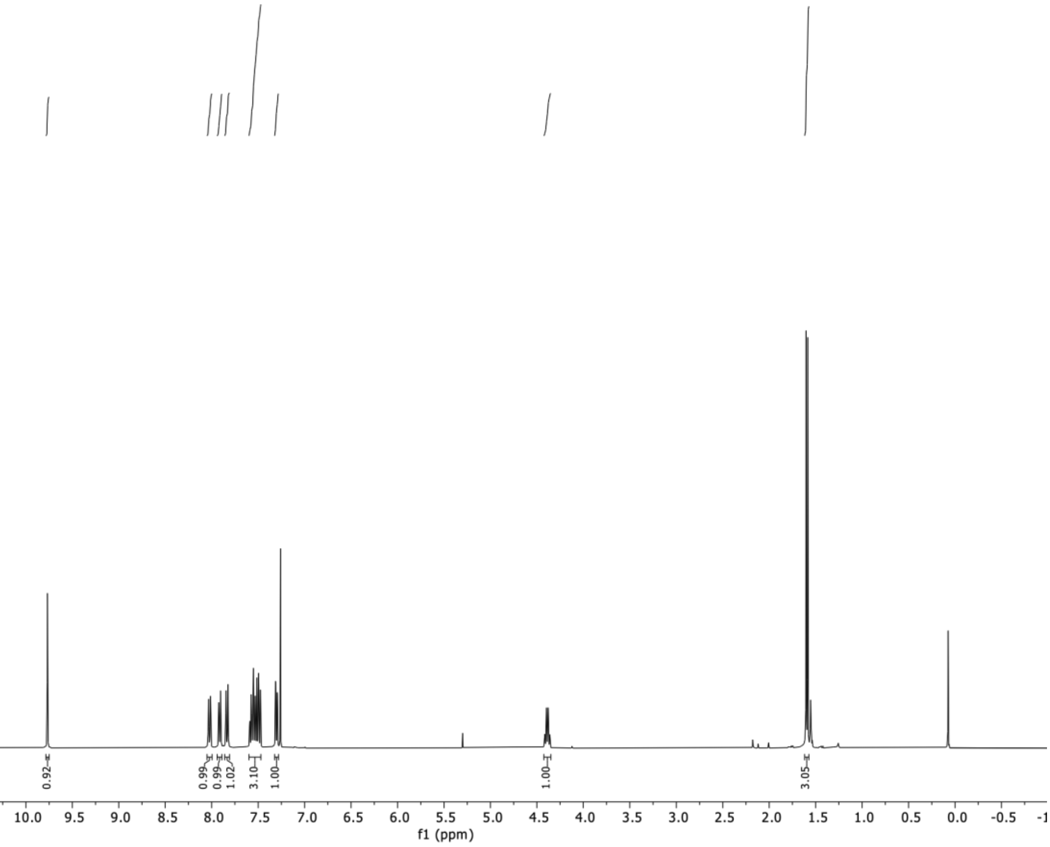

^{13}C-NMR (101 MHz, CDCl$_3$)

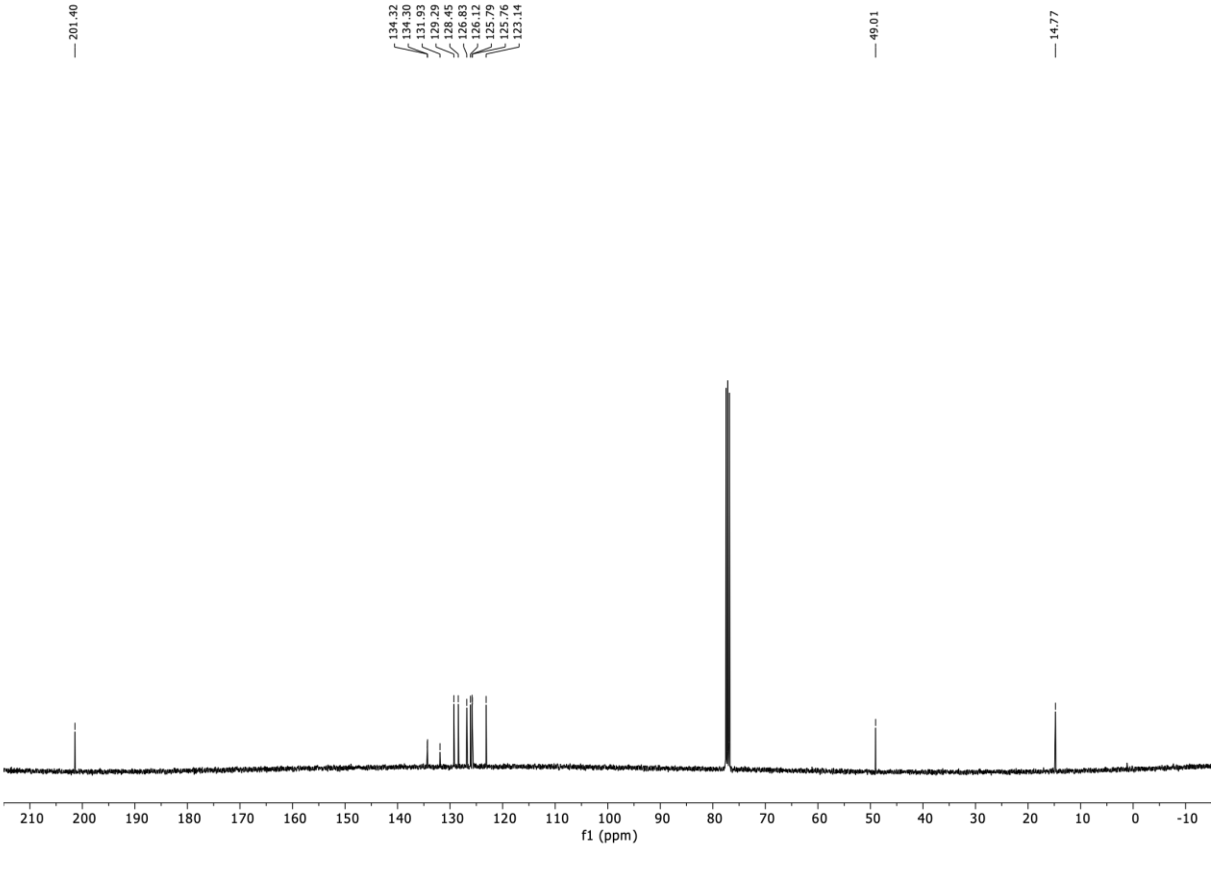

2-(4-*iso*-Butylphenyl)-propionaldehyde

^{1}H-NMR (400 MHz, $CDCl_3$)

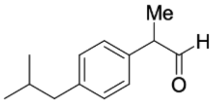

2t

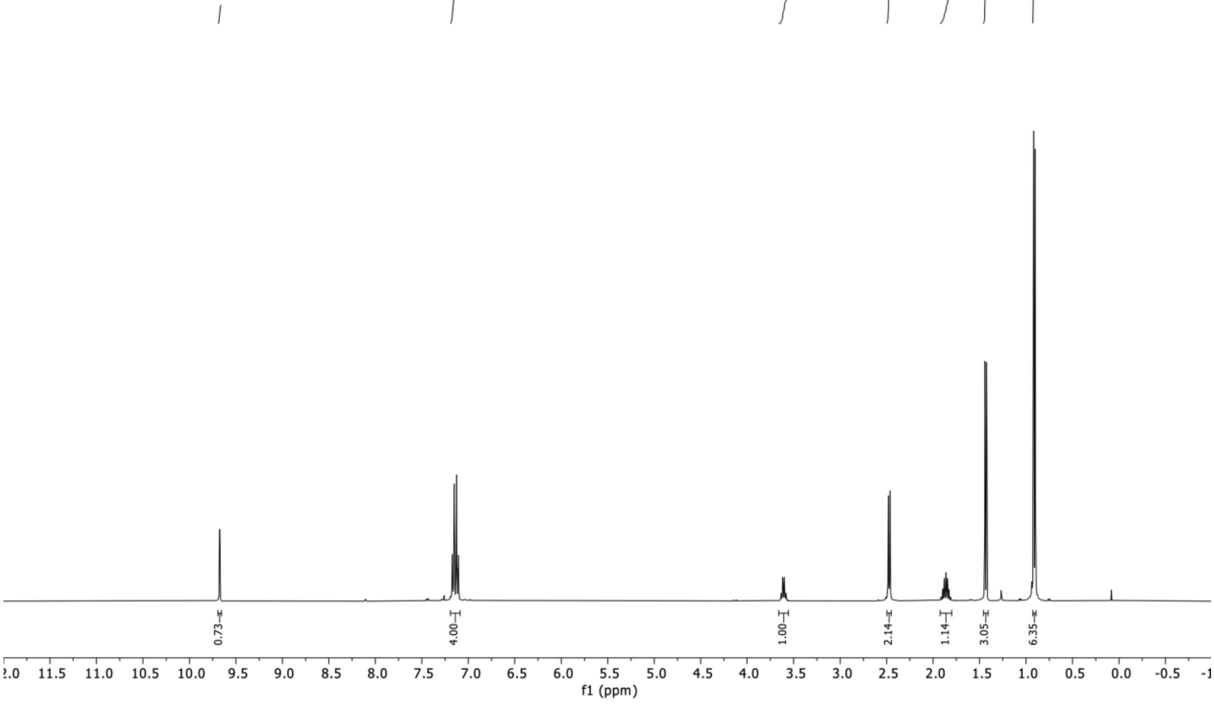

^{13}C-NMR (101 MHz, $CDCl_3$)

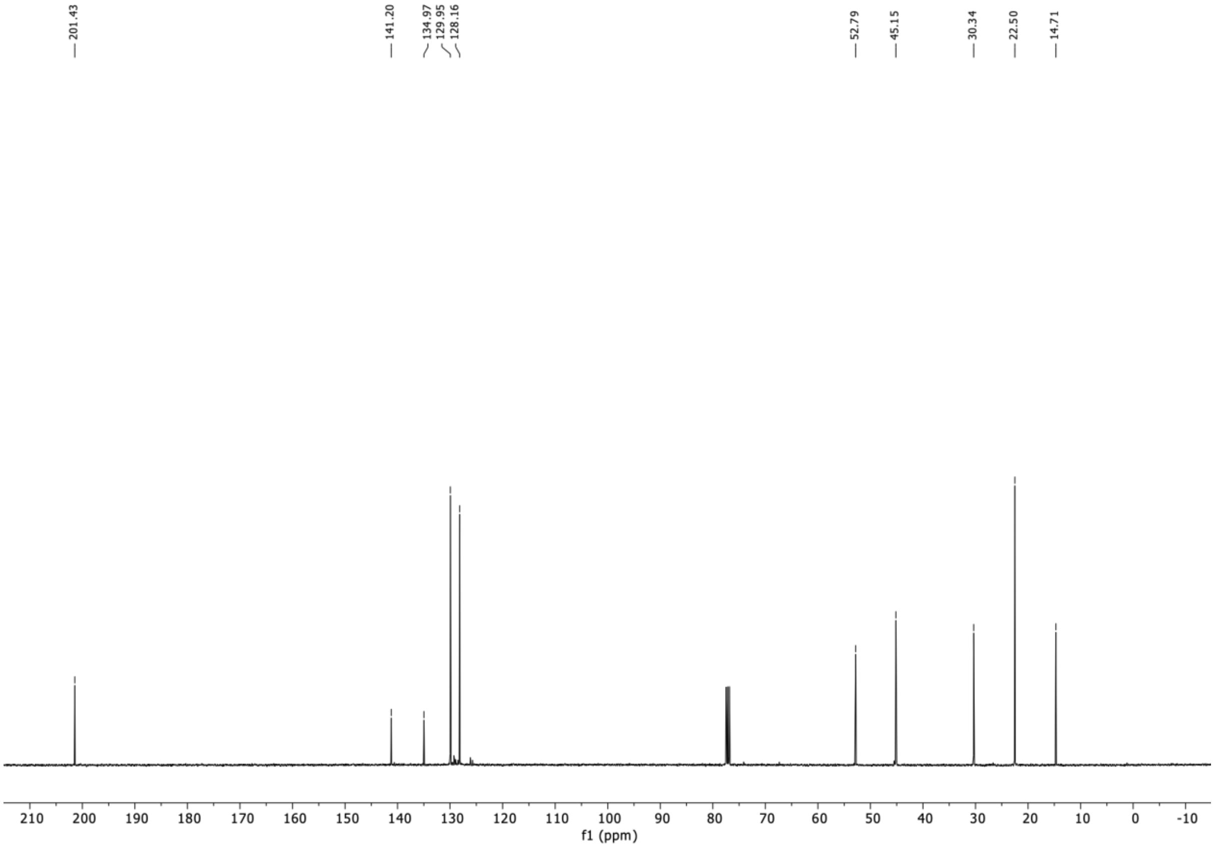

2-Methylheptanal

^{1}H-NMR (400 MHz, $CDCl_3$)

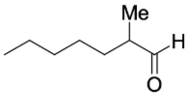

2u

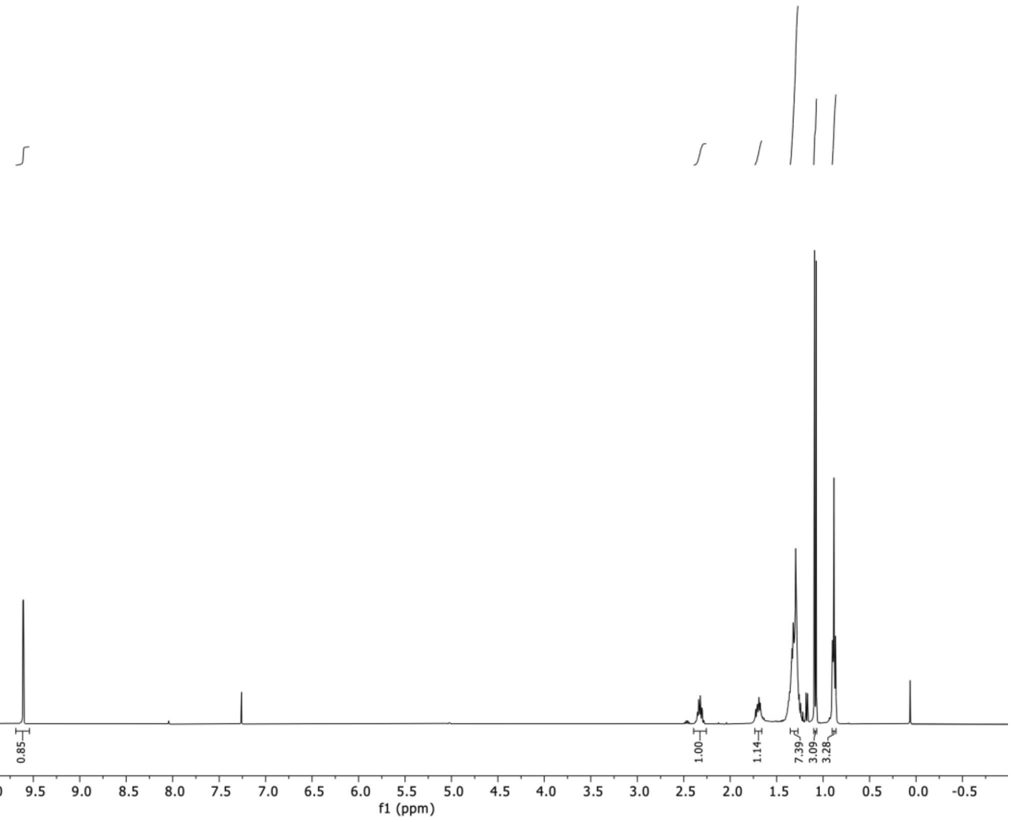

^{13}C-NMR (101 MHz, $CDCl_3$)

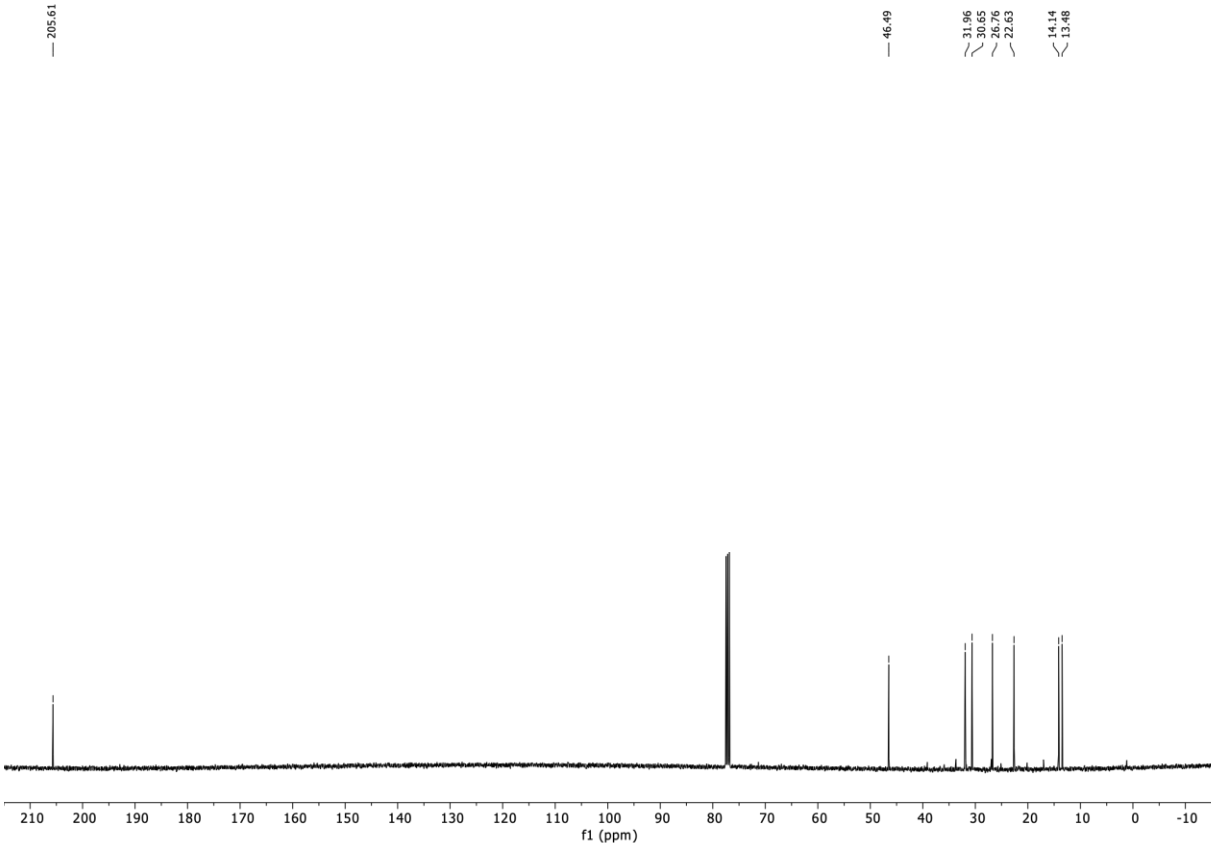

2-Cyclohexylpropionaldehyde

^{1}H-NMR (400 MHz, $CDCl_3$)

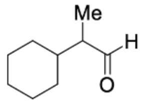

2v

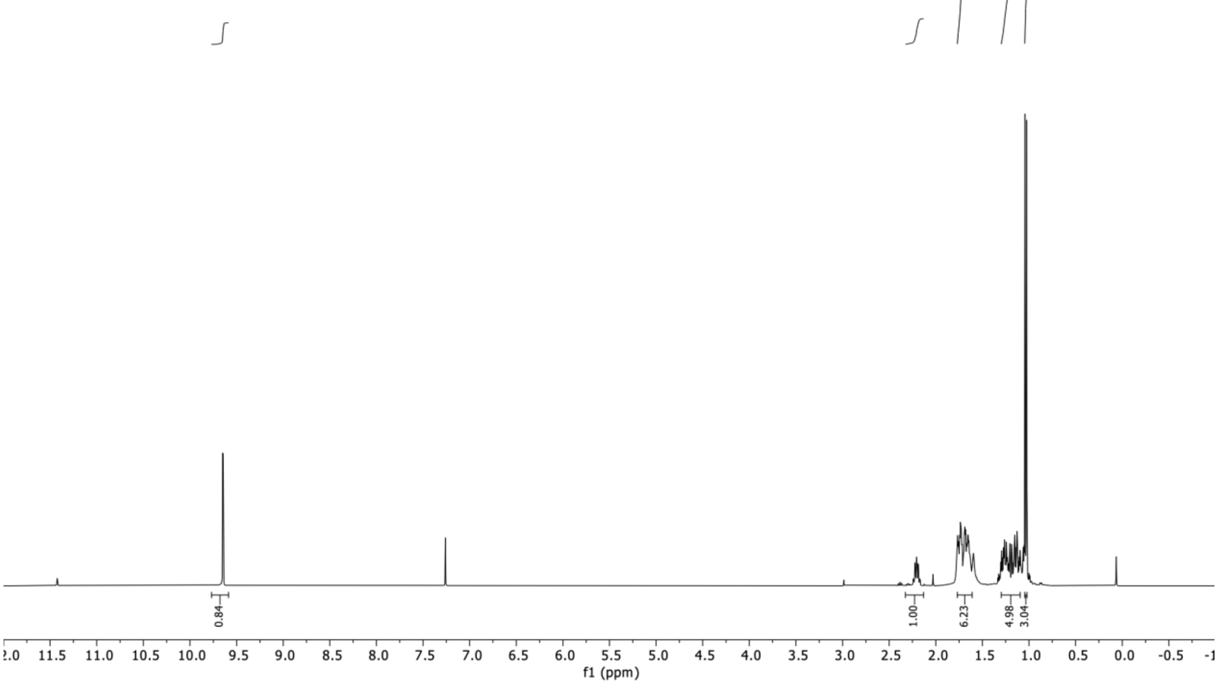

^{13}C-NMR (101 MHz, $CDCl_3$)

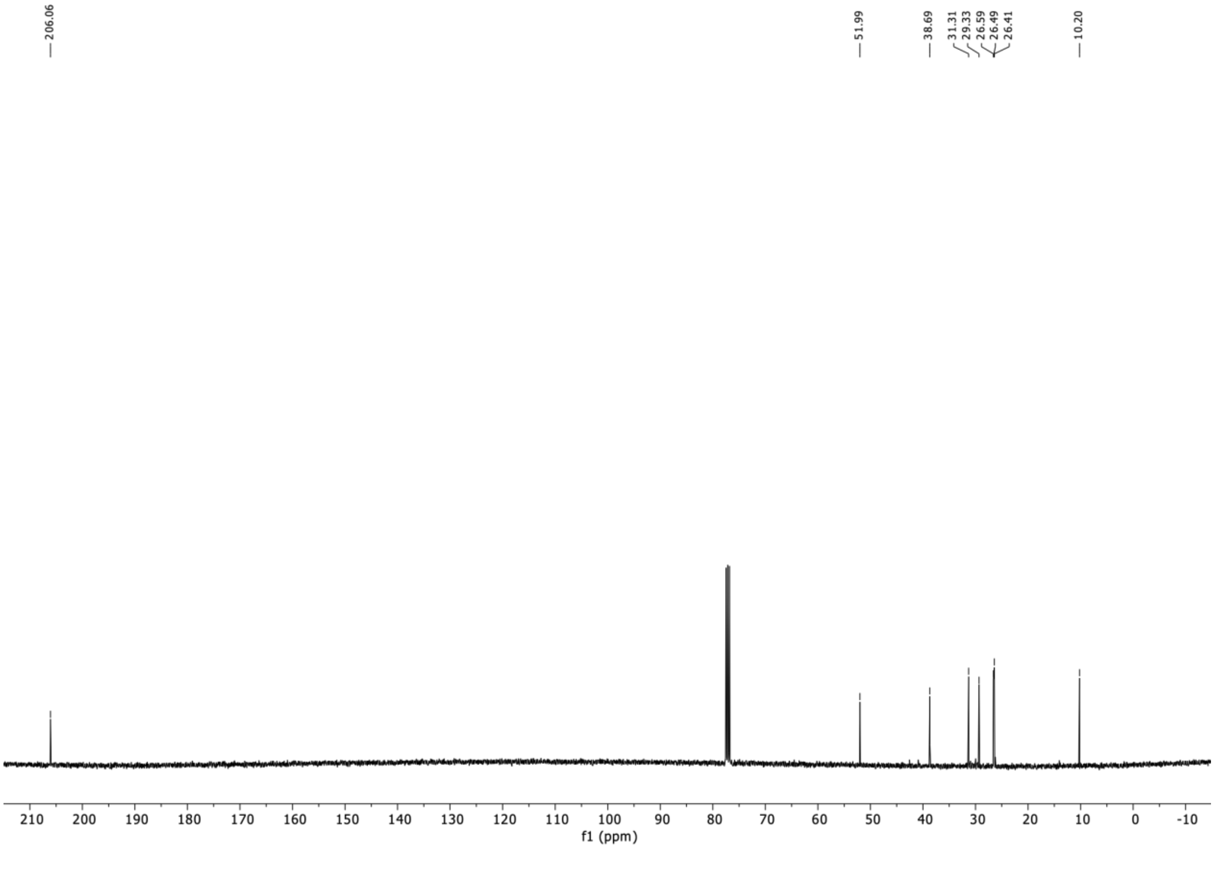

***syn*-2,5-Dimethyl-2,5-diphenyl-1,4-dioxane,**

^{1}H-NMR (400 MHz, $CDCl_3$)

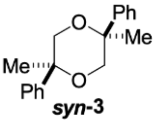

syn-**3**

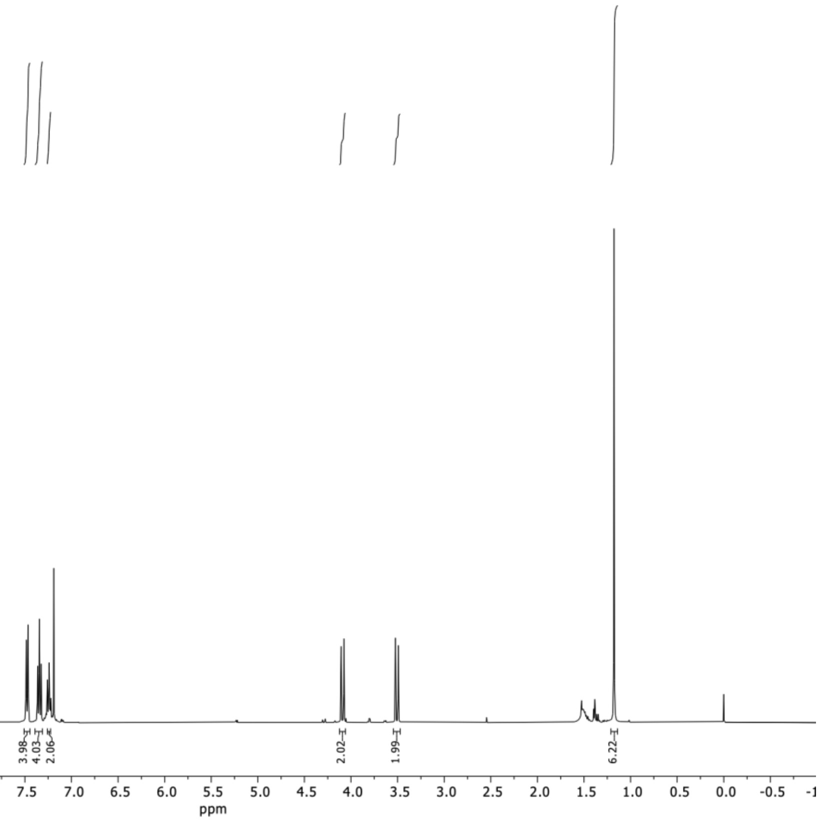

^{13}C-NMR (101 MHz, $CDCl_3$)

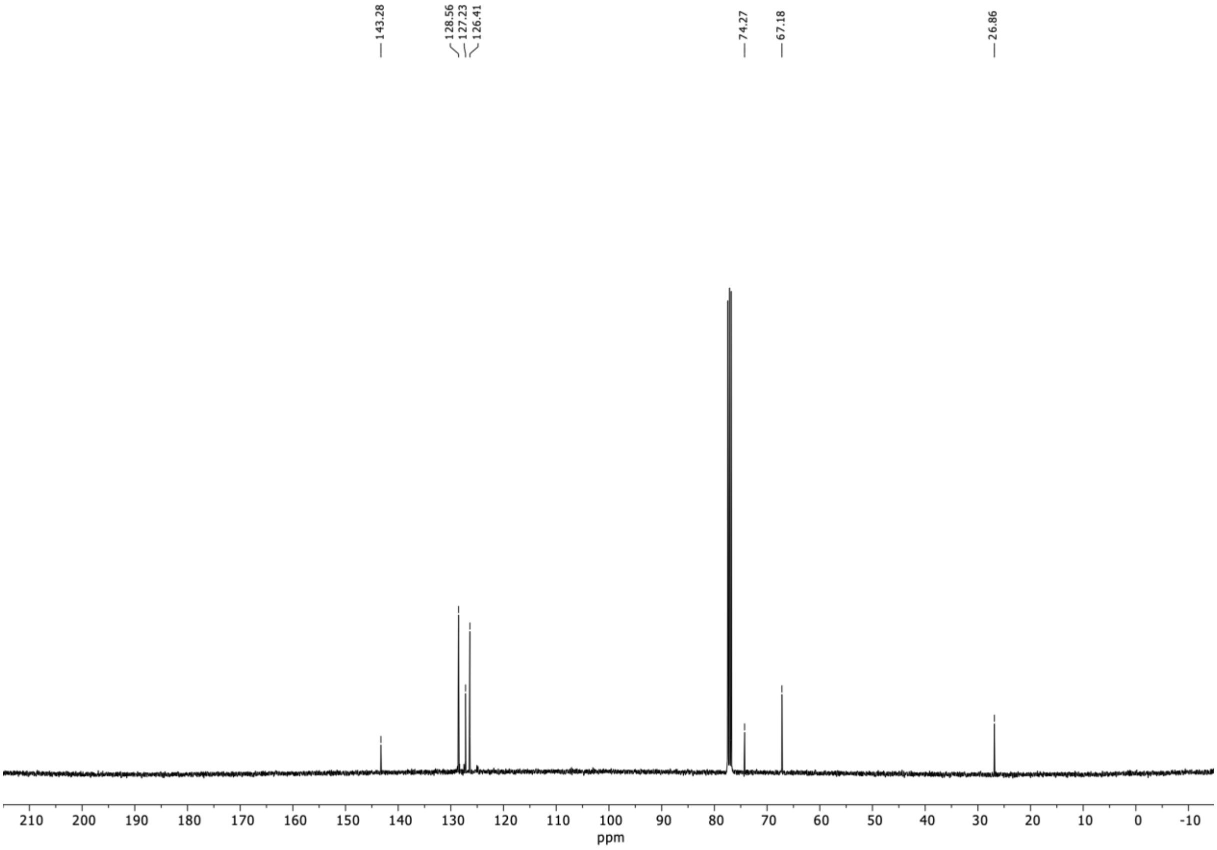

***anti*-2,5-Dimethyl-2,5-diphenyl-1,4-dioxane,**

^{1}H-NMR (400 MHz, $CDCl_3$)

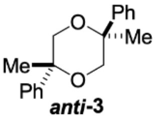

***anti*-3**

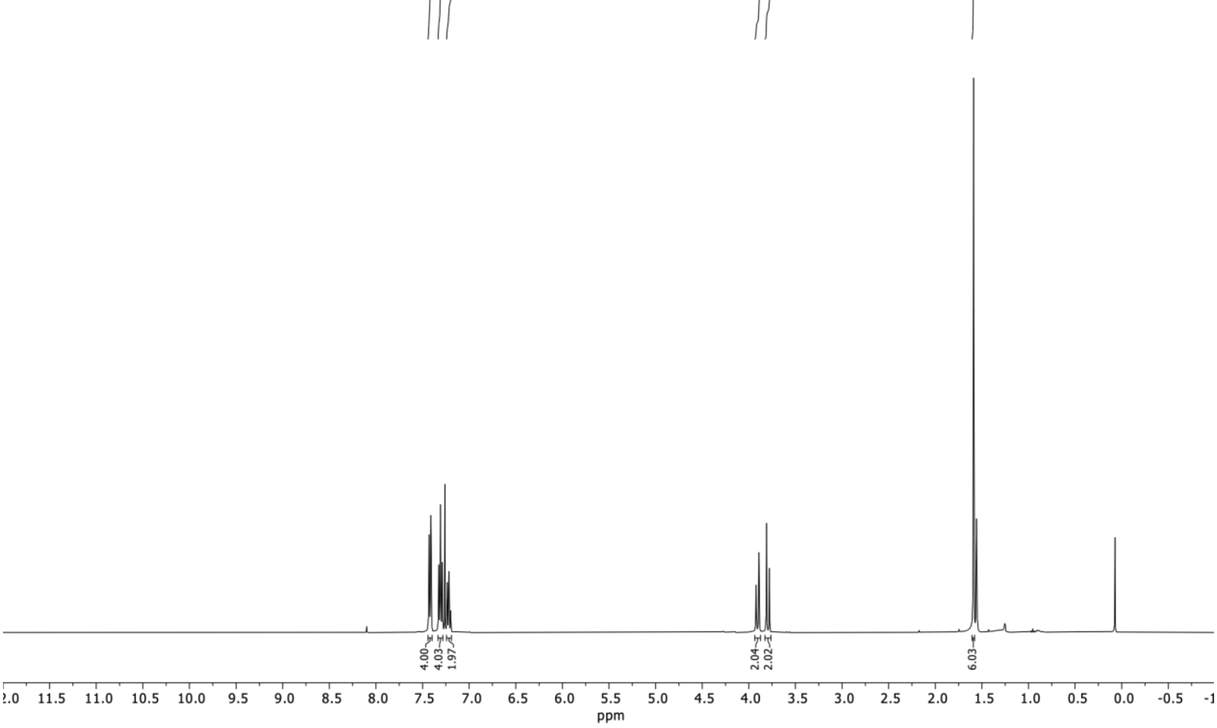

^{13}C-NMR (101 MHz, $CDCl_3$)

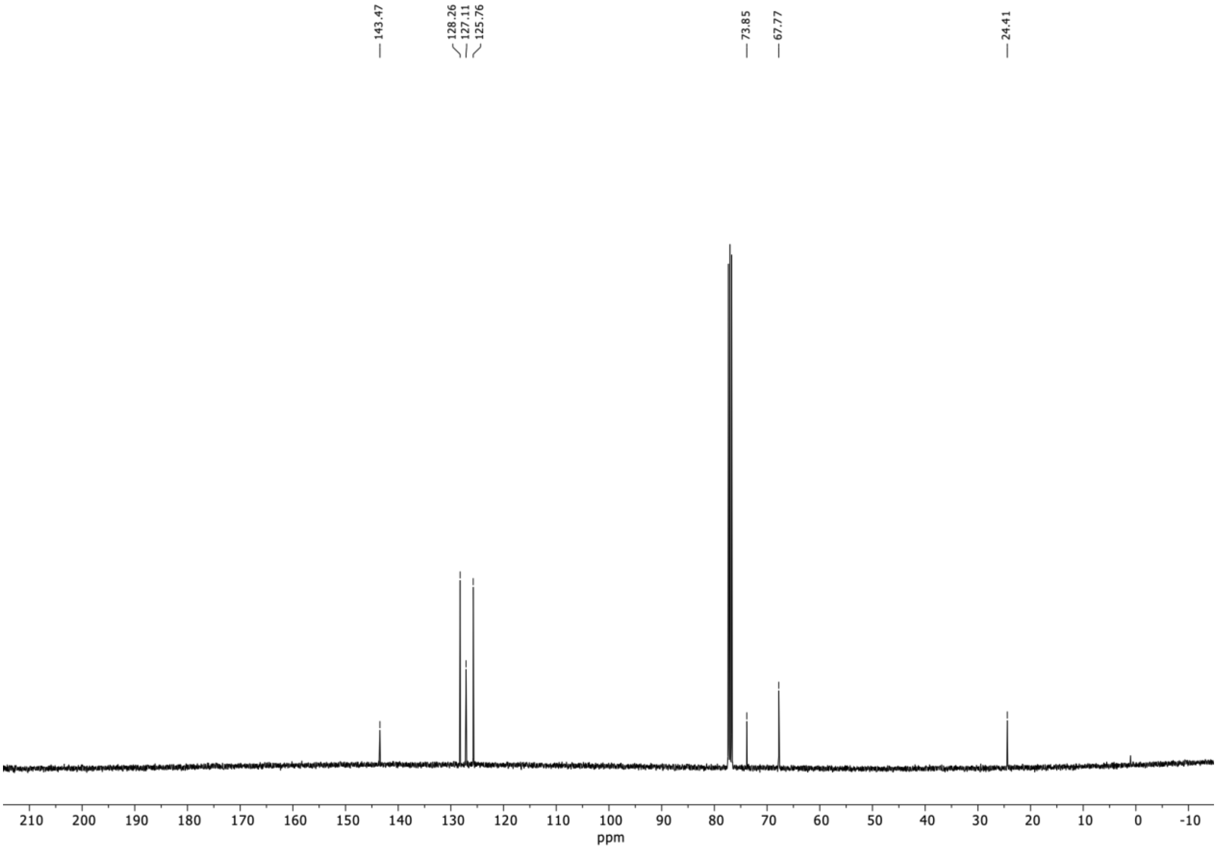

2-Phenyl-1-propanol

^{1}H-NMR (400 MHz, CD_2Cl_2)

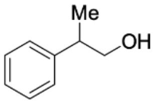

4a

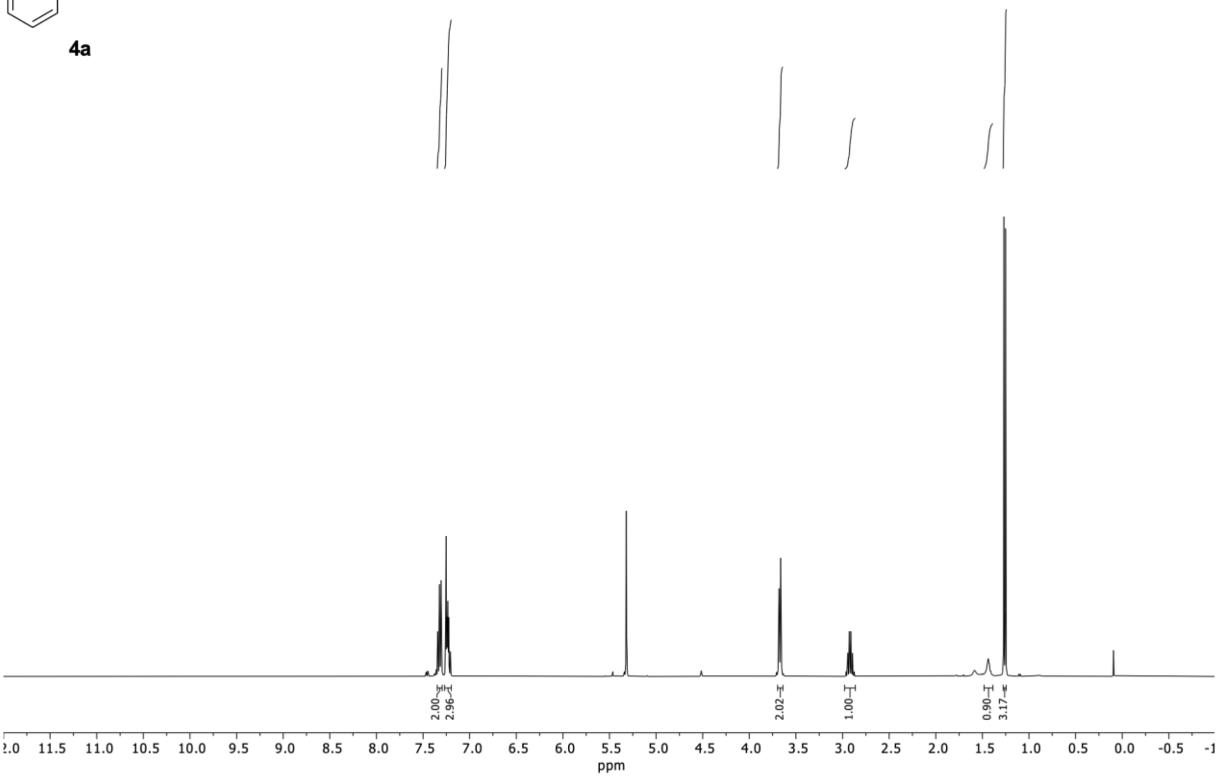

^{13}C-NMR (101 MHz, CD_2Cl_2)

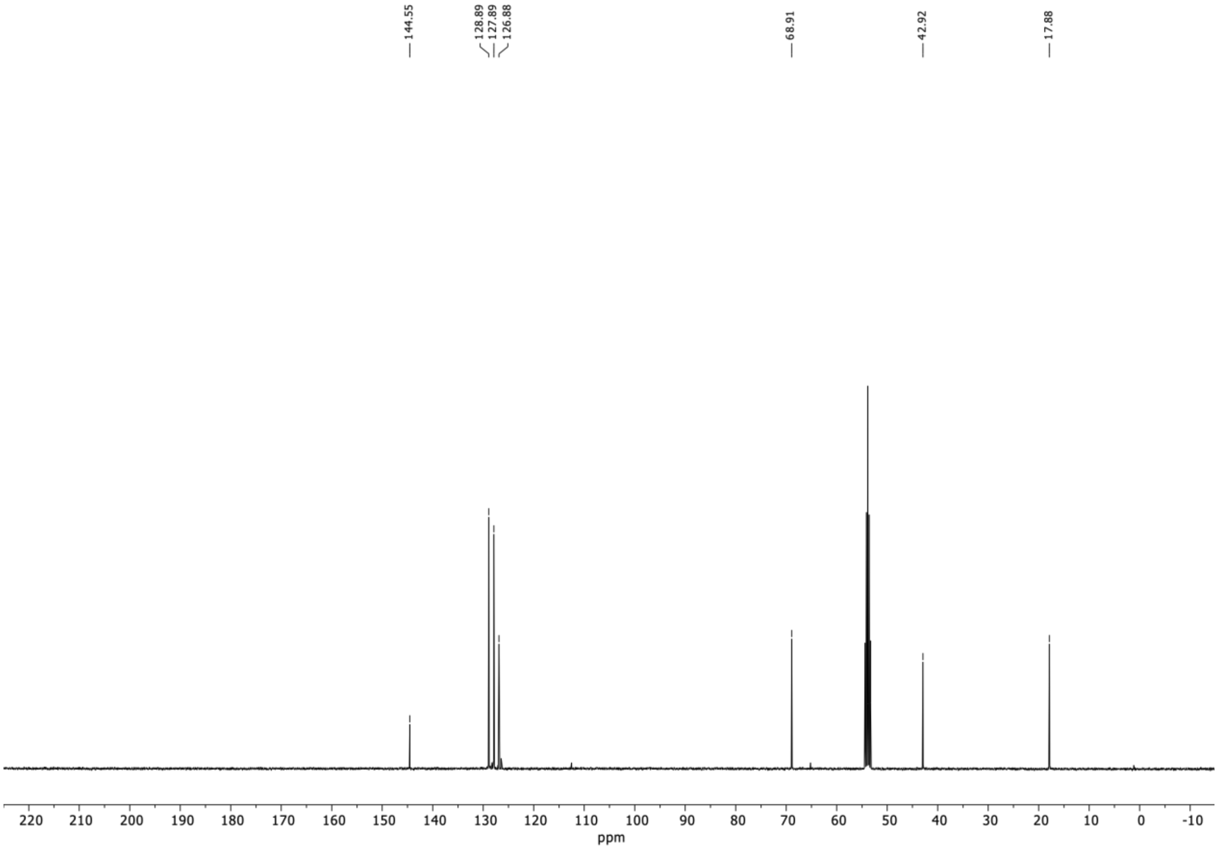

N-(Diphenylmethyl)-β-ethylbenzeneethanamine

^{1}H-NMR (400 MHz, $CDCl_3$)

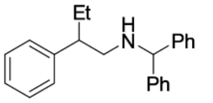

5b

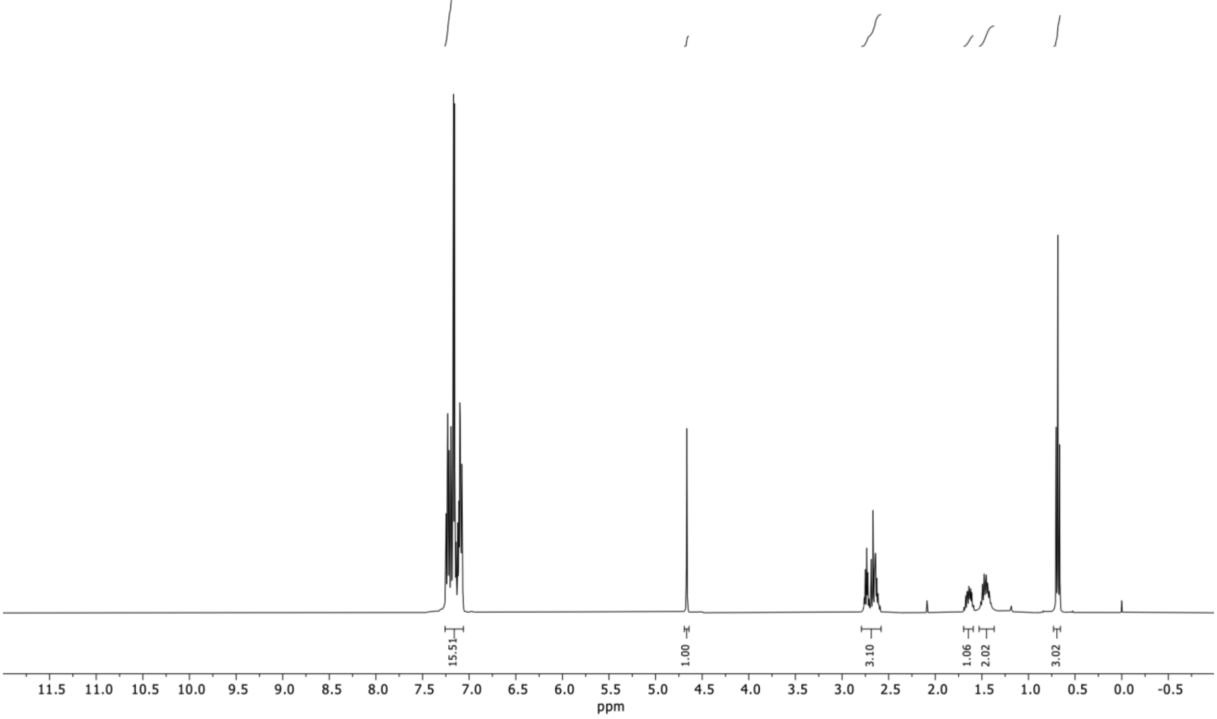

^{13}C-NMR (101 MHz, $CDCl_3$)

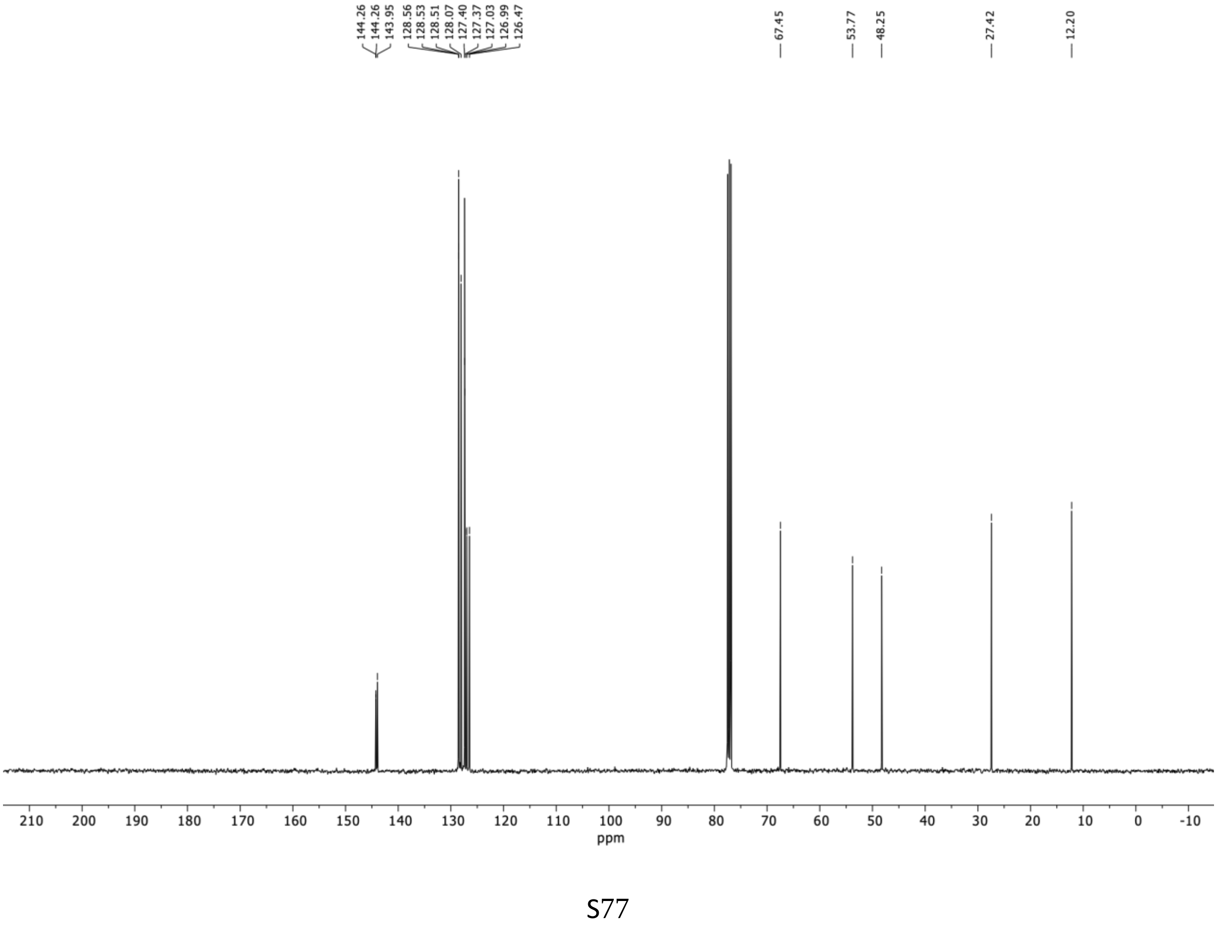

2-Phenylpropionic acid

^{1}H-NMR (400 MHz, $CDCl_3$)

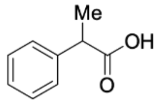

6a

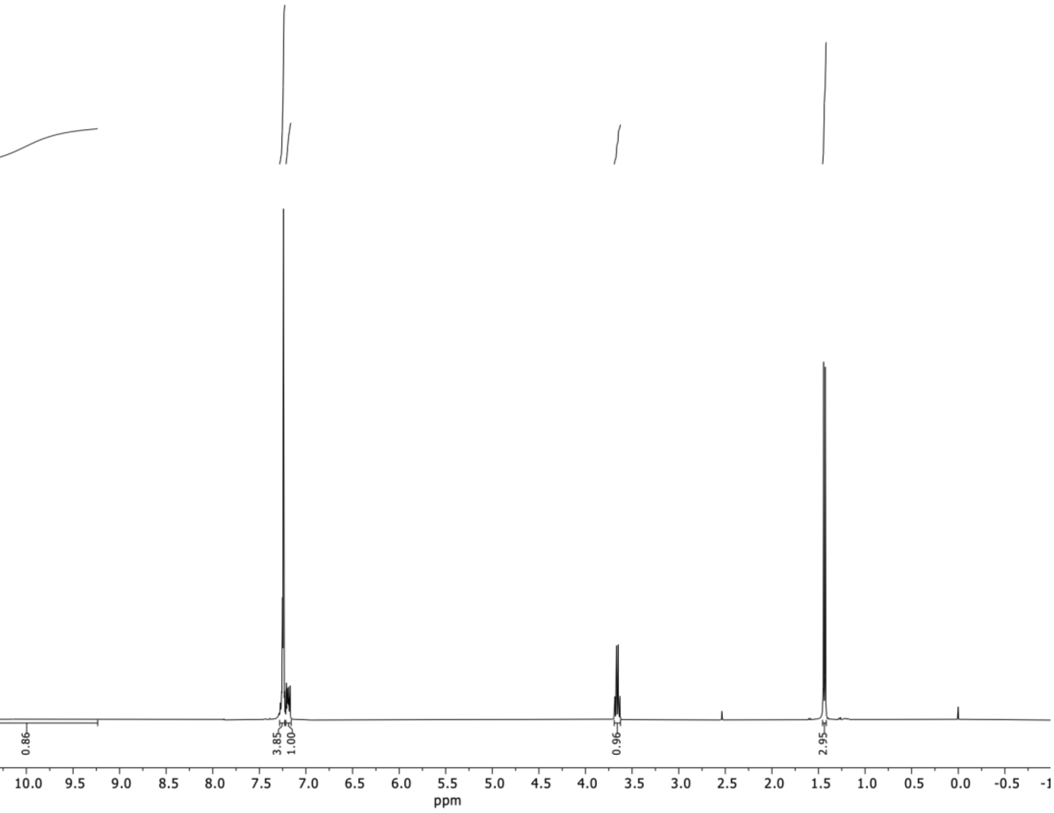

^{13}C-NMR (101 MHz, $CDCl_3$)

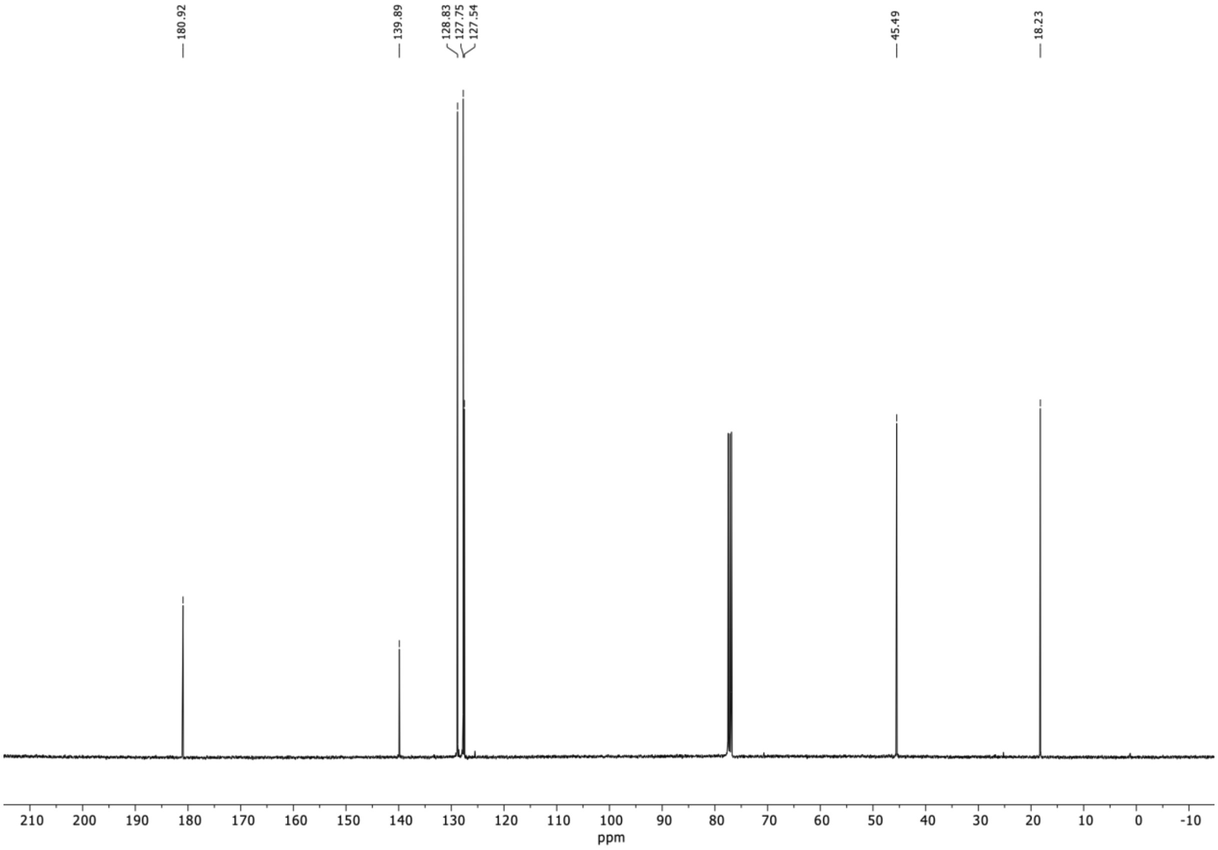

Ibuprofen

^{1}H-NMR (400 MHz, $CDCl_3$)

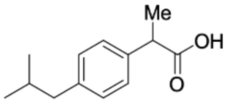

6t

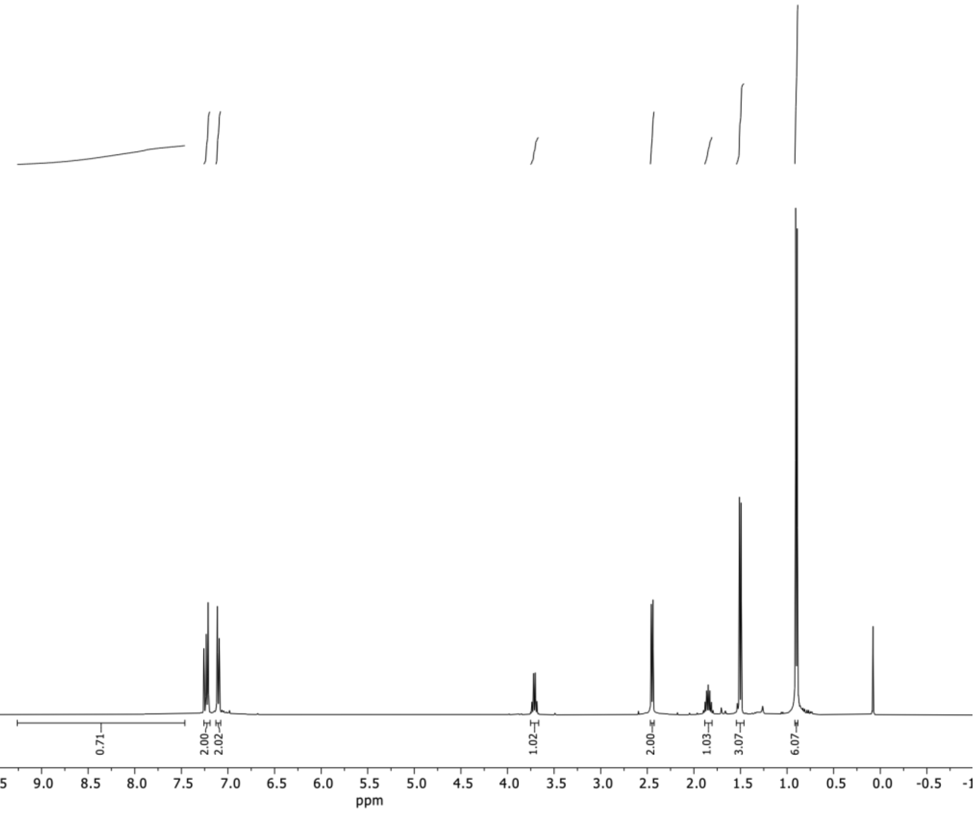

^{13}C-NMR (101 MHz, $CDCl_3$)

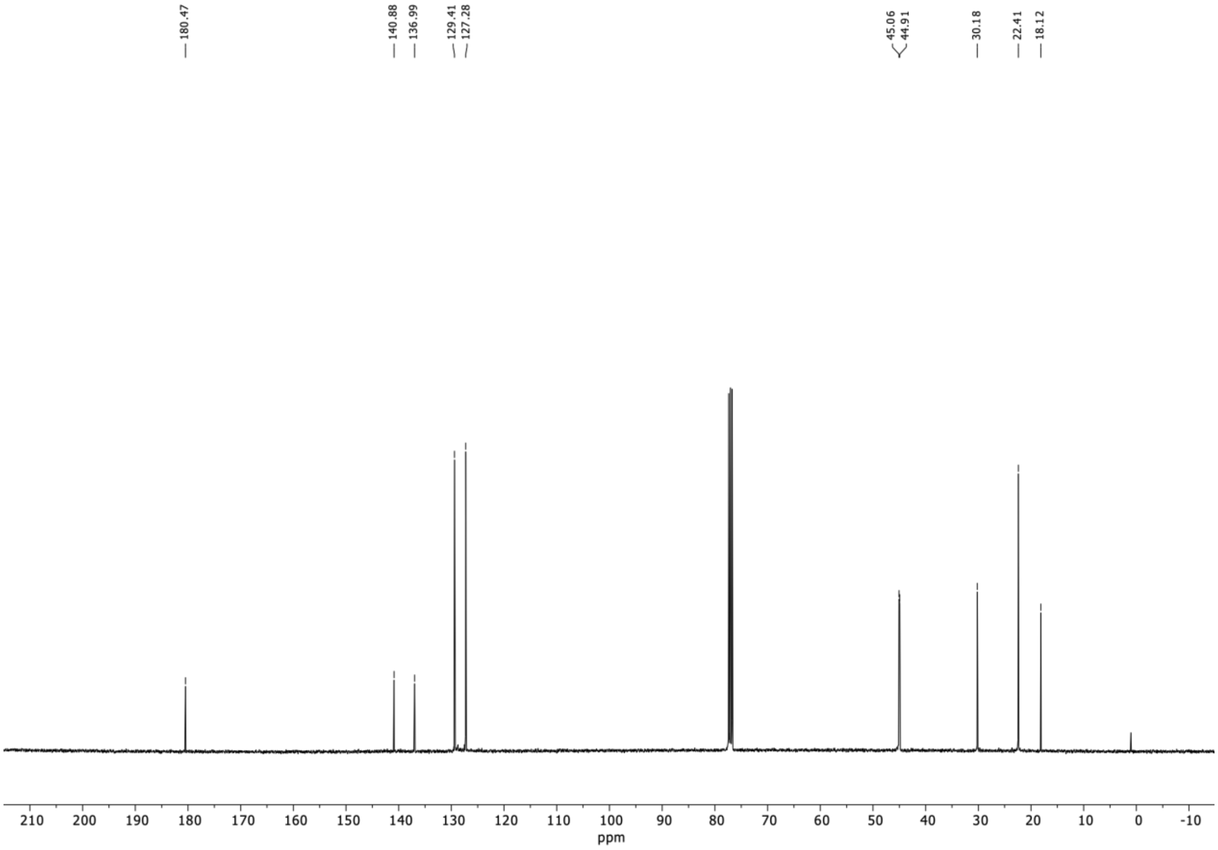

7. References

[1] A. Cabré, J. Cabezas-Giménez, G. Sciortino, G. Ujaque, X. Verdaguer, A. Lledós, A. Riera, "Mild Iridium-Catalysed Isomerization of Epoxides. Computational Insights and Application to the Synthesis of β-Alkyl Amines" *Adv. Synth. Catal.* **2019**, *361*, 3624–3631.

[2] F. Dressler, V. Öhler, C. Topp, P. R. Schreiner, "Organocatalytic, Chemoselective, and Stereospecific House–Meinwald Rearrangement of Trisubstituted Epoxides" *Synlett* **2024**, *35*, 1052–1056.

[3] N. G. Garrison, E. Holt, M. Wang, R. Rowshanpour, N. Kiame, W. Lam, F. Borukhova, T. Dudding, T. Lectka, "Complementary Tandem Reaction Manifolds and 'Switch Mechanisms' in the Reaction of Epoxides with Selectfluor" *J. Org. Chem.* **2024**, *89*, 15307–15311.

[4] A. Theodorou, G. N. Papadopoulos, C. G. Kokotos, "β-tert-Butyl aspartate as an organocatalyst for the asymmetric α-amination of α,α-disubstituted aldehydes" *Tetrahedron* **2013**, *69*, 5438–5443.

[5] H. Sun, C. Wang, R. Zhao, Z. Wang, H. Zhao, "Cobalt Supported on Ce-MOF-Derived CeO2 as a Catalyst for the Efficient Epoxidation of Styrene Under Aerobic Conditions" *Catal. Lett.* **2024**, *154*, 4649–4662.

[6] Y. Jeong, J. Ban, M. Lim, H. Rhee, "A Novel Synthesis of *N*-Sulfonylformamidines from *N*-Sulfonyl-sulfonamides" *Synthesis* **2018**, *50*, 1867–1874.

[7] H. Guo, Y. Dong, X. Zhou, X. Lu, X. Zhu, H. Que, Z. Wu, K. Cheng, X. Gu, "Synthesis and Bacteriostatic Activities of Modified Flutriafol Derivatives" *ACS Omega* **2019**, *4*, 9–14.

[8] D. J. Vyas, E. Larionov, C. Besnard, L. Guénée, C. Mazet, "Isomerization of Terminal Epoxides by a [Pd–H] Catalyst: A Combined Experimental and Theoretical Mechanistic Study" *J. Am. Chem. Soc.* **2013**, *135*, 6177–6183.

[9] N. Humbert, D. J. Vyas, C. Besnard, C. Mazet, "An air-stable cationic iridium hydride as a highly active and general catalyst for the isomerization of terminal epoxides" *Chem. Commun.* **2014**, *50*, 10592–10595.

[10] M. Vayer, S. Zhang, J. Moran, D. Lebœuf, "Rapid and Mild Metal-Free Reduction of Epoxides to Primary Alcohols Mediated by HFIP" *ACS Catal.* **2022**, *12*, 3309–3316.

[11] M. Cleij, A. Archelas, R. Furstoss, "Microbiological Transformations 43. Epoxide Hydrolases as Tools for the Synthesis of Enantiopure α-Methylstyrene Oxides: A New and Efficient Synthesis of (*S*)-Ibuprofen" *J. Org. Chem.* **1999**, *64*, 5029–5035.

[12] R. V. A. Orru, S. F. Mayer, W. Kroutil, K. Faber, "Chemoenzymatic deracemization of (±)-2,2-disubstituted oxiranes" *Tetrahedron* **1998**, *54*, 859–874.

[13] T. Sone, A. Yamaguchi, S. Matsunaga, M. Shibasaki, "Catalytic Asymmetric Synthesis of 2,2-Disubstituted Terminal Epoxides via Dimethyloxosulfonium Methylide Addition to Ketones" *J. Am. Chem. Soc.* **2008**, *130*, 10078–10079.

[14] M. R. Witten, E. N. Jacobsen, "A Simple Primary Amine Catalyst for Enantioselective α-Hydroxylations and α-Fluorinations of Branched Aldehydes" *Org. Lett.* **2015**, *17*, 2772–2775.

[15] R. Umeda, M. Muraki, Y. Nakamura, T. Tanaka, K. Kamiguchi, Y. Nishiyama, "Rhenium complex-catalyzed Meinwald rearrangement reactions of oxiranes" *Tetrahedron Lett.* **2017**, *58*, 2393–2395.

[16] M. Zhuang, H. Du, "Asymmetric rearrangement of racemic epoxides catalyzed by chiral Brønsted acids" *Org. Biomol. Chem.* **2013**, *11*, 1460.

[17] K.-J. Liu, Y.-L. Fu, L.-Y. Xie, C. Wu, W.-B. He, S. Peng, Z. Wang, W.-H. Bao, Z. Cao, X. Xu, W.-M. He, "Green and Efficient: Oxidation of Aldehydes to Carboxylic Acids and Acid Anhydrides with Air" *ACS Sustain. Chem. Eng.* **2018**, *6*, 4916–4921.
